# Subfamily C7 Raf‐like kinases MRK1, RAF26, and RAF39 regulate immune homeostasis and stomatal opening in *Arabidopsis thaliana*

**DOI:** 10.1111/nph.20198

**Published:** 2024-10-24

**Authors:** Márcia Gonçalves Dias, Bassem Doss, Anamika Rawat, Kristen R. Siegel, Tharika Mahathanthrige, Jan Sklenar, Maria Camila Rodriguez Gallo, Paul Derbyshire, Thakshila Dharmasena, Emma Cameron, R. Glen Uhrig, Cyril Zipfel, Frank L. H. Menke, Jacqueline Monaghan

**Affiliations:** ^1^ Department of Biology Queen's University Kingston ON K7L 3N6 Canada; ^2^ The Sainsbury Laboratory University of East Anglia, Norwich Research Park Norwich NR4 7UH UK; ^3^ Department of Biological Sciences University of Alberta Edmonton AB T6G 2E9 Canada; ^4^ Institute of Plant and Microbial Biology and Zurich‐Basel Plant Science Center University of Zurich Zurich 8008 Switzerland

**Keywords:** *Arabidopsis thaliana*, C7 Raf‐like kinase, CPK28, immunity, MRK1, RAF26, RAF39, stomata

## Abstract

- The calcium‐dependent protein kinase CPK28 regulates several stress pathways in multiple plant species. Here, we aimed to discover CPK28‐associated proteins in *Arabidopsis thaliana*.
- We used affinity‐based proteomics and identified several potential CPK28 binding partners, including the C7 Raf‐like kinases MRK1, RAF26, and RAF39. We used biochemistry, genetics, and physiological assays to gain insight into their function.
- We define redundant roles for these kinases in stomatal opening, immune‐triggered reactive oxygen species (ROS) production, and resistance to a bacterial pathogen. We report that CPK28 associates with and *trans*‐phosphorylates RAF26 and RAF39, and that MRK1, RAF26, and RAF39 are active kinases that localize to endomembranes. Although Raf‐like kinases share some features with mitogen‐activated protein kinase kinase kinases (MKKKs), we found that MRK1, RAF26, and RAF39 are unable to *trans*‐phosphorylate any of the 10 Arabidopsis mitogen‐activated protein kinase kinases (MKKs).
- Overall, our study suggests that C7 Raf‐like kinases associate with and are phosphorylated by CPK28, function redundantly in stomatal opening and immunity, and possess substrate specificities distinct from canonical MKKKs.

## Introduction

Plants encounter a variety of stressors in the environment that can negatively impact their growth and survival. The ability of plants to respond to danger signals such as drought, heat, cold, salinity, or pathogen attack, is critical to optimizing growth and reproduction in a changing environment. Lacking a humoral system, plants rely on innate and cell‐autonomous immune responses to fight against disease. Plant cell membranes contain high‐affinity transmembrane pattern recognition receptors (PRRs) that detect highly conserved microbial molecules known as microbe‐associated molecular patterns (MAMPs) or endogenous damage‐associated molecular patterns (DAMPs). Small peptides known as phytocytokines can also be secreted into the extracellular space, bind PRRs, and potentiate immune signaling (Gust *et al*., 2017; Segonzac & Monaghan, 2019). Plant PRRs are typically receptor kinases (RKs) or receptor‐like proteins (RPs). RKs contain a ligand‐binding ectodomain, a transmembrane domain, and an intracellular protein kinase domain, allowing them to both detect M/DAMPs and transduce the signal through substrate phosphorylation. In contrast to RKs, RPs lack a kinase domain, relying on regulatory RKs to relay the signal (DeFalco & Zipfel, 2021). The largest group of plant PRRs are the leucine‐rich repeat (LRR)‐containing RKs, which preferentially bind protein‐based M/DAMPs. The LRR‐RK FLAGELLIN SENSING 2 (FLS2) binds flg22, a 22‐amino acid epitope from the N‐terminus of bacterial flagellin, while the LRR‐RKs EF‐Tu RECEPTOR (EFR) and PEP‐RECEPTOR 1 and 2 (PEPR1/2) bind the 18‐amino acid epitope of elongation factor Tu (elf18) or endogenous peptide AtPep1, respectively (Zipfel *et al*., 2006; Chinchilla *et al*., 2007; Krol *et al*., 2010; Yamaguchi *et al*., 2010). Both RKs and RPs form heteromeric complexes with regulatory co‐receptors at the plasma membrane that typically engage in reciprocal *trans*‐phosphorylation, ultimately leading to receptor complex activation and intracellular signaling, including changes in ion flux, defense gene expression, and ROS production (Couto & Zipfel, 2016).

Many PRRs associate closely with several classes of intracellular protein kinases including receptor‐like cytoplasmic kinases (RLCKs) (Liang & Zhou, 2018), mitogen‐activated protein kinases (MAPKs) (Taj *et al*., 2010), and calcium‐dependent protein kinases (CDPKs) (Yip Delormel & Boudsocq, 2019). Here we focus on CPK28, a multi‐functional CDPK with roles in plant growth and development (Matschi *et al*., 2013), stress responses (Jin *et al*., 2017; Hu *et al*., 2021; S. Ding *et al*., 2022; Y. Ding *et al*., 2022), and defense against pathogens (Monaghan *et al*., 2014, 2015; Matschi *et al*., 2015). In immune signaling, CPK28 buffers the accumulation of the RLCK BOTRYTIS INDUCED KINASE 1 (BIK1), a common substrate of multiple receptors and a critical signaling node in plant immunity (Monaghan *et al*., 2014; J. Wang *et al*., 2018; DeFalco & Zipfel, 2021). CPK28 phosphorylates the E3 ubiquitin ligases PLANT U‐BOX 25 (PUB25) and PUB26, enhancing their ability to polyubiquitinate BIK1 resulting in its proteasomal turnover (J. Wang *et al*., 2018). The CPK28‐PUB25/26 regulatory module thus buffers BIK1 protein accumulation to optimize immune output (Dias *et al*., 2022).

In the current study, we aimed to identify additional CPK28 binding partners in Arabidopsis using a co‐immunoprecipitation‐based proteomics approach. We found that many protein kinases, including MIXED LINEAGE KINASE/RAF‐RELATED KINASE 1 (MRK1) copurify with CPK28‐YFP. Metazoan rapidly accelerated fibrosarcoma (Raf) kinases function in MAPK cascades. In mammals, the Ras–Raf–MEK–ERK pathway has been intensely studied and serves as a paradigm for membrane‐to‐nucleus signal transduction. In this pathway, binding of epidermal growth factor (EGF) to the EGF receptor at the plasma membrane results in activation and phosphorylation of its cytoplasmic kinase domain. This activates the GTPase Ras, which then binds to and activates Raf, which serves as a MAPK kinase kinase (MKKK), phosphorylating and activating a MAPK kinase (MKK), which then phosphorylates and activates a MAPK (originally named extracellular signal regulated kinase; ERK) (Terrell & Morrison, 2019). Reflecting the expansion of the protein kinase family in the plant kingdom, there are 20 MAPKs, 10 MKKs, and 80 MKKKs in Arabidopsis (González‐Coronel *et al*., 2021) – many more than in mammals. Despite their number, very little is known about MKKKs. Sequence homology defines three distinct subclasses known as MKKK, ZIK, and Raf‐like kinases. There are 48 Raf‐like kinases in Arabidopsis, divided into 11 subfamilies: B1–B4 and C1–C7 (Jonak *et al*., 2002; González‐Coronel *et al*., 2021). Phylogenetic analyses indicate that plant Raf‐like kinases do not cluster with metazoan MKKK or Raf kinases (Tang & Innes, 2002; Champion *et al*., 2004) and are considered a plant (Pl)‐specific family of tyrosine kinase‐like (TKL) proteins (TKL‐Pl‐4) (Lehti‐Shiu & Shiu, 2012). Despite this divergence, TKL‐Pl‐4 kinases share sequence features with metazoan Rafs and MLKs and may therefore function biochemically as MKKKs in MAPK cascades (Champion *et al*., 2004; Lehti‐Shiu & Shiu, 2012; González‐Coronel *et al*., 2021), however, this has not been comprehensively studied.

MRK1 belongs to the C7 subfamily of Raf‐like kinases, together with RAF26, RAF39, CONVERGENCE OF BLUE LIGHT AND CO_2_ 1 (CBC1), and CBC2 (Hiyama *et al*., 2017). CBC1 and CBC2 are highly expressed in guard cells and have established roles in light‐induced stomatal opening (Hiyama *et al*., 2017). While stomatal pores play a critical role in controlling gas exchange and water transpiration, they also represent a point of entry for microbial pathogens (Melotto *et al*., 2006), and immune‐induced stomatal closure is a well‐documented antimicrobial defense response (Melotto *et al*., 2017). Here, we define redundant roles for MRK1, RAF26, and RAF39 in the inhibition of immune‐triggered production of reactive oxygen species (ROS). We also demonstrate that MRK1, RAF26, and RAF39 function in stomatal opening, which correlates with enhanced resistance to a bacterial pathogen. We show that MRK1, RAF26, and RAF39 localize to endomembranes. We confirm that MRK1, RAF26, and RAF39 associate with CPK28 and that CPK28 can *trans*‐phosphorylate RAF26 and RAF39 *in vitro*. We further show that MRK1, RAF26, and RAF39 are active kinases that can auto‐phosphorylate *in vitro*. However, they are unable to *trans*‐phosphorylate any of the 10 Arabidopsis MKKs *in vitro*, suggesting that they possess substrate specificities distinct from canonical MKKKs. Overall, our study reveals that C7 Raf‐like kinases are CPK28 substrates that function redundantly in immune‐triggered ROS production and stomatal opening and provide evidence that they probably do not function as MKKKs.

## Materials and Methods

### Germplasm and plant growth conditions

*Arabidopsis thaliana* (L.) Heynh. insertion mutant lines were obtained from the Arabidopsis Biological Resource Centre (ABRC) and genotyped to homozygosity using gene‐specific primers in standard polymerase chain reactions (PCRs). Double and triple mutants were generated by crossing. To assess if the insertion mutations resulted in lower gene expression, target genes were amplified using quantitative reverse transcription PCR (qRT‐PCR) as described in Supporting Information Methods S1. Plants were grown in the Queen's University Phytotron as described in Methods S1. Stable transgenic lines were generated using *Agrobacterium tumefaciens*‐mediated floral dip (Clough & Bent, 1998). T_1_ lines were selected using resistance markers and genotyped to homozygosity in the T_3_ generation. Detailed information regarding all germplasm generated or used in this study, including primers used for genotyping and qRT‐PCR, is available in Table S1.

### Proteomics

Plant growth conditions, protein purification, immunoprecipitation, sample preparation, liquid chromatography followed by tandem mass spectrometry (LC‐MS/MS), and data analysis were previously described in full detail (Bender *et al*., 2017) and briefly in Methods S1. Phosphoproteomics is described in Methods S1.

### Molecular cloning

Clones were generated using various methods as outlined in detail in Methods S1. Information about all vectors used in this study, including previously published vectors, can be found in Table S1.

### *Agrobacterium*‐mediated transient expression in *Nicotiana benthamiana*

Binary vectors were transfected into *Agrobacterium tumefaciens* strain GV3101. Genes of interest were transiently expressed in *Nicotiana benthamiana* (Domin) for split‐luciferase complementation assays, confocal microscopy, and western blots as described in full detail in Methods S1.

### Immune assays

Immunogenic flg22, elf18, and AtPep1 peptides were synthesized by EZBiolab (Westfield, IN, USA). Immune‐induced ROS production was performed on 4‐ to 5‐wk‐old soil‐grown plants as previously described (Bredow *et al*., 2019). Immune‐induced activation of MAPKs was performed on 2‐wk‐old sterile seedlings as previously described (Monaghan *et al*., 2014). Bacterial infections and the assessment of stomatal apertures are described in full detail in Methods S1.

### Protein purification, immunoblotting, and *in vitro* kinase assays

All proteins were expressed and purified from *E. coli* strain BL21 (DE3) or BL21 (DE3) expressing Lambda phosphatase using the constructs outlined in Table S1 and the methods described in Methods S1. Autophosphorylation assays were performed using 2 μg kinase in a buffer containing 50 mM Tris–HCl (pH 8.0), 25 mM MgCl_2_, 25 mM MnCl_2_, 5 mM DTT, 5 μM ATP and 0.5–2 μCi γP^32^‐ATP. *Trans*‐phosphorylation assays used 2 μg kinase and 4 μg substrate in the same buffer. The buffer used in the *trans*‐phosphorylation assays with His_6_‐MBP‐CPK28 contained 500 μM CaCl_2_ and no MnCl_2_. All reactions were incubated for 60 min at 30°C. Reactions were stopped by adding 6× Laemmli buffer and heating at 80°C for 5 min. Proteins were separated by 10% SDS‐PAGE at 80 V for 30 min followed by 150 V for 1 h. The gels were then sandwiched between two sheets of transparency film, exposed to a storage phosphor screen (Molecular Dynamics) overnight and visualized using a Typhoon 8600 Imager (Molecular Dynamics, Amersham, UK). Gels were stained with Coomassie Brilliant Blue (CBB) R‐250 (MP Biomedicals; Irvine, CA, USA) or SimplyBlue SafeStain (Invitrogen; CBB G‐250) and scanned using an HP Officejet Pro 8620. All information pertaining to immunoblotting, including antibodies and titers used, is provided in Methods S1.

### Statistics

GraphPad Prism 8 or R were used to perform statistical tests on all quantitative data.

## Results

### Identification of CPK28‐associated proteins

To identify potential CPK28 interacting partners, we affinity‐purified CPK28 C‐terminally tagged with yellow fluorescent protein (YFP) from complementing *cpk28‐1/35S:CPK28‐YFP* transgenic lines (Matschi *et al*., 2013; Monaghan *et al*., 2014). We similarly affinity‐purified the CDPK CPK5‐YFP from Col‐0/35S:CPK5‐YFP (Dubiella *et al*., 2013), the plasma membrane‐localized protein NSL1‐YFP from *nsl1‐1/35S:NSL1‐YFP* (Holmes *et al*., 2021) and transmembrane protein Lti6B‐GFP from Col‐0/*35S:Lti6B‐GFP* (Cutler *et al*., 2000) lines to serve as comparative controls (Fig. S1A). Following immunoprecipitation with anti‐GFP microbeads, we performed liquid chromatography followed by tandem mass spectrometry (LC‐MS/MS) to identify peptides associated with CPK28, CPK5, NSL1, or Lti6B. We considered peptides that reliably co‐immunoprecipitated with CPK28‐YFP across independent trials, but did not co‐immunoprecipitate reliably with CPK5‐YFP, NSL1‐YFP, or Lti6B‐GFP as potential CPK28‐associated proteins (Table S2; Notes S1).

Notably, we identified peptides corresponding to experimentally validated CPK28‐associated proteins including the NADPH oxidase RESPIRATORY OXIDASE HOMOLOG D (RBOHD) (Monaghan *et al*., 2014) and calmodulin (Bender *et al*., 2017). We also identified peptides corresponding to other known CPK28‐associated proteins, including multiple isoforms of methionine adenosyltransferase (MAT) (Jin *et al*., 2017), ascorbate peroxidase (APX) (Hu *et al*., 2021) and glutamine synthase (GS) (Hu *et al*., 2021); however, peptides for all of these proteins were also observed in the NSL1‐YFP and Lti6B‐GFP controls (Table S2). It is important to consider that context‐specific associations between CPK28 and binding partners may not be captured from co‐immunoprecipitation‐based proteomics reflecting only a single time point during the plant growth cycle. Indeed, we did not recover peptides corresponding to several other experimentally validated CPK28 binding partners. While we did not identify peptides corresponding to the ARABIDOPSIS TOXICOS EN LEVADURA E3 ubiquitin ligases ATL6 or ATL31, which polyubiquitinate the active form of CPK28 resulting in its proteasomal degradation (Liu *et al*., 2022, 2023), nor any peptides corresponding to the E3 ligases PUB25 or PUB26, which are phosphorylated and partially activated by CPK28 (J. Wang *et al*., 2018), we did identify several components of the ubiquitin‐proteasome machinery (Table S2). Similarly, although we did not identify peptides corresponding to the RLCK BIK1, which associates with and reciprocally phosphorylates CPK28 (Monaghan *et al*., 2014; Bredow *et al*., 2021), we did identify five related RLCKs: BRASSINOSTEROID SIGNALING KINASE 1 (BSK1), CYTOSOLIC ABA RECEPTOR KINASE 7 (CARK7), MAZZA (MAZ/CARK5), PROLINE‐RICH EXTENSIN‐LIKE KINASE 1 (PERK1) and PERK15 (Table S2). In tomato (*Solanum lycopersicum*; *Sl*), *Sl*CPK28 associates with the phytosulfokine receptor *Sl*PSKR1 (S. Ding *et al*., 2022), and although we did not identify peptides corresponding to *At*PSKR1 in our dataset, we did identify 12 other RKs as putative CPK28 associated proteins: SUPPRESSOR OF BAK1‐INTERACTING KINASE 1 (SOBIR1), BAK1‐ASSOCIATING RECEPTOR KINASE 1 (BARK1), LEUCINE‐RICH REPEAT RECEPTOR‐LIKE KINASE WITH EXTRACELLULAR MALECTIN‐LIKE DOMAIN 1 (LMK1), NEMATODE‐INDUCED LRR‐RLK 2 (NILR2), LYSM RLK1‐INTERACTING KINASE 1 (LIK1), MDIS1‐INTERACTING RECEPTOR‐LIKE KINASE 2 (MIK2), FERONIA (FER), MEDOS 1 (MDS1), HERCULES RECEPTOR KINASE 4 (HERK4), WALL‐ASSOCIATED KINASE 1 (WAK1), WAK2, and L‐TYPE LECTIN RECEPTOR KINASE IV.1 (LECRK‐IV.1) (Table S2). CPK28 is a multi‐functional protein with roles in immune signaling (Monaghan *et al*., 2014; Monaghan *et al*., 2015; J. Wang *et al*., 2018), vegetative‐to‐reproductive stage transition (Matschi *et al*., 2013, 2015), temperature stress responses (Hu *et al*., 2021; Y. Ding *et al*., 2022), and more (Jin *et al*., 2017; S. Ding *et al*., 2022). The potential for CPK28 to associate with so many RKs and RLCKs at the plasma membrane may reflect this broad functionality.

### CPK28 associates with subfamily C7 Raf‐like protein kinases

**Fig. 1:**
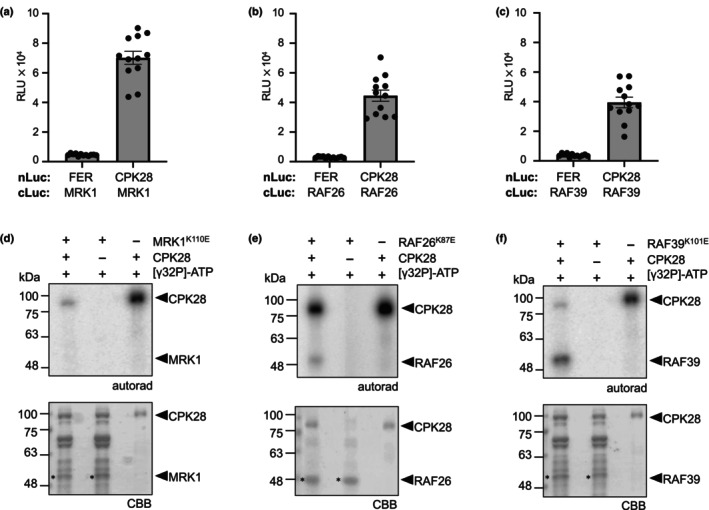
CPK28 associates with C7 Raf‐like kinases and phosphorylates RAF26 and RAF39. (a–c) Split‐luciferase (Luc) complementation assays with FER‐nLuc or CPK28‐nLuc and cLuc‐MRK1 (a), cLuc‐RAF26 (b), and cLuc‐RAF39 (c). Total photon counts are plotted as relative light units (RLU) after co‐expression of the respective proteins in *Nicotiana benthamiana*. Individual values are plotted from a representative experiment (*n* = 12) and are significantly different from each control (Student's unpaired *t*‐test; *P* < 0.0001); error bars represent SE. These assays were repeated over 4 times each by BD over a 12‐month period with similar results; representative data are shown. (d–f) *In vitro* kinase assays using His_6_‐MBP‐CPK28 as the kinase and catalytically inactive His_6_‐MRK1^K110E^ (d), His_6_‐RAF26^K87E^ (e), or His_6_‐RAF39^K101E^ (f) as substrates. Autoradiographs (autorad) indicate incorporation of γ^32^P and protein loading is indicated by poststaining with Coomassie Brilliant Blue (CBB). Asterisks indicate proteins of interest. Assays were performed more than 3 times each by MGD over a 6‐month period with similar results; representative data are shown. Cloning credits are provided in Supporting Information Table S1. All loci refer to gene names in *Arabidopsis thaliana*.

We identified 8 unique peptides corresponding to the Raf‐like protein kinase MRK1 as a putative CPK28‐associated protein (Tables 1, S2). To confirm that MRK1 associates with CPK28, we performed split‐luciferase complementation assays. In this method, one protein of interest is C‐terminally tagged with the N‐terminus of firefly luciferase (nLuc) and the other protein of interest is N‐terminally tagged with the C‐terminus of firefly luciferase (cLuc). If the two proteins associate, they reconstitute luciferase catalytic activity and emit light when provided with the substrate luciferin (H. Chen *et al*., 2008). We found that transiently co‐expressing CPK28‐HA‐nLuc and cLuc‐MRK1 in *N. benthamiana* reconstitutes the enzymatic function of luciferase. (Fig. 1a). This interaction appears to be specific, as luciferase function was not reconstituted when cLuc‐MRK1 was co‐expressed with nLuc fusion proteins of the transmembrane receptor kinases FER or WAK1, nor the protein phosphatase PP2A2 (Figs 1a, S1B). MRK1 is closely related to four other proteins, sharing 64–65% sequence identity at the amino acid level with RAF26 and RAF39 (78% identical), and CBC1 and CBC2 (Hiyama *et al*., 2017) (78% identical). Because of their similarity, we were curious if RAF26 or RAF39 could also associate with CPK28. We found that co‐expression of CPK28‐HA‐nLuc and cLuc‐RAF26 or cLuc‐RAF39 similarly reconstituted luciferase function, which was not observed when cLuc‐RAF26 or cLuc‐RAF39 were co‐expressed with FER‐HA‐nLuc, WAK1‐HA‐nLuc, or PP2A2‐HA‐nLuc (Figs 1b,c, S1C,D). In addition, we found that luciferase was reconstituted when CPK28‐HA‐nLuc was co‐expressed with cLuc‐CBC1 (Fig. S1E). We conclude that CPK28 can associate with C7 Raf‐like kinases *in vivo*.

**Table 1:** MRK1 peptides identified following affinity‐purification of CPK28‐YFP.

MRK1 peptide sequence	CPK28			CPK5			NSL1			Lti6B	
	R1	R2	R3	R1	R2	R3	R1	R2	R3	R1	R2
ASFEQEVAVWQK	–	–	1	–	–	–	–	–	–	–	–
ASFEQEVAVWQKLDHPNVTK	1	3	–	–	–	–	–	–	–	–	–
FIGASmGTSDLR	2	2	2	–	–	–	–	–	–	–	–
GLSYLHSK	2	–	2	–	–	–	–	–	–	–	–
GVYAGQEVAVK	2	1	–	–	–	–	–	–	–	–	–
IADFGVAR	3	6	2	–	2	2	–	–	–	–	–
LLEAIDTSK	2	2	2	–	–	–	–	–	–	–	–
NLRPEIPK	2	–	–	–	–	–	–	–	–	–	–
VEAQNPQDMTGETGTLGYmAPEVLEGKPYNR	–	1	1	–	–	–	–	–	–	–	–
VEAQNPQDmTGETGTLGYMAPEVLEGKPYNRK	–	–	–	–	–	–	1	–	–	–	–
VLDWGEDGYATPAETTALR	4	2	2	–	–	–	–	–	–	–	–

Total spectral counts for *Arabidopsis thaliana* MRK1 in each of the experimental replicates (R1–R3) for the bait CPK28‐YFP and negative controls CPK5‐YFP, NSL1‐YFP, and Lti6B‐GFP. Mascot search files were imported into Scaffold (2.5.1) and filtered with a 1% false discovery rate protein threshold. See Supporting Information Table S2 for more details.

### CPK28 phosphorylates RAF26 and RAF39

CPK28 displays strong kinase activity both *in vivo* (Matschi *et al*., 2013; Monaghan *et al*., 2015) and *in vitro* (Monaghan *et al*., 2014; Bender *et al*., 2017). Because they are able to associate, we hypothesized that *trans*‐phosphorylation may occur between CPK28 and C7 Raf‐like kinases. As protein kinases have well‐defined structures with a high level of conservation, it is possible to predict the location of the ATP‐binding lysine in the active site. We generated lysine (K)‐to‐glutamate (E) variants for MRK1, RAF26, and RAF39 to render them catalytically inactive in order to differentiate auto‐ from *trans*‐phosphorylation events. We then expressed and purified recombinant MRK1^K110E^, RAF26^K87E^, or RAF39^K101E^ N‐terminally tagged with His_6_, as well as CPK28 N‐terminally tagged with His_6_ and maltose‐binding protein (MBP) from *E. coli* and performed *in vitro* kinase assays using γP^32^‐ATP. While we were unable to detect CPK28‐mediated phosphorylation of MRK1^K110E^ (Fig. 1d), CPK28 was able to phosphorylate both RAF26^K87E^ (Fig. 1e) and RAF39^K101E^ (Fig. 1f), as indicated by the incorporation of γP^32^. To identify which residues are phosphorylated by CPK28, we performed additional kinase assays and analyzed phosphopeptides on RAF39^K101E^ by LC‐MS/MS. We identified a single phosphosite on RAF39 (Ser25) located in the N‐terminal variable domain (Fig. S2A). Notably, this residue is in a region of sequence conservation in RAF26 that is not shared with MRK1 (Fig. S2B), which may explain why CPK28 is not able to phosphorylate MRK1. We conclude that while CPK28 can associate with MRK1, RAF26, and RAF39 *in planta*, it is only able to *trans*‐phosphorylate RAF26 and RAF39 *in vitro*, suggesting that CPK28 possesses a high level of specificity for substrate choice.

### MRK1, RAF26, and RAF39 auto‐phosphorylate *in vitro*

**Fig. 2:**
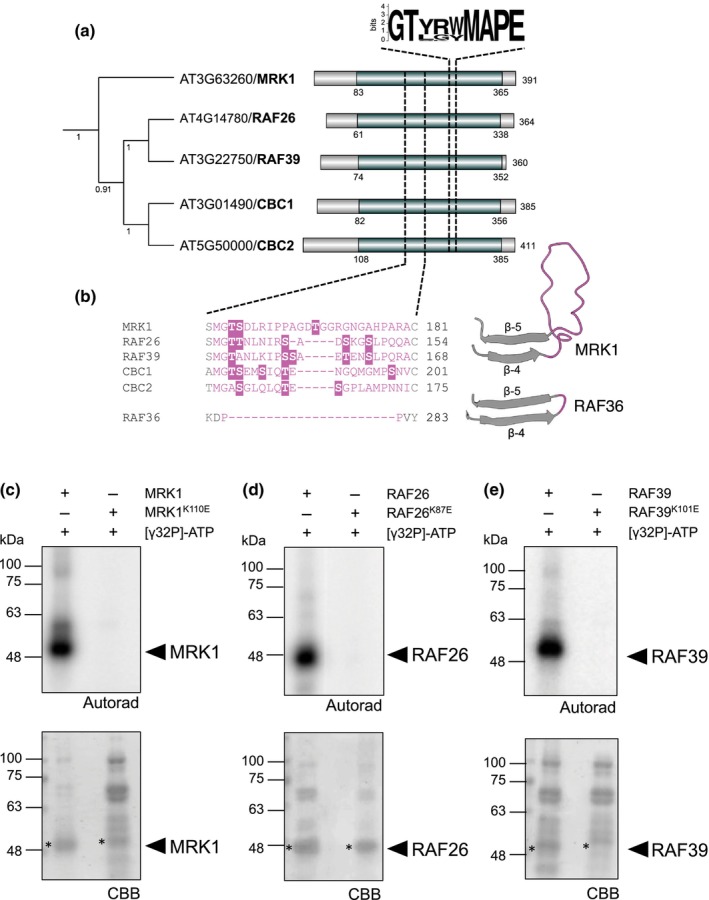
Subfamily C7 Raf‐like kinases have a unique extended loop in the N‐lobe of the kinase domain and auto‐phosphorylate *in vitro*. (a) Protein sequences from the C family of Raf‐like kinases were retrieved from The Arabidopsis Information Resource and a multiple sequence alignment was generated using the Muscle algorithm in MegaX (Kumar *et al*., 2018). The alignment was used to generate a neighbor‐joining tree with 1000 bootstraps; the tree shown here is just the C7 subfamily. The full C‐Raf family alignment was used to analyze the consensus signature motif G‐T‐x‐x‐[W/Y]‐M‐A‐P‐E and visualized here using WebLogo (Crooks *et al*., 2004). The protein kinase domains are labeled in green based on the Uniprot database; the protein lengths are indicated on the far right. (b) Multiple sequence alignment of the C7 Raf‐like kinases compared to RAF36 to illustrate the unique extension identified in C7 Raf‐like kinases, which forms an extended disordered loop between the β4 and β5 sheets of the N‐lobe (predictions shown for MRK1 and RAF36). Predicted protein structures were downloaded from Alphafold2 (Jumper *et al*., 2021) and visualized using ChimeraX (Pettersen *et al*., 2021). (c–e) *In vitro* kinase assays indicate that His_6_‐MRK1 (c), His_6_‐RAF26 (d), and (e) His_6_‐RAF39 are able to auto‐phosphorylate. Each assay included catalytically inactive variants as controls. Autoradiographs (autorad) indicate incorporation of γ^32^P and protein loading is indicated by poststaining with Coomassie Brilliant Blue (CBB). Asterisks indicate proteins of interest. JM performed the analysis in a and b; MGD performed the kinase assays in c–e at least three times over a 6‐month period with similar results and representative data is shown. Cloning credits are provided in Supporting Information Table S1. All loci refer to gene names in *Arabidopsis thaliana*.

C7 Raf‐like kinases MRK1, RAF26, RAF39, CBC1, and CBC2 all contain the TKL‐Pl‐4 consensus sequence G‐T‐x‐x‐[W/Y]‐M‐A‐P‐E in the kinase domain (Fig. 2a). Alphafold2 (Jumper *et al*., 2021) predictions suggest that C7 Raf‐like kinases adopt typical bilobal protein kinase structures; however, we noted the presence of an extended intrinsically disordered loop between the β4 and β5 sheets in the N‐lobe (Fig. 2b). A multiple sequence alignment of this area in all members of the Arabidopsis MKKK, Raf, and ZIK/WNK (With No Lysine) families revealed that while this loop typically contains 2–3 amino acids, it is uniquely extended to 21–26 residues in the C7‐Raf subfamily (Fig. 2b). Furthermore, this extension contains 3–5 phosphorylatable residues which may confer regulatory functions specific to the C7‐Raf subfamily.

Several MKKKs and Raf‐like kinases can auto‐phosphorylate *in vitro* (Ma *et al*., 2022). Indeed, recombinantly purified CBC1 and CBC2 N‐terminally tagged with GST are both capable of *in vitro* autophosphorylation (Hiyama *et al*., 2017). To determine if the other C7 Raf‐like kinases are similarly capable of autophosphorylation, we expressed and purified recombinant MRK1, RAF26, and RAF39 N‐terminally tagged with His_6_ from *E. coli* and performed autophosphorylation assays *in vitro* using γP^32^‐ATP. As controls, we included the catalytically inactive variants His_6_‐MRK1^K110E^, His_6_‐RAF26^K87E^, and His_6_‐RAF39^K101E^ to rule out the possibility of *trans*‐phosphorylation by co‐purified proteins. We found that the wild‐type variants of MRK1, RAF26, and RAF39 readily incorporated γP^32^, while the catalytically inactive variants did not, indicating that they possess kinase activity *in vitro* and can auto‐phosphorylate (Fig. 2c–e). We next assessed if MRK1, RAF26, or RAF39 are able to reciprocally phosphorylate CPK28. For this, we used the catalytically inactive CPK28^D188A^ variant (Matschi *et al*., 2013) as substrate in radiometric kinase assays. We did not observe incorporation γP^32^ (Fig. S3A–C), suggesting that CPK28 is not a substrate of MRK1, RAF26, or RAF39.

### MRK1, RAF26, and RAF39 localize to endomembranes

**Fig. 3:**
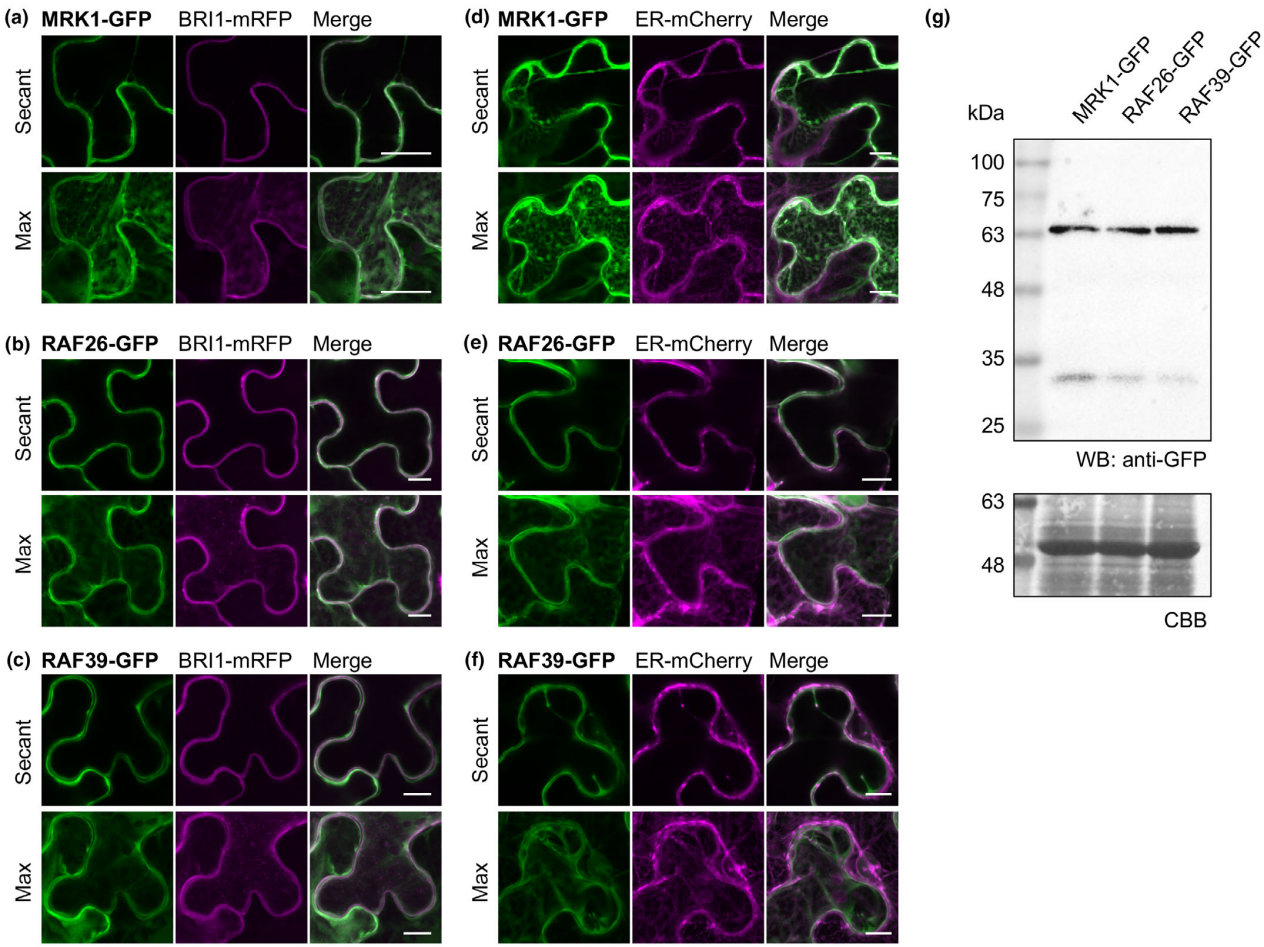
MRK1, RAF26, and RAF39 localize to endomembranes. (a–f) Confocal micrographs of green fluorescent protein (GFP)‐tagged MRK1, RAF26, and RAF39 co‐expressed with either red fluorescent protein (RFP)‐tagged BRI1 (a–c) or an mCherry‐tagged endoplasmic reticulum (ER) marker protein (d–f) in *Nicotiana benthamiana*. Maximum projections (max) are shown in the lower panels and single‐plane sections (secant) are shown in the upper panels. Bars: (a) 20 μm; (b–f) 10 μm. These assays were repeated 3 times by AR over a 6‐month period with similar results. (g) MRK1‐GFP, RAF26‐GFP, and RAF39‐GFP were expressed in *N. benthamiana*, proteins extracted and a western blot using anti‐GFP antibodies was performed. MRK1‐GFP (*c*. 69.6 kDa), RAF26‐GFP (*c*. 67.7 kDa), and RAF39‐GFP (*c*. 69.7 kDa) migrated to their expected sizes. Coomassie Brilliant Blue (CBB) of RuBisCO indicates loading. This experiment was repeated twice with identical results by MGD. Cloning credits are provided in Supporting Information Table S1. All loci refer to gene names in *Arabidopsis thaliana*.

Peptides matching MRK1, RAF26, RAF39, CBC1, and CBC2 have been identified in multiple Arabidopsis plasma membrane proteomes (Nelson *et al*., 2006; Benschop *et al*., 2007; Marmagne *et al*., 2007; Niittylä *et al*., 2007; Mitra *et al*., 2009; Kamal *et al*., 2020). Recently, CBC1‐GFP and CBC2‐GFP were found to localize to the cytosol in Arabidopsis guard cells, but can associate with another Raf‐like kinase, HIGH TEMPERATURE 1 (HT1) at the cell periphery in bimolecular fluorescence complementation experiments (Hiyama *et al*., 2017). To determine their subcellular localization, we cloned MRK1, RAF26, and RAF39 as C‐terminal translational fusions with green fluorescent protein (GFP), transiently expressed them in *N. benthamiana*, and visualized cellular fluorescence using confocal microscopy. As publicly available gene expression data indicates that *MRK1*, *RAF26*, *and RAF39* are expressed at low levels (Fig. S4A), we drove expression using the cauliflower mosaic virus (CaMV) *35S* promoter. Co‐expression with the plasma membrane marker BRASSINOSTEROID INSENSITIVE 1 (BRI1)‐mRFP (Saile *et al*., 2021) suggested that pools of MRK1‐GFP, RAF26‐GFP, and RAF39‐GFP localize to the plasma membrane; however, we also observed GFP throughout the cytosol (Fig. 3a–c). We found that MRK1‐GFP, RAF26‐GFP, and RAF39‐GFP co‐localized with endomembrane marker ER‐mCherry (Fig. 3d–f). While all proteins migrated to expected sizes in a western blot, we could also readily observe free GFP (Fig. 3g). Free GFP localizes nucleo‐cytoplasmically, and although we did not observe any signal in the nucleus, it is possible that the cytosolic signal we observe may not reflect the genuine localization of MRK1‐GFP, RAF26‐GFP, or RAF39‐GFP. In an attempt to clarify this, we generated stable transgenic lines in Col‐0 expressing MRK1‐GFP driven by its native promoter *pMRK1* or *35S*. Unfortunately, protein expression in Col‐0/*pMRK1:MRK1‐GFP* transgenics was very low compared to in Col‐0/*35S:MRK1‐GFP* (Fig. S4B), and we were unable to observe any signal when we attempted confocal imaging even in guard cells where *MRK1* expression is highest (Fig. S4A). We did, however, observe what appears to be endomembrane localization of MRK1‐GFP in Col‐0/*35S:MRK1‐GFP* (Fig. S4C); however, we are again unable to distinguish free GFP from MRK1‐GFP due to the presence of free GFP in the transgenic lines (Fig. S4B). Taken together, these results suggest that *35S‐*driven MRK1, RAF26, and RAF39 are able to localize to endomembranes *in planta* and may also broadly localize throughout the cytosol – locations that make it possible to associate with CPK28 at the plasma membrane. Because we were unable to identify classical lipidation motifs, secretory pathway sorting sequences, or endoplasmic reticulum (ER) retention signals in any of these proteins using the signal prediction tools WoLFPSORT (Horton *et al*., 2007) or SignalP 5.0 (Almagro Armenteros *et al*., 2019), we hypothesize that MRK1, RAF26, and RAF39 localize to endomembranes via additional binding partners.

### MRK1, RAF26, and RAF39 are genetically redundant regulators of immune‐triggered ROS

**Fig. 4:**
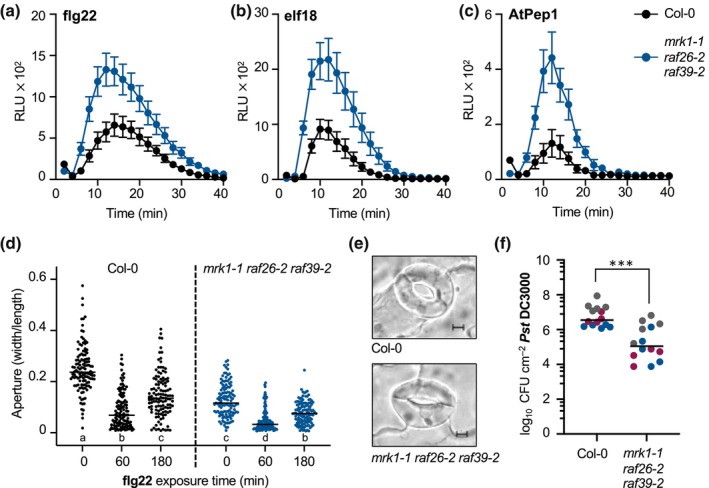
MRK1, RAF26, and RAF39 regulate immune homeostasis and stomatal opening. (a–c) Reactive oxygen species (ROS) production measured in relative light units (RLUs) after treatment with 100 nM flg22 (a), 100 nM elf18 (b), or 500 nM AtPep1 (c). Values represent means ± SE (*n* = 6–12). Data presented in a was collected by BD; data presented in b and c was collected by MGD. These assays were repeated several times by BD, MGD, EC, and JM over multiple years. (d) Stomatal apertures before (0 min) and following exposure to 1 μM flg22 (60, 180 min). Individual values are plotted and represent ratios of stomatal width : length. The straight line represents the mean (*n* = 120). Lower case letters indicate statistically significant groups, determined by a one‐way analysis of variance (ANOVA) followed by Tukey's *post hoc* test (*P* < 0.005). (e) Representative micrographs of stomata before flg22 treatment, showing visibly smaller apertures in *mrk1‐1 raf26‐2 raf39‐2* compared to Col‐0. Bar, 5 μm. Experiments in d and e were repeated 5 times by BD and AR over a 12‐month period; representative data collected by BD is shown. (f) Growth of *Pseudomonas syringae* pv *tomato* (*Pst*) isolate DC3000 3 days after spray‐inoculation. Data from three independent experimental replicates are plotted together, denoted by gray, blue, and magenta dots. Values are colony‐forming units (CFU) per leaf area (cm^2^) from 4–5 samples per genotype (each sample contains three leaf discs from three different infected plants). The line represents the mean (*n* = 14). Asterisks indicate significantly different groups, determined by a Student's unpaired *t*‐test (*P* < 0.0001). Data was collected by AR over a 12‐month period. Credits for genetic crosses and genotyping are provided in Supporting Information Table S1. All loci refer to gene names in *Arabidopsis thaliana*.

Because of their association with immune regulator CPK28, we hypothesized that C7 Raf‐like kinases may function in plant immune signaling. To test if C7 Raf‐like kinases are genetically required for plant immune responses, we obtained homozygous insertional mutants in *MRK1 (mrk1‐1)*, *RAF26 (raf26‐1*, *raf26‐2)*, *RAF39 (raf39‐1*, *raf39‐2)*, *CBC1 (cbc1‐1*, *cbc1‐2)*, and *CBC2 (cbc2‐3)* (Fig. S5A–B). We noted that leaf and rosette morphology in all mutants was comparable to wild‐type Col‐0 plants grown over multiple years in controlled environment chambers (Fig. S5C), although we did note slightly smaller growth in the *cbc1‐1 cbc2‐3* mutant as previously reported (Hiyama *et al*., 2017). Following the detection of immunogenic peptides by PRRs, RLCKs and CDPKs phosphorylate and activate the NADPH oxidase RBOHD, which catalyzes the production of a burst of apoplastic ROS within minutes (Yu *et al*., 2017). We found that the flg22‐induced ROS burst was not affected in *mrk1‐1*, *raf26‐1*, *raf26‐2*, *raf39‐1*, *raf39‐2*, *cbc1‐1*, *cbc1‐2*, or *cbc2‐3* single mutants (3/3 experimental replicates; Fig. S6A–D). As genetic redundancy was previously shown between *CBC1* and *CBC2* in blue light‐mediated stomatal opening (Hiyama *et al*., 2017), we also generated *cbc1‐1 cbc2‐3* and *raf26‐2 raf39‐2* double mutants. The flg22‐induced ROS burst was not affected in the *cbc1‐1 cbc2‐3* double mutant (12/13 experimental replicates; Fig. S6E), nor in the *raf26‐2 raf39‐2* double mutants (8/10 experimental replicates; Fig. S6F). We next generated a *mrk1‐1 raf26‐2 raf39‐2* triple mutant, as well as *mrk1‐1 raf26‐2* and *mrk1‐1 raf39‐2* double mutants. We consistently observed enhanced flg22‐triggered ROS in both the *mrk1‐1 raf26‐2* and *mrk1‐1 raf39‐2* double mutants (14/15 and 14/16 experimental replicates, respectively), as well as the *mrk1‐1 raf26‐2 raf39‐2* triple mutant (13/14 experimental replicates; Figs 4a, S6G,H). This response is not specific to flg22, as we also observed enhanced elf18‐ and AtPep1‐triggered ROS production in *mrk1‐1 raf26‐2*, *mrk1‐1 raf39‐2*, *mrk1‐1 raf26‐2 raf39‐2* (4/4 experimental replicates; Figs 4b,c, S6I,J). Importantly, we confirmed that these alleles result in lower expression of their target genes (Fig. S5B). To test if enhanced ROS confers enhanced disease resistance in *mrk1‐1 raf26‐2 raf39‐2*, we infected plants with the virulent bacterial pathogen *Pseudomonas syringae* pv *tomato* (*Pst*) DC3000 and counted *in planta* bacterial growth 3 days after syringe‐infiltration. We observed similar bacterial growth in *mrk1‐1 raf26‐2 raf39‐2* compared to Col‐0 (8/10 experimental replicates; Fig. S6K), suggesting that enhanced ROS production is not sufficient to confer enhanced resistance to *Pst* DC3000. Although we did not generate a *mrk1‐1 cbc1‐1 cbc2‐3* triple mutant, our results suggest that *MRK1* plays a key role in regulating immune‐triggered ROS, sharing unequal genetic redundancy with at least *RAF26* and *RAF39*.

### C7 Raf‐like kinases regulate stomatal aperture that correlates with enhanced resistance to a bacterial pathogen

The production of ROS is thought to provide direct antimicrobial activity in the apoplast, and also acts as a signaling molecule (Melotto *et al*., 2017). In guard cells, immune‐triggered ROS production has been linked to stomatal closure (Mersmann *et al*., 2010; Macho *et al*., 2012; Kadota *et al*., 2014; Hou *et al*., 2024). While stomatal pores play a critical role in controlling gas exchange for photosynthesis, open stomata can be seized as a point of entry for microbial pathogens; stomatal closure thus restricts access (Melotto *et al*., 2017). C7 Raf‐like kinases are expressed broadly throughout plant tissues, including in guard cells (Hayashi *et al*., 2017) (Fig. S4A). *CBC1* and *CBC2* are particularly strongly expressed in guard cells and have been shown to function redundantly in blue light and CO_2_‐mediated stomatal opening (Hiyama *et al*., 2017; Takahashi *et al*., 2022). With this in mind, we were interested to assess if MRK1, RAF26, or RAF39 similarly inhibit stomatal opening. To test this, we first confirmed altered stomatal aperture in the *cbc1‐1 cbc2‐3* double mutant compared to Col‐0 (Fig. S7A). Similar to *cbc1‐1 cbc2‐3*, we found that stomatal apertures were smaller than Col‐0 in *raf26‐2 raf39‐2*, *mrk1‐1 raf26‐2*, and *mrk1‐1 raf39‐2* double mutants (Fig. S7B–D), as well as *mrk1‐1 raf26‐2 raf39‐2* triple mutants (Fig. 4d,e). We did not observe any differences in stomatal apertures between Col‐0 and the *mrk1‐1*, *raf26‐2*, or *raf39‐2* single mutants (Fig. S7E). These results suggest that MRK1, RAF26, and RAF39 function redundantly in the regulation of stomatal opening.

During an immune response, stomata remain closed for some time but will reopen after the threat has passed (Melotto *et al*., 2006). Because of this, we were interested to assess if immune‐triggered stomatal closure and reopening is regulated by the C7 Raf‐like kinases. We thus treated plants with flg22 and measured stomatal apertures after 1 h and 3 h, to reflect the ‘closed’ and ‘reopening’ states in Col‐0. In all the double mutants, we observed strong flg22‐induced stomatal closure (Fig. S7A–D). Interestingly, stomata were closed more ‘tightly’ in the triple *mrk1‐1 raf26‐2 raf39‐2* mutant than in Col‐0 (3/3 experimental replicates; Fig. 4d). These data are congruent with previous work that indicated tighter stomatal closure in *cbc1 cbc2* mutants in response to abscisic acid (ABA) (Hiyama *et al*., 2017), and together support the model that C7 Raf kinases promote stomatal opening by derepressing stomatal closure. When we measured apertures after 3 h of exposure to flg22, we observed partial reopening in Col‐0 as well as the double and triple mutants (Figs 4d, S7A–D), suggesting that additional components regulate stomatal reopening following immune‐mediated closure.

We reasoned that smaller stomatal apertures capable of closing very tightly in response to an immune trigger might restrict pathogen entry into plant tissue. We therefore spray‐inoculated plants with *Pst* DC3000 to better mimic a natural infection and assessed *in planta* growth after 3 days. Here, we found that bacterial growth was reduced *c*. 10‐fold in *mrk1‐1 raf26‐2*, *mrk1‐1 raf39‐2*, *raf26‐2 raf39‐2*, and *mrk1‐1 raf26‐2 raf39‐2* compared to Col‐0 plants (3/3 experimental replicates; Figs 4f, S8A–C). Interestingly, we also observed reduced bacterial growth in *cbc1‐1 cbc2‐3* mutants when spray‐inoculated with *Pst* DC3000 (2/3 experimental replicates; Fig. S8D). Because *Pst* DC3000 produces the virulence factor coronatine which is capable of reopening stomata (Melotto *et al*., 2006), we next assessed the ability of the hypovirulent strain *Pst* DC3000 *COR‐* to infect the *mrk1‐1 raf26‐2 raf39‐2* mutant. While *Pst* DC3000 *COR‐* is less virulent on Col‐0 compared to *Pst* DC3000, we found no difference in bacterial growth between both strains when we spray‐infected *mrk1‐1 raf26‐2 raf39‐2* (4/4 experimental replicates; Fig. S8E). The ROS burst is an early immune response that occurs within 30 min following immune detection and before stomatal closure that occurs within 60 min following immune detection. Since immune‐triggered ROS is enhanced in *mrk1‐1 raf26‐2 raf39‐2* but not in *cbc1‐1 cbc2‐3* mutants, yet both display enhanced resistance to *Pst* DC3000, these results suggest that the smaller stomatal aperture observed in these mutants is likely responsible for the enhanced resistance to *Pst* DC3000.

### MRK1, RAF26, and RAF39 do not *trans*‐phosphorylate MKKs *in vitro*

The phosphorylation and activation of MAPKs occurs within minutes of PRR activation and in parallel with the apoplastic ROS burst (Yu *et al*., 2017). In Arabidopsis, at least two MAPK cascades are activated following MAMP perception, consisting of MAPKKK5‐MKK4/MKK5‐MPK3/6 (Asai *et al*., 2002; Yamada *et al*., 2016; Bi *et al*., 2018) or MEKK1‐MKK1/MKK2‐MPK4 (Ichimura *et al*., 2002; Ichimura *et al*., 2006; Nakagami *et al*., 2006; Suarez‐Rodriguez *et al*., 2007; M. Gao *et al*., 2008). MPK4 and MPK3/6 have diverse targets including WRKY transcription factors that drive expression of immune‐related genes and contribute to genetic reprogramming of the cell to combat infection (Mao *et al*., 2011; Guan *et al*., 2014). Because C7 Raf‐like kinases are predicted to function as MKKKs, and since *mrk1‐1 raf26‐2 raf39‐2* mutants displayed enhanced immune‐triggered ROS, we were interested to test if MRK1, RAF26, and RAF39 are involved in immune‐triggered MAPK activation. We thus assessed the phosphorylation status of MPK6, MPK3, and MPK4/MPK11 in Col‐0 compared to the *mrk1‐1 raf26‐2 raf39‐2* triple mutants following flg22 perception. Our results indicate that MAPK activation occurs similarly in Col‐0, *mrk1‐1 raf26‐2 raf39‐2*, and *cbc1‐1 cbc2‐3* mutants (Fig. S9A,B).

**Fig. 5:**
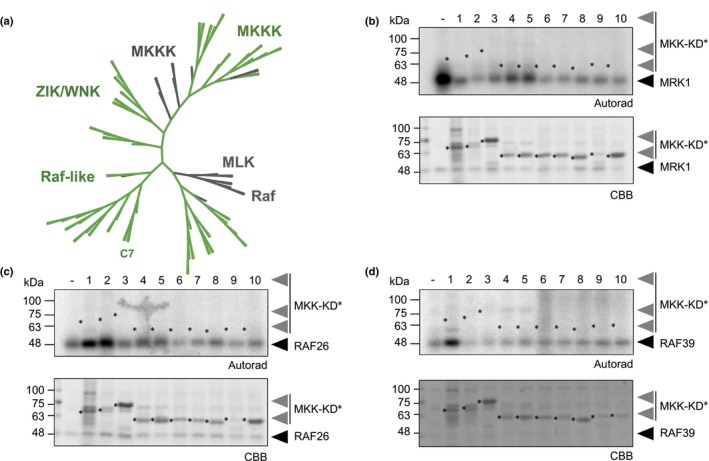
MRK1, RAF26, and RAF39 do not *trans*‐phosphorylate MKKs. (a) An unrooted phylogenetic tree of the *Arabidopsis thaliana* MKKK, ZIK/WNK, and Raf‐like subfamilies (green) together with *Homo sapiens* MKKK, MLK, and Raf kinases (gray). Subfamily C7 Raf‐like kinases are indicated. A multiple sequence alignment using the full‐length sequences of all proteins in the subfamilies was performed using Clustal Omega and the resulting neighbor‐joining phylogenetic tree was visualized using iTOL (Letunic & Bork, 2021); subfamilies are collapsed at the ends of nodes. Analysis performed by JM. (b–d) *In vitro* kinase assays indicate that His_6_‐MRK1 (b), His_6_‐RAF26 (c), and His_6_‐RAF39 (d) are unable to *trans*‐phosphorylate any of the 10 Arabidopsis MKKs N‐terminally tagged with glutathione *S*‐transferase (GST). Catalytically inactive MKK variants were used and are numbered as 1–10 for MKK1^K97E^, MKK2^K108E^, MKK3^K112E/K113E^, MKK4^K108E^, MKK5^K99E^, MKK6^K99E^, MKK7^K74E^, MKK8^K82E/K83E^, MKK9^K76E^, MKK10^K77E^, and are indicated by asterisks. Autoradiographs (autorad) indicate incorporation of γ^32^P and protein loading is indicated by poststaining with Coomassie Brilliant Blue (CBB). MGD and TD performed the assays three times over a 3‐month period with similar results and representative data is shown. Cloning credits are provided in Supporting Information Table S1. All loci in b–d refer to gene names in *A. thaliana*.

Although phylogenetically considered a subfamily of MKKKs, it is unclear if Raf‐like kinases function biochemically as kinases that phosphorylate MKKs in a MAPK cascade. Both the Raf‐like and ZIK/WNK subfamilies are divergent from canonical MKKKs, and neither cluster well with metazoan MKKK, Raf, or MLK proteins (Fig. 5a) (Champion *et al*., 2004). To clarify if MRK1, RAF26, and RAF39 can function as MKKKs, we tested if they can *trans*‐phosphorylate MKKs *in vitro*. There are 10 MKKs encoded in Arabidopsis that cluster into four subfamilies: subfamily A contains MKK1, MKK2, and MKK6; subfamily B contains MKK3; subfamily C contains MKK4 and MKK5; and subfamily D contains MKK7, MKK8, MKK9, and MKK10 (Jiang & Chu, 2018). We cloned and purified all 10 MKK proteins as catalytically inactive variants (replacing the ATP‐binding lysine with glutamate), N‐terminally tagged with GST. Kinase assays using γP^32^‐ATP indicate that none of MRK1, RAF26, or RAF39 are able to *trans*‐phosphorylate any of the 10 Arabidopsis MKKs *in vitro* (Fig. 5b–d). This suggests that they do not function biochemically as MKKKs in MAPK cascades.

## Discussion

Raf‐like kinases are a plant‐specific family with documented roles in ethylene signaling, osmotic stress, stomatal movement, and immunity (Fàbregas *et al*., 2020; González‐Coronel *et al*., 2021; Ma *et al*., 2022). Here, we focus on subfamily C7 Raf‐like kinases and provide evidence that they function in the regulation of stomatal aperture and immune signaling. Previous studies have described the redundant roles of CBC1 and CBC2 in stomatal opening (Hiyama *et al*., 2017; Hayashi *et al*., 2020; Takahashi *et al*., 2022), and we demonstrate similar function for the remaining C7 subfamily members MRK1, RAF26, and RAF39. Stomatal pores are formed between two guard cells that allow gas exchange and water transpiration to optimize plant growth but can also be co‐opted by pathogens to gain entry to plant tissues. The aperture of stomatal pores can adopt ‘open’ or ‘closed’ conformations, depending on environmental conditions that include both abiotic and biotic factors. For example, stomata adopt an open conformation under bright light or when levels of CO_2_ are limiting, thus driving photosynthesis. Conversely, stomata adopt a closed conformation in response to stress signals such as an increase in ABA or cytosolic Ca^2+^, or when levels of CO_2_ are sufficient (Shimazaki *et al*., 2007; Melotto *et al*., 2017). While there are pathway‐specific signaling mechanisms in place, opening and closing of stomata is ultimately controlled by changes in water potential that affect turgor pressure and membrane polarization/depolarization in guard cells.

In the presence of blue light, activated PHOTOTROPIN 1 and 2 (PHOT1/2) receptors facilitate H^+^‐ATPase‐mediated plasma membrane hyperpolarization, which results in stomatal opening and increased gas exchange at the stomatal pore (Kinoshita *et al*., 2001). Anion channels such as SLOW ANION CHANNEL ASSOCIATED 1 (SLAC1) are deactivated following blue light perception to inhibit membrane depolarization (Inoue & Kinoshita, 2017). In the presence of high intracellular CO_2_, the SnRK protein kinase OPEN STOMATA 1 (OST1) activates SLAC1 to trigger anion efflux and ultimately cause stomatal closure. To increase carbon uptake under low CO_2_, the subfamily C5 Raf‐like kinase HIGH LEAF TEMPERATURE 1 (HT1) inhibits OST1 activation and facilitates SLAC1 inactivation (Tian *et al*., 2015), which in turn enhances water uptake in guard cells and results in stomatal opening. Thus, exposure to blue light or low levels of CO_2_ results in stomatal opening in WT plants. However, these responses are defective in *cbc1 cbc2* double mutants, where stomata remain closed (Hiyama *et al*., 2017; Takahashi *et al*., 2022). Genetic, biochemical, and electrophysiological assays indicate that this phenotype is due to a break in the signaling pathway that enables blue light‐induced inhibition of S‐type anion channels such as SLAC1 (Hiyama *et al*., 2017). HT1 activates CBC1 by phosphorylation on several sites, including critical residues in the activation loop (Hiyama *et al*., 2017; Takahashi *et al*., 2022). In addition, CBC1 can be phosphorylated by PHOT1 *in vitro* and is rapidly phosphorylated in response to blue light *in vivo* (Hiyama *et al*., 2017), suggesting that CBC1/2 integrate signals from both blue light and CO_2_ pathways. Here, we show that *mrk1‐1 raf26‐2 raf39‐2* mutants display smaller stomatal aperture similar to *cbc1‐1 cbc2‐3*, suggesting that all C7 Raf‐like kinases participate in stomatal opening.

In response to immunogenic elicitors, activated PRRs in guard cells inhibit stomatal opening to restrict pathogen entry. Immune‐triggered ROS is thought to play a critical role in stomatal closure and have been proposed as secondary messengers. Multiple enzymes produce ROS apoplastically in response to biotic stress, including apoplastic peroxidases PRX33 and PRX34 (Daudi *et al*., 2012) as well as plasma membrane NADPH oxidases RBOHD and RBOHF (Torres *et al*., 2002). While RBOHD has been shown to be a substrate of BIK1 downstream of PRR activation (Kadota *et al*., 2014; Li *et al*., 2014), recent work indicates that RBOHF can also play a significant role in the generation of apoplastic ROS in guard cells (Arnaud *et al*., 2023). ROS are internalized via aquaporin PLASMA MEMBRANE INTRINSIC PROTEIN PIP2;1, which may also then facilitate water efflux and subsequent stomatal closure (Rodrigues *et al*., 2017; Hou *et al*., 2024). Here, we show that *mrk1‐1 raf26‐2 raf39‐2* mutants display enhanced apoplastic ROS production in response to immunogenic elicitors. It is important to note that we measured apoplastic ROS in leaf discs, which does not accurately reflect immune responses specifically occurring in guard cells. We are thus unable to draw a direct link between enhanced ROS and impaired stomatal movement. As *cbc1‐1 cbc2‐3* did not display enhanced ROS but still displayed impaired stomatal aperture, and as we observed smaller stomatal pores in *mrk1‐1 raf26‐2 raf39‐2* before immune induction, it is possible that the two phenotypes do not directly correlate.

Regulation of enzyme function through phosphorylation is well‐documented. While several phosphorylation sites on MRK1, RAF39, CBC1, and CBC2 have been curated from shotgun phosphoproteomics studies (Hoehenwarter *et al*., 2013; P. Wang *et al*., 2013; X. Wang *et al*., 2013; Wu *et al*., 2013; Roitinger *et al*., 2015; Marondedze *et al*., 2016; Nukarinen *et al*., 2016; Bhaskara *et al*., 2017; Al‐Momani *et al*., 2018; Song *et al*., 2018) as well as targeted studies (Hiyama *et al*., 2017; Takahashi *et al*., 2022), functional roles have so far only been assigned for Ser43 and Ser45 located at the N‐terminus of CBC1 (Hiyama *et al*., 2017). Here we show that CPK28 associates with C7 Raf‐like kinases *in vivo* and is able to phosphorylate RAF26 and RAF39 *in vitro*. We also mapped RAF39‐Ser25 as a CPK28‐mediated phosphorylation site. While a Thr residue is in a conserved position on RAF26, this is not the case for MRK1. Indeed, the N‐terminal regions are highly divergent between MRK1 and both RAF26 and RAF39 overall (Fig. S2B). While it remains possible that CPK28 may phosphorylate MRK1 *in vivo*, these results could reflect different regulatory mechanisms between highly similar proteins. Notably, the majority of other phosphosites on MRK1, RAF39, CBC1, and CBC2 map to their N‐termini in areas of low sequence conservation and low intrinsic order, with additional sites mapping to areas known to be involved in kinase activation, including the Gly‐rich and activation loops (Fig. S2B). It will be of interest to assess the functional role of N‐terminal phosphorylation on C7 Raf‐like kinases, as these are likely to represent areas of isoform‐specific regulation.

Publicly available gene expression data indicates that *CPK28* and all C7 Raf‐like genes are expressed in guard cells (Yang *et al*., 2008) (Fig. S4A), but *CBC1* and *CBC2* are the most highly expressed (Yang *et al*., 2008; Hiyama *et al*., 2017). A role for CPK28 in stomatal aperture has not yet been described, and previous work found no differences in flg22‐induced stomatal closure in two *CPK28‐OE* lines compared to WT plants (Monaghan *et al*., 2014). Here, we show that both *cbc1‐1 cbc2‐3* and *mrk1‐1 raf26‐2 raf39‐2* mutants are more resistant to spray‐inoculation of the bacterial pathogen *Pst* DC3000, which we consider may be a consequence of their smaller stomatal aperture. In addition, we found that while immune‐triggered ROS was unchanged in *cbc1‐1 cbc2‐3* mutants, the *mrk1‐1 raf26‐2 raf39‐2* mutants displayed enhanced ROS which suggests both unique and overlapping functions within this gene family. Neither *cbc1‐1 cbc2‐3* nor *mrk1‐1 raf26‐2 raf39‐2* displayed differences in flg22‐induced MAPK activation, which occurs in parallel to immune‐triggered ROS. Interestingly, viral‐induced gene silencing of the wheat (*Triticum aestivum; Ta*) ortholog of RAF39, *Ta*Raf46, similarly results in enhanced ROS accumulation, defense gene expression, and protection against the rust stripe pathogen *Puccinia striiformis* f. sp. *tritici* (*Pst*) isolates CYR23 and CYR31 (Wan *et al*., 2022). Conversely, overexpression of *Ta*Raf46 results in a loss of immune responses and enhanced susceptibility to *Pst* CYR23 (Wan *et al*., 2022). In addition, the cotton (*Gossypium hirsutum; Gh*) orthologs of RAF39, *Gh*MAP3K65 and *Gh*Raf39_1, function in stomatal opening and resistance to fungal pathogens *Rhizoctonia solani* and *Verticillium dahliae*, as well as bacterial pathogen *Ralstonia solanacerum* (Zhai *et al*., 2017; Mi *et al*., 2024). Although the functional relationship between CPK28 and MRK1, RAF26, and RAF39 is yet to be determined, it would be interesting to know if orthologs of CPK28 phosphorylate RAF39 in wheat and cotton.

To counteract immune responses and enable disease, pathogens secrete effector proteins that target key components of the immune system, including many protein kinases. In resistant plants, pathogen effectors are detected by intracellular nucleotide‐binding LRR receptors (NLRs) that can trigger localized programmed cell death when activated (El Kasmi, 2021). Interestingly, the *N. benthamiana* ortholog of RAF39 was identified in an *in planta* biotin ligase labeling assay as a protein in close proximity to the *Pst* DC3000 effector AvrPto at the plasma membrane (Conlan *et al*., 2018). Although a direct protein:protein association between AvrPto and *Nb*RAF39 was not confirmed, this raised the possibility that C7 Raf‐like kinases may be recruited or targeted by pathogen effectors. Recently, Pst27791, a serine‐rich effector protein from the stripe rust pathogen *Puccinia striiformis* f. sp. *tritici* isolate CYR23 (*Pst* CYR23) was shown to interact with and stabilize the accumulation of *Ta*Raf46 when heterologously expressed in *N. benthamiana* (Wan *et al*., 2022). Transgenic overexpression of Pst27791 in wheat results in enhanced susceptibility to *Pst* CYR23 only when *Ta*Raf46 is expressed, suggesting that Pst27791 requires *Ta*Raf46 for its virulence (Wan *et al*., 2022). In Arabidopsis, MRK1 is ubiquitinated on residue K342 (Grubb *et al*., 2021) and its protein abundance decreases by 50% following flg22 treatment (Benschop *et al*., 2007), which could reflect a derepression mechanism to enable immune signaling. In this scenario, effector‐mediated stabilization of C7 Raf‐like kinases could result in sustained repression of immune signaling to further pathogen spread. All of this evidence supports a role for C7 Raf‐like kinases as regulators of stomatal aperture and immune homeostasis in multiple plant species and may therefore be of interest to breeders.

Although Raf‐like kinases are considered a subfamily of MKKKs, their *bona fide* role as MKKKs has been debated (Champion *et al*., 2004; Ma *et al*., 2022). Canonical MKKKs phosphorylate MKKs at specific Ser/Thr residues located within a conserved S/T‐X_3‐5_‐S/T motif in the activation loop, which activates MKKs and allows them to phosphorylate MAPK targets (Rodriguez *et al*., 2010). Thus, to behave as a canonical MKKK in a MAPK cascade, a kinase would need to phosphorylate this consensus motif in an MKK. Some Raf‐like kinases can phosphorylate MKKs, but there is limited evidence that this phosphorylation occurs within the S/T‐X_3‐5_‐S/T motif. Recently, the subfamily C1 Raf‐like kinase RAF27 (also known as BLUE LIGHT‐DEPENDENT H+‐ATPASE PHOSPHORYLATION; BHP, or INTEGRIN‐LIKE KINASE 5; ILK5) was shown to associate with and phosphorylate MKK5, and that mutation of Thr215 and Ser221 in the S/T‐X_3‐5_‐S/T motif to nonphosphorylatable Ala residues reduced *trans*‐phosphorylation by RAF27/BHP/ILK5 (D. Kim *et al*., 2023). This suggests that RAF27/BHP/ILK5 phosphorylates MKK5 at the consensus motif as well as at other sites. Something similar was demonstrated for the subfamily B3 Raf‐like kinase MKKK δ‐1 (MKD1), which can *trans*‐phosphorylate both MKK1 and MKK5 *in vitro* (Asano *et al*., 2020). Mass spectrometry analysis indicated that while MKD1 phosphorylates MKK5 at Thr215 and Ser221 (within the S/T‐X_3‐5_‐S/T motif), it additionally phosphorylates MKK5 at Thr83 and MKK1 at Ser46 – N‐terminal residues that are not found within the activation loop consensus motif (Asano *et al*., 2020). In rice (*Oryza sativa; Os*), the subfamily C2 Raf‐like kinase *Os*ILA1 phosphorylates *Os*MKK4 on multiple N‐terminal residues including the key site Thr34, and not in the consensus motif (J. Chen *et al*., 2021). Additional evidence that Raf‐like kinases are atypical MKKKs comes from studies indicating that some can phosphorylate substrates that are not MKKs. For example, the subfamily B3 Raf‐like kinase CONSTITUTIVE TRIPLE RESPONSE 1 (CTR1), a well‐known kinase involved in ethylene signaling, phosphorylates ETHYLENE INSENSITIVE 2 (EIN2) at multiple residues (Ju *et al*., 2012). In addition, several other subfamily B Raf‐like kinases phosphorylate members of the sucrose nonfermenting‐1‐related protein kinase (SnRK) family in osmotic stress signaling (Saruhashi *et al*., 2015; Fàbregas *et al*., 2020; Katsuta *et al*., 2020; Lin *et al*., 2020; Soma *et al*., 2020; Takahashi *et al*., 2020), and the C5 Raf‐like kinase HT1 phosphorylates multiple sites on CBC1 including Thr256 and Ser280 in the activation loop (Hiyama *et al*., 2017; Takahashi *et al*., 2022). The B3 Raf‐like kinase ENHANCED DISEASE SUSCEPTIBILITY 1 (EDR1) negatively regulates immune signaling (Frye *et al*., 2001; Ma *et al*., 2022) and has been shown to associate with MKK4 and MKK5 (Zhao *et al*., 2014). Interestingly, *edr1* mutants accumulate less MKK4/MKK5 and MPK6/MPK3 proteins (Zhao *et al*., 2014), and EDR1 associates with E3 ligases KEEP ON GOING (KEG) (Wawrzynska *et al*., 2008; Gu & Innes, 2011) and ATL1 (Serrano *et al*., 2014). While it is unknown if any of these proteins are EDR1 substrates, KEG ubiquitinates MKK4/MKK5 resulting in their proteasomal turnover (C. Gao *et al*., 2021), suggesting that EDR1 regulates MKK accumulation via modulation of E3 ligases. The rice ortholog of EDR1 also negatively regulates immunity (Shen *et al*., 2011; J.‐A. Kim *et al*., 2003) and associates with but does not phosphorylate *Os*MKK10.2 (Ma *et al*., 2021). All together, these data suggest that EDR1 acts as a noncanonical MKKK in both rice and Arabidopsis. Notably, even some MEKK‐like MKKKs play noncanonical roles in signaling pathways. For example, MKKK7 is differentially phosphorylated in response to flg22 and attenuates flg22‐induced immune signaling including the activation of MPKs (Mithoe *et al*., 2016). Thus, it seems that the expansion of the MKKK family in plants has allowed for the evolution of novel functions. While it remains possible that certain Raf‐like kinases may operate as canonical MKKKs, it is evident that some Raf‐like kinases accept alternative substrate proteins. Our finding that MRK1, RAF26, and RAF39 cannot phosphorylate any of the 10 Arabidopsis MKKs *in vitro* suggests that they likely do not function as canonical MKKKs *in vivo*. An important next step will be to identify biologically relevant substrates for C7 Raf‐like kinases, of which currently none are known.

## Competing interests

None declared.

## Author contributions

JM and KRS designed the project. MGD, BD, AR, KRS, TM, TD, EC and JM generated materials, performed experiments, and analyzed results. JS and PD processed and analyzed CPK28‐associated proteins identified by proteomics, supervised by FLHM, JM, and CZ. MCRG processed and analyzed phosphoproteomics, supervised by RGU. Individual credits are included wherever possible in the figure captions and table legends. JM guided the work, secured funding and wrote the paper with input from all authors.

## Supporting information


**Fig. S1** CPK28 associates with MRK1, RAF26, RAF39, and CBC1.
**Fig. S2** Phosphorylation sites on C7 Raf‐like kinases.
**Fig. S3** MRK1, RAF26, and RAF39 do not phosphorylate CPK28 *in vitro*.
**Fig. S4** Analysis of MRK1‐GFP transgenic lines.
**Fig. S5** Genetic characterization of C7‐Raf loss‐of‐function mutants.
**Fig. S6** Immune‐triggered ROS production in single and double C7‐Raf mutants.
**Fig. S7** Stomatal aperture in single and double C7‐Raf mutants.
**Fig. S8** Infection assays with *Pst* DC3000 and *Pst* DC3000 *COR‐*.
**Fig. S9** Flg22‐triggered activation of MAPKs in C7 Raf‐like mutants.
**Methods S1** Full details pertaining to the materials and methods used in this study.

**Notes S1** Analysis of spectral counts of CPK28‐GFP enriched proteins.

**Table S1** Germplasm, clones, and primers generated in this study.

**Table S2** List of CPK28‐associated proteins identified by LC‐MS/MS.
Please note: Wiley is not responsible for the content or functionality of any Supporting Information supplied by the authors. Any queries (other than missing material) should be directed to the *New Phytologist* Central Office.

## Data Availability

Any novel germplasm or clones described in this article will be made freely available upon request. The person responsible for sharing materials is the author of correspondence jacqueline.monaghan@queensu.ca. Proteomics data has been deposited to the ProteomeXchange Consortium via the PRIDE (Perez‐Riverol *et al*., 2019; https://www.ebi.ac.uk/pride/) partner repository with the dataset identifiers PXD052803 and PXD055293.
