# Supplementary material for: Subfamily C7 Raf‐like kinases MRK1, RAF26, and RAF39 regulate immune homeostasis and stomatal opening in Arabidopsis thaliana
Source: New Phytol. 2024 Oct 24;244(6):2278–94. doi: 10.1111/nph.20198 (PMC11579443; doi:10.1111/nph.20198)
Supplement: Supplementary file 1 — Fig. S1 CPK28 associates with MRK1, RAF26, RAF39, and CBC1. Fig. S2 Phosphorylation sites on C7 Raf‐like kinases. Fig. S3 MRK1, RAF26, and RAF39 do not phosphorylate CPK28 in vitro. Fig. S4 Analysis of MRK1‐GFP transgenic lines. Fig. S5 Genetic characterization of C7‐Raf loss‐of‐function mutants. Fig. S6 Immune‐triggered ROS production in single and double C7‐Raf mutants. Fig. S7 Stomatal aperture in single and double C7‐Raf mutants. Fig. S8 Infection assays with Pst DC3000 and Pst DC3000 COR‐. Fig. S9 Flg22‐triggered activation of MAPKs in C7 Raf‐like mutants. Methods S1 Full details pertaining to the materials and methods used in this study. [file NPH-244-2278-s004.pdf]

New Phytologist Supporting Information

Article title: Subfamily C7 Raf-like kinases MRK1, RAF26, and RAF39 regulate immune homeostasis and stomatal opening in *Arabidopsis thaliana.*

Authors: Márcia Gonçalves Dias, Bassem Doss, Anamika Rawat, Kristen R. Siegel, Tharika Mahathanthrige, Jan Sklenar, Maria Camila Rodriguez Gallo, Paul Derbyshire, Thakshila Dharmasena, Emma Cameron, R. Glen Uhrig, Cyril Zipfel, Frank L.H. Menke, and Jacqueline Monaghan.

Article acceptance date: 26 September 2024.

The following Supporting Information is available for this article:

Fig. S1 - CPK28 associates with MRK1, RAF26, RAF39, and CBC1.

Fig. S2 - Phosphorylation sites on C7 Raf-like kinases.

Fig. S3 - MRK1, RAF26, and RAF39 do not phosphorylate CPK28 *in vitro*.

Fig. S4 - Analysis of MRK1-GFP transgenic lines.

Fig. S5 - Genetic characterization of C7-Raf loss-of-function mutants.

Fig. S6 - Immune-triggered ROS production in single and double C7-Raf mutants.

Fig. S7 - Stomatal aperture in single and double C7-Raf mutants.

Fig. S8 - Infection assays with *Pst* DC3000 and *Pst* DC3000 *COR-*.

Fig. S9 - Flg22-triggered activation of MAPKs in C7 Raf-like mutants.

Methods S1 - Full details pertaining to the materials and methods used in this study.

Table S1 - Germplasm, clones, and primers generated in this study.
Provided as a separate Excel sheet.

Table S2 - List of CPK28-associated proteins identified by LC-MS/MS.
Provided as a separate Excel sheet.

Notes S1 - Analysis of spectral counts of CPK28-GFP enriched proteins. Fold-change ratios of spectral counts between CPK28, CPK5, NSL1, or PM-GFP. Data used is the same as **Supporting Information Table S2**.
Provided as a separate PDF.

Supporting Information - Figures

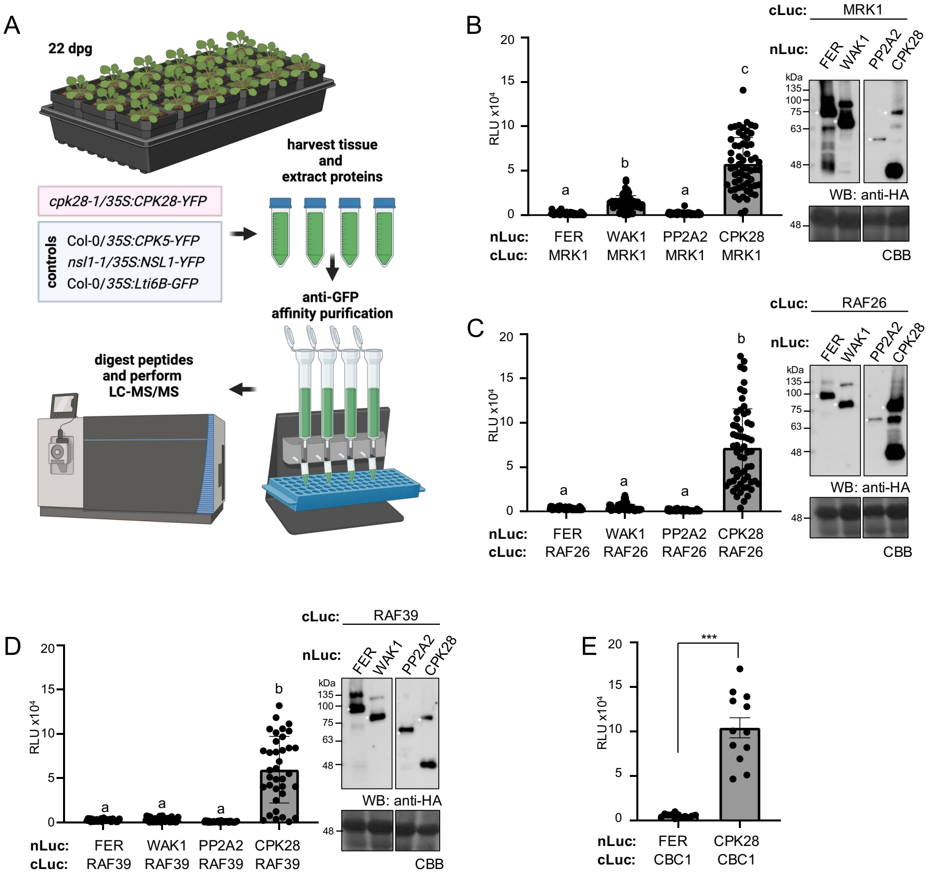

Figure S1. CPK28 associates with MRK1, RAF26, RAF39, and CBC1.

(A) Schematic describing the identification of CPK28-associated proteins in *A. thaliana*. This figure was created with Biorender.com by JM. **(B-D)** Split-luciferase (Luc) complementation assays with cLuc-MRK1 **(B)**, cLuc-RAF26 **(C)**, or cLuc-RAF39 **(D)** with FER-HA-nLuc, WAK1-HA-nLuc, PP2A2-HA-nLuc, or CPK28-HA-nLuc. Total photon counts are plotted as relative light units (RLU) after co-expression of the respective proteins in *N. benthamiana*. Individual values are plotted from 3 independent experiments (n=36). Lower case letters indicate statistically significant groups, determined by a one-way analysis of variance (ANOVA) followed by Tukey's post-hoc test (p<0.0001). These assays were conducted by MDG, TM, and BD. Anti-HA western blots indicate that all hemagglutinin (HA)-nLuc-tagged proteins are expressed; protein loading is indicated by post-staining with Coomassie Brilliant Blue (CBB). Asterisks indicate the expected molecular weight of the proteins. Westerns were conducted three times by MDG with similar results. **(E)** Split-luciferase complementation assays with FER-HA-nLuc or CPK28-HA-nLuc and cLuc-CBC1. Total photon counts are plotted as relative light units (RLU) after co-expression of the respective proteins in *N. benthamiana*. Individual values are plotted from a representative experiment (n=12) and are significantly different from each control (Student's unpaired t-test; p<0.0001). These assays were repeated over 4 times each by TM over a 6-month period with similar results. Cloning credits available in **Supporting Information Table S1**. All loci refer to gene names in *A. thaliana*.

A

Protein	Phosphopeptide	Phosphosite
**RAF39 (At3g22750.1)**	GADGNN**[pS]**KKDMIFR	S25

B

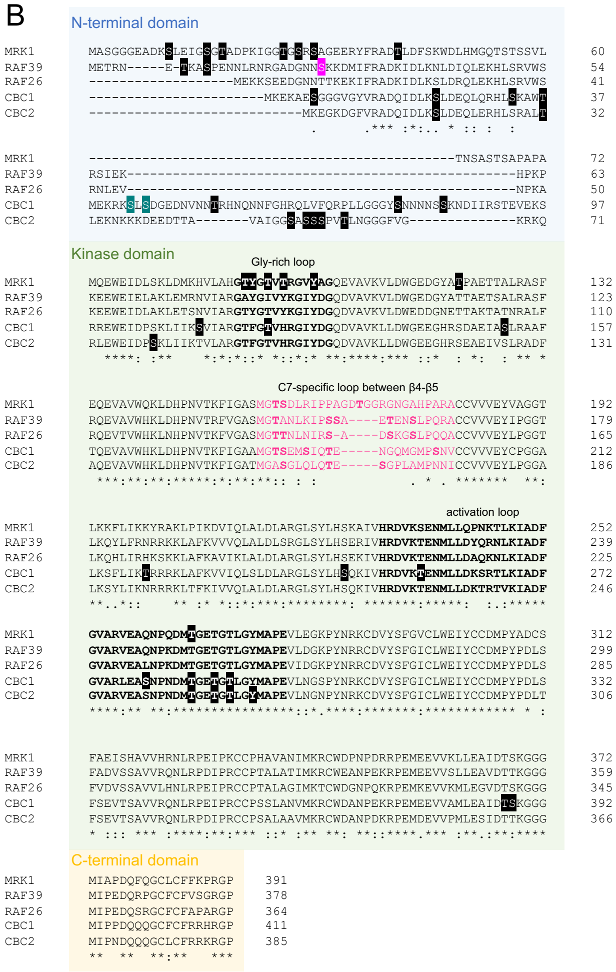

Figure S2. Phosphorylation sites on C7 Raf-like kinases.

(A) CPK28 phosphorylates RAF39 on Ser25. This RAF39 phosphopeptide was identified in 4/4 independent replicates and not found in control samples with ATP but without CPK28. Kinase assays performed by MGD; trypsin digests and liquid chromatography followed by tandem mass spectrometry (LC-MS/MS) performed by MCRG. **(B)** Multiple sequence alignment of the C7 Raf-like kinases indicating the general locations of the N-terminal (blue), kinase (green), and C-terminal (yellow) domains. CPK28-mediated phosphorylation site Ser25 on RAF39 is outlined in magenta. Residues outlined in black have been identified as phosphosites curated in online databases based on the following studies (Bhaskara et al. 2017; Al-Momani et al. 2018; Marondedze et al. 2016; Roitinger et al. 2015; Nukarinen et al. 2016; Hoehenwarter et al. 2013; Song et al. 2018; P. Wang et al. 2013; X. Wang et al. 2013; Wu et al. 2013; Hiyama et al. 2017; Takahashi et al. 2022). Ser43 and Ser45 of CBC1 are outlined in turquoise as they have been functionally assessed (Hiyama et al. 2017). Alignment was generated using Clustal Omega; asterisks indicate identical amino acids and colons indicate similar amino acids. Analysis by BD and JM. All loci refer to gene names in *A. thaliana*.

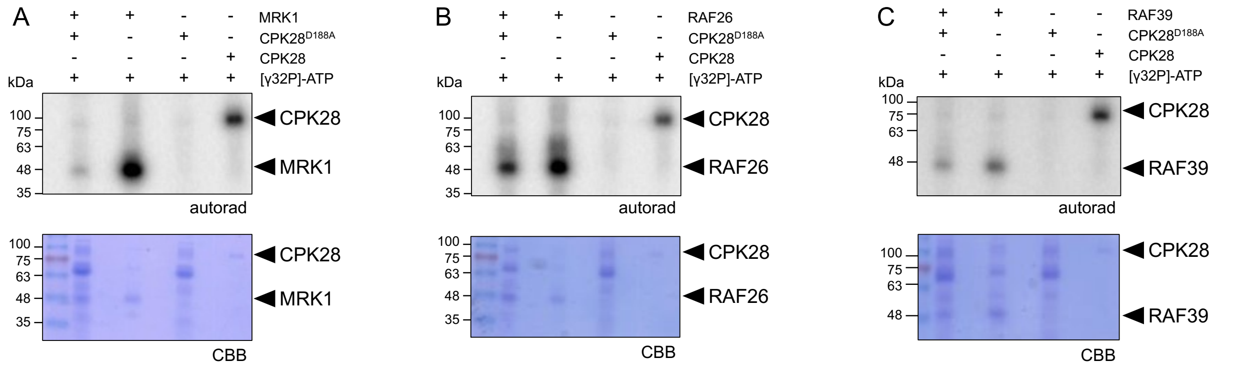

Figure S3. MRK1, RAF26, and RAF39 do not phosphorylate CPK28 *in vitro.*

In vitro kinase assays using His$_6$-MRK1 (A), His$_6$-RAF26 (B) and His$_6$-RAF39 **(C)** as the kinases and catalytically inactive His$_6$-MBP-CPK28^{D188A} as substrate. His$_6$-MBP-CPK28 was used as a positive control of CPK28 autophosphorylation. Autoradiographs (autorad) indicate incorporation of $\gamma^{32}P$ and protein loading is indicated by post-staining with Coomassie Brilliant Blue (CBB). Assays were performed more than 3 times each by MGD over a 6-month period with similar results; representative data are shown. Cloning credits are provided in Supporting Information Table S1. All loci refer to gene names in *A. thaliana*.

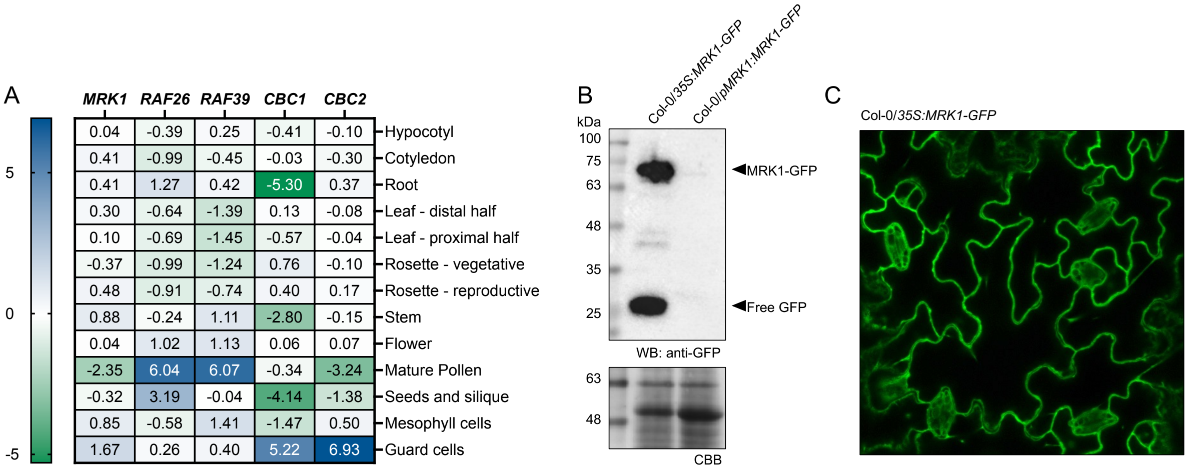

Figure S4. Analysis of MRK1-GFP transgenic lines.

(A) Heat map of relative expression levels of C7 Raf-like kinases throughout Arabidopsis development and in specific cell types. Data was curated from the ePlant database (Waese et al. 2017) from published datasets (Yang et al. 2008; Craigon et al. 2004). **(B)** Anti-green fluorescent protein (GFP) western blot (WB) of proteins extracted from the indicated genotypes (MRK1-GFP is ~69.6 kDa, while free GFP is ~25 kDa). Protein loading is indicated by post-straining with Coomassie Brilliant Blue (CBB). This experiment was repeated 3 times by MGD and KRS; a representative blot by MGD is shown. **(C)** Confocal micrograph of epidermal cells from the cotyledons of a Col-0/35S:MRK1-GFP transgenic line. This localization pattern was observed in multiple samples from independent biological replicates by KRS and MGD; a representative micrograph collected by KRS is shown. All germplasm is *A. thaliana*.

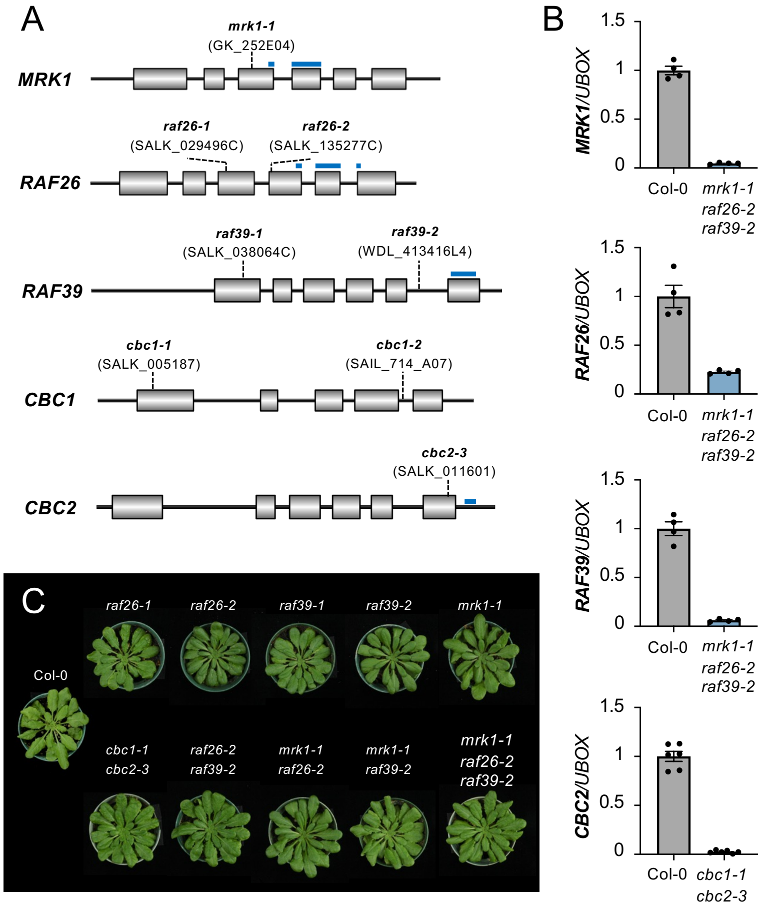

Figure S5. Genetic characterization of C7-Raf loss-of-function mutants.

(A) Schematic representation, drawn to scale, of subfamily C7 genes, indicating exons (boxes), untranslated regions (lines), and the location of T-DNA insertion alleles. Genomic information was retrieved from The Arabidopsis Information Resource by KRS and JM. Lines were genotyped to homozygosity by KRS, EC, JM, BD, and AR as described in **Supporting Information Table S1**. The amplicons analyzed in **C** are indicated by blue lines. **(B)** Quantitative real-time qRT-PCR of target genes relative to *UBOX* and normalized to expression in Col-0. Means for 3-4 independent biological replicates are shown +/- standard error of the mean. Data for *MRK1, RAF26,* and *RAF39* expression in *mrk1-1 raf26-2 raf39-2* was collected by AR, while data for *CBC2* expression in *cbc1-1 cbc2-3* was collected by KRS. Lower expression of *CBC1* has already been confirmed for the *cbc1-1* allele (SALK_005187) (Hayashi et al. 2020). Primers for genotyping and qRT-PCR are provided in **Supporting Information Table S1**. **(C)** Photographs of representative plants of each genotype after 5 weeks of growth on soil under short-day conditions. Photographs taken by MGD. All germplasm is *A. thaliana*.

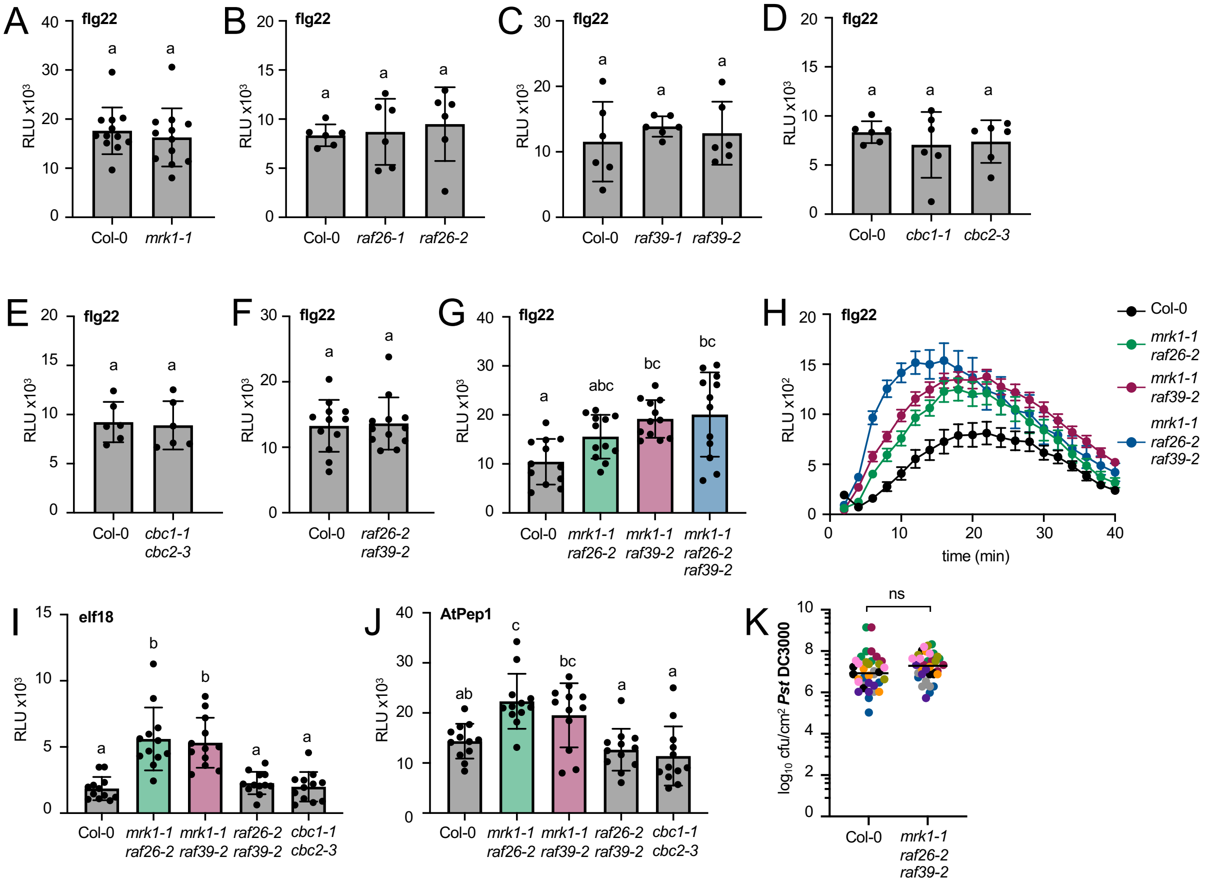

Figure S6. Immune-triggered ROS production in single and double C7-Raf mutants.

(A-H) Reative oxygen species (ROS) production measured in relative light units (RLU) after treatment with 100 nM flg22. Values represent means +/- standard deviation (n=6-12). Data presented in A, B, and D was collected by KRS; data in C was collected by MGD; data in E was collected by JM; data in F, G, and H was collected by BD. Data in G and H are from the same experiment, presented in G as total RLU and in H as a burst over 40 minutes (values in H are means +/- standard error (n=12). Lower-case letters indicate statistically significant groups determined by a one-way analysis of variance (ANOVA) followed by Tukey's post-hoc test (p<0.005). These assays were repeated several times over a 5-year period by KRS, MGD, BD and JM; representative experiments are shown. **(I-J)** ROS production measured in RLU after treatment with 100 nM elf18 **(I)** or 500 nM AtPep1 **(J)**. Values represent means +/- standard deviation (n=6-12). Lower-case letters indicate statistically significant groups determined by a one-way ANOVA followed by Tukey's post-hoc test (p<0.005). These assays were repeated three times with similar results over a 6-month period by MGD; representative experiments are shown. **(K)** Growth of *Pseudomonas syringae* pv. *tomato* (*Pst*) isolate DC3000 3 days after syringe-inoculation. Data from 9 independent biological replicates are plotted together, denoted by different coloured dots. Values are colony forming units (cfu) per leaf area (cm^2) from 4 samples per genotype (each sample contains 3 leaf discs from 3 different infected plants). The line represents the mean (n=36). A Student's unpaired t-test indicates no significant difference (p=0.1998). Data was collected by MGD over an 18-month period. Credits for genetic crosses and genotyping are provided in **Supporting Information Table S1**. All germplasm is *A. thaliana*.

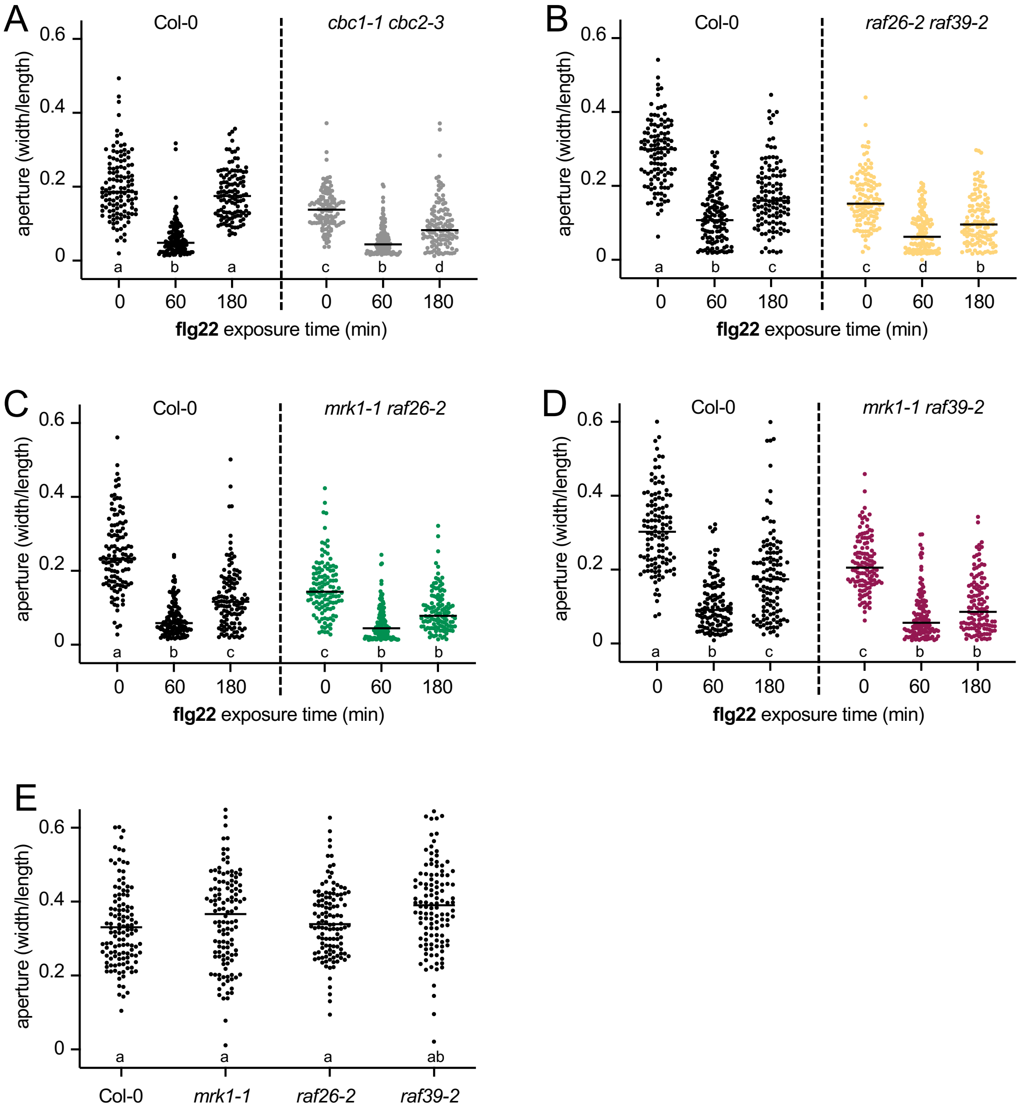

Figure S7. Stomatal aperture in single and double C7-Raf mutants.

(A-E) Stomatal apertures prior to (0 min) and following exposure to 1 µM flg22 (60, 180 min). Individual values are plotted and represent ratios of stomatal width:length. The straight line represents the mean (n=120). Lower case letters indicate statistically significant groups, determined by a one-way analysis of variance (ANOVA) followed by Tukey's post-hoc test (p<0.025). These experiments were completed at least 3 times each over a 12-month period with similar results by BD; representative data is shown. Credits for genetic crosses and genotyping are provided in **Supporting Information Table S1**. All germplasm is *A. thaliana*.

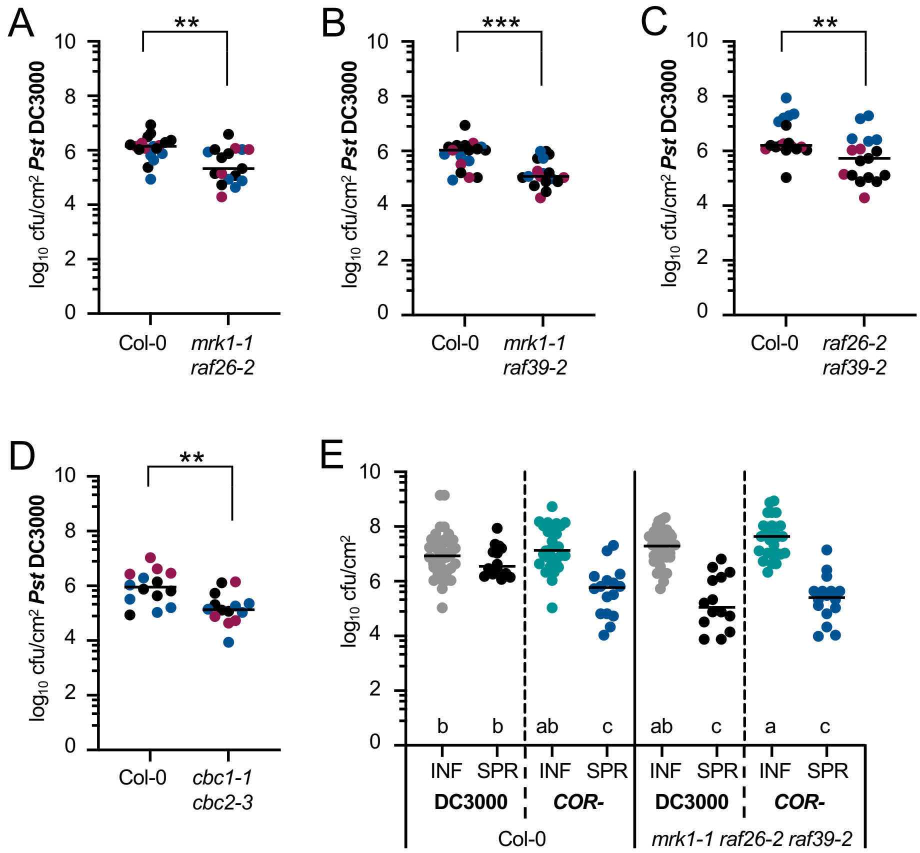

Figure S8. Infection assays with *Pst* DC3000 and *Pst* DC3000 *COR-*.

(A-D) Growth of *Pseudomonas syringae* pv. *tomato* (*Pst*) isolate DC3000 3 days after spray-inoculation in *mrk1-1 raf26-2* **(A)**, *mrk1-1 raf39-2* **(B)**, *raf26-2 raf39-2* **(C)**, and *cbc1-1 cbc2-3* **(D)** compared to Col-0. Data from 3-4 independent biological replicates are plotted together, denoted by black, gray, blue, and magenta dots. Values are colony forming units (cfu) per leaf area (cm^2) from 4-5 samples per genotype (each sample contains 3 leaf discs from 3 different infected plants). The line represents the mean (n=12-16). Asterisks indicate significantly different groups, determined by a Student's unpaired t-tests (** $p < 0.008$; *** $p = 0.0005$). Data was collected by AR over a 6-month period. **(E)** Growth of *Pst* DC3000 (DC3000; gray, black) and *Pst* DC3000 *COR-* (*COR-*; teal, blue) 3 days after syringe infiltration (INF) or spray inoculation (SPR). Values are colony forming units (cfu) per leaf area (cm^2) from 4-5 samples per genotype (each sample contains 3 leaf discs from 3 different infected plants). The line represents the mean (n=16-28). Data from 3-7 independent biological replicates are plotted together. Data for DC3000 SPR are the same as presented in **Figure 4F**; Data for DC3000 INF is the same as presented in **Supporting Information Figure S6K**. Data for *COR-* SPR and *COR-* INF were collected by MDG over a 6-month period. Statistically significant groups are indicated by lower-case letters as determined by a one-way analysis of variance (ANOVA) followed by Tukey's posthoc test ($p < 0.05$). All germplasm is *A. thaliana*.

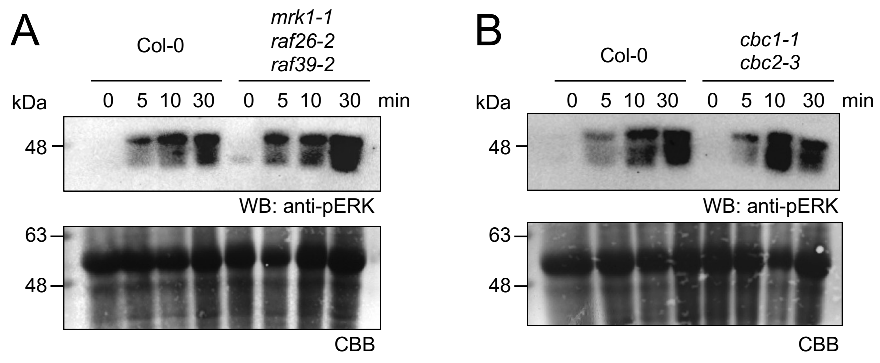

Figure S9. Flg22-triggered activation of MAPKs in C7 Raf-like mutants.

(A-B) Western blots (WB) indicating the activation of MAPKs before (0 min) and after exposure to 1 µM flg22 (5, 10, 30 min) in the indicated genotypes. The anti-pERK antibody recognizes the phosphorylated/activated forms of *A. thaliana* MPK6, MPK3, and MPK4/11. Coomassie Brilliant Blue (CBB) staining of the same membranes indicates loading. Experiments were completed at least 3 times over a 6-month period with similar results by MGD; representative data is shown. Credits for genetic crosses and genotyping are provided in **Supporting Information Table S1**. All germplasm is *A. thaliana*.

Supporting Information - Methods

Methods S1. Full details pertaining to the materials and methods used in this study.

Plant growth conditions

The experimental conditions used to grow and harvest samples from *cpk28-1/35S:CPK28-YFP,* Col-0/*35S:CPK5-YFP, nsl1-1/35S:NSL1-YFP* and Col-0/*35S:Lti6B-GFP* for the proteomics screen was previously described (Bender et al. 2017). Briefly, plants were grown on soil for 22 days under 10-h-light/14-h-dark cycle at 22˚C in controlled environmental chambers at the John Innes Centre (Norwich Research Park). All other plants were grown in the Queen's University Phytotron. For aseptic growth, Arabidopsis seeds were surface sterilized with 40% bleach, sown on petri plates containing 0.5x Murashige and Skoog (MS) media (Cedarlane) and 0.8% agar, and stratified for 3-5 days at 4˚C in the dark prior to exposure to light. For soil-grown plants, seeds were sown directly on potting soil (Sungro Sunshine Mix 1 or Fafard's Agro G6 w. Coco) and seedlings were transplanted into pots as individual plants in 3" pots or as 6 plants/pot in 8" pots two weeks after sowing. Plants were grown in controlled growth chambers (BioChambers) with a 10-h-light/14-h-dark cycle at 22˚C, with 30% relative humidity and a light intensity of 150 μM photonsm^2 s^{-1}, top-watered when needed (typically every other day), and fertilized biweekly with a solution of 1.5 g/L 20:20:20 N:P:K. *Nicotiana benthamiana* seeds were sown directly on potting soil as above, transplanted as individual seedlings per pot, and grown in a dedicated growth chamber (Conviron) under similar conditions, except with a 16-h-light/8-h-dark cycle and fertilized weekly. Mite bags containing *Amblyseius swirskii* (Koppert) were added to each tray of plants bi-weekly to prevent pest infestations.

RNA extractions and qRT-PCR

To assess if the insertion mutations resulted in lower gene expression, target genes were amplified using quantitative reverse transcription (qRT)-PCR. For this, leaf tissue was ground in liquid N_2 and total RNA was extracted using the Aurum Total RNA Mini Kit (BioRad) according to the manufacturer's instructions. Superscript III reverse transcriptase (Invitrogen) was used with oligo dT18 to generate cDNA according to the manufacturer's instructions. cDNA was diluted and target gene expression assessed by qRT-PCR using gene-specific primers and SsoAdvanced Universal SYBR Green Supermix (BioRad). Detailed information regarding all germplasm generated or used in this study, including primers used for genotyping and qRT-PCR, is available in **Supporting Information Table S1**.

Identification of CPK28-associated proteins

Proteins identified in immunoaffinity-enriched samples were measured with data dependent method on high resolution LC-MS systems, Orbitrap Fusion (Thermo Fisher Scientific). The acquired spectra were peak-picked and searched by Mascot search engine (Matrix Science Ltd.) to identify the peptide sequences from the search space defined by the background proteome. The peptides were combined into proteins based on the principle of parsimony by the search engine. Resulting proteins were further described by quantitative values based on the number of spectra that identified them. The individual runs were combined in the Scaffold program (Proteome Software Inc.), where the data were evaluated and filtered to contain less than 1% false positives (FDR) and the resulting matrix was exported as a spreadsheet. The matrix of proteins detected in different samples served as the input for an R script for further processing and visualization (**Supporting Information Notes S1**).

Molecular cloning

Information about all vectors used in this study, including previously published vectors, can be found in **Supporting Information Table S1.** The coding sequence of *MRK1* was amplified from DKLAT3G63260 (Popescu et al. 2007), while the genomic sequence of *MRK1* including the native promoter 1000 bp upstream of the translational start site was amplified from Col-0 genomic DNA using Q5 *Taq* Polymerase (NEB). Gateway-compatible pENTR-MRK1 np/ns (coding sequence, no promoter, no stop codon) and pENTR-MRK1 op/ns (genomic sequence, own promoter, no stop codon) clones were generated the using Gibson Assembly Master Mix (NEB) according to the manufacturer's instructions. Gateway-compatible pTwistENTR vectors containing the coding sequences for *RAF26* or *RAF39* were synthesized by Twist BioSciences. An additional guanine was added to the inserts to maintain the first coding frame for C-terminal fusions following recombination into destination vectors. Recombination into the binary pK7FWG2 destination vector (Karimi, Inzé, and Depicker 2002) for expression of *MRK1, RAF26*, and *RAF39* driven by the cauliflower mosaic virus (CaMV) *35S* promoter and C-terminally tagged with green fluorescent protein (GFP), or the pGWB4 destination vector (Nakagawa et al. 2007) for expression under the *pMRK1* promoter C-terminally tagged with GFP, was achieved using Gateway LR Clonase II (Invitrogen) according to the manufacturer's instructions.

Vectors suitable for split-luciferase complementation were generated either by traditional digestion-ligation cloning or Gateway LR reactions. For digestion-ligation cloning, engineered 5' and 3' endonuclease sites flanking the target genes facilitated ligation into pCAMBIA1300-nLuc for *35S*-driven expression of recombinant proteins C-terminally tagged with the N-terminal 416 amino acids of firefly luciferase, or into pCAMBIA1300-cLuc for *35S*-driven expression of recombinant proteins N-terminally tagged with the C-terminal 153 amino acids of firefly luciferase (Chen et al. 2008). The coding sequences of *FER, WAK1,* and

PP2A2 were amplified from DKLAT vectors (Popescu et al. 2007) or Col-0 cDNA with Q5 *Taq* Polymerase (NEB) and desalted using GenepHlow PCR Cleanup Kit (GeneAid) according to the manufacturer's instructions. *MRK1, RAF26, RAF39,* and other coding sequences were synthesized by Twist BioSciences and rehydrated in pure water. Fragments and vector backbones were digested with appropriate endonucleases (NEB), desalted using GenepHlow PCR Cleanup Kit (GeneAid) and ligated with T4 DNA ligase (NEB), each step according to manufacturer's directions. For Gateway-compatible clones, we used pGWB-nLuc or pGWB-cLuc vectors engineered from pCAMBIA1300-nLuc and pCAMBIA1300-cLuc (Yu et al. 2020). Entry vectors were either synthesized by Twist Biosciences or obtained from the ABRC, as outlined in **Supporting Information Table S1**. Recombination into destination vectors was achieved using Gateway LR Clonase II (Invitrogen) according to the manufacturer's instructions. Whenever entry and destination vectors had the same antibiotic resistance markers, the entry vector backbone was linearized by endonuclease digestion prior to the LR reaction. Vectors suitable for expression and purification of His_6- and/or glutathione S-transferase (GST)-tagged recombinant proteins in *Escherichia coli* were either cloned by Twist Biosciences into the pET28a+ backbone (EMD Biosciences) or cloned in-house into the pGex6p.1 backbone (GE Healthcare). When needed, mutations were incorporated directly at the synthesis stage.

Plasmids were transfected into *E. coli* Top10 cells, selected on petri plates with 1% agar and Luria-Bertani (LB) media (BioShop Canada) supplemented with appropriate antibiotics. Single colonies were used to inoculate liquid cultures and plasmids were extracted using the Presto Mini Plasmid Kit (GeneAid) according to manufacturer's instructions. Successful assemblies were confirmed either by Sanger sequencing (Centre for Applied Genomics, Toronto ON, Canada) or by whole-plasmid sequencing (Plasmidosaurus, Eugene OR, USA).

Agrobacterium-mediated transient expression in *N. benthamiana*

Binary vectors were transfected into *Agrobacterium tumefaciens* strain GV3101 cells and grown on LB plates containing appropriate antibiotics. A single colony was transferred to liquid LB media with appropriate antibiotics, and grown for 12-16 h at 28˚C. Cells were pelleted gently at 600 x *g*, resuspended in induction buffer (10 mM $MgCl_2$, 10 mM MES pH 6.3), incubated for 2-3 h at room temperature on an orbital shaker, and normalized to OD_{600}=0.2 using a microplate reader (SpectraMax Paradigm). Fully expanded upper leaves were selected from 4-week-old *N. benthamiana* plants for transformation. All constructs were co-transformed with viral suppressor P19 (Voinnet et al. 2003), and leaves were infiltrated on the abaxial side using a 1 mL needleless syringe. Tissue for confocal imaging or split-luciferase complementation was harvested three days after infiltration.

Split-luciferase complementation

A. tumefaciens carrying plasmids suitable for split-luciferase complementation assays (either pCAMBIA1300-n/cLuc or pGWB-n/cLuc; see **Supporting Information Table S1**) were used to transiently express proteins of interest in *N. benthamiana* as described above. Three days post infiltration, leaf disks (n=12) were collected using a 4 mm biopsy punch and placed in 100 µL of double-distilled water (ddH_2O) in a white 96 well plate. Once all samples were collected, the water was replaced with 50 µL 1 mM D-Luciferin (Gold Biotechnology), incubated in the dark for 15 minutes, and luminescence recorded in a plate reader with an integration time of 1 s/well (SpectraMax Paradigm).

Confocal microscopy

Leaf samples were collected with a 4 mm biopsy punch and mounted abaxial-side-up on a glass slide in a drop of water. Fluorescent proteins were excited with a 488 Argon laser and imaged using separate channels to detect emission of GFP (510-540 nm) or RFP (635-680 nm). For co-localization we used ER-mCherry, which was created by translationally fusing mCherry with an N-terminal secretion signal and a C-terminal HDEL sequence (Nelson, Cai, and Nebenführ 2007), and BRI1-mRFP (Saile et al. 2021). Images were taken using a Zeiss LSM710 confocal microscope in the Biology Department at Queen's University and processed using Zeiss Zen Software.

Immune assays

Immunogenic flg22, elf18, and AtPep1 peptides were synthesized by EZ Biotech (Indiana USA). Immune-induced ROS production was performed on 4- to 5-week-old soil-grown plants as previously described (Bredow et al. 2019). Immune-induced activation of MAPKs was performed on 2-week-old sterile seedlings as previously described (Monaghan et al. 2014). Bacterial infections were performed on 4- to 5-week-old soil-grown plants. For spray-inoculation, *Pseudomonas syringae* pv. *tomato* (*Pst*) DC3000 or *Pst* DC3000 *COR-* was cultured at 28˚C in LB media supplemented with rifampicin (*Pst* DC3000) or rifampicin and kanamycin (*Pst* DC3000 *COR-*). Cells were gently pelleted and diluted to OD_{600}=0.02 (10^7 cfu/mL for *Pst* DC3000) or OD_{600}=0.2 (10^8 cfu/mL for *Pst* DC3000 *COR-*) in 10 mM $MgCl_2$ and spray-inoculated onto 4-week-old plants until run-off. Right before spraying, 0.04% Silguard was added as a surfactant (Mireault, Paris, and Germain 2014). Three days after inoculation, leaf tissue was harvested using a 4-mm biopsy punch and homogenized in 10 mM $MgCl_2$. Four samples per genotype were collected by combining leaf discs from three different plants, serially diluted in 10 mM $MgCl_2$, and bacterial growth was determined by expressing the number of colony forming units (cfu) per leaf area. Syringe-inoculations were performed similarly, however *Pst* DC3000 or *Pst* DC3000 *COR-* were diluted to OD_{600}=0.0002 (10^5 cfu/mL) in 10 mM $MgCl_2$ with no surfactant prior to pressure-infiltration of half of the abaxial side of the leaf.

Stomatal apertures were measured across four middle-aged leaves of 4- to 5-week-old soil-grown

plants. The middle portion of each leaf was cut into three squares, avoiding the petiole, midrib, leaf base, tip, and margins. The leaf samples were placed in a buffer containing 50 mM KCl and 10 mM MES, pH ~6 in a sterile 12 well plate, covered with a transparent lid, and placed in a growth chamber for 3 h. Following this stomatal opening period, the leaf squares were separated into T0, T1, and T3 sampling groups and incubated with 1 µM flg22 for 60 min (T1) or 180 min (T3) to induce stomatal closing and re-opening. At the appropriate time point, the abaxial sides of the leaf squares were mounted to a piece of double-sided tape attached to a microscope slide and carefully scraped using a razor blade until only the epidermal layer was left. Multiple fields-of-view of the epidermal tissue layer, including stomata, were imaged using a Zeiss Axioplan microscope with a 40X oil immersion lens objective. Images and scales were converted to JPEG files using Zeiss Zen software. The width and length of 120 individual stomata per time point for each genotype were measured using Image J software (Schindelin et al. 2015) and converted to aperture values in R.

Protein purification

Proteins expressed in plant tissue: Relatively equal amounts of *N. benthamiana* tissue (twelve 4 mm leaf discs per sample) were flash-frozen and ground to a fine powder in liquid N_2, and proteins were extracted in standard Laemmli Buffer at 80˚C for 10 minutes prior to SDS-PAGE and immunoblotting. ***Proteins purified from E. coli:*** All proteins were expressed and purified from *E. coli* strain BL21 using the constructs outlined in **Supporting Information Table S1**. The cultures were grown at 37°C in LB containing appropriate antibiotics until the OD_{600} reached 0.7-0.8. Protein expression was induced by adding 0.5 mM or 1 mM of β-D-1-thio-galactopyranoside (IPTG) with shaking for 20 h at 28°C. Bacterial cells were harvested at 3,234 x *g* for 25 min at 4°C. The His_6-tagged proteins were resuspended in extraction buffer consisting of 50 mM Tris·HCl (pH 7.5), 100 mM NaCl, and 1 mM phenylmethylsulfonyl fluoride (PMSF). The GST-tagged proteins were resuspended in phosphate-buffered saline (PBS) (Thermo Fisher) containing 1 mM dithiothreitol (DTT) and 1 mM PMSF. Cells were lysed by passing the resuspended pellets three times through a French Press G-M® High Pressure Cell Disruption (Clifton, NJ, USA). Lysates were clarified by centrifugation at 15,400 × *g* for 40 min at 4°C. The supernatants were loaded into a conical tube containing either nickel-nitriloacetic acid (HisPur™ 25215, Thermo Fisher Scientific) or glutathione agarose beads (G4510, Sigma Aldrich) with shaking for 1-2 hours at 4°C. His_6 proteins were eluted from Ni-NTA beads by sequential washes with extraction buffer containing different imidazole concentrations (10 mM, 25 mM, 50 mM, 250 mM and 500 mM). Elution fractions were dialyzed in 2,000 volumes of 25 mM Tris·HCl (pH 7.5), 50 mM NaCl, and 1 mM DTT overnight at 4°C. GST proteins were eluted from glutathione agarose beads by washing with the elution buffer (50 mM Tris·HCl (pH 7.5), 10 mM reduced glutathione, 5 mM DTT). All proteins were concentrated using Amicon Ultra-15 centrifugal filter unit (10 or 30 KDa MWCO, MilliporeSigma). Protein concentrations were determined using Bradford reagent (23200, Thermo Fisher) and aliquots were flash frozen in liquid N2 and stored at –80°C until use.

SDS-PAGE and immunoblotting

Samples were loaded on a 10% SDS polyacrylamide mini-gel using a Bio-Rad PROTEAN III system and separated at 75 V for 30 min followed by 150 V for 1 h in 1x SDS running buffer (25 mM Tris-HCl pH 6.8, 190 mM glycine, 0.1% (w/v) SDS). For immunoblots, proteins were then transferred to an EtOH-activated polyvinylidene difluoride (PVDF) membrane at 100 V for 1.5 h at 4˚C in a wet transfer buffer (25 mM Tris-HCl pH 6.8, 190 mM glycine, 20% EtOH). Membranes were blocked in a 5% skim milk/TBST (20 mM Tris-HCl pH 6.8, 150 mM NaCl, 0.1% Tween-20) solution for 1 h at room temperature, and incubated in the appropriate primary antibody for 12-16 h at 4˚C. If secondary antibodies were required, the membrane was washed with TBST prior to secondary incubation. All membranes were washed twice in TBST and once in TBS (20 mM Tris-HCl pH 6.8, 150 mM NaCl) for 10 min prior to enhanced chemiluminescence (ECL) detection of horseradish peroxidase (HRP)-conjugated antibodies. Membranes were incubated with ECL Clarity Substrate (BioRad) and visualized on a ChemiDoc Touch Imaging System (BioRad). Antibodies and titers used: 1:5,000 mouse anti-GFP (Roche 1814460001); 1:3,000 rat anti-GFP (ChromoTek 3H9); 1:5,000 rabbit anti-His (Cell Signaling 2365); 1:5,000 mouse anti-GST (Sigma SAB4200237); 1:2,000 rabbit anti-p44/42 MAPK (Erk1/2) (Cell Signaling 9102S); rat 1:5,000 anti-HA-HRP (Roche 12013819001); 1:10,000 goat anti-rabbit-IgG (Sigma A0545); 1:10,000 rabbit anti-rat IgG-HRP (Sigma A5795); 1:10,000 goat anti-mouse-HRP (Sigma A0168). Depending on the experiment, gels or membranes were stained with Coomassie Brilliant Blue (CBB) R-250 (MP Biomedicals) or SimplyBlue SafeStain (Invitrogen; CBB G-250) to assess protein levels or verify loading.

Phosphoproteomics

SDS-PAGE protein gel slices were destained four times with 50% acetonitrile (ACN) in 100 mM triethylammonium bicarbonate (TEAB) for 10 min at 37°C. Gel pieces were washed with 100 mM TEAB for 10 min at 37°C, and then dehydrated by incubating in 100% ACN for 10 min at room temperature (RT). ACN was removed and gel pieces were then fully dried for 5 min at 37°C. Cysteine residues were reduced by adding 10 mM dithiothreitol solution in 100 mM TEAB for 45 min at 37°C and then alkylated with 55 mM iodoacetamide in 100 mM TEAB buffer in the dark for 1 h at RT. Gel pieces were then washed in 50 mM TEAB buffer for 10 min, followed by two incubations in 100% ACN at RT and then fully dried at 37°C for 5 min. The peptides were digested by the addition of 6 ng/µL trypsin (Promega Sequencing Grade - V5113) in 50 mM TEAB and allowed to digest for 16 h at 37°C with gentle shaking. In-solution tryptic peptides were retained in a separate tube, and in-gel digested peptides were further extracted by adding 1% formic acid, 2% acetonitrile in 100 mM TEAB and incubated for 1 h at 37°C. In-gel tryptic peptides were further extracted by a 1 h 37°C incubation using a 1:1 mixture of 1% formic acid in 50 mM TEAB and 100% acetonitrile

extraction buffer. The in-solution tryptic peptides were pooled with the aforementioned secondary extraction and dried down. Dried peptides were re-suspended in 3% (v/v) ACN / 0.1% (v/v) Trifluoroacetic acid and desalted using ZipTip C18 pipette tips (ZTC18S960; Millipore), as previously described (Uhrig et al. 2019). Desalted peptides were then dried and re-suspended in 3% (v/v) ACN / 0.1% (v/v) formic acid immediately prior to MS analysis. Data was acquired using a Orbitrap Fusion Lumos Tribrid system (Thermo Fisher Scientific). Peptides were eluted into the mass spectrometer using an nLC-1200 (Thermo Fisher Scientific) mounted with an ES903 column (Thermo Fisher Scientific). Peptides were eluted using a 45 min gradient of increasing buffer B (80% ACN, 0.1 % FA) 0-46% (40 min to buffer A (3% ACN, 0.1 % FA). All mass spectra were acquired in data dependent acquisition mode. MS1 data were acquired using a resolution of 120000 scan range; 375-2000; 40 RF lens. MS2 data were acquired using the ion trap in rapid scan rate mode, with maximum injection time set to dynamic. A 30% HCD collision energy was used for peptide fragmentation. Subsequently, all data was analyzed using MaxQuant 2.0.3.0(Cox and Mann 2008) using default parameters and the Araport 11 database(Cheng et al. 2017) with decoy mode set to revert. In brief, data search parameters included: trypsin cleavage permitting 2 missed cleavages, carbamidomethylation of cysteine residues (fixed modification), while methionine oxidation and phosphorylated serine/threonine/tyrosine were set as variable modifications. A PSM, peptide and protein FDR threshold of 0.01 was employed.

Supporting Information - References

Al-Momani, Shireen, Da Qi, Zhe Ren, and Andrew R. Jones. 2018. “Comparative Qualitative Phosphoproteomics Analysis Identifies Shared Phosphorylation Motifs and Associated Biological Processes in Evolutionary Divergent Plants.” *Journal of Proteomics* 181 (June):152–59.

Bender, Kyle W., R. Kevin Blackburn, Jacqueline Monaghan, Paul Derbyshire, Frank L. H. Menke, Cyril Zipfel, Michael B. Goshe, Raymond E. Zielinski, and Steven C. Huber. 2017. “Autophosphorylation-Based Calcium (Ca2+) Sensitivity Priming and Ca2+/Calmodulin Inhibition of Arabidopsis Thaliana Ca2+-Dependent Protein Kinase 28 (CPK28).” *The Journal of Biological Chemistry* 292 (10): 3988–4002.

Bhaskara, Govinal Badiger, Tuan-Nan Wen, Thao Thi Nguyen, and Paul E. Verslues. 2017. “Protein Phosphatase 2Cs and Microtubule-Associated Stress Protein 1 Control Microtubule Stability, Plant Growth, and Drought Response.” *The Plant Cell* 29 (1): 169–91.

Bredow, Melissa, Irina Sementchoukova, Kristen Siegel, and Jacqueline Monaghan. 2019. “Pattern-Triggered Oxidative Burst and Seedling Growth Inhibition Assays in Arabidopsis Thaliana.” *Journal of Visualized Experiments: JoVE*, no. 147 (May). https://doi.org/10.3791/59437.

Cheng, Chia-Yi, Vivek Krishnakumar, Agnes P. Chan, Françoise Thibaud-Nissen, Seth Schobel, and Christopher D. Town. 2017. “Araport11: A Complete Reannotation of the Arabidopsis Thaliana Reference Genome.” *The Plant Journal: For Cell and Molecular Biology* 89 (4):

789–804.
Chen, Huamin, Yan Zou, Yulei Shang, Huiqiong Lin, Yujing Wang, Run Cai, Xiaoyan Tang, and Jian-Min Zhou. 2008. "Firefly Luciferase Complementation Imaging Assay for Protein-Protein Interactions in Plants." *Plant Physiology* 146 (2): 368–76.
Cox, Jürgen, and Matthias Mann. 2008. "MaxQuant Enables High Peptide Identification Rates, Individualized P.p.b.-Range Mass Accuracies and Proteome-Wide Protein Quantification." *Nature Biotechnology* 26 (12): 1367–72.
Craigon, David J., Nick James, John Okyere, Janet Higgins, Joan Jotham, and Sean May. 2004. "NASCArrays: A Repository for Microarray Data Generated by NASC's Transcriptomics Service." *Nucleic Acids Research* 32 (Database issue): D575–77.
Hayashi, Maki, Hodaka Sugimoto, Hirotaka Takahashi, Motoaki Seki, Kazuo Shinozaki, Tatsuya Sawasaki, Toshinori Kinoshita, and Shin-Ichiro Inoue. 2020. "Raf-like Kinases CBC1 and CBC2 Negatively Regulate Stomatal Opening by Negatively Regulating Plasma Membrane H + -ATPase Phosphorylation in Arabidopsis." *Photochemical & Photobiological Sciences: Official Journal of the European Photochemistry Association and the European Society for Photobiology* 19 (1): 88–98.
Hiyama, Asami, Atsushi Takemiya, Shintaro Munemasa, Eiji Okuma, Naoyuki Sugiyama, Yasuomi Tada, Yoshiyuki Murata, and Ken-Ichiro Shimazaki. 2017. "Blue Light and CO2 Signals Converge to Regulate Light-Induced Stomatal Opening." *Nature Communications* 8 (1): 1284.
Hoehenwarter, Wolfgang, Martin Thomas, Ella Nukarinen, Volker Egelhofer, Horst Röhrig, Wolfram Weckwerth, Uwe Conrath, and Gerold J. M. Beckers. 2013. "Identification of Novel in Vivo MAP Kinase Substrates in Arabidopsis Thaliana through Use of Tandem Metal Oxide Affinity Chromatography." *Molecular & Cellular Proteomics: MCP* 12 (2): 369–80.
Karimi, Mansour, Dirk Inzé, and Ann Depicker. 2002. "GATEWAY Vectors for Agrobacterium-Mediated Plant Transformation." *Trends in Plant Science* 7 (5): 193–95.
Marondedze, Claudius, Arnoud J. Groen, Ludivine Thomas, Kathryn S. Lilley, and Chris Gehring. 2016. "A Quantitative Phosphoproteome Analysis of cGMP-Dependent Cellular Responses in Arabidopsis Thaliana." *Molecular Plant* 9 (4): 621–23.
Mireault, Caroline, Louise-Emmanuelle Paris, and Hugo Germain. 2014. "Enhancement of the Arabidopsis Floral Dip Method with XIAMETER OFX-0309 as Alternative to Silwet L-77 Surfactant." *Botany* 92 (7): 523–25.
Monaghan, Jacqueline, Susanne Matschi, Oluwaseyi Shorinola, Hanna Rovenich, Alexandra Matei, Cécile Segonzac, Frederikke Gro Malinovsky, et al. 2014. "The Calcium-Dependent Protein Kinase CPK28 Buffers Plant Immunity and Regulates BIK1 Turnover." *Cell Host & Microbe* 16 (5): 605–15.
Nakagawa, Tsuyoshi, Takayuki Kurose, Takeshi Hino, Katsunori Tanaka, Makoto Kawamukai, Yasuo Niwa, Kiminori Toyooka, Ken Matsuoka, Tetsuro Jinbo, and Tetsuya Kimura. 2007. "Development of Series of Gateway Binary Vectors, pGWBs, for Realizing Efficient Construction of Fusion Genes for Plant Transformation." *Journal of Bioscience and Bioengineering* 104 (1): 34–41.
Nelson, Brook K., Xue Cai, and Andreas Nebenführ. 2007. "A Multicolored Set of in Vivo Organelle Markers for Co-Localization Studies in Arabidopsis and Other Plants." *The Plant Journal: For Cell and Molecular Biology* 51 (6): 1126–36.
Nukarinen, Ella, Thomas Nägele, Lorenzo Pedrotti, Bernhard Wurzinger, Andrea Mair, Ramona Landgraf, Frederik Börnke, et al. 2016. "Quantitative Phosphoproteomics Reveals the Role of the AMPK Plant Ortholog SnRK1 as a Metabolic Master Regulator under Energy Deprivation." *Scientific Reports* 6 (August):31697.
Popescu, Sorina C., George V. Popescu, Shawn Bachan, Zimei Zhang, Montrell Seay, Mark

Gerstein, Michael Snyder, and S. P. Dinesh-Kumar. 2007. "Differential Binding of Calmodulin-Related Proteins to Their Targets Revealed through High-Density *Arabidopsis* Protein Microarrays." *Proceedings of the National Academy of Sciences of the United States of America* 104 (11): 4730–35.

Roitinger, Elisabeth, Manuel Hofer, Thomas Köcher, Peter Pichler, Maria Novatchkova, Jianhua Yang, Peter Schlögelhofer, and Karl Mechtler. 2015. "Quantitative Phosphoproteomics of the Ataxia Telangiectasia-Mutated (ATM) and Ataxia Telangiectasia-Mutated and rad3-Related (ATR) Dependent DNA Damage Response in Arabidopsis Thaliana." *Molecular & Cellular Proteomics: MCP* 14 (3): 556–71.

Saile, Svenja C., Frank M. Ackermann, Sruthi Sunil, Jutta Keicher, Adam Bayless, Vera Bonardi, Li Wan, et al. 2021. "Arabidopsis ADR1 Helper NLR Immune Receptors Localize and Function at the Plasma Membrane in a Phospholipid Dependent Manner." *The New Phytologist* 232 (6): 2440–56.

Schindelin, Johannes, Curtis T. Rueden, Mark C. Hiner, and Kevin W. Eliceiri. 2015. "The ImageJ Ecosystem: An Open Platform for Biomedical Image Analysis." *Molecular Reproduction and Development* 82 (7-8): 518–29.

Song, Gaoyuan, Libuse Brachova, Basil J. Nikolau, Alan M. Jones, and Justin W. Walley. 2018. "Heterotrimeric G-Protein-Dependent Proteome and Phosphoproteome in Unstimulated Arabidopsis Roots." *Proteomics* 18 (24): e1800323.

Takahashi, Yohei, Krystal C. Bosmans, Po-Kai Hsu, Karnelia Paul, Christian Seitz, Chung-Yueh Yeh, Yuh-Shuh Wang, et al. 2022. "Stomatal CO_2/bicarbonate Sensor Consists of Two Interacting Protein Kinases, Raf-like HT1 and Non-Kinase-Activity Requiring MPK12/MPK4." *Science Advances* 8 (49): eabq6161.

Uhrig, R. Glen, Pascal Schläpfer, Bernd Roschitzki, Matthias Hirsch-Hoffmann, and Wilhelm Gruissem. 2019. "Diurnal Changes in Concerted Plant Protein Phosphorylation and Acetylation in Arabidopsis Organs and Seedlings." *The Plant Journal: For Cell and Molecular Biology* 99 (1): 176–94.

Voinnet, Olivier, Susana Rivas, Pere Mestre, and David Baulcombe. 2003. "An Enhanced Transient Expression System in Plants Based on Suppression of Gene Silencing by the p19 Protein of Tomato Bushy Stunt Virus." *The Plant Journal: For Cell and Molecular Biology* 33 (5): 949–56.

Waese, Jamie, Jim Fan, Asher Pasha, Hans Yu, Geoffrey Fucile, Ruian Shi, Matthew Cumming, et al. 2017. "ePlant: Visualizing and Exploring Multiple Levels of Data for Hypothesis Generation in Plant Biology." *The Plant Cell* 29 (8): 1806–21.

Wang, Pengcheng, Liang Xue, Giorgia Batelli, Shinyoung Lee, Yueh-Ju Hou, Michael J. Van Oosten, Huiming Zhang, W. Andy Tao, and Jian-Kang Zhu. 2013. "Quantitative Phosphoproteomics Identifies SnRK2 Protein Kinase Substrates and Reveals the Effectors of Abscisic Acid Action." *Proceedings of the National Academy of Sciences of the United States of America* 110 (27): 11205–10.

Wang, Xu, Yangyang Bian, Kai Cheng, Li-Fei Gu, Mingliang Ye, Hanfa Zou, Samuel Sai-Ming Sun, and Jun-Xian He. 2013. "A Large-Scale Protein Phosphorylation Analysis Reveals Novel Phosphorylation Motifs and Phosphoregulatory Networks in Arabidopsis." *Journal of Proteomics* 78 (January):486–98.

Wu, Xu Na, Clara Sanchez Rodriguez, Heidi Pertl-Obermeyer, Gerhard Obermeyer, and Waltraud X. Schulze. 2013. "Sucrose-Induced Receptor Kinase SIRK1 Regulates a Plasma Membrane Aquaporin in Arabidopsis." *Molecular & Cellular Proteomics: MCP* 12 (10): 2856–73.

Yang, Yingzhen, Alex Costa, Nathalie Leonhardt, Robert S. Siegel, and Julian I. Schroeder. 2008. "Isolation of a Strong Arabidopsis Guard Cell Promoter and Its Potential as a

Research Tool." *Plant Methods* 4 (February):6.

Yu, Gang, Liu Xian, Hao Xue, Wenjia Yu, Jose S. Rufian, Yuying Sang, Rafael J. L. Morcillo, Yaru Wang, and Alberto P. Macho. 2020. "A Bacterial Effector Protein Prevents MAPK-Mediated Phosphorylation of SGT1 to Suppress Plant Immunity." *PLoS Pathogens* 16 (9): e1008933.
