# Supplementary material for: Subfamily C7 Raf‐like kinases MRK1, RAF26, and RAF39 regulate immune homeostasis and stomatal opening in Arabidopsis thaliana
Source: New Phytol. 2024 Oct 24;244(6):2278–94. doi: 10.1111/nph.20198 (PMC11579443; doi:10.1111/nph.20198)
Supplement: Supplementary file 2 — Notes S1 Analysis of spectral counts of CPK28‐GFP enriched proteins. [file NPH-244-2278-s002.pdf]

Notes S1 Analysis of spectral counts of enriched proteins

Immunoaffinity enrichment (co-IP) assay to study protein-protein interaction

For: Jacqueline Monaghan

By: Jan Sklenar

04 June, 2024, 17:09

- 1 Introduction
  - 1.1 Protein-protein interaction assay
- 2 Main script (data analysis)
  - 2.1 Data description
  - 2.2 Initial operations
    - 2.2.1 Read in the data
    - 2.2.2 Data cleanup
    - 2.2.3 Remove contaminants and decoys
    - 2.2.4 Define groups and replicates
    - 2.2.5 Show all the groups
    - 2.2.6 Show the selected groups
  - 2.3 Global visualization
    - 2.3.1 Calculate totals of spectral counts in sample groups
    - 2.3.2 Correlation matrix and heatmap
    - 2.3.3 Upsetr plot for groups: m28_22, m5_22, mPMG_22
    - 2.3.4 Visualizaiton of missing values
  - 2.4 Main calculations
    - 2.4.1 Pairwise comparisons
    - 2.4.2 Set thresholds to filter the results
    - 2.4.3 Main Loop
    - 2.4.4 Imputation
    - 2.4.5 Fold changes
  - 2.5 Evaluation of the results and plots
    - 2.5.1 Plotting the pairs
  - 2.6 Proteins of interest
    - 2.6.1 Working with the results
    - 2.6.2 The selected proteins, imputed values
    - 2.6.3 The selected proteins, original values
  - 2.7 Comparison of fold changes of the pairs
  - 2.8 End

1 Introduction

1.1 Protein-protein interaction assay

We study protein-protein interactions using immunoaffinity enrichment experiments, a.k.a. coimmunoprecipitation (coIP). The tagged proteins specifically enrich on an affinity media and the proteins co-purified with them form a list candidates that may form a complex with the tagged protein of interest. To increase specificity of the enrichment we use a negative controls, i.e. wild type, mutant or the tag only that in theory should only capture random or non specifically bound proteins.

2 Main script (data analysis)

2.1 Data description

Proteins identified in immunoaffinity enrichment (pull-down) were measured with data dependent method on high resolution LC-MS systems, Orbitrap Fusion. The acquired spectra were peak-picked and searched by Mascot search engine (Matrix Science Ltd.) to identify the peptide sequences from the search space defined by the background proteome. The peptides were combined into proteins based on the principle of parsimony by the search engine. Resulting proteins can be further described by quantitative values based on the number of spectra that identify them. The individual runs were combined in the Scaffold program (Proteome Software Inc.), where the data were evaluated and filtered to contain less than 1% false positives (FDR) and resulted matrix exported as a spreadsheet.

The matrix of proteins detected in different samples serves as the input for an R script for further processing and visualization.

2.2 Initial operations

2.2.1 Read in the data

Read the ‘csv’ file.

2.2.2 Data cleanup

Find where the data starts and ends in the input peptide search results, remove empty rows, corrections.

2.2.3 Remove contaminants and decoys

Here we remove hits of the typical proteomics artefacts such as keratin, trypsin, and decoy search hits.

2.2.4 Define groups and replicates

The raw file names need to be replaced with a comprehensible names, then the samples need to be grouped.

2.2.5 Show all the groups

```
## groups

print(knitr::kable(as.data.frame(mygroups),format = "markdown"))
```

```
##
##
## |                 |mygroups |
## |:----------------|:--------|
## |CPK28_mock_LD22  |m28_22   |
## |CPK5_mock_LD22   |m5_22    |
## |NSL1_mock_LD22   |mNSL_22  |
## |PM-GFP_mock_LD22 |mPMG_22  |
```

```
## raw file names
print(knitr::kable(mynames,format = "markdown"))
```

```
##
##
## |rawname          |mygroup | id|
## |:----------------|:-------|--:|
## |LD22_mock_R1_A   |m28_22  |  8|
## |LD22_mock_R2_A   |m28_22  |  9|
## |LD22_mock_R3_A   |m28_22  | 10|
## |CPK5_mock_R1_G   |m5_22   | 32|
## |CPK5_mock_R2_G   |m5_22   | 33|
## |CPK5_mock_R3_G   |m5_22   | 34|
## |NSL1_LD22_R3_K   |mNSL_22 | 41|
## |LD22_R1_K        |mNSL_22 | 43|
## |LD22_R2_K        |mNSL_22 | 44|
## |PM.GFP_LD22_R3_M |mPMG_22 | 48|
## |LD22_R2_M        |mPMG_22 | 51|
```

2.2.6 Show the selected groups

2.3 Global visualization

2.3.1 Calculate totals of spectral counts in sample groups

Total spectral counts of replicates within the sample groups should not change profoundly and serve as a basic quality control.

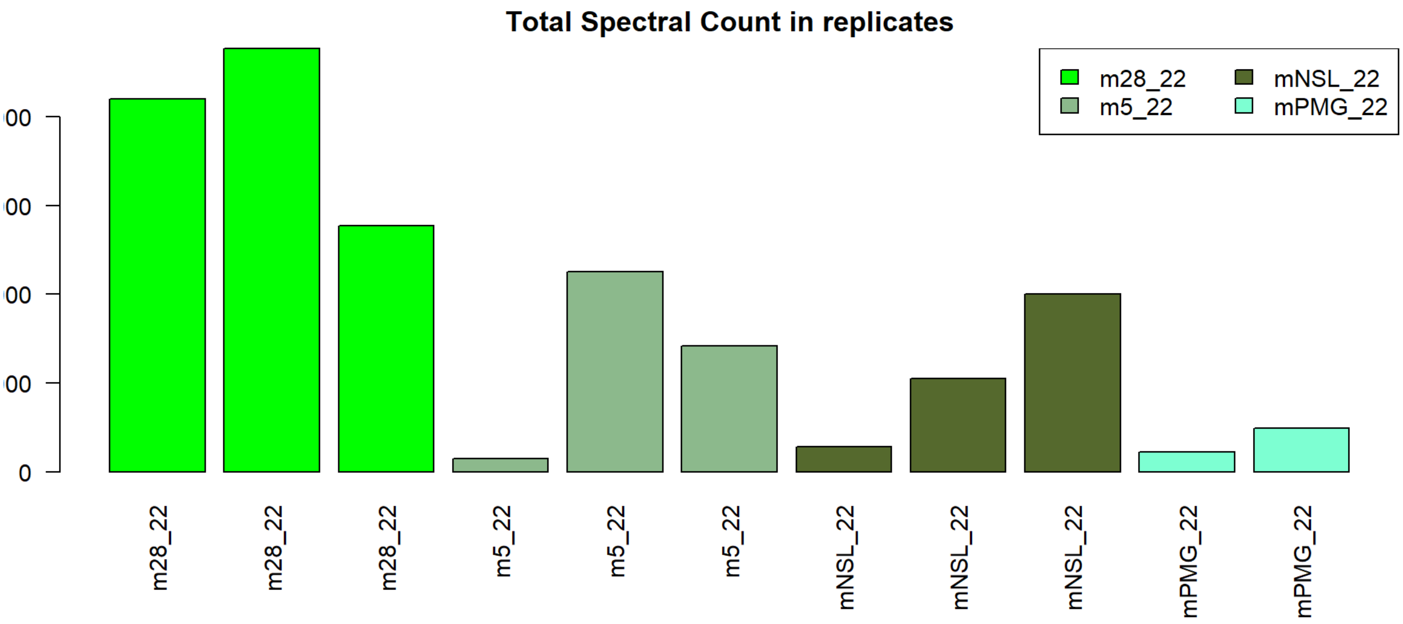

2.3.2 Correlation matrix and heatmap

```
##
## Attaching package: 'Hmisc'
```

```
## The following objects are masked from 'package:dplyr':
##
##     src, summarize
```

```
## The following objects are masked from 'package:base':
##
##     format.pval, units
```

```
## corrplot 0.92 loaded
```

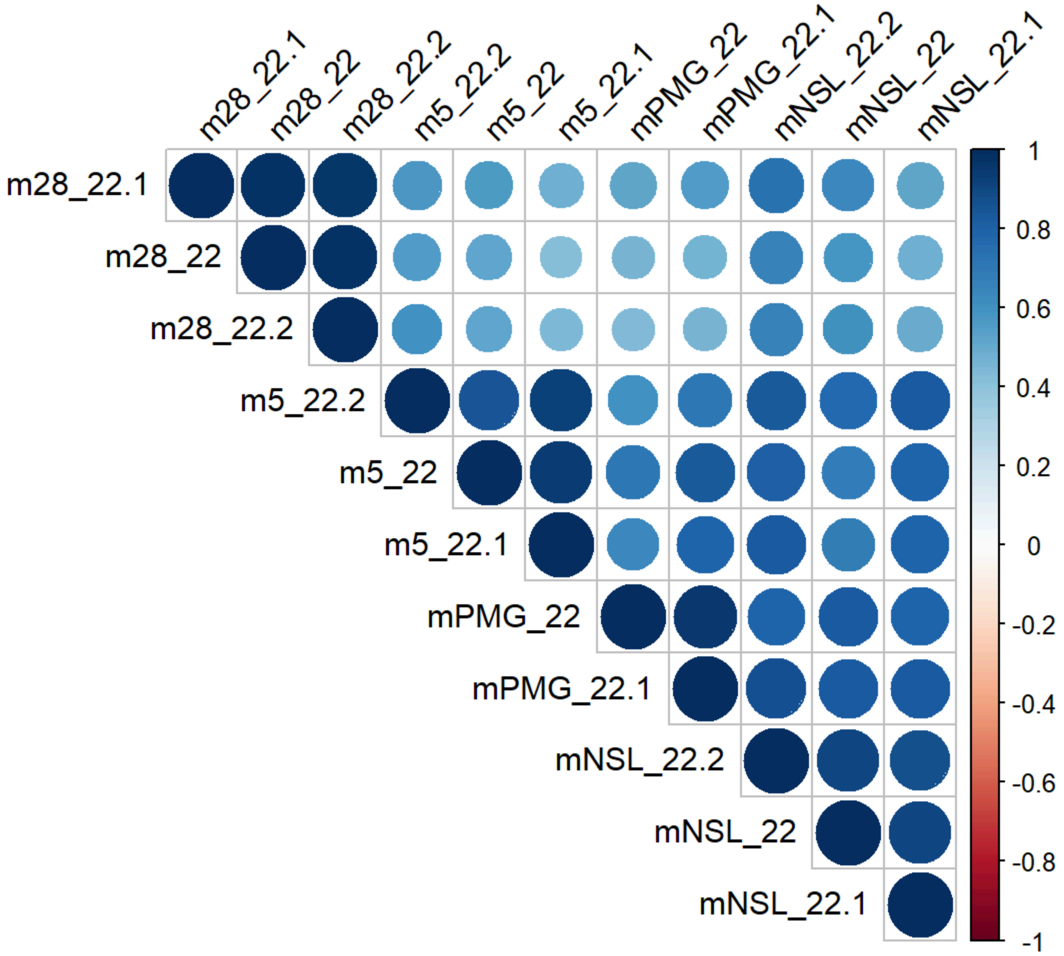
m28_22.1
m28_22
m28_22.2
m5_22.2
m5_22
m5_22.1
mPMG_22
mPMG_22.1
mNSL_22.2
mNSL_22
mNSL_22.1
1
0.8
0.6
0.4
0.2
0
-0.2
-0.4
-0.6
-0.8
-1

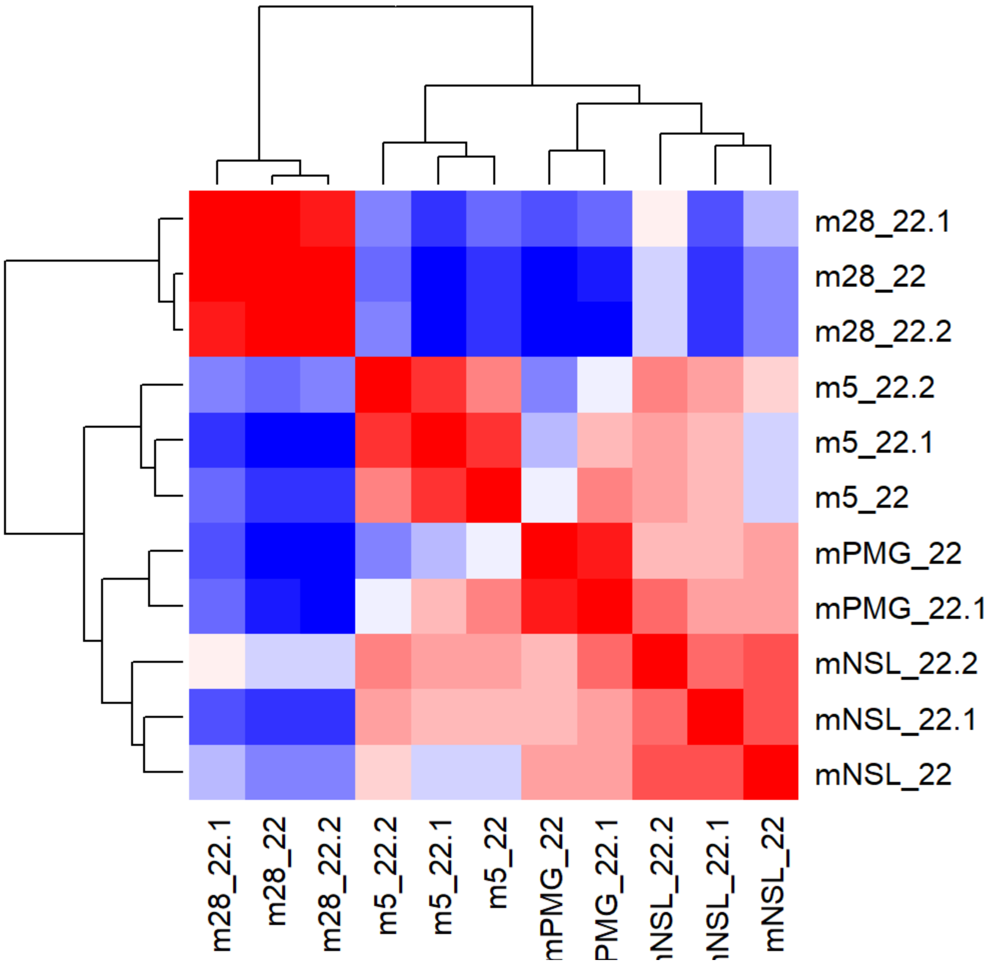
m28_22.1
m28_22
m28_22.2
m5_22.2
m5_22.1
m5_22
mPMG_22
mPMG_22.1
mNSL_22.2
mNSL_22.1
mNSL_22

2.3.3 Upsetr plot for groups: m28_22, m5_22, mPMG_22

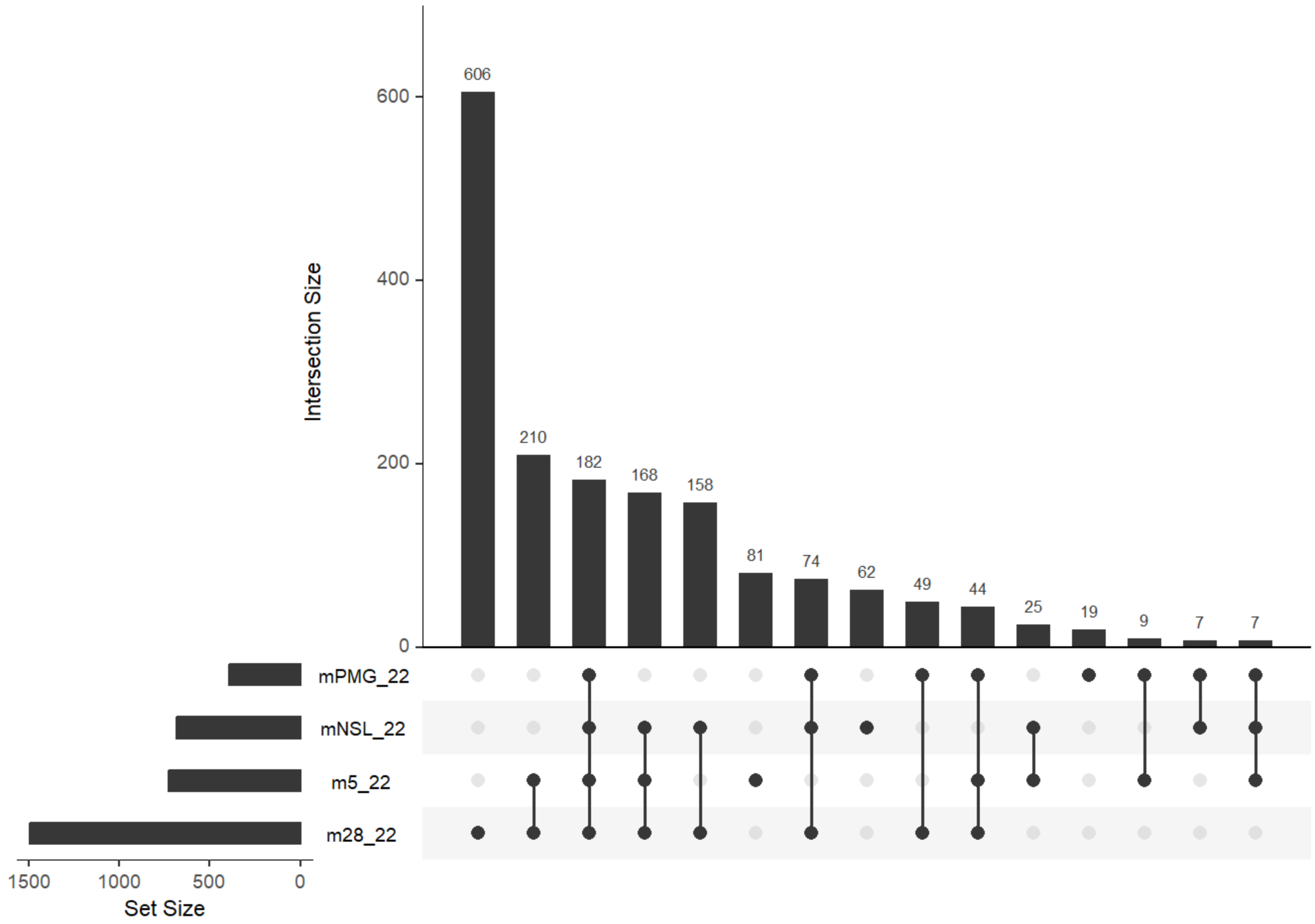

2.3.4 Visualizaiton of missing values

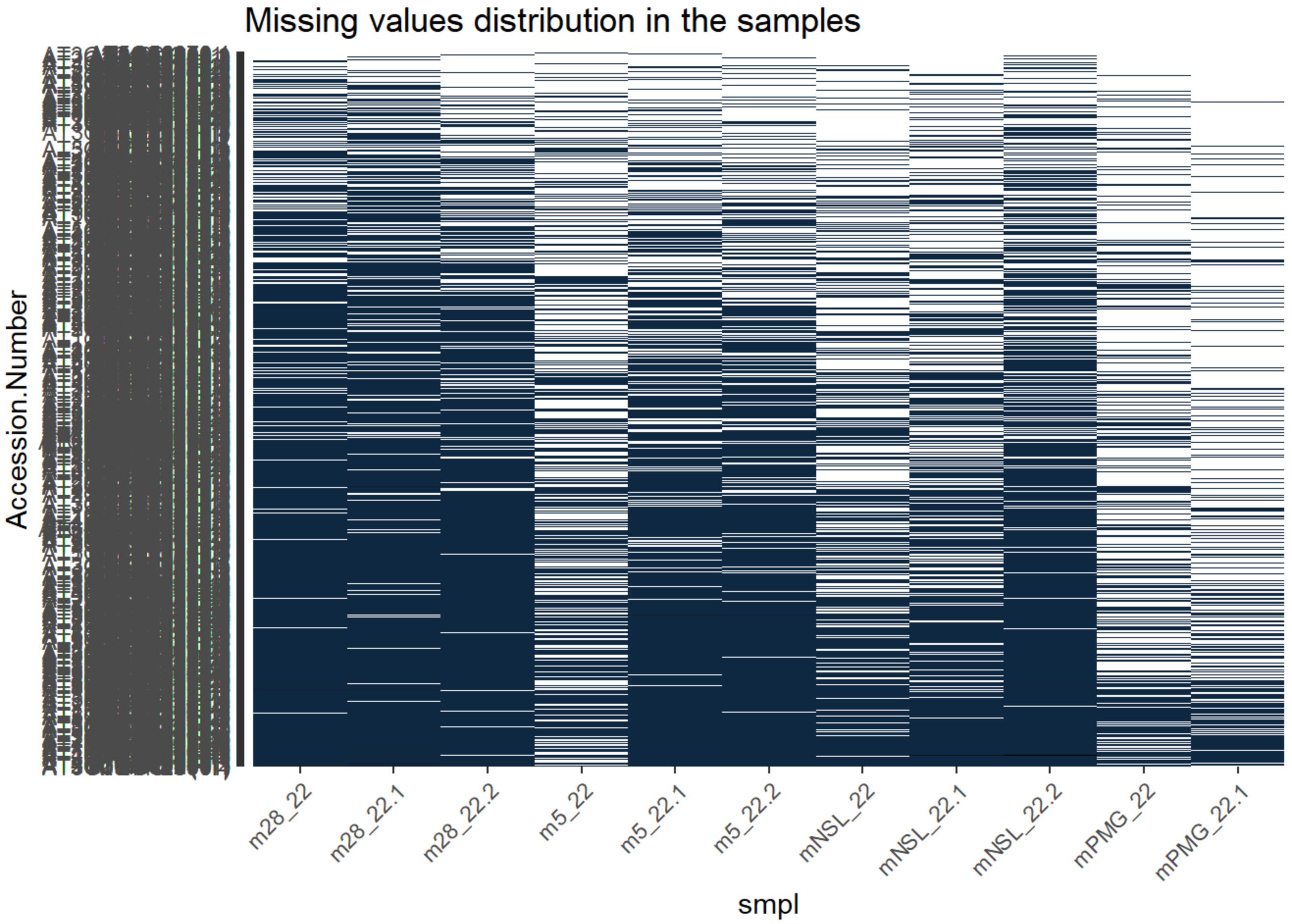


```
## This is quite typical, the most frequent value is a missing value. For this reason re
producibility and fold change filters will be used as tresholds for a conservative resul
t. We are going to calculate the thresholds below.
```

2.4 Main calculations

2.4.1 Pairwise comparisons

We reduce the data analysis to a series of binary comparisons by selecting “treatments” and “controls”. This simplifies complex experimental designs and helps to determine how the individual samples compare to controls. A disadvantage is that we may get several plots and tables that has to be compared and evaluated.

```
## The following groups of samples will be compared in
selected_pairs
```

```
## [[1]]
## [[1]]$treat
## [1] "m28_22"
##
## [[1]]$ctrl
## [1] "mNSL_22" "mPMG_22"
##
##
## [[2]]
## [[2]]$treat
## [1] "m5_22"
##
## [[2]]$ctrl
## [1] "mNSL_22" "mPMG_22"
##
##
## [[3]]
## [[3]]$treat
## [1] "m28_22"
##
## [[3]]$ctrl
## [1] "m5_22"
```

```
## The actual columns in the data
selected_pairs_cols
```

```
## [[1]]
## [[1]]$treat
## [1]  8  9 10
##
## [[1]]$ctrl
## [1] 41 43 44 48 51
##
##
## [[2]]
## [[2]]$treat
## [1] 32 33 34
##
## [[2]]$ctrl
## [1] 41 43 44 48 51
##
##
## [[3]]
## [[3]]$treat
## [1]  8  9 10
##
## [[3]]$ctrl
## [1] 32 33 34
```

```
## Meaningful group names for replicates
#names(ename[ename %in% unlist(lapply(selected_pairs, function(x) unique(x)))])

## How many replicates?
selected_pairs_lengths <- lapply(selected_pairs_cols, function(x) sapply(x, function(y)
length(y)))
selected_pairs_lengths
```

```
## [[1]]
## treat  ctrl
##     3     5
##
## [[2]]
## treat  ctrl
##     3     5
##
## [[3]]
## treat  ctrl
##     3     3
```

2.4.2 Set thresholds to filter the results

The filters help us to find the specific and reproducible results.

1. Fold changes
2. Percentage non missing values in sample replicates

```
############################################################
## PROCESSING PARAMETERS, FILTERS, VARIABLES
## Select
## Imputation options:
# imp = 'med'
# imp = 'mea'
imp <-  'fix'
impval <- 1 # when and for fixed value imputation only

## Define filters
## Fold change (log2) filter fc - we keep 'larger than' AND 'smaller than' fc,
#  a specified value, with exception of the hits identified uniquely. The latter we keep
as well.
#  The unique hit has a missing value in the other sample type, e.g. in 'ctrl'.
#  Fold change cannot be zero, as we use both pos/neg values of fc in the filter
fc <- 2

## Reproducibility filter (%) - we keep only hits found in at least
## certain percentage of replicates (of the same sample type)
perc  <- 50
##############################################################
```

2.4.3 Main Loop

1. Removal of typical contaminants, such as keratin, trypsin, etc.
2. Imputation of missing values (MVs) to allow the fold change calculation
3. Calculate mean spectral count for the treatment and the control
4. Calculate ratio log2(treatment/control)
5. Set threshold for the ratios that signify the specific protein-protein interaction
6. Set reproducibility Filter - minimum percentage of replicates the proteins must be found therein
7. Visualize the results in a bar-plot and a table

2.4.4 Imputation

Data imputation in proteomics is an active research area. While several approaches were suggested, there has not been a clear consensus in the community of how to deal with the missing data in various experiments and data formats. Here our strategy attempts for the imputation to be as optimistic as possible to provide a list of protein-protein interaction candidates. We cannot make any assumptions on the number of proteins that will be enriched and the abundance of the specific proteins. For this reason normalization is not used, and we simply assume the same amount of input tissue was used in every experiment. We can expect a substantial number of proteins to bind non specifically to the immunoaffinity beads. After the enrichment, we are dealing with a specific sub-proteome. These facts are contributing factors to many missing values that are often observed in the data. We impute them in the following stages.

- Zero values in Scaffold software mean a lower probability hit, we convert it to 1.
- MVs are then imputed within sample groups in pairs being compared.
- A group unique hits (a hit with only missing values in the other group being compared) are converted to 1.
- All the other MVs within sample groups are imputed as a mean or meadia or fixed value. When the meanis used there is noeffect on fold change ratio magnitude. Changing the parameter ‘imp’ and ‘impval’ in the scrip changes the imputed value. See above.

2.4.5 Fold changes

After the MV’s imputation we can calculate mean spectral counts within the selected sample groups, treatment and control. Then we take log2 ratio treatment / control. We mark proteins that were identified uniquely in the groups as they might be the best result.

2.5 Evaluation of the results and plots

The plots below show the calculated log2 fold changes between the treatment and the control.

Three different situations are color coded:

- Red: Unique in Treatment [t]
- Blue: Unique in Control [c]
- Green: Ratio [t/c]

In the immunoaffinity enrichment (IAE) experiments, the proteins of interest can be identified exclusively in the samples with the tagged protein [t] (red) or in controls [c] (blue). While the former is the sought result, the latter proteins are irrelevant to studied tagged protein, identified due to their non specific binding to the affinity media.

The proteins that occur both in the control and in the treatment [t/c] (green) could still be binding the tagged protein with limited specificity if the ratio shows an extreme (positive) value. Proteins found in the same/similar ratio are randomly sticking to the IAE media.

It is our evaluation that makes identified proteins a possible protein-protein interaction candidates with given tagged protein.

A substantial problem encountered in IAEs form frequent missing values (MVs) in sample replicates. As explained above, to calculate the fold change ratio, the MVs have been replaced with imputed values.

In the situation when there was nothing found in the control and we have to ask: Is the fact we do not detect anything in the control due to the protein-protein complex of the tagged protein with a co-purified protein or was specifically enriched? Or was it found by a chance? Answer is not straightforward, however more spectra we identify specifically for a protein increases the chance the protein is involved in a complex with our tagged protein. Another criterion for considering a protein to be an interaction candidate is reproducibility of its detection in biological sample replicates.

2.5.1 Plotting the pairs

Filtered with details in a table

===================== [1] "Pair No.: 1" [1] "CPK28 (m22) /// NSL1, PM-GFP (m22)"
=====================

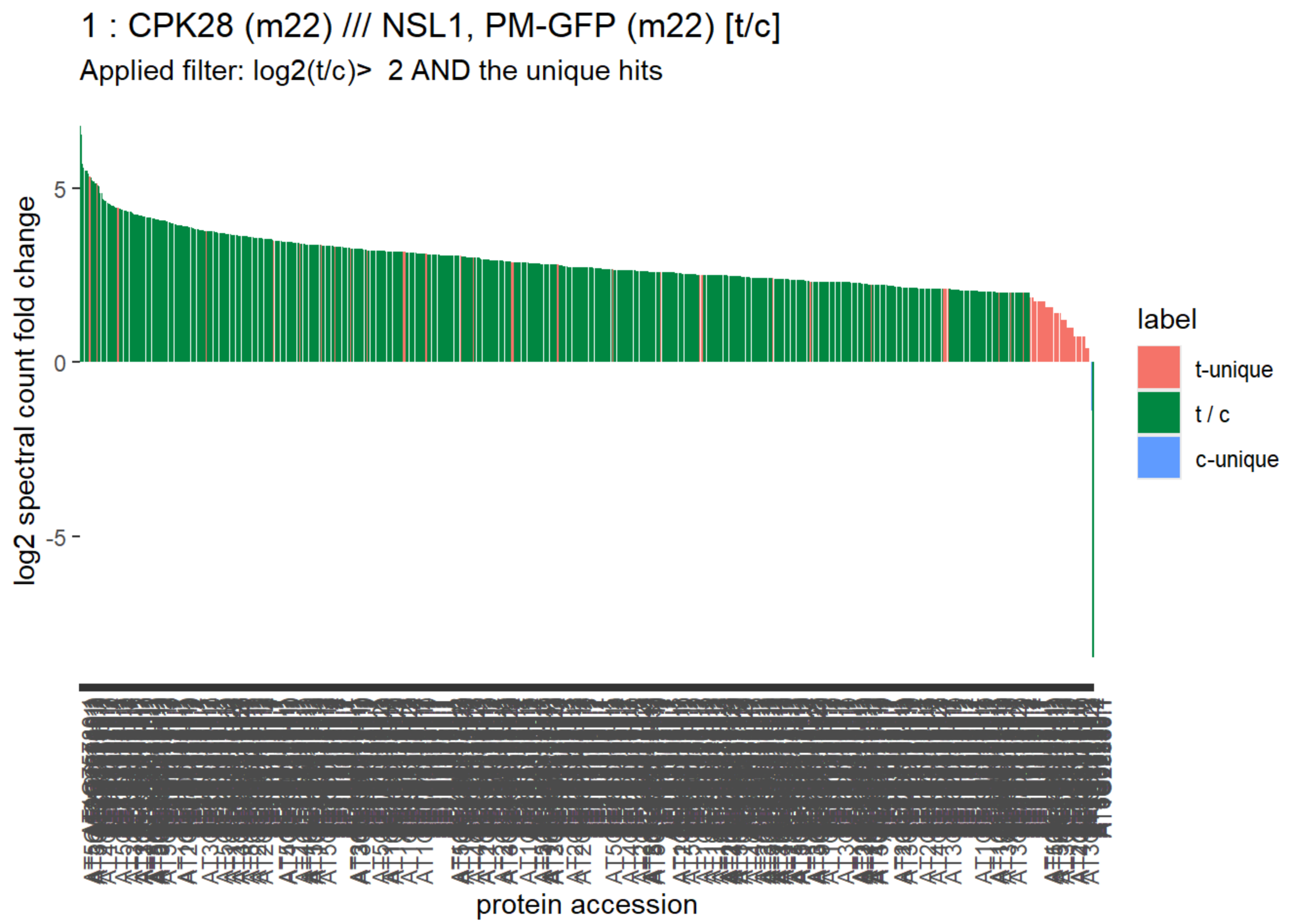

	Accession.Number	log2tc	label	Description
1527	AT1G75780.1	6.81	t / c	|TUB1 | tubulin beta-1 chain | chr1:28451378-

	Accession.Number	log2tc	label	Description
75	AT5G13490.1 (+1)	6.56	t / c	|AAC2 | ADP/ATP carrier 2 | chr5:4336034-4337
984	AT3G09840.1	5.71	t / c	|CDC48, ATCDC48, CDC48A | cell division cycle
27	AT2G17890.1	5.58	t / c	|CPK16 | calcium-dependent protein kinase 16
99	AT2G18960.1	5.51	t / c	|AHA1, PMA, OST2, HA1 | H(+)-ATPase 1 | chr2:
1493	AT3G08510.1 (+1)	5.51	t / c	|ATPLC2, PLC2 | phospholipase C 2 | chr3:2582
1528	AT1G72730.1	5.51	t / c	| | DEA(D/H)-box RNA helicase family protein
1	AT5G66210.1 (+1)	5.42	t / c	|CPK28 | calcium-dependent protein kinase 28
1541	AT2G20140.1	5.33	t-unique	| | AAA-type ATPase family protein | chr2:869
313	AT4G29130.1	5.32	t / c	|ATHXK1, GIN2, HXK1 | hexokinase 1 | chr4:143
108	AT4G30190.1	5.22	t / c	|AHA2, PMA2, HA2 | H(+)-ATPase 2 | chr4:14770
458	AT4G22690.1	5.21	t / c	|CYP706A1 | cytochrome P450, family 706, subf
536	AT1G01790.1	5.20	t / c	|KEA1, ATKEA1 | K+ efflux antiporter 1 | chr1
658	AT4G33090.1	5.15	t / c	|APM1, ATAPM1 | aminopeptidase M1 | chr4:1596
1476	AT4G28520.1	5.14	t-unique	|CRU3, CRC | cruciferin 3 | chr4:14087596-140
500	AT4G31480.1 (+1)	5.09	t / c	| | Coatomer, beta subunit | chr4:15264145-15
1530	AT4G36070.2	5.05	t / c	|CPK18 | calcium-dependent protein kinase 18
142	AT5G62670.1	4.87	t / c	|AHA11, HA11 | H(+)-ATPase 11 | chr5:25159495
435	AT4G35470.1	4.87	t / c	|PIRL4 | plant intracellular ras group-relate
430	AT4G34450.1	4.70	t / c	| | coatomer gamma-2 subunit, putative / gamm
826	AT5G46580.1	4.66	t / c	| | pentatricopeptide (PPR) repeat-containing
1538	AT1G02150.1	4.64	t / c	| | Tetratricopeptide repeat (TPR)-like super
348	AT2G15620.1	4.63	t / c	|NIR1, NIR, ATHNIR | nitrite reductase 1 | ch
657	AT2G20580.1	4.59	t / c	|RPN1A, ATRPN1A | 26S proteasome regulatory s
718	AT1G17840.1	4.56	t / c	|WBC11, ABCG11, DSO, COF1, ATWBC11 | white-br
754	AT1G59870.1	4.55	t / c	|PEN3, PDR8, ATPDR8, ABCG36, ATABCG36 | ABC-2
1000	AT3G46970.1	4.52	t / c	|ATPHS2, PHS2 | alpha-glucan phosphorylase 2
141	AT3G52750.1	4.50	t / c	|FTSZ2-2 | Tubulin/FtsZ family protein | chr3
152	AT5G57350.1 (+1)	4.50	t / c	|AHA3, ATAHA3, HA3 | H(+)-ATPase 3 | chr5:232
1562	AT3G14840.2	4.46	t / c	| | Leucine-rich repeat transmembrane protein
527	AT1G06410.1	4.44	t / c	|ATTPS7, TPS7, ATTPSA | trehalose-phosphatase

	Accession.Number	log2tc	label	Description
1200	AT2G46520.1	4.44	t / c	| | cellular apoptosis susceptibility protein
385	AT2G45960.3	4.44	t-unique	|PIP1B, TMP-A, ATHH2, PIP1;2 | plasma membran
317	AT2G22500.1	4.42	t / c	|UCP5, ATPUMP5, DIC1 | uncoupling protein 5 |
885	AT1G69830.1	4.41	t / c	|ATAMY3, AMY3 | alpha-amylase-like 3 | chr1:2
1400	AT5G16715.1	4.39	t / c	|EMB2247 | ATP binding;valine-tRNA ligases;am
1584	AT5G01920.1	4.39	t / c	|STN8 | Protein kinase superfamily protein |
245	AT3G59350.1 (+1)	4.37	t / c	| | Protein kinase superfamily protein | chr3
529	AT5G66680.1	4.37	t / c	|DGL1 | dolichyl-diphosphooligosaccharide-pro
434	AT1G79050.1	4.35	t / c	| | recA DNA recombination family protein | c
45	AT4G20890.1	4.33	t / c	|TUB9 | tubulin beta-9 chain | chr4:11182218-
111	AT5G46800.1	4.33	t / c	|BOU | Mitochondrial substrate carrier family
359	AT1G54520.1	4.32	t / c	| | unknown protein; FUNCTIONS IN: molecular_
1149	AT2G41560.1	4.32	t / c	|ACA4 | autoinhibited Ca(2+)-ATPase, isoform
88	AT2G36250.1 (+1)	4.29	t / c	|FTSZ2-1, ATFTSZ2-1 | Tubulin/FtsZ family pro
1482	AT3G50950.1 (+1)	4.27	t / c	|ZAR1 | HOPZ-ACTIVATED RESISTANCE 1 | chr3:18
696	AT1G13320.1 (+1)	4.25	t / c	|PP2AA3 | protein phosphatase 2A subunit A3
1070	AT3G25800.1	4.25	t / c	|PDF1, PR 65, PP2AA2 | protein phosphatase 2A
246	AT1G53750.1	4.24	t / c	|RPT1A | regulatory particle triple-A 1A | ch
263	AT1G73110.1	4.24	t / c	| | P-loop containing nucleoside triphosphate
290	AT1G68830.1	4.22	t / c	|STN7 | STT7 homolog STN7 | chr1:25872654-258
1510	AT5G20490.1	4.22	t / c	|XIK, ATXIK, XI-17 | Myosin family protein wi
165	AT5G65720.1	4.21	t / c	|ATNIFS1, NIFS1, NFS1, ATNFS1 | nitrogen fixa
1047	AT1G30360.1	4.20	t / c	|ERD4 | Early-responsive to dehydration stres
1583	AT5G43470.1 (+1)	4.20	t / c	|RPP8, HRT, RCY1 | Disease resistance protein
1585	AT4G23650.1	4.20	t / c	|CDPK6, CPK3 | calcium-dependent protein kina
471	AT1G56500.1	4.17	t / c	| | haloacid dehalogenase-like hydrolase fami
595	AT5G05010.1 (+1)	4.17	t / c	| | clathrin adaptor complexes medium subunit
1526	AT5G23860.1 (+1)	4.16	t / c	|TUB8 | tubulin beta 8 | chr5:8042962-8044528
616	AT2G47650.1	4.15	t / c	|UXS4 | UDP-xylose synthase 4 | chr2:19538751
722	AT3G62830.1 (+1)	4.15	t / c	|UXS2, ATUXS2, AUD1 | NAD(P)-binding Rossmann

	Accession.Number	log2tc	label	Description
425	AT4G10790.1	4.14	t / c	| | UBX domain-containing protein | chr4:6640
1017	AT5G38660.2	4.14	t / c	|APE1 | acclimation of photosynthesis to env
229	AT1G78570.1	4.13	t / c	|RHM1, ROL1, ATRHM1 | rhamnose biosynthesis 1
158	AT1G80480.1	4.12	t / c	|PTAC17 | plastid transcriptionally active 17
760	AT3G27240.1	4.12	t / c	| | Cytochrome C1 family | chr3:10056144-1005
1066	AT5G57110.1 (+1)	4.12	t / c	|ACA8, AT-ACA8 | autoinhibited Ca2+ -ATPase,
1168	AT1G18270.3	4.09	t / c	| | ketose-bisphosphate aldolase class-II fam
1621	AT1G05150.1	4.09	t / c	| | Calcium-binding tetratricopeptide family
160	AT5G58290.1	4.08	t / c	|RPT3 | regulatory particle triple-A ATPase 3
296	AT3G54110.1	4.08	t / c	|ATPUMP1, UCP, PUMP1, ATUCP1, UCP1 | plant un
262	AT3G59780.1	4.07	t / c	| | Rhodanese/Cell cycle control phosphatase
994	AT3G29320.1	4.07	t / c	| | Glycosyl transferase, family 35 | chr3:11
1323	AT5G08540.1	4.06	t / c	| | unknown protein; FUNCTIONS IN: molecular_
856	AT4G28470.1	4.04	t / c	|RPN1B, ATRPN1B | 26S proteasome regulatory s
639	AT4G00630.1	4.03	t / c	|KEA2, ATKEA2 | K+ efflux antiporter 2 | chr4
1614	AT2G39010.1	4.03	t / c	|PIP2E, PIP2;6 | plasma membrane intrinsic pr
410	AT3G63410.1	4.00	t / c	|APG1, VTE3, IEP37, E37 | S-adenosyl-L-methio
274	AT5G19990.1	3.99	t / c	|RPT6A, ATSUG1 | regulatory particle triple-A
523	AT4G29040.1	3.99	t / c	|RPT2a | regulatory particle AAA-ATPase 2A |
452	AT3G63260.1	3.97	t / c	|ATMRK1 | Protein kinase superfamily protein
477	AT1G06700.1 (+1)	3.97	t / c	| | Protein kinase superfamily protein | chr1
1511	AT5G22800.1	3.94	t / c	|EMB86, EMB1030, EMB263 | Alanyl-tRNA synthet
203	AT2G47110.1 (+1)	3.93	t / c	|UBQ6 | ubiquitin 6 | chr2:19344701-19345174
489	AT2G41790.1	3.93	t / c	| | Insulinase (Peptidase family M16) family
697	AT2G32730.1	3.93	t / c	| | 26S proteasome regulatory complex, non-AT
824	AT5G58260.1	3.93	t / c	| | oxidoreductases, acting on NADH or NADPH,
674	AT4G19710.2	3.91	t / c	|AK-HSDH II, AK-HSDH | aspartate kinase-homos
690	AT1G50480.1	3.91	t / c	|THFS | 10-formyltetrahydrofolate synthetase
835	AT4G17090.1	3.91	t / c	|CT-BMY, BAM3, BMY8 | chloroplast beta-amylas
1036	AT1G01960.1	3.91	t / c	|EDA10 | SEC7-like guanine nucleotide exchang

	Accession.Number	log2tc	label	Description
1652	AT3G09740.1	3.91	t / c	|SYP71, ATSYP71 | syntaxin of plants 71 | chr
341	AT1G10510.1	3.89	t / c	|emb2004 | RNI-like superfamily protein | chr
40	AT2G29550.1	3.87	t / c	|TUB7 | tubulin beta-7 chain | chr2:12644258-
1357	AT2G31880.1	3.87	t / c	|SOBIR1, EVR | Leucine-rich repeat protein ki
31	AT5G44340.1	3.86	t / c	|TUB4 | tubulin beta chain 4 | chr5:17859442-
891	AT5G51820.1	3.85	t / c	|PGM, ATPGMP, PGM1, STF1 | phosphoglucomutase
391	AT5G22060.1	3.84	t / c	|ATJ2, J2 | DNAJ homologue 2 | chr5:7303798-7
1622	AT2G32450.1	3.84	t / c	| | Calcium-binding tetratricopeptide family
624	AT4G24190.1	3.83	t / c	|SHD, HSP90.7, AtHsp90.7, AtHsp90-7 | Chapero
340	AT1G70410.2	3.82	t / c	|ATBCA4, BCA4 | beta carbonic anhydrase 4 | c
35	AT3G08580.1 (+1)	3.81	t / c	|AAC1 | ADP/ATP carrier 1 | chr3:2605706-2607
1290	AT4G35830.1	3.81	t / c	|ACO1 | aconitase 1 | chr4:16973007-16977949
1674	AT1G60780.1	3.81	t / c	|HAP13 | Clathrin adaptor complexes medium su
301	AT3G48750.1	3.79	t / c	|CDKA;1, CDC2AAT, CDK2, CDC2, CDC2A, CDKA1 |
147	AT5G54770.1	3.78	t / c	|THI1, TZ, THI4 | thiazole biosynthetic enzym
326	AT1G45000.1	3.78	t / c	| | AAA-type ATPase family protein | chr1:170
1582	AT1G52290.1	3.77	t-unique	| | Protein kinase superfamily protein | chr1
314	AT5G55280.1	3.77	t / c	|FTSZ1-1, ATFTSZ1-1, CPFTSZ | homolog of bact
715	AT5G55610.1	3.77	t / c	| | unknown protein; LOCATED IN: mitochondrio
860	AT4G28080.1	3.77	t / c	| | Tetratricopeptide repeat (TPR)-like super
1076	AT3G26710.1	3.77	t / c	|CCB1 | cofactor assembly of complex C | chr3
82	AT1G06950.1	3.76	t / c	|ATTIC110, TIC110 | translocon at the inner e
603	AT4G16390.1	3.76	t / c	|SVR7 | pentatricopeptide (PPR) repeat-contai
26	AT5G62690.1 (+1)	3.74	t / c	|TUB2 | tubulin beta chain 2 | chr5:25181560-
408	AT1G80410.2	3.74	t / c	|EMB2753 | tetratricopeptide repeat (TPR)-con
776	AT5G05170.1	3.74	t / c	|CESA3, IXR1, ATCESA3, ATH-B, CEV1 | Cellulos
254	AT1G64740.1	3.72	t / c	|TUA1 | alpha-1 tubulin | chr1:24050114-24052
345	AT5G16070.1	3.72	t / c	| | TCP-1/cpn60 chaperonin family protein | c
73	AT5G14040.1	3.71	t / c	|PHT3;1 | phosphate transporter 3;1 | chr5:45
539	AT1G74960.1 (+2)	3.71	t / c	|FAB1, KAS2, ATKAS2 | fatty acid biosynthesis

	Accession.Number	log2tc	label	Description
570	AT1G01320.2	3.71	t / c	| | Tetratricopeptide repeat (TPR)-like super
1344	AT5G61020.1	3.71	t / c	|ECT3 | evolutionarily conserved C-terminal r
840	AT1G03160.1	3.70	t / c	|FZL | FZO-like | chr1:761321-766053 FORWARD
1119	AT2G44160.1	3.70	t / c	|MTHFR2 | methylenetetrahydrofolate reductase
1427	AT3G46740.1	3.70	t / c	|TOC75-III, MAR1 | translocon at the outer en
123	AT3G05530.1	3.69	t / c	|RPT5A, ATS6A.2 | regulatory particle triple-
265	AT4G22890.1 (+2)	3.68	t / c	|PGR5-LIKE A | PGR5-LIKE A | chr4:12007157-12
175	AT3G19820.1 (+2)	3.66	t / c	|DWF1, DIM, EVE1, DIM1, CBB1 | cell elongatio
889	AT3G23820.1	3.66	t / c	|GAE6 | UDP-D-glucuronate 4-epimerase 6 | chr
1303	AT4G29900.1	3.66	t / c	|ACA10, CIF1, ATACA10 | autoinhibited Ca(2+)-
1524	AT1G30470.1	3.66	t / c	| | SIT4 phosphatase-associated family protei
48	AT5G12250.1	3.65	t / c	|TUB6 | beta-6 tubulin | chr5:3961317-3962971
1478	AT5G27380.1	3.65	t / c	|GSH2, GSHB | glutathione synthetase 2 | chr5
136	AT5G50850.1	3.63	t / c	|MAB1 | Transketolase family protein | chr5:2
239	AT1G79040.1	3.63	t / c	|PSBR | photosystem II subunit R | chr1:29736
1627	AT5G48880.2 (+1)	3.63	t / c	|PKT2, KAT5 | peroxisomal 3-keto-acyl-CoA thi
211	AT5G60790.1	3.62	t / c	|ATGCN1, GCN1 | ABC transporter family protei
420	AT2G26250.1	3.62	t / c	|FDH, KCS10 | 3-ketoacyl-CoA synthase 10 | ch
654	AT2G19860.1	3.62	t / c	|ATHXK2, HXK2 | hexokinase 2 | chr2:8570818-8
1136	AT3G44340.1	3.62	t / c	|CEF | clone eighty-four | chr3:16011923-1601
1638	AT5G13630.1	3.62	t / c	|GUN5, CCH, CHLH, CCH1, ABAR | magnesium-chel
394	AT2G38040.1 (+1)	3.61	t / c	|CAC3 | acetyl Co-enzyme a carboxylase carbox
497	AT1G73990.1	3.61	t / c	|SPPA, SPPA1 | signal peptide peptidase | chr
855	AT5G17380.1	3.60	t / c	| | Thiamine pyrophosphate dependent pyruvate
30	AT1G20010.1	3.59	t / c	|TUB5 | tubulin beta-5 chain | chr1:6938033-6
181	AT1G22410.1	3.59	t / c	| | Class-II DAHP synthetase family protein |
118	AT5G19760.1	3.58	t / c	| | Mitochondrial substrate carrier family pr
303	AT2G32080.1 (+1)	3.58	t / c	|PUR ALPHA-1 | purin-rich alpha 1 | chr2:1364
1234	AT1G31230.1	3.58	t / c	|AK-HSDH I, AK-HSDH | aspartate kinase-homose
1688	AT2G18730.1	3.58	t / c	|ATDGK3, DGK3 | diacylglycerol kinase 3 | chr

	Accession.Number	log2tc	label	Description
292	AT4G25080.4	3.57	t / c	|CHLM | magnesium-protoporphyrin IX methyltra
209	AT5G46290.1	3.56	t / c	|KASI, KAS1 | 3-ketoacyl-acyl carrier protein
505	AT5G03940.1	3.56	t / c	|FFC, 54CP, CPSRP54, SRP54CP | chloroplast si
643	AT1G53500.1	3.56	t / c	|MUM4, RHM2, ATRHM2, ATMUM4 | NAD-dependent e
200	AT4G39980.1	3.54	t / c	|DHS1 | 3-deoxy-D-arabino-heptulosonate 7-pho
284	AT1G23190.1	3.54	t / c	|PGM3 | Phosphoglucomutase/phosphomannomutase
492	AT2G04842.1	3.54	t / c	|EMB2761 | threonyl-tRNA synthetase, putative
493	AT5G03880.1	3.54	t / c	| | Thioredoxin family protein | chr5:1038674
601	AT5G24690.1	3.54	t / c	| | Protein of unknown function (DUF3411) | c
774	AT4G25960.1	3.54	t / c	|PGP2 | P-glycoprotein 2 | chr4:13177438-1318
1397	AT1G53440.1	3.54	t / c	| | Leucine-rich repeat transmembrane protein
1398	AT4G04040.1	3.54	t / c	|MEE51 | Phosphofructokinase family protein |
247	AT1G72150.1	3.51	t / c	|PATL1 | PATELLIN 1 | chr1:27148558-27150652
1319	AT5G54160.1	3.50	t-unique	|ATOMT1, OMT1 | O-methyltransferase 1 | chr5:
511	AT3G01290.1	3.50	t / c	| | SPFH/Band 7/PHB domain-containing membran
512	AT3G01060.1	3.50	t / c	| | unknown protein; Has 640 Blast hits to 63
879	AT5G64940.1 (+1)	3.50	t / c	|ATATH13, ATH13, ATOSA1, OSA1 | ABC2 homolog
275	AT4G35100.1 (+1)	3.49	t / c	|PIP3, PIP3A, PIP2;7, SIMIP | plasma membrane
322	AT5G23540.1	3.48	t / c	| | Mov34/MPN/PAD-1 family protein | chr5:793
360	AT2G32480.1	3.48	t / c	|ARASP | ARABIDOPSIS SERIN PROTEASE | chr2:13
584	AT1G20200.1	3.46	t / c	|EMB2719, HAP15 | PAM domain (PCI/PINT associ
620	AT5G52320.1	3.46	t / c	|CYP96A4 | cytochrome P450, family 96, subfam
691	AT5G19690.1	3.46	t / c	|STT3A | staurosporin and temperature sensiti
1207	AT5G23630.1	3.46	t / c	|PDR2, MIA | phosphate deficiency response 2
1685	AT1G25490.1	3.46	t / c	|RCN1, REGA, ATB BETA BETA, EER1 | ARM repeat
395	AT1G03475.1	3.45	t / c	|LIN2, HEMF1, ATCPO-I | Coproporphyrinogen II
419	AT5G19550.1	3.45	t / c	|ASP2, AAT2 | aspartate aminotransferase 2 |
874	AT1G79600.1	3.45	t / c	| | Protein kinase superfamily protein | chr1
1477	AT3G19480.1	3.45	t / c	| | D-3-phosphoglycerate dehydrogenase | chr3
134	AT3G54890.1	3.42	t / c	|LHCA1 | photosystem I light harvesting compl

	Accession.Number	log2tc	label	Description
578	AT4G21150.1 (+1)	3.42	t / c	|HAP6 | ribophorin II (RPN2) family protein |
927	AT3G10380.1	3.42	t / c	|SEC8, ATSEC8 | subunit of exocyst complex 8
1020	AT5G66200.1	3.42	t / c	|ARO2 | armadillo repeat only 2 | chr5:264536
1231	AT5G04130.1	3.42	t / c	|GYRB2 | DNA GYRASE B2 | chr5:1122084-1128031
1459	AT4G24330.1	3.42	t / c	| | Protein of unknown function (DUF1682) | c
1516	AT1G45474.1 (+1)	3.42	t-unique	|LHCA5 | photosystem I light harvesting compl
184	AT2G30950.1	3.41	t / c	|VAR2, FTSH2 | FtsH extracellular protease fa
294	AT1G52510.1	3.41	t / c	| | alpha/beta-Hydrolases superfamily protein
286	AT1G64190.1	3.40	t / c	| | 6-phosphogluconate dehydrogenase family p
319	AT5G41670.1 (+1)	3.40	t / c	| | 6-phosphogluconate dehydrogenase family p
255	AT4G33510.1	3.39	t / c	|DHS2 | 3-deoxy-d-arabino-heptulosonate 7-pho
533	AT1G57720.1 (+1)	3.39	t / c	| | Translation elongation factor EF1B, gamma
556	AT5G17990.1	3.39	t / c	|TRP1, pat1 | tryptophan biosynthesis 1 | chr
1101	AT3G07100.1	3.39	t / c	|ERMO2, SEC24A | Sec23/Sec24 protein transpor
124	AT3G14210.1	3.38	t / c	|ESM1 | epithiospecifier modifier 1 | chr3:47
178	AT1G09130.3	3.38	t / c	| | ATP-dependent caseinolytic (Clp) protease
582	AT1G06530.1	3.37	t / c	| | Tropomyosin-related | chr1:2001625-200259
929	AT2G47450.1	3.37	t / c	|CAO, CPSRP43 | chloroplast signal recognitio
1593	AT3G62010.2	3.37	t / c	| | unknown protein; LOCATED IN: cellular_com
1596	AT1G65260.1	3.37	t / c	|PTAC4, VIPP1 | plastid transcriptionally act
1629	AT5G06530.1 (+1)	3.37	t / c	| | ABC-2 type transporter family protein | c
1656	AT2G37710.1	3.37	t / c	|RLK | receptor lectin kinase | chr2:15814934
2121	AT3G22640.1	3.37	t / c	|PAP85 | cupin family protein | chr3:8011902-
1277	AT1G51500.1	3.37	t-unique	|CER5, D3, ABCG12, WBC12, ATWBC12 | ABC-2 typ
615	AT1G79560.1	3.36	t / c	|EMB156, EMB36, EMB1047, FTSH12 | FTSH protea
751	AT3G53520.4	3.36	t / c	|UXS1 | UDP-glucuronic acid decarboxylase 1 |
757	AT4G34090.2	3.36	t / c	| | unknown protein; FUNCTIONS IN: molecular_
1489	AT4G38630.1	3.36	t / c	|RPN10, MCB1, ATMCB1, MBP1 | regulatory parti
180	AT3G44110.1	3.35	t / c	|ATJ3, ATJ | DNAJ homologue 3 | chr3:15869115
399	AT2G33150.1	3.35	t / c	|PKT3, PED1, KAT2 | peroxisomal 3-ketoacyl-Co

	Accession.Number	log2tc	label	Description
201	AT2G44490.1	3.34	t / c	|PEN2, BGLU26 | Glycosyl hydrolase superfamil
236	AT3G19170.1	3.34	t / c	|ATPREP1, ATZNMP, PREP1 | presequence proteas
427	AT5G14780.1	3.34	t / c	|FDH | formate dehydrogenase | chr5:4777043-4
622	AT1G04810.1	3.34	t / c	| | 26S proteasome regulatory complex, non-AT
190	AT2G04030.2	3.33	t / c	|CR88, Hsp88.1, AtHsp90.5 | Chaperone protein
1759	AT4G37200.1	3.32	t-unique	|HCF164 | Thioredoxin superfamily protein | c
332	AT4G01100.1	3.32	t / c	|ADNT1 | adenine nucleotide transporter 1 | c
370	AT4G30010.1	3.32	t / c	| | unknown protein; FUNCTIONS IN: molecular_
524	AT3G63460.1	3.32	t / c	| | transducin family protein / WD-40 repeat
930	AT2G27600.1	3.32	t / c	|SKD1, VPS4, ATSKD1 | AAA-type ATPase family
971	AT3G07160.1	3.32	t / c	|ATGSL10, gsl10, CALS9 | glucan synthase-like
65	AT1G09340.1	3.31	t / c	|CRB, CSP41B, HIP1.3 | chloroplast RNA bindin
92	AT1G50250.1	3.29	t / c	|FTSH1 | FTSH protease 1 | chr1:18614398-1861
159	ATCG00500.1	3.29	t / c	|ACCD | acetyl-CoA carboxylase carboxyl trans
1102	AT3G43300.1	3.29	t / c	|ATMIN7, BEN1 | HOPM interactor 7 | chr3:1523
125	AT4G35250.1	3.28	t / c	| | NAD(P)-binding Rossmann-fold superfamily
168	AT2G19940.1 (+1)	3.28	t / c	| | oxidoreductases, acting on the aldehyde o
1148	AT3G61050.1 (+1)	3.28	t / c	|NTMC2TYPE4, NTMC2T4 | Calcium-dependent lipi
1728	AT3G28710.1	3.27	t-unique	| | ATPase, V0/A0 complex, subunit C/D | chr3
478	AT3G02360.1 (+1)	3.27	t / c	| | 6-phosphogluconate dehydrogenase family p
785	AT1G29310.1	3.27	t / c	| | SecY protein transport family protein | c
813	AT5G20890.1	3.27	t / c	| | TCP-1/cpn60 chaperonin family protein | c
910	AT2G05710.1	3.27	t / c	|ACO3 | aconitase 3 | chr2:2141591-2146350 FO
966	AT5G64740.1	3.27	t / c	|CESA6, IXR2, E112, PRC1 | cellulose synthase
1179	AT5G58410.1	3.27	t / c	| | HEAT/U-box domain-containing protein | ch
1309	AT4G32410.1	3.27	t / c	|CESA1, RSW1, AtCESA1 | cellulose synthase 1
1748	AT5G02890.1	3.27	t / c	| | HXXXD-type acyl-transferase family protei
1749	AT4G30340.1	3.27	t / c	|ATDGK7, DGK7 | diacylglycerol kinase 7 | chr
1766	AT2G34560.1	3.27	t / c	| | P-loop containing nucleoside triphosphate
243	AT3G55360.1	3.25	t / c	|CER10, ECR, ATTSC13, TSC13 | 3-oxo-5-alpha-s

	Accession.Number	log2tc	label	Description
540	AT4G34200.1	3.25	t / c	|EDA9 | D-3-phosphoglycerate dehydrogenase |
827	AT3G24430.1	3.25	t / c	|HCF101 | ATP binding | chr3:8868731-8872154
1705	AT1G34000.1	3.22	t-unique	|OHP2 | one-helix protein 2 | chr1:12358151-1
192	AT5G12470.1	3.22	t / c	| | Protein of unknown function (DUF3411) | c
780	AT3G19960.1	3.22	t / c	|ATM1 | myosin 1 | chr3:6949787-6956736 FORWA
878	AT5G22770.1 (+2)	3.22	t / c	|alpha-ADR | alpha-adaptin | chr5:7579844-758
1144	AT3G23750.1	3.22	t / c	| | Leucine-rich repeat protein kinase family
1214	AT5G43900.3	3.22	t / c	|MYA2 | myosin 2 | chr5:17657241-17667413 REV
1281	AT3G28860.1	3.22	t / c	|ATMDR1, ATMDR11, PGP19, MDR11, MDR1, ATPGP19
1302	AT1G70320.1	3.22	t / c	|UPL2 | ubiquitin-protein ligase 2 | chr1:264
1418	AT1G01220.1	3.22	t / c	|FKGP, AtFKGP | L-fucokinase/GDP-L-fucose pyr
1729	AT5G58670.1	3.22	t / c	|ATPLC1, ATPLC, PLC1 | phospholipase C1 | chr
1760	AT3G45190.1	3.22	t / c	| | SIT4 phosphatase-associated family protei
1795	AT1G64430.1 (+1)	3.22	t / c	| | Pentatricopeptide repeat (PPR) superfamil
365	AT1G54780.1	3.21	t / c	|TLP18.3 | thylakoid lumen 18.3 kDa protein |
520	AT2G33040.1	3.21	t / c	|ATP3 | gamma subunit of Mt ATP synthase | ch
84	AT1G74470.1	3.20	t / c	| | Pyridine nucleotide-disulphide oxidoreduc
766	AT1G80030.1 (+2)	3.20	t / c	| | Molecular chaperone Hsp40/DnaJ family pro
306	AT3G63160.1	3.19	t / c	| | FUNCTIONS IN: molecular_function unknown;
590	AT5G40770.1	3.18	t / c	|ATPHB3, PHB3 | prohibitin 3 | chr5:16315589-
655	AT1G15730.1	3.18	t / c	| | Cobalamin biosynthesis CobW-like protein
1387	AT5G27390.1	3.18	t / c	| | Mog1/PsbP/DUF1795-like photosystem II rea
369	AT1G70730.1	3.17	t / c	|PGM2 | Phosphoglucomutase/phosphomannomutase
565	ATCG00430.1	3.17	t / c	|PSBG | photosystem II reaction center protei
682	AT3G51140.1	3.17	t / c	| | Protein of unknown function (DUF3353) | c
772	AT2G20990.3	3.17	t / c	|SYTA | synaptotagmin A | chr2:9014827-901782
908	AT5G22330.1	3.17	t / c	|ATTIP49A, RIN1 | P-loop containing nucleosid
1099	AT4G16990.2	3.17	t / c	|RLM3 | disease resistance protein (TIR-NBS c
1124	AT2G43950.1	3.17	t / c	|OEP37, ATOEP37 | chloroplast outer envelope
1199	AT1G78915.1 (+2)	3.17	t / c	| | Tetratricopeptide repeat (TPR)-like super

	Accession.Number	log2tc	label	Description
1514	AT1G21250.1	3.17	t / c	|WAK1, PRO25 | cell wall-associated kinase |
1632	AT1G07650.2	3.17	t / c	| | Leucine-rich repeat transmembrane protein
1721	AT3G18190.1	3.17	t / c	| | TCP-1/cpn60 chaperonin family protein | c
485	AT1G22530.1	3.17	t-unique	|PATL2 | PATELLIN 2 | chr1:7955773-7958326 RE
1871	AT3G23660.1	3.17	t-unique	| | Sec23/Sec24 protein transport family prot
1592	AT4G39960.1	3.16	t / c	| | Molecular chaperone Hsp40/DnaJ family pro
935	AT1G70940.1	3.15	t / c	|PIN3, ATPIN3 | Auxin efflux carrier family p
1556	AT5G52520.1	3.15	t / c	|OVA6, PRORS1 | Class II aaRS and biotin synt
120	AT5G25460.1	3.14	t / c	| | Protein of unknown function, DUF642 | chr
146	AT1G71500.1	3.14	t / c	| | Rieske (2Fe-2S) domain-containing protein
312	AT2G42210.2	3.14	t / c	|ATOEP16-3, OEP16-3 | Mitochondrial import in
576	AT1G34430.1	3.14	t / c	|EMB3003 | 2-oxoacid dehydrogenases acyltrans
1604	AT1G70770.1 (+1)	3.14	t / c	| | Protein of unknown function DUF2359, tran
1619	AT2G45710.1	3.14	t / c	| | Zinc-binding ribosomal protein family pro
70	AT5G42270.1	3.13	t / c	|VAR1, FTSH5 | FtsH extracellular protease fa
151	AT5G11420.1	3.12	t / c	| | Protein of unknown function, DUF642 | chr
354	AT2G44640.1	3.12	t / c	| | FUNCTIONS IN: molecular_function unknown;
938	AT3G62700.1	3.12	t / c	|ATMRP10, MRP10, ABCC14 | multidrug resistanc
1112	AT2G40840.1	3.12	t / c	|DPE2 | disproportionating enzyme 2 | chr2:17
1366	AT4G26300.1	3.12	t / c	|emb1027 | Arginyl-tRNA synthetase, class Ic
1739	AT4G12320.1	3.12	t / c	|CYP706A6 | cytochrome P450, family 706, subf
1778	AT3G63520.1	3.12	t / c	|CCD1, ATCCD1, ATNCED1, NCED1 | carotenoid cl
688	AT1G01610.1	3.12	t-unique	|ATGPAT4, GPAT4 | glycerol-3-phosphate acyltr
328	AT1G06430.1	3.11	t / c	|FTSH8 | FTSH protease 8 | chr1:1960214-19625
454	AT1G76030.1	3.11	t / c	| | ATPase, V1 complex, subunit B protein | c
488	AT5G17170.1	3.11	t / c	|ENH1 | rubredoxin family protein | chr5:5649
561	AT5G05780.1	3.11	t / c	|RPN8A, AE3, ATHMOV34 | RP non-ATPase subunit
803	AT4G04770.1	3.11	t / c	|ATABC1, LAF6, ATNAP1, ABC1 | ATP binding cas
846	AT3G20000.1	3.11	t / c	|TOM40 | translocase of the outer mitochondri
235	AT4G03280.1	3.10	t / c	|PETC, PGR1 | photosynthetic electron transfe

	Accession.Number	log2tc	label	Description
388	AT1G05140.1	3.10	t / c	| | Peptidase M50 family protein | chr1:14826
1192	AT3G61470.1	3.10	t / c	|LHCA2 | photosystem I light harvesting compl
1637	AT1G09620.1	3.10	t / c	| | ATP binding;leucine-tRNA ligases;aminoacy
797	AT1G51805.1	3.09	t / c	| | Leucine-rich repeat protein kinase family
451	AT1G30380.1	3.08	t / c	|PSAK | photosystem I subunit K | chr1:107223
746	AT1G51980.1	3.08	t / c	| | Insulinase (Peptidase family M16) protein
42	AT1G29930.1	3.06	t / c	|CAB1, AB140, CAB140, LHCB1.3 | chlorophyll A
386	AT1G16300.1	3.06	t / c	|GAPCP-2 | glyceraldehyde-3-phosphate dehydro
659	AT2G31810.1	3.06	t / c	| | ACT domain-containing small subunit of ac
789	AT1G04530.1	3.06	t / c	|TPR4 | Tetratricopeptide repeat (TPR)-like s
802	AT5G45510.2	3.06	t / c	| | Leucine-rich repeat (LRR) family protein
906	AT5G09870.1	3.06	t / c	|CESA5 | cellulose synthase 5 | chr5:3073356-
1074	AT1G62640.1 (+1)	3.06	t / c	|KAS III | 3-ketoacyl-acyl carrier protein sy
1150	AT5G03040.1 (+2)	3.06	t / c	|iqd2 | IQ-domain 2 | chr5:710380-712406 REVE
1223	AT3G16950.1	3.06	t / c	|LPD1, ptlpd1 | lipoamide dehydrogenase 1 | c
1464	AT3G04340.1	3.06	t / c	|emb2458 | FtsH extracellular protease family
1468	AT4G21710.1	3.06	t / c	|NRPB2, EMB1989, RPB2 | DNA-directed RNA poly
1600	AT1G53430.1 (+1)	3.06	t / c	| | Leucine-rich repeat transmembrane protein
1684	AT3G51160.1	3.06	t / c	|MUR1, MUR_1, GMD2 | NAD(P)-binding Rossmann-
1702	AT2G27730.1	3.06	t / c	| | copper ion binding | chr2:11820056-118208
1780	AT2G29200.1	3.06	t / c	|APUM1, PUM1 | pumilio 1 | chr2:12549483-1255
1056	AT4G08850.1	3.06	t-unique	| | Leucine-rich repeat receptor-like protein
210	ATCG01110.1	3.05	t / c	|NDHH | NAD(P)H dehydrogenase subunit H | chr
649	AT4G38510.5	3.05	t / c	| | ATPase, V1 complex, subunit B protein | c
756	AT2G22360.1	3.03	t / c	| | DNAJ heat shock family protein | chr2:949
1159	AT4G11420.1	3.03	t / c	|EIF3A, ATEIF3A-1, EIF3A-1, ATTIF3A1, TIF3A1
278	AT4G31990.1 (+2)	3.02	t / c	|ASP5, AAT3, ATAAT1 | aspartate aminotransfer
101	AT1G23310.1	3.01	t / c	|GGT1, AOAT1, GGAT1 | glutamate:glyoxylate am
431	AT3G20790.1	3.01	t / c	| | NAD(P)-binding Rossmann-fold superfamily
541	AT1G09640.1	3.01	t / c	| | Translation elongation factor EF1B, gamma

	Accession.Number	log2tc	label	Description
1165	AT4G02510.1	3.01	t / c	|TOC159, TOC86, PPI2, TOC160, ATTOC159 | tran
644	AT1G11410.1	3.00	t / c	| | S-locus lectin protein kinase family prot
894	AT1G11260.1	3.00	t-unique	|STP1, ATSTP1 | sugar transporter 1 | chr1:37
939	AT2G27860.1	3.00	t / c	|AXS1 | UDP-D-apiose/UDP-D-xylose synthase 1
1041	AT2G29190.1 (+1)	3.00	t / c	|APUM2, PUM2 | pumilio 2 | chr2:12544260-1254
1083	AT1G08930.1 (+1)	3.00	t / c	|ERD6 | Major facilitator superfamily protein
1608	AT5G38990.1	3.00	t / c	| | Malectin/receptor-like protein kinase fam
1747	AT5G02160.1	3.00	t / c	| | unknown protein; FUNCTIONS IN: molecular_
231	AT5G58330.2	2.98	t / c	| | lactate/malate dehydrogenase family prote
503	AT4G30950.1	2.97	t / c	|FAD6, FADC, SFD4 | fatty acid desaturase 6 |
468	AT3G51820.1	2.96	t / c	|ATG4, G4, CHLG | UbiA prenyltransferase fami
1590	AT3G01280.1	2.96	t / c	|VDAC1, ATVDAC1 | voltage dependent anion cha
1628	AT3G02450.1	2.96	t / c	| | cell division protein ftsH, putative | ch
1687	AT5G49810.1	2.96	t / c	|MMT | methionine S-methyltransferase | chr5:
105	AT3G47470.1	2.95	t / c	|LHCA4, CAB4 | light-harvesting chlorophyll-p
1665	AT1G72750.1	2.95	t / c	|ATTIM23-2, TIM23-2 | translocase inner membr
85	AT5G23060.1	2.94	t / c	|CaS | calcium sensing receptor | chr5:773676
648	AT1G70520.1	2.94	t / c	|CRK2 | cysteine-rich RLK (RECEPTOR-like prot
866	AT2G47240.1 (+1)	2.94	t / c	|CER8, LACS1 | AMP-dependent synthetase and l
1019	AT4G34830.1	2.94	t / c	|MRL1 | Pentatricopeptide repeat (PPR) superf
1343	AT5G49030.3	2.94	t / c	|OVA2 | tRNA synthetase class I (I, L, M and
1745	AT3G11830.1	2.94	t / c	| | TCP-1/cpn60 chaperonin family protein | c
1852	AT4G04850.2	2.94	t / c	|KEA3 | K+ efflux antiporter 3 | chr4:2453174
106	AT4G27440.1 (+1)	2.93	t / c	|PORB | protochlorophyllide oxidoreductase B
1451	AT3G46060.1 (+2)	2.93	t / c	|ARA3, ARA-3, ATRABE1C, ATRAB8A, RAB8A | RAB
138	AT2G38230.1	2.92	t / c	|ATPDX1.1, PDX1.1 | pyridoxine biosynthesis 1
564	AT1G65960.2	2.92	t / c	|GAD2 | glutamate decarboxylase 2 | chr1:2455
598	AT1G18500.1	2.92	t / c	|MAML-4, IPMS1 | methylthioalkylmalate syntha
414	AT1G14810.1	2.91	t / c	| | semialdehyde dehydrogenase family protein
804	AT1G08380.1	2.91	t / c	|PSAO | photosystem I subunit O | chr1:264100

	Accession.Number	log2tc	label	Description
1269	AT3G10920.2	2.91	t / c	|MSD1 | manganese superoxide dismutase 1 | ch
1529	AT3G23810.1	2.91	t / c	|SAHH2, ATSAHH2 | S-adenosyl-l-homocysteine (
1680	AT5G11770.1	2.91	t / c	| | NADH-ubiquinone oxidoreductase 20 kDa sub
1738	AT4G27700.1	2.91	t / c	| | Rhodanese/Cell cycle control phosphatase
1365	AT4G15550.1	2.87	t-unique	|IAGLU | indole-3-acetate beta-D-glucosyltran
1724	AT4G22310.1	2.87	t-unique	| | Uncharacterised protein family (UPF0041)
1799	AT1G22710.1	2.87	t-unique	|SUC2, SUT1, ATSUC2 | sucrose-proton symporte
946	AT5G64580.1	2.87	t / c	| | AAA-type ATPase family protein | chr5:258
988	AT3G52500.1	2.87	t / c	| | Eukaryotic aspartyl protease family prote
1040	AT4G31500.1	2.87	t / c	|CYP83B1, SUR2, RNT1, RED1, ATR4 | cytochrome
1164	AT1G26850.1 (+1)	2.87	t / c	| | S-adenosyl-L-methionine-dependent methylt
1247	AT4G24810.2	2.87	t / c	| | Protein kinase superfamily protein | chr4
1639	ATCG01060.1	2.87	t / c	|PSAC | iron-sulfur cluster binding;electron
1697	AT1G63000.1	2.87	t / c	|NRS/ER, UER1 | nucleotide-rhamnose synthase/
1710	AT5G59730.2	2.87	t / c	|ATEXO70H7, EXO70H7 | exocyst subunit exo70 f
1723	AT4G23250.1	2.87	t / c	|EMB1290, DUF26-21, RKC1, CRK17 | kinases;pro
1726	AT3G51550.1	2.87	t / c	|FER | Malectin/receptor-like protein kinase
1734	AT1G19450.1	2.87	t / c	| | Major facilitator superfamily protein | c
1754	AT5G60540.1	2.87	t / c	|EMB2407, ATPDX2, PDX2 | pyridoxine biosynthe
41	AT3G12110.1	2.85	t / c	|ACT11 | actin-11 | chr3:3858116-3859609 FORW
631	AT3G07770.1	2.85	t / c	|Hsp89.1, AtHsp90.6, AtHsp90-6 | HEAT SHOCK P
1345	AT1G77590.1	2.85	t / c	|LACS9 | long chain acyl-CoA synthetase 9 | c
71	AT5G19770.1 (+1)	2.84	t / c	|TUA3 | tubulin alpha-3 | chr5:6682761-668447
128	AT3G53420.1 (+1)	2.84	t / c	|PIP2A, PIP2, PIP2;1 | plasma membrane intrin
374	AT3G61440.1	2.84	t / c	|ATCYSC1, ARATH;BSAS3;1, CYSC1 | cysteine syn
522	AT4G39710.1	2.84	t / c	|FKBP16-2 | FK506-binding protein 16-2 | chr4
577	ATCG01090.1	2.84	t / c	|NDHI | NADPH dehydrogenases | chrC:119244-11
1326	AT5G42390.1	2.84	t / c	| | Insulinase (Peptidase family M16) family
1612	AT4G01800.1	2.84	t / c	|AGY1, AtcpSecA, SECA1 | Albino or Glassy Yel
57	AT1G04820.1 (+1)	2.82	t / c	|TUA4, TOR2 | tubulin alpha-4 chain | chr1:13

	Accession.Number	log2tc	label	Description
679	AT4G37925.1	2.82	t / c	|NDH-M | subunit NDH-M of NAD(P)H:plastoquino
546	AT2G45060.1	2.81	t / c	| | Uncharacterised conserved protein UCP0222
557	AT2G35840.1 (+2)	2.81	t / c	| | Sucrose-6F-phosphate phosphohydrolase fam
837	AT3G20810.1 (+1)	2.81	t / c	|JMJD5 | 2-oxoglutarate (2OG) and Fe(II)-depe
863	AT3G01310.2	2.81	t / c	| | Phosphoglycerate mutase-like family prote
914	AT3G53180.1	2.81	t / c	| | glutamate-ammonia ligases;catalytics;glut
1173	AT4G03550.1	2.81	t / c	|ATGSL05, GSL05, ATGSL5, PMR4, GSL5 | glucan
1307	AT2G24180.1	2.81	t / c	|CYP71B6 | cytochrome p450 71b6 | chr2:102818
1624	AT3G18130.1	2.81	t / c	|RACK1C_AT | receptor for activated C kinase
1713	AT2G38670.1	2.81	t / c	|PECT1 | phosphorylethanolamine cytidylyltran
1752	AT4G38580.1	2.81	t / c	|ATFP6, HIPP26, FP6 | farnesylated protein 6
1884	AT5G05200.1	2.81	t / c	| | Protein kinase superfamily protein | chr5
1940	AT3G47620.1	2.81	t / c	|AtTCP14, TCP14 | TEOSINTE BRANCHED, cycloide
1115	AT1G73650.3	2.81	t-unique	| | Protein of unknown function (DUF1295) | c
1813	AT1G21270.1	2.81	t-unique	|WAK2 | wall-associated kinase 2 | chr1:74449
792	AT5G64290.1	2.80	t / c	|DCT, DIT2.1 | dicarboxylate transport 2.1 |
821	AT1G48520.1	2.80	t / c	|GATB | GLU-ADT subunit B | chr1:17940185-179
1401	AT5G13430.1	2.79	t / c	| | Ubiquinol-cytochrome C reductase iron-sul
327	AT2G33210.2	2.77	t / c	|HSP60-2 | heat shock protein 60-2 | chr2:140
494	AT2G22250.2 (+1)	2.77	t / c	|ATAAT, AAT, MEE17 | aspartate aminotransfera
129	AT3G21180.1	2.76	t / c	|ACA9, ATACA9 | autoinhibited Ca(2+)-ATPase 9
234	AT3G56940.1	2.75	t / c	|CRD1, CHL27, ACSF | dicarboxylate diiron pro
8	AT2G39730.1	2.74	t / c	|RCA | rubisco activase | chr2:16570951-16573
113	AT3G08940.2	2.74	t / c	|LHCB4.2 | light harvesting complex photosyst
481	AT1G19920.1	2.74	t / c	|APS2, ASA1 | Pseudouridine synthase/archaeos
671	AT5G67630.1	2.74	t / c	| | P-loop containing nucleoside triphosphate
678	AT2G46820.1 (+1)	2.74	t / c	|PTAC8, TMP14, PSAP, PSI-P | photosystem I P
812	AT3G52730.1	2.74	t / c	| | ubiquinol-cytochrome C reductase UQCRX/QC
964	AT1G17580.1	2.74	t / c	|MYA1, ATMYA1, XI-1 | myosin 1 | chr1:6039453
972	AT2G25800.1	2.74	t / c	| | Protein of unknown function (DUF810) | ch

	Accession.Number	log2tc	label	Description
1052	AT3G57650.1	2.74	t / c	|LPAT2 | lysophosphatidyl acyltransferase 2 |
1167	AT2G47390.1	2.74	t / c	| | Prolyl oligopeptidase family protein | ch
1250	AT5G42240.1	2.74	t / c	|scpl42 | serine carboxypeptidase-like 42 | c
1472	AT5G11380.1	2.74	t / c	|DXPS3 | 1-deoxy-D-xylulose 5-phosphate synth
1647	AT5G42790.1	2.74	t / c	|PAF1, ATPSM30, ARS5 | proteasome alpha subun
1682	AT3G20050.1	2.74	t / c	|ATTCP-1, TCP-1 | T-complex protein 1 alpha s
1686	AT5G36230.1	2.74	t / c	| | ARM repeat superfamily protein | chr5:142
1711	AT5G09900.2	2.74	t / c	|EMB2107, RPN5A, MSA | 26S proteasome regulat
1716	AT4G02620.1	2.74	t / c	| | vacuolar ATPase subunit F family protein
1773	AT4G37000.1	2.74	t / c	|ACD2, ATRCCR | accelerated cell death 2 (ACD
1837	AT1G06000.1	2.74	t / c	| | UDP-Glycosyltransferase superfamily prote
1859	AT3G02350.1	2.74	t / c	|GAUT9 | galacturonosyltransferase 9 | chr3:4
126	AT1G45201.1	2.72	t / c	|ATTLL1, TLL1 | triacylglycerol lipase-like 1
207	AT5G50920.1	2.72	t / c	|CLPC, ATHSP93-V, HSP93-V, DCA1, CLPC1 | CLPC
28	AT5G09810.1	2.71	t / c	|ACT7 | actin 7 | chr5:3052809-3054220 FORWAR
36	AT1G49240.1	2.71	t / c	|ACT8 | actin 8 | chr1:18216539-18217947 FORW
330	AT3G48870.1	2.71	t / c	|ATCLPC, ATHSP93-III, HSP93-III | Clp ATPase
368	AT3G22960.1	2.70	t / c	|PKP1, PKP-ALPHA | Pyruvate kinase family pro
901	AT4G13770.1	2.70	t / c	|CYP83A1, REF2 | cytochrome P450, family 83,
1431	AT3G23400.1	2.70	t / c	|FIB4 | Plastid-lipid associated protein PAP
1623	AT1G52360.1	2.70	t / c	| | Coatomer, beta' subunit | chr1:19499282-1
206	AT4G34670.1	2.69	t / c	| | Ribosomal protein S3Ae | chr4:16548724-16
514	AT5G12860.1 (+1)	2.69	t / c	|DiT1 | dicarboxylate transporter 1 | chr5:40
191	AT1G03630.2	2.68	t / c	|POR C, PORC | protochlorophyllide oxidoreduc
444	AT3G42050.1	2.68	t / c	| | vacuolar ATP synthase subunit H family pr
725	AT1G15980.1	2.68	t / c	|NDF1, NDH48 | NDH-dependent cyclic electron
777	AT3G22845.1	2.68	t / c	| | emp24/gp25L/p24 family/GOLD family protei
816	AT2G23670.1	2.68	t / c	|YCF37 | homolog of Synechocystis YCF37 | chr
1473	AT1G29900.1	2.68	t / c	|CARB | carbamoyl phosphate synthetase B | ch
353	AT2G47730.1	2.67	t / c	|ATGSTF8, ATGSTF5, GST6, GSTF8 | glutathione

	Accession.Number	log2tc	label	Description
1931	AT1G22700.2	2.66	t-unique	| | Tetratricopeptide repeat (TPR)-like super
176	AT3G02090.1	2.66	t / c	|MPPBETA | Insulinase (Peptidase family M16)
272	AT5G23120.1	2.66	t / c	|HCF136 | photosystem II stability/assembly f
403	AT3G58140.1	2.66	t / c	| | phenylalanyl-tRNA synthetase class IIc fa
815	AT5G21430.1	2.66	t / c	| | Chaperone DnaJ-domain superfamily protein
861	AT2G16950.1	2.66	t / c	|TRN1, ATTRN1 | transportin 1 | chr2:7353939-
1146	AT4G10120.1 (+1)	2.66	t / c	|ATSPS4F | Sucrose-phosphate synthase family
1413	AT2G23200.1	2.66	t / c	| | Protein kinase superfamily protein | chr2
1641	AT1G16670.1	2.66	t / c	| | Protein kinase superfamily protein | chr1
1661	AT4G00400.1	2.66	t / c	|GPAT8, AtGPAT8 | glycerol-3-phosphate acyltr
1746	AT1G33810.1	2.66	t / c	| | unknown protein; FUNCTIONS IN: molecular_
1805	AT1G09795.1	2.66	t / c	|ATATP-PRT2, HISN1B, ATP-PRT2 | ATP phosphori
1810	AT1G63770.5	2.66	t / c	| | Peptidase M1 family protein | chr1:236577
1819	AT1G64710.1	2.66	t / c	| | GroES-like zinc-binding dehydrogenase fam
1854	AT2G35780.1	2.66	t / c	|scpl26 | serine carboxypeptidase-like 26 | c
2085	AT1G14930.1	2.66	t / c	| | Polyketide cyclase/dehydrase and lipid tr
49	AT2G37620.1 (+2)	2.65	t / c	|ACT1, AAc1 | actin 1 | chr2:15779761-1578124
1605	AT5G13650.2	2.65	t / c	| | elongation factor family protein | chr5:4
251	AT5G46110.3	2.64	t / c	|APE2, TPT | Glucose-6-phosphate/phosphate tr
763	AT4G23850.1	2.64	t / c	|LACS4 | AMP-dependent synthetase and ligase
1484	AT3G01440.1	2.63	t / c	|PQL1, PQL2 | PsbQ-like 1 | chr3:168478-16940
1667	AT4G24620.1	2.63	t / c	|PGI1, PGI | phosphoglucose isomerase 1 | chr
677	AT3G04790.1	2.62	t / c	| | Ribose 5-phosphate isomerase, type A prot
64	AT1G13440.1	2.61	t / c	|GAPC-2, GAPC2 | glyceraldehyde-3-phosphate d
362	AT2G39770.1 (+1)	2.61	t / c	|CYT1, VTC1, SOZ1, EMB101, GMP1 | Glucose-1-p
669	AT3G15980.1 (+3)	2.61	t / c	| | Coatomer, beta' subunit | chr3:5411699-54
887	AT5G17020.1 (+1)	2.61	t / c	|XPO1A, ATCRM1, ATXPO1, XPO1, HIT2 | exportin
951	AT4G23940.1	2.61	t / c	| | FtsH extracellular protease family | chr4
1379	AT5G45390.1	2.61	t / c	|CLPP4, NCLPP4 | CLP protease P4 | chr5:18396
1662	AT5G14060.1 (+1)	2.61	t / c	|CARAB-AK-LYS | Aspartate kinase family prote

	Accession.Number	log2tc	label	Description
1856	AT5G08530.1	2.61	t / c	|CI51 | 51 kDa subunit of complex I | chr5:27
324	AT5G63570.1	2.60	t / c	|GSA1 | glutamate-1-semialdehyde-2,1-aminomut
506	AT2G18710.1	2.60	t / c	|SCY1 | SECY homolog 1 | chr2:8112231-8114452
58	AT3G04120.1	2.59	t / c	|GAPC, GAPC-1, GAPC1 | glyceraldehyde-3-phosp
198	AT3G62250.1	2.59	t / c	|UBQ5 | ubiquitin 5 | chr3:23037138-23037611
667	AT5G42650.1	2.59	t / c	|AOS, CYP74A, DDE2 | allene oxide synthase |
829	AT3G11710.1	2.59	t / c	|ATKRS-1 | lysyl-tRNA synthetase 1 | chr3:370
956	AT5G13280.1	2.59	t / c	|AK-LYS1, AK1, AK | aspartate kinase 1 | chr5
1079	AT5G65620.1	2.59	t / c	| | Zincin-like metalloproteases family prote
1635	ATCG00670.1	2.59	t / c	|CLPP1, PCLPP | plastid-encoded CLP P | chrC:
1655	AT4G12830.1	2.59	t / c	| | alpha/beta-Hydrolases superfamily protein
630	AT3G25680.1	2.58	t-unique	| | FUNCTIONS IN: molecular_function unknown;
166	AT5G35360.3	2.58	t / c	|CAC2 | acetyl Co-enzyme a carboxylase biotin
379	AT4G01050.1	2.58	t / c	|TROL | thylakoid rhodanese-like | chr4:45587
495	AT3G62530.1	2.58	t / c	| | ARM repeat superfamily protein | chr3:231
534	AT3G58730.1	2.58	t / c	| | vacuolar ATP synthase subunit D (VATD) /
555	AT1G12770.1	2.58	t / c	|ISE1, EMB1586 | P-loop containing nucleoside
1005	AT5G47910.1	2.58	t / c	|RBOHD, ATRBOHD | respiratory burst oxidase h
1381	AT4G00360.1	2.58	t / c	|CYP86A2, ATT1 | cytochrome P450, family 86,
1391	AT1G50430.1 (+1)	2.58	t / c	|DWF5, PA, LE, ST7R, 7RED | Ergosterol biosyn
1707	AT2G28800.1 (+1)	2.58	t / c	|ALB3 | 63 kDa inner membrane family protein
1814	AT4G33220.1	2.58	t / c	|PME44, ATPME44 | pectin methylesterase 44 |
1891	AT4G31390.1	2.58	t / c	| | Protein kinase superfamily protein | chr4
225	AT1G56070.1	2.57	t / c	|LOS1 | Ribosomal protein S5/Elongation facto
607	AT2G33530.1	2.57	t / c	|scpl46 | serine carboxypeptidase-like 46 | c
1094	AT1G71220.1	2.57	t / c	|EBS1, UGGT, PSL2 | UDP-glucose:glycoprotein
216	AT1G09750.1	2.56	t / c	| | Eukaryotic aspartyl protease family prote
223	AT5G15490.1	2.56	t / c	| | UDP-glucose 6-dehydrogenase family protei
436	AT5G28840.1 (+1)	2.56	t / c	|GME | GDP-D-mannose 3’,5’-epimerase | chr5:1
171	AT5G01410.1	2.54	t / c	|PDX1, ATPDX1.3, RSR4, PDX1.3, ATPDX1 | Aldol

	Accession.Number	log2tc	label	Description
338	AT3G18490.1	2.54	t / c	| | Eukaryotic aspartyl protease family prote
371	AT5G30510.1	2.54	t / c	|RPS1, ARRPS1 | ribosomal protein S1 | chr5:1
496	AT1G08520.1	2.54	t / c	|ALB1, ALB-1V, V157, PDE166, CHLD | ALBINA 1
550	AT2G21960.1	2.54	t / c	| | unknown protein; LOCATED IN: chloroplast;
695	AT5G24650.1	2.54	t / c	| | Mitochondrial import inner membrane trans
781	AT1G14150.1	2.54	t / c	|PQL1, PQL2 | PsbQ-like 2 | chr1:4839885-4840
1170	AT3G48110.1	2.54	t / c	|EDD1, EDD | glycine-tRNA ligases | chr3:1776
1491	AT1G55160.3	2.54	t / c	| | unknown protein; FUNCTIONS IN: molecular_
1573	AT1G35720.1	2.54	t / c	|ANNAT1, OXY5, ATOXY5 | annexin 1 | chr1:1322
59	AT1G61520.1 (+1)	2.53	t / c	|LHCA3 | photosystem I light harvesting compl
508	AT3G61820.1	2.53	t / c	| | Eukaryotic aspartyl protease family prote
574	AT5G62790.1	2.52	t / c	|DXR, PDE129 | 1-deoxy-D-xylulose 5-phosphate
1043	AT5G63420.1	2.52	t / c	|emb2746 | RNA-metabolising metallo-beta-lact
476	AT3G14940.1	2.51	t / c	|ATPPC3, PPC3 | phosphoenolpyruvate carboxyla
779	AT4G36250.1	2.50	t-unique	|ALDH3F1 | aldehyde dehydrogenase 3F1 | chr4:
1220	AT1G20440.1	2.50	t-unique	|COR47, RD17, AtCOR47 | cold-regulated 47 | c
1770	AT1G15930.1 (+1)	2.50	t-unique	| | Ribosomal protein L7Ae/L30e/S12e/Gadd45 f
122	ATCG00470.1	2.50	t / c	|ATPE | ATP synthase epsilon chain | chrC:522
248	AT1G11750.1	2.50	t / c	|CLPP6, NCLPP1, NCLPP6 | CLP protease proteol
465	AT4G36220.1	2.50	t / c	|FAH1, CYP84A1 | ferulic acid 5-hydroxylase 1
646	AT1G17745.2	2.50	t / c	|PGDH | D-3-phosphoglycerate dehydrogenase |
768	AT2G20760.1	2.50	t / c	| | Clathrin light chain protein | chr2:89432
873	AT1G30400.1 (+1)	2.50	t / c	|ATMRP1, EST1, ABCC1, ATABCC1, MRP1 | multidr
968	AT3G51890.1	2.50	t / c	| | Clathrin light chain protein | chr3:19249
1011	AT2G36810.1	2.50	t / c	| | ARM repeat superfamily protein | chr2:154
1090	AT2G05840.1	2.50	t / c	|PAA2 | 20S proteasome subunit PAA2 | chr2:22
1310	AT3G44330.1	2.50	t / c	| | INVOLVED IN: protein processing; LOCATED
1327	AT1G23080.3	2.50	t / c	|PIN7 | Auxin efflux carrier family protein |
1439	AT1G44446.1	2.50	t / c	|CH1, ATCAO, CAO | Pheophorbide a oxygenase f
1555	AT1G76180.1 (+1)	2.50	t / c	|ERD14 | Dehydrin family protein | chr1:28587

	Accession.Number	log2tc	label	Description
1640	AT2G44060.1 (+1)	2.50	t / c	| | Late embryogenesis abundant protein, grou
1712	AT4G30810.1	2.50	t / c	|scpl29 | serine carboxypeptidase-like 29 | c
1755	AT2G32060.1 (+2)	2.50	t / c	| | Ribosomal protein L7Ae/L30e/S12e/Gadd45 f
1792	AT5G58100.1	2.50	t / c	| | unknown protein; INVOLVED IN: pollen exin
1853	AT4G36480.1 (+1)	2.50	t / c	|ATLCB1, LCB1, EMB2779, FBR11 | long-chain ba
1888	AT3G61650.1	2.50	t / c	|TUBG1 | gamma-tubulin | chr3:22812601-228150
1899	AT4G17770.1	2.50	t / c	|ATTPS5, TPS5 | trehalose phosphatase/synthas
1927	AT1G50370.1	2.50	t / c	| | Calcineurin-like metallo-phosphoesterase
67	AT5G25980.2	2.49	t / c	|TGG2, BGLU37 | glucoside glucohydrolase 2 |
212	AT3G54050.1 (+1)	2.48	t / c	|HCEF1 | high cyclic electron flow 1 | chr3:2
222	AT1G62020.1	2.48	t / c	| | Coatomer, alpha subunit | chr1:22919814-2
487	AT1G49970.1	2.48	t / c	|CLPR1, NCLPP5, SVR2 | CLP protease proteolyt
765	AT5G20280.1	2.48	t / c	|ATSPS1F, SPS1F | sucrose phosphate synthase
329	AT1G58684.1 (+2)	2.47	t / c	| | Ribosomal protein S5 family protein | chr
407	AT2G38750.1	2.47	t / c	|ANNAT4 | annexin 4 | chr2:16196582-16198431
868	AT1G09780.1	2.47	t / c	| | Phosphoglycerate mutase, 2,3-bisphosphogl
1691	AT3G20390.1	2.47	t / c	| | endoribonuclease L-PSP family protein | c
1737	AT3G06580.1	2.47	t / c	|GAL1, GALK | Mevalonate/galactokinase family
1779	AT2G22125.1	2.47	t / c	|CSI1 | binding | chr2:9406793-9414223 FORWAR
100	AT3G13920.1	2.46	t / c	|EIF4A1, RH4, TIF4A1 | eukaryotic translation
115	ATCG00130.1	2.46	t / c	|ATPF | ATPase, F0 complex, subunit B/B’, bac
795	AT4G24820.1 (+1)	2.46	t / c	| | 26S proteasome, regulatory subunit Rpn7;P
157	ATCG00800.1	2.45	t / c	| | structural constituent of ribosome | chrC
406	AT1G62750.1	2.45	t / c	|ATSCO1, ATSCO1/CPEF-G, SCO1 | Translation el
1158	AT5G66470.1	2.45	t / c	| | RNA binding;GTP binding | chr5:26541986-2
143	AT2G21390.1	2.44	t / c	| | Coatomer, alpha subunit | chr2:9152428-91
1227	AT4G16130.1	2.44	t / c	|ARA1, ISA1, ATISA1 | arabinose kinase | chr4
96	AT1G54270.1	2.43	t / c	|EIF4A-2 | eif4a-2 | chr1:20260495-20262018 F
182	AT3G08530.1	2.43	t / c	| | Clathrin, heavy chain | chr3:2587171-2595
422	AT2G10940.1 (+1)	2.43	t / c	| | Bifunctional inhibitor/lipid-transfer pro

	Accession.Number	log2tc	label	Description
724	AT1G15690.1	2.43	t / c	|AVP1, ATAVP3, AVP-3, AtVHP1;1 | Inorganic H
1063	AT1G02560.1	2.43	t / c	|CLPP5, NCLPP5, NCLPP1 | nuclear encoded CLP
1392	AT4G13930.1	2.43	t / c	|SHM4 | serine hydroxymethyltransferase 4 | c
161	AT3G44310.1 (+1)	2.42	t / c	|NIT1, ATNIT1, NITI | nitrilase 1 | chr3:1598
281	AT3G22890.1	2.42	t / c	|APS1 | ATP sulfurylase 1 | chr3:8112837-8114
537	AT4G30920.1	2.42	t / c	| | Cytosol aminopeptidase family protein | c
552	AT3G49720.1 (+1)	2.42	t / c	| | unknown protein; FUNCTIONS IN: molecular_
706	AT2G20920.1	2.42	t / c	| | Protein of unknown function (DUF3353) | c
726	AT2G44530.2	2.42	t / c	| | Phosphoribosyltransferase family protein
1001	AT3G59110.1	2.42	t / c	| | Protein kinase superfamily protein | chr3
1709	AT5G67385.1	2.42	t / c	| | Phototropic-responsive NPH3 family protei
1800	AT1G29790.1 (+1)	2.42	t / c	| | S-adenosyl-L-methionine-dependent methylt
1826	AT5G26830.1	2.42	t / c	| | Threonyl-tRNA synthetase | chr5:9437351-9
1829	ATCG00170.1	2.42	t / c	|RPOC2 | DNA-directed RNA polymerase family p
1965	AT4G24220.1 (+1)	2.42	t / c	|VEP1, AWI31 | NAD(P)-binding Rossmann-fold s
2030	AT3G09090.1 (+1)	2.42	t / c	|DEX1 | defective in exine formation protein
1316	AT3G42170.1	2.42	t-unique	| | BED zinc finger ;hAT family dimerisation
34	AT2G07698.1	2.41	t / c	| | ATPase, F1 complex, alpha subunit protein
253	AT1G02500.1 (+1)	2.40	t / c	|SAM1, SAM-1, MAT1, AtSAM1 | S-adenosylmethio
276	AT2G31610.1	2.40	t / c	| | Ribosomal protein S3 family protein | chr
1625	AT5G11670.1	2.40	t / c	|ATNADP-ME2, NADP-ME2 | NADP-malic enzyme 2 |
1675	AT4G29840.1	2.40	t / c	|MTO2, TS | Pyridoxal-5’-phosphate-dependent
1715	AT5G59880.1	2.40	t / c	|ADF3 | actin depolymerizing factor 3 | chr5:
1818	AT5G47200.1	2.40	t / c	|ATRABD2B, ATRAB1A, RAB1A | RAB GTPase homolo
37	AT4G04640.1	2.39	t / c	|ATPC1 | ATPase, F1 complex, gamma subunit pr
213	AT3G29360.1 (+1)	2.39	t / c	| | UDP-glucose 6-dehydrogenase family protei
592	AT1G71810.1	2.39	t / c	| | Protein kinase superfamily protein | chr1
632	AT1G32500.1	2.39	t / c	|ATNAP6, NAP6 | non-intrinsic ABC protein 6 |
737	AT1G74730.1	2.39	t / c	| | Protein of unknown function (DUF1118) | c
1581	AT5G15090.1 (+1)	2.39	t / c	|VDAC3, ATVDAC3 | voltage dependent anion cha

	Accession.Number	log2tc	label	Description
47	AT1G15820.1	2.38	t / c	|LHCB6, CP24 | light harvesting complex photo
259	AT1G48030.1 (+1)	2.38	t / c	|mtLPD1 | mitochondrial lipoamide dehydrogena
1517	AT5G25757.1 (+1)	2.38	t / c	| | RNA polymerase I-associated factor PAF67
1633	AT1G29150.1	2.38	t / c	|ATS9, RPN6 | non-ATPase subunit 9 | chr1:101
347	AT3G06510.2	2.37	t / c	|SFR2 | Glycosyl hydrolase superfamily protei
460	AT3G23990.1	2.37	t / c	|HSP60, HSP60-3B | heat shock protein 60 | ch
764	AT1G59900.1	2.37	t / c	|AT-E1 ALPHA, E1 ALPHA | pyruvate dehydrogena
1598	AT1G63940.4	2.37	t / c	|MDAR6 | monodehydroascorbate reductase 6 | c
258	AT4G32260.1	2.36	t / c	| | ATPase, F0 complex, subunit B/B’, bacteri
297	AT3G06650.1	2.36	t / c	|ACLB-1 | ATP-citrate lyase B-1 | chr3:207924
377	AT3G03960.1	2.36	t / c	| | TCP-1/cpn60 chaperonin family protein | c
599	AT5G51070.1	2.36	t / c	|ERD1, CLPD, SAG15 | Clp ATPase | chr5:207644
1606	ATCG00420.1	2.36	t / c	|NDHJ | NADH dehydrogenase subunit J | chrC:4
256	AT3G03780.1 (+2)	2.35	t / c	|ATMS2, MS2 | methionine synthase 2 | chr3:95
428	AT1G55670.1	2.35	t / c	|PSAG | photosystem I subunit G | chr1:208028
438	AT3G58610.1 (+2)	2.35	t / c	| | ketol-acid reductoisomerase | chr3:216715
155	AT3G11130.1	2.34	t / c	| | Clathrin, heavy chain | chr3:3482575-3491
446	AT1G61790.1	2.32	t-unique	| | Oligosaccharyltransferase complex/magnesi
1789	AT5G20090.1 (+1)	2.32	t-unique	| | Uncharacterised protein family (UPF0041)
339	AT2G34590.1	2.32	t / c	| | Transketolase family protein | chr2:14568
597	AT3G52880.1	2.32	t / c	|ATMDAR1, MDAR1 | monodehydroascorbate reduct
673	AT1G01080.1	2.32	t / c	| | RNA-binding (RRM/RBD/RNP motifs) family p
723	AT2G36390.1	2.32	t / c	|SBE2.1, BE3 | starch branching enzyme 2.1 |
755	AT3G13330.1	2.32	t / c	|PA200 | proteasome activating protein 200 |
761	AT1G79870.1	2.32	t / c	| | D-isomer specific 2-hydroxyacid dehydroge
773	AT5G67030.1	2.32	t / c	|ABA1, LOS6, NPQ2, ATABA1, ZEP, IBS3, ATZEP |
808	AT1G63970.1 (+1)	2.32	t / c	|ISPF, MECPS | isoprenoid F | chr1:23738923-2
981	AT3G10690.1	2.32	t / c	|GYRA | DNA GYRASE A | chr3:3339612-3346243 R
989	AT3G15730.1	2.32	t / c	|PLDALPHA1, PLD | phospholipase D alpha 1 | c
1026	AT5G10690.1	2.32	t / c	| | pentatricopeptide (PPR) repeat-containing

	Accession.Number	log2tc	label	Description
1050	AT3G01300.1	2.32	t / c	| | Protein kinase superfamily protein | chr3
1057	AT2G36850.1	2.32	t / c	|ATGSL08, GSL8, GSL08, ATGSL8, CHOR | glucan
1073	AT3G51420.1	2.32	t / c	|SSL4, ATSSL4 | strictosidine synthase-like 4
1095	AT2G01350.1	2.32	t / c	|QPT | quinolinate phoshoribosyltransferase |
1125	AT5G26360.1	2.32	t / c	| | TCP-1/cpn60 chaperonin family protein | c
1157	AT1G73600.2	2.32	t / c	| | S-adenosyl-L-methionine-dependent methylt
1180	AT3G52180.1	2.32	t / c	|ATPTPKIS1, DSP4, SEX4, ATSEX4 | dual specifi
1239	AT1G21630.1	2.32	t / c	| | Calcium-binding EF hand family protein |
1342	AT3G54470.1	2.32	t / c	| | uridine 5'-monophosphate synthase / UMP s
1373	AT3G25690.1 (+1)	2.32	t / c	|CHUP1 | Hydroxyproline-rich glycoprotein fam
1406	AT1G79920.1	2.32	t / c	| | Heat shock protein 70 (Hsp 70) family pro
1644	AT2G21870.1	2.32	t / c	|MGP1 | copper ion binding;cobalt ion binding
1677	AT1G78830.1	2.32	t / c	| | Curculin-like (mannose-binding) lectin fa
1701	AT3G23300.1	2.32	t / c	| | S-adenosyl-L-methionine-dependent methylt
1714	AT5G47930.1	2.32	t / c	| | Zinc-binding ribosomal protein family pro
1782	AT3G05910.1	2.32	t / c	| | Pectinacetylesterase family protein | chr
1791	AT2G43560.1	2.32	t / c	| | FKBP-like peptidyl-prolyl cis-trans isome
1816	AT1G13110.1	2.32	t / c	|CYP71B7 | cytochrome P450, family 71 subfami
1831	AT4G01690.1	2.32	t / c	|PPOX, HEMG1, PPO1 | Flavin containing amine
1898	AT1G62180.1	2.32	t / c	|APR2, APSR, PRH43, PRH, ATAPR2 | 5'adenylylp
2000	AT5G14120.1	2.32	t / c	| | Major facilitator superfamily protein | c
189	AT1G72370.1 (+1)	2.30	t / c	|P40, AP40, RP40, RPSAA | 40s ribosomal prote
119	AT3G04840.1	2.29	t / c	| | Ribosomal protein S3Ae | chr3:1329751-133
604	AT3G56150.1 (+1)	2.29	t / c	|EIF3C, ATEIF3C-1, EIF3C-1, ATTIF3C1, TIF3C1
238	AT3G47520.1	2.28	t / c	|MDH | malate dehydrogenase | chr3:17513657-1
594	AT2G05990.1 (+1)	2.28	t / c	|MOD1, ENR1 | NAD(P)-binding Rossmann-fold su
699	AT1G16720.1	2.28	t / c	|HCF173 | high chlorophyll fluorescence pheno
730	AT2G28900.1	2.28	t / c	|OEP16, ATOEP16-L, ATOEP16-1, OEP16-1 | outer
60	AT4G10340.1	2.27	t / c	|LHCB5 | light harvesting complex of photosys
357	AT1G02280.1 (+1)	2.27	t / c	|TOC33, ATTOC33, PPI1 | translocon at the out

	Accession.Number	log2tc	label	Description
450	AT4G08870.1	2.27	t / c	| | Arginase/deacetylase superfamily protein
418	AT3G48730.1	2.26	t / c	|GSA2 | glutamate-1-semialdehyde 2,1-aminomut
872	AT5G50950.2	2.26	t / c	|FUM2 | FUMARASE 2 | chr5:20729687-20733636 F
1643	AT2G40100.1	2.26	t / c	|LHCB4.3 | light harvesting complex photosyst
86	AT1G78900.1 (+1)	2.25	t / c	|VHA-A | vacuolar ATP synthase subunit A | ch
573	AT3G06050.1	2.25	t / c	|PRXIIF, ATPRXIIF | peroxiredoxin IIF | chr3:
1531	AT4G01850.1 (+1)	2.25	t / c	|SAM-2, MAT2, SAM2, AtSAM2 | S-adenosylmethio
80	AT1G31330.1	2.23	t / c	|PSAF | photosystem I subunit F | chr1:112150
132	AT5G17920.1 (+1)	2.23	t / c	|ATCIMS, ATMETS, ATMS1 | Cobalamin-independen
1952	AT5G59420.1	2.22	t-unique	|ORP3C | OSBP(oxysterol binding protein)-rela
11	ATCG00120.1	2.22	t / c	|ATPA | ATP synthase subunit alpha | chrC:993
647	AT4G24570.1	2.22	t / c	|DIC2 | dicarboxylate carrier 2 | chr4:126865
668	AT1G67730.1	2.22	t / c	|YBR159, KCR1, ATKCR1 | beta-ketoacyl reducta
920	AT2G26910.1	2.22	t / c	|PDR4, ATPDR4 | pleiotropic drug resistance 4
1022	AT4G03430.1	2.22	t / c	|STA1, EMB2770 | pre-mRNA splicing factor-rel
1210	AT1G65220.1	2.22	t / c	| | ARM repeat superfamily protein | chr1:242
1283	AT1G47550.1	2.22	t / c	|SEC3A | exocyst complex component sec3A | ch
1657	AT5G15650.1	2.22	t / c	|RGP2, ATRGP2 | reversibly glycosylated polyp
1668	AT1G14610.1	2.22	t / c	|TWN2, VALRS | valyl-tRNA synthetase / valine
1901	AT3G57280.1	2.22	t / c	| | Transmembrane proteins 14C | chr3:2119396
1923	AT1G14670.1	2.22	t / c	| | Endomembrane protein 70 protein family |
1959	AT4G30610.1	2.22	t / c	|BRS1, SCPL24 | alpha/beta-Hydrolases superfa
2017	AT4G36390.1	2.22	t / c	| | Methylthiotransferase | chr4:17194746-171
1305	AT1G12000.1	2.21	t / c	| | Phosphofructokinase family protein | chr1
735	AT3G56910.1	2.20	t / c	|PSRP5 | plastid-specific 50S ribosomal prote
784	AT3G17020.1	2.20	t / c	| | Adenine nucleotide alpha hydrolases-like
918	AT3G10670.1	2.20	t / c	|ATNAP7, NAP7 | non-intrinsic ABC protein 7 |
1187	AT2G07707.1 (+1)	2.20	t / c	| | Plant mitochondrial ATPase, F0 complex, s
1609	AT1G30230.1 (+1)	2.20	t / c	| | Glutathione S-transferase, C-terminal-lik
32	AT3G26650.1	2.18	t / c	|GAPA, GAPA-1 | glyceraldehyde 3-phosphate de

	Accession.Number	log2tc	label	Description
871	AT3G59970.3	2.18	t / c	|MTHFR1 | methylenetetrahydrofolate reductase
1205	AT2G28190.1	2.18	t / c	|CSD2, CZSOD2 | copper/zinc superoxide dismut
29	AT1G12900.1	2.17	t / c	|GAPA-2 | glyceraldehyde 3-phosphate dehydrog
33	AT3G14420.1 (+1)	2.17	t / c	| | Aldolase-type TIM barrel family protein |
447	AT3G09790.1	2.17	t / c	|UBQ8 | ubiquitin 8 | chr3:3004111-3006006 RE
144	AT3G46780.1	2.15	t / c	|PTAC16 | plastid transcriptionally active 16
551	AT3G09820.2	2.15	t / c	|ADK1 | adenosine kinase 1 | chr3:3012645-301
717	AT3G25860.1	2.15	t / c	|LTA2, PLE2 | 2-oxoacid dehydrogenases acyltr
960	AT5G19620.1	2.15	t / c	|EMB213, OEP80, ATOEP80, TOC75 | outer envelo
1032	AT4G11150.1	2.15	t / c	|TUF, emb2448, TUFF, VHA-E1 | vacuolar ATP sy
1278	AT3G50930.1	2.15	t / c	|BCS1 | cytochrome BC1 synthesis | chr3:18929
1329	AT1G07250.1	2.15	t / c	|UGT71C4 | UDP-glucosyl transferase 71C4 | ch
1824	AT5G35590.1	2.15	t / c	|PAA1 | proteasome alpha subunit A1 | chr5:13
507	AT1G18080.1	2.14	t / c	|ATARCA, RACK1A_AT, RACK1A | Transducin/WD40
267	AT5G02870.1	2.13	t / c	| | Ribosomal protein L4/L1 family | chr5:657
429	AT5G55190.1	2.13	t / c	|RAN3, ATRAN3 | RAN GTPase 3 | chr5:22392285-
448	AT2G39800.3	2.13	t / c	|P5CS1 | delta1-pyrroline-5-carboxylate synth
1651	AT4G38740.1	2.13	t / c	|ROC1 | rotamase CYP 1 | chr4:18083620-180841
1736	AT2G48070.1 (+1)	2.13	t / c	|RPH1 | resistance to phytophthora 1 | chr2:1
23	AT1G42970.1	2.12	t / c	|GAPB | glyceraldehyde-3-phosphate dehydrogen
484	AT4G27680.1	2.12	t / c	| | P-loop containing nucleoside triphosphate
567	AT5G16660.2	2.12	t / c	| | unknown protein; FUNCTIONS IN: molecular_
707	AT2G18330.1	2.12	t / c	| | AAA-type ATPase family protein | chr2:796
741	AT1G24510.1	2.12	t / c	| | TCP-1/cpn60 chaperonin family protein | c
1003	AT1G31800.1	2.12	t / c	|CYP97A3, LUT5 | cytochrome P450, family 97,
1107	AT5G61810.1	2.12	t / c	| | Mitochondrial substrate carrier family pr
1156	AT4G01330.2	2.12	t / c	| | Protein kinase superfamily protein | chr4
1268	AT4G32250.1 (+2)	2.12	t / c	| | Protein kinase superfamily protein | chr4
1352	AT5G23300.1	2.12	t / c	|PYRD | pyrimidine d | chr5:7847792-7850243 R
1681	AT5G04590.1	2.12	t / c	|SIR | sulfite reductase | chr5:1319404-13222

	Accession.Number	log2tc	label	Description
1696	AT5G03320.1	2.12	t / c	| | Protein kinase superfamily protein | chr5
1767	AT3G10060.1	2.12	t / c	| | FKBP-like peptidyl-prolyl cis-trans isome
1777	AT5G45620.1	2.12	t / c	| | Proteasome component (PCI) domain protein
1828	AT4G32300.1	2.12	t / c	|SD2-5 | S-domain-2 5 | chr4:15599970-1560243
1843	AT1G64900.1	2.12	t / c	|CYP89A2, CYP89 | cytochrome P450, family 89,
1877	AT2G40060.1	2.12	t / c	| | Clathrin light chain protein | chr2:16726
1906	AT5G63510.2	2.12	t / c	|GAMMA CAL1 | gamma carbonic anhydrase like 1
1929	AT4G00026.1	2.12	t / c	| | FUNCTIONS IN: molecular_function unknown;
1958	AT5G59250.1	2.12	t / c	| | Major facilitator superfamily protein | c
1996	AT2G28430.1	2.12	t / c	| | unknown protein; Has 28 Blast hits to 28
1120	AT3G44620.1 (+1)	2.12	t-unique	| | protein tyrosine phosphatases;protein tyr
1492	AT1G22280.1	2.12	t-unique	|PAPP2C | phytochrome-associated protein phos
1935	AT5G58490.1	2.12	t-unique	| | NAD(P)-binding Rossmann-fold superfamily
1961	AT3G03100.1	2.12	t-unique	| | NADH:ubiquinone oxidoreductase, 17.2kDa s
2106	AT3G25070.1	2.12	t-unique	|RIN4 | RPM1 interacting protein 4 | chr3:913
261	AT3G63140.1	2.11	t / c	|CSP41A | chloroplast stem-loop binding prote
300	AT1G01090.1	2.11	t / c	|PDH-E1 ALPHA | pyruvate dehydrogenase E1 alp
241	AT4G02930.1	2.10	t / c	| | GTP binding Elongation factor Tu family p
299	AT4G34350.1	2.10	t / c	|CLB6, ISPH, HDR | 4-hydroxy-3-methylbut-2-en
864	AT5G61790.1	2.10	t / c	|CNX1, ATCNX1 | calnexin 1 | chr5:24827394-24
76	AT5G14740.2	2.09	t / c	|CA2, CA18, BETA CA2 | carbonic anhydrase 2 |
750	AT3G48000.1	2.09	t / c	|ALDH2B4, ALDH2, ALDH2A | aldehyde dehydrogen
1610	AT3G52300.1	2.09	t / c	|ATPQ | ATP synthase D chain, mitochondrial |
398	AT5G47210.1	2.08	t / c	| | Hyaluronan / mRNA binding family | chr5:1
25	AT4G20360.1	2.07	t / c	|ATRAB8D, ATRABE1B, RABE1b | RAB GTPase homol
516	AT5G65010.2	2.07	t / c	|ASN2 | asparagine synthetase 2 | chr5:259692
83	AT4G13940.1	2.06	t / c	|HOG1, EMB1395, SAHH1, MEE58, ATSAHH1 | S-ade
367	AT2G36530.1	2.06	t / c	|LOS2, ENO2 | Enolase | chr2:15321081-1532378
549	AT1G01300.1	2.06	t / c	| | Eukaryotic aspartyl protease family prote
844	AT2G20420.1	2.06	t / c	| | ATP citrate lyase (ACL) family protein |

	Accession.Number	log2tc	label	Description
1080	AT1G49750.1	2.06	t / c	| | Leucine-rich repeat (LRR) family protein
1361	AT1G16880.1	2.06	t / c	| | uridylyltransferase-related | chr1:577379
1603	AT1G74040.1	2.06	t / c	|IMS1, MAML-3, IPMS2 | 2-isopropylmalate synt
1659	AT2G35490.1	2.06	t / c	| | Plastid-lipid associated protein PAP / fi
1757	AT4G39080.1	2.06	t / c	|VHA-A3 | vacuolar proton ATPase A3 | chr4:18
1806	AT1G74910.1 (+1)	2.06	t / c	| | ADP-glucose pyrophosphorylase family prot
1889	AT5G11450.1	2.06	t / c	| | Mog1/PsbP/DUF1795-like photosystem II rea
1974	AT3G49560.1	2.06	t / c	| | Mitochondrial import inner membrane trans
72	AT2G33800.1	2.05	t / c	| | Ribosomal protein S5 family protein | chr
521	AT3G25520.1	2.05	t / c	|ATL5, PGY3, OLI5, RPL5A | ribosomal protein
62	AT2G34430.1	2.03	t / c	|LHB1B1, LHCB1.4 | light-harvesting chlorophy
172	AT3G01500.2	2.03	t / c	|CA1 | carbonic anhydrase 1 | chr3:194853-197
466	AT1G66200.1	2.03	t / c	|ATGSR2, GSR2, GLN1;2 | glutamine synthase cl
509	AT1G52230.1	2.03	t / c	|PSAH2, PSAH-2, PSI-H | photosystem I subunit
543	AT4G34870.1	2.03	t / c	|ROC5, ATCYP1 | rotamase cyclophilin 5 | chr4
553	AT5G07030.1	2.03	t / c	| | Eukaryotic aspartyl protease family prote
676	AT4G17040.1	2.03	t / c	|CLPR4 | CLP protease R subunit 4 | chr4:9586
202	AT3G09630.1	2.02	t / c	| | Ribosomal protein L4/L1 family | chr3:295
432	AT1G69740.1 (+1)	2.02	t / c	|HEMB1 | Aldolase superfamily protein | chr1:
461	AT1G30630.1	2.02	t / c	| | Coatomer epsilon subunit | chr1:10858546-
623	AT1G79550.1 (+1)	2.02	t / c	|PGK | phosphoglycerate kinase | chr1:2992434
917	AT5G58140.3	2.02	t / c	|PHOT2, NPL1 | phototropin 2 | chr5:23524771-
933	AT4G24830.1	2.02	t / c	| | arginosuccinate synthase family | chr4:12
1035	ATCG01130.1	2.02	t / c	|YCF1.2 | Ycf1 protein | chrC:123884-129244 R
1730	AT4G24750.1	2.02	t / c	| | Rhodanese/Cell cycle control phosphatase
384	AT5G54270.1	2.01	t / c	|LHCB3, LHCB3*1 | light-harvesting chlorophyl
642	AT3G09830.1 (+1)	2.00	t / c	| | Protein kinase superfamily protein | chr3
728	AT2G31040.1	2.00	t-unique	| | ATP synthase protein I -related | chr2:13
903	ATCG00190.1	2.00	t / c	|RPOB | RNA polymerase subunit beta | chrC:23
923	AT4G28710.1	2.00	t / c	|XIH, ATXIH | Myosin family protein with Dil

	Accession.Number	log2tc	label	Description
955	AT3G48720.1	2.00	t / c	| | HXXXD-type acyl-transferase family protei
1015	AT2G35800.1	2.00	t / c	| | mitochondrial substrate carrier family pr
1045	AT5G67560.1	2.00	t / c	|ATARLA1D, ARLA1D | ADP-ribosylation factor-l
1331	AT3G17970.1	2.00	t / c	|atToc64-III, TOC64-III | translocon at the o
1335	AT4G24770.1	2.00	t / c	|RBP31, ATRBP31, CP31, ATRBP33 | 31-kDa RNA b
1353	AT3G07700.1 (+2)	2.00	t / c	| | Protein kinase superfamily protein | chr3
1371	AT5G11040.1	2.00	t / c	|TRS120, AtTRS120 | TRS120 | chr5:3495332-350
1415	AT4G32400.1	2.00	t-unique	|EMB104, SHS1, EMB42, ATBT1 | Mitochondrial s
1426	AT2G25870.1	2.00	t / c	| | haloacid dehalogenase-like hydrolase fami
1446	AT5G64860.1	2.00	t / c	|DPE1 | disproportionating enzyme | chr5:2592
1717	AT2G42220.1	2.00	t / c	| | Rhodanese/Cell cycle control phosphatase
1787	AT5G66140.1	2.00	t / c	|PAD2 | proteasome alpha subunit D2 | chr5:26
1823	AT1G56050.1	2.00	t / c	| | GTP-binding protein-related | chr1:209637
1832	AT4G16180.2	2.00	t / c	| | unknown protein; FUNCTIONS IN: molecular_
1867	AT3G62360.1	2.00	t / c	| | Carbohydrate-binding-like fold | chr3:230
1874	AT5G61910.4	2.00	t / c	| | DCD (Development and Cell Death) domain p
1894	AT5G23140.1	2.00	t / c	|CLPP2, NCLPP7 | nuclear-encoded CLP protease
1942	AT4G24550.2	2.00	t / c	| | Clathrin adaptor complexes medium subunit
1993	AT3G15000.1	2.00	t-unique	| | cobalt ion binding | chr3:5050321-5052121
2035	AT3G24530.1	2.00	t / c	| | AAA-type ATPase family protein / ankyrin
2038	AT3G48140.1	2.00	t / c	| | B12D protein | chr3:17778471-17779299 FOR
2089	AT3G04210.1	2.00	t / c	| | Disease resistance protein (TIR-NBS class
2102	AT4G04570.1	2.00	t / c	|CRK40 | cysteine-rich RLK (RECEPTOR-like pro
2172	AT3G05000.1	2.00	t / c	| | Transport protein particle (TRAPP) compon
572	AT2G34470.1	1.87	t-unique	|UREG, PSKF109 | urease accessory protein G |
621	AT5G06870.1	1.87	t-unique	|PGIP2, ATPGIP2 | polygalacturonase inhibitin
2098	AT5G04530.1	1.87	t-unique	|KCS19 | 3-ketoacyl-CoA synthase 19 | chr5:12
606	AT5G11480.1	1.74	t-unique	| | P-loop containing nucleoside triphosphate
710	AT1G74640.1	1.74	t-unique	| | alpha/beta-Hydrolases superfamily protein
996	AT3G53700.1	1.74	t-unique	|MEE40 | Pentatricopeptide repeat (PPR) super

	Accession.Number	log2tc	label	Description
1104	AT4G30580.1	1.74	t-unique	|ATS2, EMB1995, LPAT1 | Phospholipid/glycerol
1443	AT2G19520.1	1.74	t-unique	|FVE, ACG1, MSI4, NFC4, NFC04, ATMSI4 | Trans
1481	AT2G41040.1	1.74	t-unique	| | S-adenosyl-L-methionine-dependent methylt
1663	AT1G30440.1	1.74	t-unique	| | Phototropic-responsive NPH3 family protei
1725	AT1G04430.1 (+1)	1.74	t-unique	| | S-adenosyl-L-methionine-dependent methylt
2145	AT3G24570.1	1.74	t-unique	| | Peroxisomal membrane 22 kDa (Mpv17/PMP22)
2179	AT5G10780.1 (+1)	1.74	t-unique	| | CONTAINS InterPro DOMAIN/s: Uncharacteris
1410	AT1G32220.1	1.58	t-unique	| | NAD(P)-binding Rossmann-fold superfamily
1488	AT1G74690.1	1.58	t-unique	|IQD31 | IQ-domain 31 | chr1:28061498-2806392
1521	AT4G27070.1	1.58	t-unique	|TSB2 | tryptophan synthase beta-subunit 2 |
2029	ATCG00740.1	1.58	t-unique	|RPOA | RNA polymerase subunit alpha | chrC:7
2044	AT5G51110.1 (+1)	1.58	t-unique	| | Transcriptional coactivator/pterin dehydr
2225	AT3G62550.1	1.58	t-unique	| | Adenine nucleotide alpha hydrolases-like
2284	AT3G57260.1	1.58	t-unique	|BGL2, PR2, BG2, PR-2 | beta-1,3-glucanase 2
738	AT5G03455.1	1.42	t-unique	|CDC25, ARATH;CDC25, ACR2 | Rhodanese/Cell cy
1225	AT5G56760.1	1.42	t-unique	|ATSERAT1;1, SAT5, SAT-52, SERAT1;1 | serine
1318	AT3G54760.1 (+1)	1.42	t-unique	| | dentin sialophosphoprotein-related | chr3
1383	AT5G54180.1	1.42	t-unique	|PTAC15 | plastid transcriptionally active 15
2094	AT2G33120.1	1.42	t-unique	|SAR1, VAMP722, ATVAMP722 | synaptobrevin-rel
2319	AT5G07190.2	1.42	t-unique	|ATS3 | seed gene 3 | chr5:2237783-2238488 FO
1370	AT1G05350.1	1.22	t-unique	| | NAD(P)-binding Rossmann-fold superfamily
2113	AT3G15680.1	1.22	t-unique	| | Ran BP2/NZF zinc finger-like superfamily
2162	AT3G47960.1	1.22	t-unique	| | Major facilitator superfamily protein | c
2199	AT1G75130.1	1.22	t-unique	|CYP721A1 | cytochrome P450, family 721, subf
2282	AT2G27290.1	1.22	t-unique	| | Protein of unknown function (DUF1279) | c
1332	AT5G11840.1	1.00	t-unique	| | Protein of unknown function (DUF1230) | c
1756	AT2G20530.1 (+1)	1.00	t-unique	|ATPHB6, PHB6 | prohibitin 6 | chr2:8842300-8
1842	AT1G55480.1	1.00	t-unique	|ZKT | protein containing PDZ domain, a K-box
1955	AT4G26070.2 (+1)	1.00	t-unique	|MEK1, NMAPKK, ATMEK1, MKK1 | MAP kinase/ ERK
2103	AT5G63620.1	1.00	t-unique	| | GroES-like zinc-binding alcohol dehydroge

	Accession.Number	log2tc	label	Description
2114	AT1G42960.1	1.00	t-unique	| | expressed protein localized to the inner
1108	AT1G10430.1	0.74	t-unique	|PP2A-2 | protein phosphatase 2A-2 | chr1:342
1182	AT5G54110.1	0.74	t-unique	|ATMAMI, MAMI | membrane-associated mannitol-
1202	AT5G47890.1	0.74	t-unique	| | NADH-ubiquinone oxidoreductase B8 subunit
1417	AT1G64090.1	0.74	t-unique	|RTNLB3 | Reticulan like protein B3 | chr1:23
1807	AT1G67280.1	0.74	t-unique	| | Glyoxalase/Bleomycin resistance protein/D
1881	AT3G43980.1 (+2)	0.74	t-unique	| | Ribosomal protein S14p/S29e family protei
2039	AT5G48230.2	0.74	t-unique	|EMB1276, ACAT2 | acetoacetyl-CoA thiolase 2
2093	AT5G48810.1	0.74	t-unique	|ATB5-B, B5 #3, ATCB5-D, CB5-D | cytochrome B
2186	AT4G35860.1	0.74	t-unique	|ATRABB1B, ATGB2, ATRAB2C, GB2 | GTP-binding
2268	AT1G16890.2	0.74	t-unique	|UBC36, UBC13B | ubiquitin-conjugating enzyme
1402	AT2G20230.1	0.42	t-unique	| | Tetraspanin family protein | chr2:8725762
2107	AT4G35230.1	0.42	t-unique	|BSK1 | BR-signaling kinase 1 | chr4:16755325
2185	AT3G56140.1	0.42	t-unique	| | Protein of unknown function (DUF399 and D
1919	AT1G66150.1	0.00	t-unique	|TMK1 | transmembrane kinase 1 | chr1:2463150
2063	AT5G67590.1	0.00	t-unique	|FRO1 | NADH-ubiquinone oxidoreductase-relate
1786	AT5G02240.1	-1.38	c-unique	| | NAD(P)-binding Rossmann-fold superfamily
183	AT1G28380.1	-8.49	t / c	|NSL1 | MAC/Perforin domain-containing protei

===================== [1] "Pair No.: 2" [1] "CPK5 (m22) /// NSL1, PM-GFP (m22)" =====================

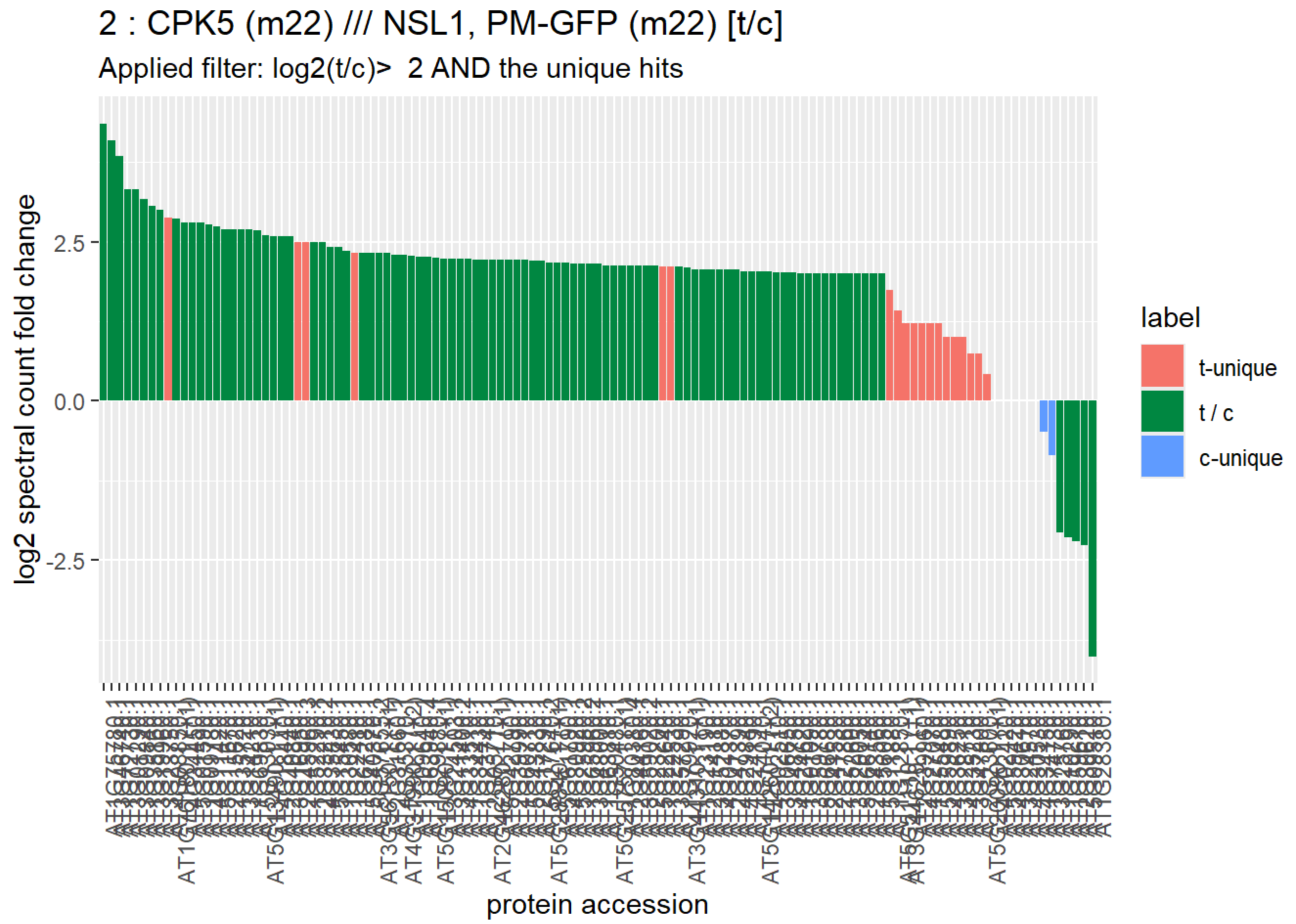

	Accession.Number	log2tc	label	Description
1527	AT1G75780.1	4.36	t / c	|TUB1 | tubulin beta-1 chain | chr1:28451378-
1427	AT3G46740.1	4.09	t / c	|TOC75-III, MAR1 | translocon at the outer en
1528	AT1G72730.1	3.85	t / c	| | DEA(D/H)-box RNA helicase family protein
511	AT3G01290.1	3.32	t / c	| | SPFH/Band 7/PHB domain-containing membran
984	AT3G09840.1	3.32	t / c	|CDC48, ATCDC48, CDC48A | cell division cycle
1559	AT1G35160.1	3.17	t / c	|GRF4, 14-3-3PHI, GF14 PHI | GF14 protein phi
780	AT3G19960.1	3.06	t / c	|ATM1 | myosin 1 | chr3:6949787-6956736 FORWA
1144	AT3G23750.1	3.00	t / c	| | Leucine-rich repeat protein kinase family
1056	AT4G08850.1	2.87	t-unique	| | Leucine-rich repeat receptor-like protein
1555	AT1G76180.1 (+1)	2.87	t / c	|ERD14 | Dehydrin family protein | chr1:28587
430	AT4G34450.1	2.81	t / c	| | coatomer gamma-2 subunit, putative / gamm
1558	AT5G01590.1	2.81	t / c	| | unknown protein; FUNCTIONS IN: molecular_
868	AT1G09780.1	2.80	t / c	| | Phosphoglycerate mutase, 2,3-bisphosphogl

	Accession.Number	log2tc	label	Description
1159	AT4G11420.1	2.77	t / c	|EIF3A, ATEIF3A-1, EIF3A-1, ATTIF3A1, TIF3A1
1625	AT5G11670.1	2.74	t / c	|ATNADP-ME2, NADP-ME2 | NADP-malic enzyme 2 |
348	AT2G15620.1	2.70	t / c	|NIR1, NIR, ATHNIR | nitrite reductase 1 | ch
1573	AT1G35720.1	2.70	t / c	|ANNAT1, OXY5, ATOXY5 | annexin 1 | chr1:1322
124	AT3G14210.1	2.69	t / c	|ESM1 | epithiospecifier modifier 1 | chr3:47
885	AT1G69830.1	2.69	t / c	|ATAMY3, AMY3 | alpha-amylase-like 3 | chr1:2
197	AT5G13030.1	2.68	t / c	| | unknown protein; FUNCTIONS IN: molecular_
75	AT5G13490.1 (+1)	2.61	t / c	|AAC2 | ADP/ATP carrier 2 | chr5:4336034-4337
828	AT4G18440.1	2.58	t / c	| | L-Aspartase-like family protein | chr4:10
1112	AT2G40840.1	2.58	t / c	|DPE2 | disproportionating enzyme 2 | chr2:17
1223	AT3G16950.1	2.58	t / c	|LPD1, ptlpd1 | lipoamide dehydrogenase 1 | c
385	AT2G45960.3	2.50	t-unique	|PIP1B, TMP-A, ATHH2, PIP1;2 | plasma membran
1582	AT1G52290.1	2.50	t-unique	| | Protein kinase superfamily protein | chr1
327	AT2G33210.2	2.50	t / c	|HSP60-2 | heat shock protein 60-2 | chr2:140
1290	AT4G35830.1	2.50	t / c	|ACO1 | aconitase 1 | chr4:16973007-16977949
1479	AT5G15450.1	2.42	t / c	|APG6, CLPB3, CLPB-P | casein lytic proteinas
1520	AT1G30580.1	2.42	t / c	| | GTP binding | chr1:10831953-10835454 REVE
181	AT1G22410.1	2.36	t / c	| | Class-II DAHP synthetase family protein |
1807	AT1G67280.1	2.32	t-unique	| | Glyoxalase/Bleomycin resistance protein/D
336	AT5G40370.1	2.32	t / c	| | Glutaredoxin family protein | chr5:161478
585	AT5G57655.2	2.32	t / c	| | xylose isomerase family protein | chr5:23
604	AT3G56150.1 (+1)	2.32	t / c	|EIF3C, ATEIF3C-1, EIF3C-1, ATTIF3C1, TIF3C1
1139	AT2G31660.1	2.32	t / c	|SAD2, URM9 | ARM repeat superfamily protein
121	AT4G35310.1	2.30	t / c	|CPK5, ATCPK5 | calmodulin-domain protein kin
278	AT4G31990.1 (+2)	2.29	t / c	|ASP5, AAT3, ATAAT1 | aspartate aminotransfer
541	AT1G09640.1	2.28	t / c	| | Translation elongation factor EF1B, gamma
1598	AT1G63940.4	2.27	t / c	|MDAR6 | monodehydroascorbate reductase 6 | c
1719	AT1G29880.1	2.27	t / c	| | glycyl-tRNA synthetase / glycine–tRNA li
1581	AT5G15090.1 (+1)	2.25	t / c	|VDAC3, ATVDAC3 | voltage dependent anion cha
501	AT3G24503.1	2.24	t / c	|ALDH2C4, ALDH1A, REF1 | aldehyde dehydrogena

	Accession.Number	log2tc	label	Description
1233	AT3G11400.2	2.24	t / c	|EIF3G1, ATEIF3G1 | eukaryotic translation in
255	AT4G33510.1	2.23	t / c	|DHS2 | 3-deoxy-d-arabino-heptulosonate 7-pho
576	AT1G34430.1	2.23	t / c	|EMB3003 | 2-oxoacid dehydrogenases acyltrans
416	AT1G23740.1	2.22	t / c	| | Oxidoreductase, zinc-binding dehydrogenas
910	AT2G05710.1	2.22	t / c	|ACO3 | aconitase 3 | chr2:2141591-2146350 FO
1567	AT2G46280.1 (+1)	2.22	t / c	|TRIP-1, TIF3I1 | TGF-beta receptor interacti
1647	AT5G42790.1	2.22	t / c	|PAF1, ATPSM30, ARS5 | proteasome alpha subun
1694	AT2G39990.1	2.22	t / c	|EIF2, AteIF3f, eIF3F | eukaryotic translatio
1847	AT1G60710.1	2.22	t / c	|ATB2 | NAD(P)-linked oxidoreductase superfam
1858	AT5G43830.1	2.22	t / c	| | Aluminium induced protein with YGL and LR
1525	AT2G17290.1	2.21	t / c	|CPK6, ATCDPK3, ATCPK6 | Calcium-dependent pr
1368	AT5G27640.2	2.20	t / c	|TIF3B1, EIF3B, ATEIF3B-1, EIF3B-1, ATTIF3B1
436	AT5G28840.1 (+1)	2.18	t / c	|GME | GDP-D-mannose 3’,5’-epimerase | chr5:1
864	AT5G61790.1	2.18	t / c	|CNX1, ATCNX1 | calnexin 1 | chr5:24827394-24
537	AT4G30920.1	2.17	t / c	| | Cytosol aminopeptidase family protein | c
67	AT5G25980.2	2.16	t / c	|TGG2, BGLU37 | glucoside glucohydrolase 2 |
564	AT1G65960.2	2.15	t / c	|GAD2 | glutamate decarboxylase 2 | chr1:2455
750	AT3G48000.1	2.15	t / c	|ALDH2B4, ALDH2, ALDH2A | aldehyde dehydrogen
2061	AT1G63810.1	2.15	t / c	| | CONTAINS InterPro DOMAIN/s: Nrap protein
690	AT1G50480.1	2.13	t / c	|THFS | 10-formyltetrahydrofolate synthetase
1517	AT5G25757.1 (+1)	2.13	t / c	| | RNA polymerase I-associated factor PAF67
940	AT1G80380.4	2.12	t / c	| | P-loop containing nucleoside triphosphate
1119	AT2G44160.1	2.12	t / c	|MTHFR2 | methylenetetrahydrofolate reductase
1151	AT3G59020.2	2.12	t / c	| | ARM repeat superfamily protein | chr3:218
1413	AT2G23200.1	2.12	t / c	| | Protein kinase superfamily protein | chr2
1580	AT5G22640.1	2.12	t / c	|emb1211 | MORN (Membrane Occupation and Reco
485	AT1G22530.1	2.12	t-unique	|PATL2 | PATELLIN 2 | chr1:7955773-7958326 RE
1588	AT3G57290.1	2.12	t-unique	|EIF3E, TIF3E1, ATEIF3E-1, INT-6, ATINT6, INT
1406	AT1G79920.1	2.11	t / c	| | Heat shock protein 70 (Hsp 70) family pro
161	AT3G44310.1 (+1)	2.10	t / c	|NIT1, ATNIT1, NITI | nitrilase 1 | chr3:1598

	Accession.Number	log2tc	label	Description
284	AT1G23190.1	2.06	t / c	|PGM3 | Phosphoglucomutase/phosphomannomutase
382	AT2G14110.1	2.06	t / c	| | Haloacid dehalogenase-like hydrolase (HAD
597	AT3G52880.1	2.06	t / c	|ATMDAR1, MDAR1 | monodehydroascorbate reduct
902	AT4G01370.1	2.06	t / c	|ATMPK4, MPK4 | MAP kinase 4 | chr4:567219-56
1167	AT2G47390.1	2.06	t / c	| | Prolyl oligopeptidase family protein | ch
1675	AT4G29840.1	2.06	t / c	|MTO2, TS | Pyridoxal-5’-phosphate-dependent
624	AT4G24190.1	2.04	t / c	|SHD, HSP90.7, AtHsp90.7, AtHsp90-7 | Chapero
1620	AT4G14040.1	2.04	t / c	|EDA38, SBP2 | selenium-binding protein 2 | c
684	AT5G14260.1 (+2)	2.03	t / c	| | Rubisco methyltransferase family protein
1165	AT4G02510.1	2.03	t / c	|TOC159, TOC86, PPI2, TOC160, ATTOC159 | tran
297	AT3G06650.1	2.02	t / c	|ACLB-1 | ATP-citrate lyase B-1 | chr3:207924
301	AT3G48750.1	2.02	t / c	|CDKA;1, CDC2AAT, CDK2, CDC2, CDC2A, CDKA1 |
1667	AT4G24620.1	2.02	t / c	|PGI1, PGI | phosphoglucose isomerase 1 | chr
300	AT1G01090.1	2.01	t / c	|PDH-E1 ALPHA | pyruvate dehydrogenase E1 alp
262	AT3G59780.1	2.00	t / c	| | Rhodanese/Cell cycle control phosphatase
529	AT5G66680.1	2.00	t / c	|DGL1 | dolichyl-diphosphooligosaccharide-pro
852	AT2G41530.1	2.00	t / c	|ATSFGH, SFGH | S-formylglutathione hydrolase
939	AT2G27860.1	2.00	t / c	|AXS1 | UDP-D-apiose/UDP-D-xylose synthase 1
1618	AT1G52600.1	2.00	t / c	| | Peptidase S24/S26A/S26B/S26C family prote
1682	AT3G20050.1	2.00	t / c	|ATTCP-1, TCP-1 | T-complex protein 1 alpha s
1787	AT5G66140.1	2.00	t / c	|PAD2 | proteasome alpha subunit D2 | chr5:26
1791	AT2G43560.1	2.00	t / c	| | FKBP-like peptidyl-prolyl cis-trans isome
1823	AT1G56050.1	2.00	t / c	| | GTP-binding protein-related | chr1:209637
1875	AT5G11880.1	2.00	t / c	| | Pyridoxal-dependent decarboxylase family
1910	AT1G12850.1	1.74	t-unique	| | Phosphoglycerate mutase family protein |
2044	AT5G51110.1 (+1)	1.42	t-unique	| | Transcriptional coactivator/pterin dehydr
1120	AT3G44620.1 (+1)	1.22	t-unique	| | protein tyrosine phosphatases;protein tyr
1236	AT2G39960.1	1.22	t-unique	| | Microsomal signal peptidase 25 kDa subuni
1521	AT4G27070.1	1.22	t-unique	|TSB2 | tryptophan synthase beta-subunit 2 |
1842	AT1G55480.1	1.22	t-unique	|ZKT | protein containing PDZ domain, a K-box

	Accession.Number	log2tc	label	Description
2147	AT5G59890.1	1.22	t-unique	|ADF4, ATADF4 | actin depolymerizing factor 4
779	AT4G36250.1	1.00	t-unique	|ALDH3F1 | aldehyde dehydrogenase 3F1 | chr4:
1728	AT3G28710.1	1.00	t-unique	| | ATPase, V0/A0 complex, subunit C/D | chr3
2107	AT4G35230.1	1.00	t-unique	|BSK1 | BR-signaling kinase 1 | chr4:16755325
719	AT3G47800.1	0.74	t-unique	| | Galactose mutarotase-like superfamily pro
2244	AT2G23600.1	0.74	t-unique	|ACL, ATMES2, MES2, ATME8, ME8 | acetone-cyan
1789	AT5G20090.1 (+1)	0.42	t-unique	| | Uncharacterised protein family (UPF0041)
1821	AT4G02420.1	0.00	t-unique	| | Concanavalin A-like lectin protein kinase
1952	AT5G59420.1	0.00	t-unique	|ORP3C | OSBP(oxysterol binding protein)-rela
1959	AT4G30610.1	0.00	c-unique	|BRS1, SCPL24 | alpha/beta-Hydrolases superfa
2145	AT3G24570.1	0.00	t-unique	| | Peroxisomal membrane 22 kDa (Mpv17/PMP22)
2277	AT4G30530.1	0.00	t-unique	| | Class I glutamine amidotransferase-like s
2327	AT4G34150.1	0.00	t-unique	| | Calcium-dependent lipid-binding (CaLB dom
737	AT1G74730.1	-0.49	c-unique	| | Protein of unknown function (DUF1118) | c
1328	AT3G25760.1	-0.85	c-unique	|AOC1, ERD12 | allene oxide cyclase 1 | chr3:
535	AT1G10290.1	-2.07	t / c	|ADL6, DRP2A | dynamin-like protein 6 | chr1:
637	AT1G59610.1	-2.15	t / c	|ADL3, CF1, DRP2B, DL3 | dynamin-like 3 | chr
689	AT2G30620.1	-2.20	t / c	| | winged-helix DNA-binding transcription fa
1571	AT5G09510.1	-2.26	t / c	| | Ribosomal protein S19 family protein | ch
183	AT1G28380.1	-4.02	t / c	|NSL1 | MAC/Perforin domain-containing protei

==================== [1] “Pair No.: 3” [1] “CPK28 (m22) /// CPK5 (m22)” =====================

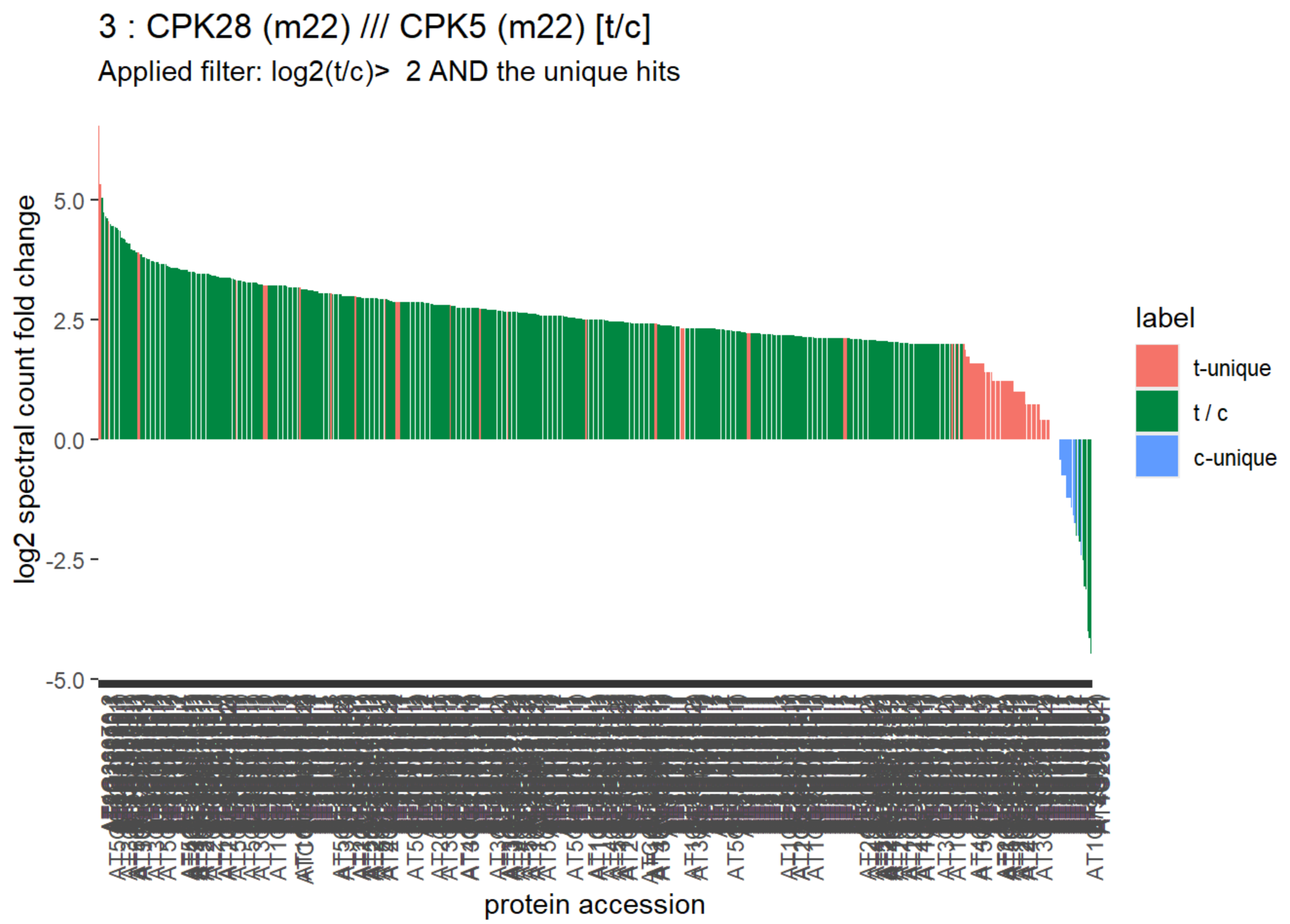

	Accession.Number	log2tc	label	Description
1530	AT4G36070.2	6.54	t-unique	|CPK18 | calcium-dependent protein kinase 18
1541	AT2G20140.1	5.33	t-unique	| | AAA-type ATPase family protein | chr2:869
27	AT2G17890.1	5.05	t / c	|CPK16 | calcium-dependent protein kinase 16
1476	AT4G28520.1	4.73	t / c	|CRU3, CRC | cruciferin 3 | chr4:14087596-140
826	AT5G46580.1	4.66	t / c	| | pentatricopeptide (PPR) repeat-containing
1	AT5G66210.1 (+1)	4.61	t / c	|CPK28 | calcium-dependent protein kinase 28
718	AT1G17840.1	4.56	t-unique	|WBC11, ABCG11, DSO, COF1, ATWBC11 | white-br
108	AT4G30190.1	4.49	t / c	|AHA2, PMA2, HA2 | H(+)-ATPase 2 | chr4:14770
435	AT4G35470.1	4.46	t / c	|PIRL4 | plant intracellular ras group-relate
536	AT1G01790.1	4.46	t / c	|KEA1, ATKEA1 | K+ efflux antiporter 1 | chr1
1200	AT2G46520.1	4.44	t / c	| | cellular apoptosis susceptibility protein
317	AT2G22500.1	4.42	t / c	|UCP5, ATPUMP5, DIC1 | uncoupling protein 5 |
245	AT3G59350.1 (+1)	4.37	t / c	| | Protein kinase superfamily protein | chr3
434	AT1G79050.1	4.35	t / c	| | recA DNA recombination family protein | c

	Accession.Number	log2tc	label	Description
1510	AT5G20490.1	4.22	t / c	|XIK, ATXIK, XI-17 | Myosin family protein wi
1047	AT1G30360.1	4.20	t / c	|ERD4 | Early-responsive to dehydration stres
1149	AT2G41560.1	4.17	t / c	|ACA4 | autoinhibited Ca(2+)-ATPase, isoform
1066	AT5G57110.1 (+1)	4.12	t / c	|ACA8, AT-ACA8 | autoinhibited Ca2+ -ATPase,
1168	AT1G18270.3	4.09	t / c	| | ketose-bisphosphate aldolase class-II fam
1621	AT1G05150.1	4.09	t / c	| | Calcium-binding tetratricopeptide family
477	AT1G06700.1 (+1)	3.97	t / c	| | Protein kinase superfamily protein | chr1
75	AT5G13490.1 (+1)	3.95	t / c	|AAC2 | ADP/ATP carrier 2 | chr5:4336034-4337
874	AT1G79600.1	3.94	t / c	| | Protein kinase superfamily protein | chr1
1036	AT1G01960.1	3.91	t / c	|EDA10 | SEC7-like guanine nucleotide exchang
674	AT4G19710.2	3.91	t-unique	|AK-HSDH II, AK-HSDH | aspartate kinase-homos
1357	AT2G31880.1	3.87	t-unique	|SOBIR1, EVR | Leucine-rich repeat protein ki
141	AT3G52750.1	3.87	t / c	|FTSZ2-2 | Tubulin/FtsZ family protein | chr3
99	AT2G18960.1	3.81	t / c	|AHA1, PMA, OST2, HA1 | H(+)-ATPase 1 | chr2:
1674	AT1G60780.1	3.81	t / c	|HAP13 | Clathrin adaptor complexes medium su
1493	AT3G08510.1 (+1)	3.78	t / c	|ATPLC2, PLC2 | phospholipase C 2 | chr3:2582
715	AT5G55610.1	3.77	t / c	| | unknown protein; LOCATED IN: mitochondrio
1102	AT3G43300.1	3.77	t / c	|ATMIN7, BEN1 | HOPM interactor 7 | chr3:1523
425	AT4G10790.1	3.73	t / c	| | UBX domain-containing protein | chr4:6640
1562	AT3G14840.2	3.72	t / c	| | Leucine-rich repeat transmembrane protein
527	AT1G06410.1	3.70	t / c	|ATTPS7, TPS7, ATTPSA | trehalose-phosphatase
840	AT1G03160.1	3.70	t / c	|FZL | FZO-like | chr1:761321-766053 FORWARD
1627	AT5G48880.2 (+1)	3.70	t / c	|PKT2, KAT5 | peroxisomal 3-keto-acyl-CoA thi
679	AT4G37925.1	3.67	t / c	|NDH-M | subunit NDH-M of NAD(P)H:plastoquino
1000	AT3G46970.1	3.67	t / c	|ATPHS2, PHS2 | alpha-glucan phosphorylase 2
1538	AT1G02150.1	3.67	t / c	| | Tetratricopeptide repeat (TPR)-like super
1303	AT4G29900.1	3.66	t / c	|ACA10, CIF1, ATACA10 | autoinhibited Ca(2+)-
1524	AT1G30470.1	3.66	t / c	| | SIT4 phosphatase-associated family protei
1017	AT5G38660.2	3.62	t / c	|APE1 | acclimation of photosynthesis to env
158	AT1G80480.1	3.60	t / c	|PTAC17 | plastid transcriptionally active 17

	Accession.Number	log2tc	label	Description
313	AT4G29130.1	3.58	t / c	|ATHXK1, GIN2, HXK1 | hexokinase 1 | chr4:143
359	AT1G54520.1	3.58	t / c	| | unknown protein; FUNCTIONS IN: molecular_
994	AT3G29320.1	3.58	t / c	| | Glycosyl transferase, family 35 | chr3:11
1234	AT1G31230.1	3.58	t / c	|AK-HSDH I, AK-HSDH | aspartate kinase-homose
1688	AT2G18730.1	3.58	t / c	|ATDGK3, DGK3 | diacylglycerol kinase 3 | chr
595	AT5G05010.1 (+1)	3.56	t / c	| | clathrin adaptor complexes medium subunit
500	AT4G31480.1 (+1)	3.55	t / c	| | Coatomer, beta subunit | chr4:15264145-15
1344	AT5G61020.1	3.55	t / c	|ECT3 | evolutionarily conserved C-terminal r
774	AT4G25960.1	3.54	t / c	|PGP2 | P-glycoprotein 2 | chr4:13177438-1318
1148	AT3G61050.1 (+1)	3.54	t / c	|NTMC2TYPE4, NTMC2T4 | Calcium-dependent lipi
1623	AT1G52360.1	3.54	t / c	| | Coatomer, beta' subunit | chr1:19499282-1
696	AT1G13320.1 (+1)	3.51	t / c	|PP2AA3 | protein phosphatase 2A subunit A3
1070	AT3G25800.1	3.51	t / c	|PDF1, PR 65, PP2AA2 | protein phosphatase 2A
512	AT3G01060.1	3.50	t / c	| | unknown protein; Has 640 Blast hits to 63
879	AT5G64940.1 (+1)	3.50	t / c	|ATATH13, ATH13, ATOSA1, OSA1 | ABC2 homolog
394	AT2G38040.1 (+1)	3.48	t / c	|CAC3 | acetyl Co-enzyme a carboxylase carbox
180	AT3G44110.1	3.47	t / c	|ATJ3, ATJ | DNAJ homologue 3 | chr3:15869115
254	AT1G64740.1	3.47	t / c	|TUA1 | alpha-1 tubulin | chr1:24050114-24052
458	AT4G22690.1	3.47	t / c	|CYP706A1 | cytochrome P450, family 706, subf
620	AT5G52320.1	3.46	t / c	|CYP96A4 | cytochrome P450, family 96, subfam
678	AT2G46820.1 (+1)	3.46	t / c	|PTAC8, TMP14, PSAP, PSI-P | photosystem I P
1207	AT5G23630.1	3.46	t / c	|PDR2, MIA | phosphate deficiency response 2
1547	AT3G05590.1	3.46	t / c	|RPL18 | ribosomal protein L18 | chr3:1621511
1585	AT4G23650.1	3.46	t / c	|CDPK6, CPK3 | calcium-dependent protein kina
1489	AT4G38630.1	3.43	t / c	|RPN10, MCB1, ATMCB1, MBP1 | regulatory parti
1020	AT5G66200.1	3.42	t / c	|ARO2 | armadillo repeat only 2 | chr5:264536
1231	AT5G04130.1	3.42	t / c	|GYRB2 | DNA GYRASE B2 | chr5:1122084-1128031
1459	AT4G24330.1	3.42	t / c	| | Protein of unknown function (DUF1682) | c
539	AT1G74960.1 (+2)	3.39	t / c	|FAB1, KAS2, ATKAS2 | fatty acid biosynthesis
557	AT2G35840.1 (+2)	3.39	t / c	| | Sucrose-6F-phosphate phosphohydrolase fam

	Accession.Number	log2tc	label	Description
134	AT3G54890.1	3.38	t / c	|LHCA1 | photosystem I light harvesting compl
1478	AT5G27380.1	3.38	t / c	|GSH2, GSHB | glutathione synthetase 2 | chr5
582	AT1G06530.1	3.37	t / c	| | Tropomyosin-related | chr1:2001625-200259
929	AT2G47450.1	3.37	t / c	|CAO, CPSRP43 | chloroplast signal recognitio
1277	AT1G51500.1	3.37	t / c	|CER5, D3, ABCG12, WBC12, ATWBC12 | ABC-2 typ
1629	AT5G06530.1 (+1)	3.37	t / c	| | ABC-2 type transporter family protein | c
2121	AT3G22640.1	3.37	t / c	|PAP85 | cupin family protein | chr3:8011902-
142	AT5G62670.1	3.35	t / c	|AHA11, HA11 | H(+)-ATPase 11 | chr5:25159495
855	AT5G17380.1	3.35	t / c	| | Thiamine pyrophosphate dependent pyruvate
296	AT3G54110.1	3.34	t / c	|ATPUMP1, UCP, PUMP1, ATUCP1, UCP1 | plant un
1759	AT4G37200.1	3.32	t-unique	|HCF164 | Thioredoxin superfamily protein | c
658	AT4G33090.1	3.32	t / c	|APM1, ATAPM1 | aminopeptidase M1 | chr4:1596
1326	AT5G42390.1	3.32	t / c	| | Insulinase (Peptidase family M16) family
1526	AT5G23860.1 (+1)	3.31	t / c	|TUB8 | tubulin beta 8 | chr5:8042962-8044528
631	AT3G07770.1	3.29	t / c	|Hsp89.1, AtHsp90.6, AtHsp90-6 | HEAT SHOCK P
639	AT4G00630.1	3.29	t / c	|KEA2, ATKEA2 | K+ efflux antiporter 2 | chr4
785	AT1G29310.1	3.27	t / c	| | SecY protein transport family protein | c
966	AT5G64740.1	3.27	t / c	|CESA6, IXR2, E112, PRC1 | cellulose synthase
1401	AT5G13430.1	3.27	t / c	| | Ubiquinol-cytochrome C reductase iron-sul
1482	AT3G50950.1 (+1)	3.27	t / c	|ZAR1 | HOPZ-ACTIVATED RESISTANCE 1 | chr3:18
1748	AT5G02890.1	3.27	t / c	| | HXXXD-type acyl-transferase family protei
1749	AT4G30340.1	3.27	t / c	|ATDGK7, DGK7 | diacylglycerol kinase 7 | chr
1766	AT2G34560.1	3.27	t / c	| | P-loop containing nucleoside triphosphate
165	AT5G65720.1	3.25	t / c	|ATNIFS1, NIFS1, NFS1, ATNFS1 | nitrogen fixa
391	AT5G22060.1	3.23	t / c	|ATJ2, J2 | DNAJ homologue 2 | chr5:7303798-7
452	AT3G63260.1	3.23	t / c	|ATMRK1 | Protein kinase superfamily protein
754	AT1G59870.1	3.23	t / c	|PEN3, PDR8, ATPDR8, ABCG36, ATABCG36 | ABC-2
1705	AT1G34000.1	3.22	t-unique	|OHP2 | one-helix protein 2 | chr1:12358151-1
1729	AT5G58670.1	3.22	t-unique	|ATPLC1, ATPLC, PLC1 | phospholipase C1 | chr
1795	AT1G64430.1 (+1)	3.22	t-unique	| | Pentatricopeptide repeat (PPR) superfamil

	Accession.Number	log2tc	label	Description
468	AT3G51820.1	3.22	t / c	|ATG4, G4, CHLG | UbiA prenyltransferase fami
493	AT5G03880.1	3.22	t / c	| | Thioredoxin family protein | chr5:1038674
1214	AT5G43900.3	3.22	t / c	|MYA2 | myosin 2 | chr5:17657241-17667413 REV
1281	AT3G28860.1	3.22	t / c	|ATMDR1, ATMDR11, PGP19, MDR11, MDR1, ATPGP19
1302	AT1G70320.1	3.22	t / c	|UPL2 | ubiquitin-protein ligase 2 | chr1:264
1418	AT1G01220.1	3.22	t / c	|FKGP, AtFKGP | L-fucokinase/GDP-L-fucose pyr
1760	AT3G45190.1	3.22	t / c	| | SIT4 phosphatase-associated family protei
654	AT2G19860.1	3.21	t / c	|ATHXK2, HXK2 | hexokinase 2 | chr2:8570818-8
751	AT3G53520.4	3.21	t / c	|UXS1 | UDP-glucuronic acid decarboxylase 1 |
1136	AT3G44340.1	3.21	t / c	|CEF | clone eighty-four | chr3:16011923-1601
1638	AT5G13630.1	3.21	t / c	|GUN5, CCH, CHLH, CCH1, ABAR | magnesium-chel
489	AT2G41790.1	3.19	t / c	| | Insulinase (Peptidase family M16) family
565	ATCG00430.1	3.17	t / c	|PSBG | photosystem II reaction center protei
1099	AT4G16990.2	3.17	t / c	|RLM3 | disease resistance protein (TIR-NBS c
1124	AT2G43950.1	3.17	t / c	|OEP37, ATOEP37 | chloroplast outer envelope
1199	AT1G78915.1 (+2)	3.17	t / c	| | Tetratricopeptide repeat (TPR)-like super
1346	ATCG00840.1 (+1)	3.17	t / c	|RPL23.1, RPL23 | ribosomal protein L23.1 | c
1632	AT1G07650.2	3.17	t / c	| | Leucine-rich repeat transmembrane protein
1871	AT3G23660.1	3.17	t / c	| | Sec23/Sec24 protein transport family prot
804	AT1G08380.1	3.17	t-unique	|PSAO | photosystem I subunit O | chr1:264100
497	AT1G73990.1	3.14	t / c	|SPPA, SPPA1 | signal peptide peptidase | chr
901	AT4G13770.1	3.13	t / c	|CYP83A1, REF2 | cytochrome P450, family 83,
1398	AT4G04040.1	3.13	t / c	|MEE51 | Phosphofructokinase family protein |
1684	AT3G51160.1	3.13	t / c	|MUR1, MUR_1, GMD2 | NAD(P)-binding Rossmann-
263	AT1G73110.1	3.12	t / c	| | P-loop containing nucleoside triphosphate
688	AT1G01610.1	3.12	t / c	|ATGPAT4, GPAT4 | glycerol-3-phosphate acyltr
938	AT3G62700.1	3.12	t / c	|ATMRP10, MRP10, ABCC14 | multidrug resistanc
1227	AT4G16130.1	3.12	t / c	|ARA1, ISA1, ATISA1 | arabinose kinase | chr4
655	AT1G15730.1	3.10	t / c	| | Cobalamin biosynthesis CobW-like protein
1622	AT2G32450.1	3.10	t / c	| | Calcium-binding tetratricopeptide family

	Accession.Number	log2tc	label	Description
111	AT5G46800.1	3.09	t / c	|BOU | Mitochondrial substrate carrier family
178	AT1G09130.3	3.06	t / c	| | ATP-dependent caseinolytic (Clp) protease
607	AT2G33530.1	3.06	t / c	|scpl46 | serine carboxypeptidase-like 46 | c
906	AT5G09870.1	3.06	t / c	|CESA5 | cellulose synthase 5 | chr5:3073356-
1464	AT3G04340.1	3.06	t / c	|emb2458 | FtsH extracellular protease family
1468	AT4G21710.1	3.06	t / c	|NRPB2, EMB1989, RPB2 | DNA-directed RNA poly
1702	AT2G27730.1	3.06	t / c	| | copper ion binding | chr2:11820056-118208
1780	AT2G29200.1	3.06	t / c	|APUM1, PUM1 | pumilio 1 | chr2:12549483-1255
1150	AT5G03040.1 (+2)	3.06	t-unique	|iqd2 | IQ-domain 2 | chr5:710380-712406 REVE
209	AT5G46290.1	3.04	t / c	|KASI, KAS1 | 3-ketoacyl-acyl carrier protein
657	AT2G20580.1	3.04	t / c	|RPN1A, ATRPN1A | 26S proteasome regulatory s
669	AT3G15980.1 (+3)	3.04	t / c	| | Coatomer, beta' subunit | chr3:5411699-54
691	AT5G19690.1	3.04	t / c	|STT3A | staurosporin and temperature sensiti
1076	AT3G26710.1	3.03	t / c	|CCB1 | cofactor assembly of complex C | chr3
1614	AT2G39010.1	3.03	t / c	|PIP2E, PIP2;6 | plasma membrane intrinsic pr
243	AT3G55360.1	3.00	t / c	|CER10, ECR, ATTSC13, TSC13 | 3-oxo-5-alpha-s
290	AT1G68830.1	3.00	t / c	|STN7 | STT7 homolog STN7 | chr1:25872654-258
601	AT5G24690.1	3.00	t / c	| | Protein of unknown function (DUF3411) | c
894	AT1G11260.1	3.00	t / c	|STP1, ATSTP1 | sugar transporter 1 | chr1:37
935	AT1G70940.1	3.00	t / c	|PIN3, ATPIN3 | Auxin efflux carrier family p
1041	AT2G29190.1 (+1)	3.00	t / c	|APUM2, PUM2 | pumilio 2 | chr2:12544260-1254
1083	AT1G08930.1 (+1)	3.00	t / c	|ERD6 | Major facilitator superfamily protein
1578	AT5G48620.1	3.00	t / c	| | Disease resistance protein (CC-NBS-LRR cl
1747	AT5G02160.1	3.00	t-unique	| | unknown protein; FUNCTIONS IN: molecular_
201	AT2G44490.1	2.98	t / c	|PEN2, BGLU26 | Glycosyl hydrolase superfamil
360	AT2G32480.1	2.97	t / c	|ARASP | ARABIDOPSIS SERIN PROTEASE | chr2:13
1583	AT5G43470.1 (+1)	2.97	t / c	|RPP8, HRT, RCY1 | Disease resistance protein
203	AT2G47110.1 (+1)	2.96	t / c	|UBQ6 | ubiquitin 6 | chr2:19344701-19345174
326	AT1G45000.1	2.96	t / c	| | AAA-type ATPase family protein | chr1:170
88	AT2G36250.1 (+1)	2.95	t / c	|FTSZ2-1, ATFTSZ2-1 | Tubulin/FtsZ family pro

	Accession.Number	log2tc	label	Description
160	AT5G58290.1	2.95	t / c	|RPT3 | regulatory particle triple-A ATPase 3
211	AT5G60790.1	2.95	t / c	|ATGCN1, GCN1 | ABC transporter family protei
471	AT1G56500.1	2.95	t / c	| | haloacid dehalogenase-like hydrolase fami
514	AT5G12860.1 (+1)	2.95	t / c	|DiT1 | dicarboxylate transporter 1 | chr5:40
561	AT5G05780.1	2.95	t / c	|RPN8A, AE3, ATHMOV34 | RP non-ATPase subunit
1043	AT5G63420.1	2.95	t / c	|emb2746 | RNA-metabolising metallo-beta-lact
1593	AT3G62010.2	2.95	t / c	| | unknown protein; LOCATED IN: cellular_com
265	AT4G22890.1 (+2)	2.94	t / c	|PGR5-LIKE A | PGR5-LIKE A | chr4:12007157-12
866	AT2G47240.1 (+1)	2.94	t / c	|CER8, LACS1 | AMP-dependent synthetase and l
1019	AT4G34830.1	2.94	t / c	|MRL1 | Pentatricopeptide repeat (PPR) superf
1852	AT4G04850.2	2.94	t / c	|KEA3 | K+ efflux antiporter 3 | chr4:2453174
816	AT2G23670.1	2.94	t-unique	|YCF37 | homolog of Synechocystis YCF37 | chr
889	AT3G23820.1	2.93	t / c	|GAE6 | UDP-D-glucuronate 4-epimerase 6 | chr
971	AT3G07160.1	2.91	t / c	|ATGSL10, gsl10, CALS9 | glucan synthase-like
306	AT3G63160.1	2.90	t / c	| | FUNCTIONS IN: molecular_function unknown;
1619	AT2G45710.1	2.89	t / c	| | Zinc-binding ribosomal protein family pro
198	AT3G62250.1	2.88	t / c	|UBQ5 | ubiquitin 5 | chr3:23037138-23037611
1592	AT4G39960.1	2.88	t / c	| | Molecular chaperone Hsp40/DnaJ family pro
737	AT1G74730.1	2.87	t-unique	| | Protein of unknown function (DUF1118) | c
988	AT3G52500.1	2.87	t-unique	| | Eukaryotic aspartyl protease family prote
1754	AT5G60540.1	2.87	t-unique	|EMB2407, ATPDX2, PDX2 | pyridoxine biosynthe
553	AT5G07030.1	2.87	t / c	| | Eukaryotic aspartyl protease family prote
592	AT1G71810.1	2.87	t / c	| | Protein kinase superfamily protein | chr1
887	AT5G17020.1 (+1)	2.87	t / c	|XPO1A, ATCRM1, ATXPO1, XPO1, HIT2 | exportin
918	AT3G10670.1	2.87	t / c	|ATNAP7, NAP7 | non-intrinsic ABC protein 7 |
1040	AT4G31500.1	2.87	t / c	|CYP83B1, SUR2, RNT1, RED1, ATR4 | cytochrome
1247	AT4G24810.2	2.87	t / c	| | Protein kinase superfamily protein | chr4
1365	AT4G15550.1	2.87	t / c	|IAGLU | indole-3-acetate beta-D-glucosyltran
1540	AT4G36130.1	2.87	t / c	| | Ribosomal protein L2 family | chr4:170976
1639	ATCG01060.1	2.87	t / c	|PSAC | iron-sulfur cluster binding;electron

	Accession.Number	log2tc	label	Description
1710	AT5G59730.2	2.87	t / c	|ATEXO70H7, EXO70H7 | exocyst subunit exo70 f
1723	AT4G23250.1	2.87	t / c	|EMB1290, DUF26-21, RKC1, CRK17 | kinases;pro
1726	AT3G51550.1	2.87	t / c	|FER | Malectin/receptor-like protein kinase
1734	AT1G19450.1	2.87	t / c	| | Major facilitator superfamily protein | c
1799	AT1G22710.1	2.87	t / c	|SUC2, SUT1, ATSUC2 | sucrose-proton symporte
1309	AT4G32410.1	2.86	t / c	|CESA1, RSW1, AtCESA1 | cellulose synthase 1
1571	AT5G09510.1	2.86	t / c	| | Ribosomal protein S19 family protein | ch
286	AT1G64190.1	2.85	t / c	| | 6-phosphogluconate dehydrogenase family p
303	AT2G32080.1 (+1)	2.85	t / c	|PUR ALPHA-1 | purin-rich alpha 1 | chr2:1364
1323	AT5G08540.1	2.84	t / c	| | unknown protein; FUNCTIONS IN: molecular_
603	AT4G16390.1	2.83	t / c	|SVR7 | pentatricopeptide (PPR) repeat-contai
40	AT2G29550.1	2.81	t / c	|TUB7 | tubulin beta-7 chain | chr2:12644258-
546	AT2G45060.1	2.81	t / c	| | Uncharacterised conserved protein UCP0222
695	AT5G24650.1	2.81	t / c	| | Mitochondrial import inner membrane trans
837	AT3G20810.1 (+1)	2.81	t / c	|JMJD5 | 2-oxoglutarate (2OG) and Fe(II)-depe
863	AT3G01310.2	2.81	t / c	| | Phosphoglycerate mutase-like family prote
1115	AT1G73650.3	2.81	t / c	| | Protein of unknown function (DUF1295) | c
1173	AT4G03550.1	2.81	t / c	|ATGSL05, GSL05, ATGSL5, PMR4, GSL5 | glucan
1752	AT4G38580.1	2.81	t / c	|ATFP6, HIPP26, FP6 | farnesylated protein 6
1884	AT5G05200.1	2.81	t / c	| | Protein kinase superfamily protein | chr5
1940	AT3G47620.1	2.81	t / c	|AtTCP14, TCP14 | TEOSINTE BRANCHED, cycloide
1713	AT2G38670.1	2.81	t-unique	|PECT1 | phosphorylethanolamine cytidylyltran
113	AT3G08940.2	2.79	t / c	|LHCB4.2 | light harvesting complex photosyst
451	AT1G30380.1	2.79	t / c	|PSAK | photosystem I subunit K | chr1:107223
824	AT5G58260.1	2.78	t / c	| | oxidoreductases, acting on NADH or NADPH,
45	AT4G20890.1	2.75	t / c	|TUB9 | tubulin beta-9 chain | chr4:11182218-
275	AT4G35100.1 (+1)	2.75	t / c	|PIP3, PIP3A, PIP2;7, SIMIP | plasma membrane
478	AT3G02360.1 (+1)	2.75	t / c	| | 6-phosphogluconate dehydrogenase family p
570	AT1G01320.2	2.75	t / c	| | Tetratricopeptide repeat (TPR)-like super
1514	AT1G21250.1	2.75	t / c	|WAK1, PRO25 | cell wall-associated kinase |

	Accession.Number	log2tc	label	Description
1680	AT5G11770.1	2.75	t / c	| | NADH-ubiquinone oxidoreductase 20 kDa sub
31	AT5G44340.1	2.74	t / c	|TUB4 | tubulin beta chain 4 | chr5:17859442-
328	AT1G06430.1	2.74	t / c	|FTSH8 | FTSH protease 8 | chr1:1960214-19625
341	AT1G10510.1	2.74	t / c	|emb2004 | RNI-like superfamily protein | chr
964	AT1G17580.1	2.74	t / c	|MYA1, ATMYA1, XI-1 | myosin 1 | chr1:6039453
1052	AT3G57650.1	2.74	t / c	|LPAT2 | lysophosphatidyl acyltransferase 2 |
1472	AT5G11380.1	2.74	t / c	|DXPS3 | 1-deoxy-D-xylulose 5-phosphate synth
1779	AT2G22125.1	2.74	t / c	|CSI1 | binding | chr2:9406793-9414223 FORWAR
1859	AT3G02350.1	2.74	t / c	|GAUT9 | galacturonosyltransferase 9 | chr3:4
972	AT2G25800.1	2.74	t-unique	| | Protein of unknown function (DUF810) | ch
82	AT1G06950.1	2.73	t / c	|ATTIC110, TIC110 | translocon at the inner e
122	ATCG00470.1	2.73	t / c	|ATPE | ATP synthase epsilon chain | chrC:522
1379	AT5G45390.1	2.72	t / c	|CLPP4, NCLPP4 | CLP protease P4 | chr5:18396
1565	AT1G27400.1	2.72	t / c	| | Ribosomal protein L22p/L17e family protei
175	AT3G19820.1 (+2)	2.70	t / c	|DWF1, DIM, EVE1, DIM1, CBB1 | cell elongatio
184	AT2G30950.1	2.70	t / c	|VAR2, FTSH2 | FtsH extracellular protease fa
239	AT1G79040.1	2.70	t / c	|PSBR | photosystem II subunit R | chr1:29736
332	AT4G01100.1	2.70	t / c	|ADNT1 | adenine nucleotide transporter 1 | c
1587	AT2G39390.1	2.70	t / c	| | Ribosomal L29 family protein | chr2:1645
1739	AT4G12320.1	2.70	t / c	|CYP706A6 | cytochrome P450, family 706, subf
697	AT2G32730.1	2.69	t / c	| | 26S proteasome regulatory complex, non-AT
722	AT3G62830.1 (+1)	2.68	t / c	|UXS2, ATUXS2, AUD1 | NAD(P)-binding Rossmann
1451	AT3G46060.1 (+2)	2.68	t / c	|ARA3, ARA-3, ATRABE1C, ATRAB8A, RAB8A | RAB
1652	AT3G09740.1	2.68	t / c	|SYP71, ATSYP71 | syntaxin of plants 71 | chr
35	AT3G08580.1 (+1)	2.67	t / c	|AAC1 | ADP/ATP carrier 1 | chr3:2605706-2607
246	AT1G53750.1	2.67	t / c	|RPT1A | regulatory particle triple-A 1A | ch
1931	AT1G22700.2	2.66	t-unique	| | Tetratricopeptide repeat (TPR)-like super
815	AT5G21430.1	2.66	t / c	| | Chaperone DnaJ-domain superfamily protein
1146	AT4G10120.1 (+1)	2.66	t / c	|ATSPS4F | Sucrose-phosphate synthase family
1387	AT5G27390.1	2.66	t / c	| | Mog1/PsbP/DUF1795-like photosystem II rea

	Accession.Number	log2tc	label	Description
1400	AT5G16715.1	2.66	t / c	|EMB2247 | ATP binding;valine-tRNA ligases;am
1496	AT1G09430.1	2.66	t / c	|ACLA-3 | ATP-citrate lyase A-3 | chr1:304213
2085	AT1G14930.1	2.66	t / c	| | Polyketide cyclase/dehydrase and lipid tr
26	AT5G62690.1 (+1)	2.65	t / c	|TUB2 | tubulin beta chain 2 | chr5:25181560-
48	AT5G12250.1	2.65	t / c	|TUB6 | beta-6 tubulin | chr5:3961317-3962971
1101	AT3G07100.1	2.65	t / c	|ERMO2, SEC24A | Sec23/Sec24 protein transpor
1566	ATCG00780.1	2.65	t / c	|RPL14 | ribosomal protein L14 | chrC:80696-8
329	AT1G58684.1 (+2)	2.64	t / c	| | Ribosomal protein S5 family protein | chr
1074	AT1G62640.1 (+1)	2.64	t / c	|KAS III | 3-ketoacyl-acyl carrier protein sy
584	AT1G20200.1	2.63	t / c	|EMB2719, HAP15 | PAM domain (PCI/PINT associ
846	AT3G20000.1	2.63	t / c	|TOM40 | translocase of the outer mitochondri
146	AT1G71500.1	2.62	t / c	| | Rieske (2Fe-2S) domain-containing protein
395	AT1G03475.1	2.62	t / c	|LIN2, HEMF1, ATCPO-I | Coproporphyrinogen II
1665	AT1G72750.1	2.62	t / c	|ATTIM23-2, TIM23-2 | translocase inner membr
152	AT5G57350.1 (+1)	2.61	t / c	|AHA3, ATAHA3, HA3 | H(+)-ATPase 3 | chr5:232
427	AT5G14780.1	2.60	t / c	|FDH | formate dehydrogenase | chr5:4777043-4
157	ATCG00800.1	2.59	t / c	| | structural constituent of ribosome | chrC
190	AT2G04030.2	2.59	t / c	|CR88, Hsp88.1, AtHsp90.5 | Chaperone protein
388	AT1G05140.1	2.58	t / c	| | Peptidase M50 family protein | chr1:14826
524	AT3G63460.1	2.58	t / c	| | transducin family protein / WD-40 repeat
555	AT1G12770.1	2.58	t / c	|ISE1, EMB1586 | P-loop containing nucleoside
630	AT3G25680.1	2.58	t / c	| | FUNCTIONS IN: molecular_function unknown;
644	AT1G11410.1	2.58	t / c	| | S-locus lectin protein kinase family prot
776	AT5G05170.1	2.58	t / c	|CESA3, IXR1, ATCESA3, ATH-B, CEV1 | Cellulos
1005	AT5G47910.1	2.58	t / c	|RBOHD, ATRBOHD | respiratory burst oxidase h
1186	ATCG00660.1	2.58	t / c	|RPL20 | ribosomal protein L20 | chrC:68512-6
1628	AT3G02450.1	2.58	t / c	| | cell division protein ftsH, putative | ch
1714	AT5G47930.1	2.58	t / c	| | Zinc-binding ribosomal protein family pro
1716	AT4G02620.1	2.58	t / c	| | vacuolar ATPase subunit F family protein
1814	AT4G33220.1	2.58	t / c	|PME44, ATPME44 | pectin methylesterase 44 |

	Accession.Number	log2tc	label	Description
1891	AT4G31390.1	2.58	t / c	| | Protein kinase superfamily protein | chr4
319	AT5G41670.1 (+1)	2.57	t / c	| | 6-phosphogluconate dehydrogenase family p
105	AT3G47470.1	2.56	t / c	|LHCA4, CAB4 | light-harvesting chlorophyll-p
30	AT1G20010.1	2.55	t / c	|TUB5 | tubulin beta-5 chain | chr1:6938033-6
488	AT5G17170.1	2.55	t / c	|ENH1 | rubredoxin family protein | chr5:5649
274	AT5G19990.1	2.54	t / c	|RPT6A, ATSUG1 | regulatory particle triple-A
616	AT2G47650.1	2.54	t / c	|UXS4 | UDP-xylose synthase 4 | chr2:19538751
1179	AT5G58410.1	2.54	t / c	| | HEAT/U-box domain-containing protein | ch
118	AT5G19760.1	2.53	t / c	| | Mitochondrial substrate carrier family pr
760	AT3G27240.1	2.53	t / c	| | Cytochrome C1 family | chr3:10056144-1005
1584	AT5G01920.1	2.52	t / c	|STN8 | Protein kinase superfamily protein |
1595	AT4G25740.1	2.52	t / c	| | RNA binding Plectin/S10 domain-containing
659	AT2G31810.1	2.51	t / c	| | ACT domain-containing small subunit of ac
812	AT3G52730.1	2.51	t / c	| | ubiquinol-cytochrome C reductase UQCRX/QC
1770	AT1G15930.1 (+1)	2.50	t-unique	| | Ribosomal protein L7Ae/L30e/S12e/Gadd45 f
465	AT4G36220.1	2.50	t / c	|FAH1, CYP84A1 | ferulic acid 5-hydroxylase 1
873	AT1G30400.1 (+1)	2.50	t / c	|ATMRP1, EST1, ABCC1, ATABCC1, MRP1 | multidr
1011	AT2G36810.1	2.50	t / c	| | ARM repeat superfamily protein | chr2:154
1319	AT5G54160.1	2.50	t / c	|ATOMT1, OMT1 | O-methyltransferase 1 | chr5:
1327	AT1G23080.3	2.50	t / c	|PIN7 | Auxin efflux carrier family protein |
1439	AT1G44446.1	2.50	t / c	|CH1, ATCAO, CAO | Pheophorbide a oxygenase f
1712	AT4G30810.1	2.50	t / c	|scpl29 | serine carboxypeptidase-like 29 | c
1730	AT4G24750.1	2.50	t / c	| | Rhodanese/Cell cycle control phosphatase
1853	AT4G36480.1 (+1)	2.50	t / c	|ATLCB1, LCB1, EMB2779, FBR11 | long-chain ba
1899	AT4G17770.1	2.50	t / c	|ATTPS5, TPS5 | trehalose phosphatase/synthas
1927	AT1G50370.1	2.50	t / c	| | Calcineurin-like metallo-phosphoesterase
156	AT5G26742.2	2.49	t / c	|emb1138 | DEAD box RNA helicase (RH3) | chr5
878	AT5G22770.1 (+2)	2.49	t / c	|alpha-ADR | alpha-adaptin | chr5:7579844-758
365	AT1G54780.1	2.47	t / c	|TLP18.3 | thylakoid lumen 18.3 kDa protein |
1126	AT1G07320.1	2.47	t / c	|RPL4 | ribosomal protein L4 | chr1:2249190-2

	Accession.Number	log2tc	label	Description
340	AT1G70410.2	2.46	t / c	|ATBCA4, BCA4 | beta carbonic anhydrase 4 | c
447	AT3G09790.1	2.46	t / c	|UBQ8 | ubiquitin 8 | chr3:3004111-3006006 RE
766	AT1G80030.1 (+2)	2.46	t / c	| | Molecular chaperone Hsp40/DnaJ family pro
946	AT5G64580.1	2.46	t / c	| | AAA-type ATPase family protein | chr5:258
1187	AT2G07707.1 (+1)	2.46	t / c	| | Plant mitochondrial ATPase, F0 complex, s
1685	AT1G25490.1	2.46	t / c	|RCN1, REGA, ATB BETA BETA, EER1 | ARM repeat
1697	AT1G63000.1	2.46	t / c	|NRS/ER, UER1 | nucleotide-rhamnose synthase/
1724	AT4G22310.1	2.46	t / c	| | Uncharacterised protein family (UPF0041)
345	AT5G16070.1	2.45	t / c	| | TCP-1/cpn60 chaperonin family protein | c
1527	AT1G75780.1	2.45	t / c	|TUB1 | tubulin beta-1 chain | chr1:28451378-
314	AT5G55280.1	2.44	t / c	|FTSZ1-1, ATFTSZ1-1, CPFTSZ | homolog of bact
492	AT2G04842.1	2.44	t / c	|EMB2761 | threonyl-tRNA synthetase, putative
682	AT3G51140.1	2.43	t / c	| | Protein of unknown function (DUF3353) | c
757	AT4G34090.2	2.43	t / c	| | unknown protein; FUNCTIONS IN: molecular_
908	AT5G22330.1	2.43	t / c	|ATTIP49A, RIN1 | P-loop containing nucleosid
1606	ATCG00420.1	2.43	t / c	|NDHJ | NADH dehydrogenase subunit J | chrC:4
177	AT2G21660.1	2.42	t / c	|ATGRP7, CCR2, GR-RBP7, GRP7 | cold, circadia
825	ATCG00830.1 (+1)	2.42	t / c	|RPL2.1 | ribosomal protein L2 | chrC:84337-8
960	AT5G19620.1	2.42	t / c	|EMB213, OEP80, ATOEP80, TOC75 | outer envelo
1329	AT1G07250.1	2.42	t / c	|UGT71C4 | UDP-glucosyl transferase 71C4 | ch
1516	AT1G45474.1 (+1)	2.42	t / c	|LHCA5 | photosystem I light harvesting compl
1824	AT5G35590.1	2.42	t / c	|PAA1 | proteasome alpha subunit A1 | chr5:13
1829	ATCG00170.1	2.42	t / c	|RPOC2 | DNA-directed RNA polymerase family p
1965	AT4G24220.1 (+1)	2.42	t / c	|VEP1, AWI31 | NAD(P)-binding Rossmann-fold s
2030	AT3G09090.1 (+1)	2.42	t / c	|DEX1 | defective in exine formation protein
2056	AT2G45220.1	2.42	t / c	| | Plant invertase/pectin methylesterase inh
706	AT2G20920.1	2.42	t-unique	| | Protein of unknown function (DUF3353) | c
1278	AT3G50930.1	2.42	t-unique	|BCS1 | cytochrome BC1 synthesis | chr3:18929
92	AT1G50250.1	2.41	t / c	|FTSH1 | FTSH protease 1 | chr1:18614398-1861
159	ATCG00500.1	2.41	t / c	|ACCD | acetyl-CoA carboxylase carboxyl trans

	Accession.Number	log2tc	label	Description
229	AT1G78570.1	2.39	t / c	|RHM1, ROL1, ATRHM1 | rhamnose biosynthesis 1
244	AT1G76160.1	2.39	t / c	|sks5 | SKU5 similar 5 | chr1:28578211-285810
856	AT4G28470.1	2.39	t / c	|RPN1B, ATRPN1B | 26S proteasome regulatory s
914	AT3G53180.1	2.39	t / c	| | glutamate-ammonia ligases;catalytics;glut
984	AT3G09840.1	2.39	t / c	|CDC48, ATCDC48, CDC48A | cell division cycle
1307	AT2G24180.1	2.39	t / c	|CYP71B6 | cytochrome p450 71b6 | chr2:102818
1813	AT1G21270.1	2.39	t / c	|WAK2 | wall-associated kinase 2 | chr1:74449
487	AT1G49970.1	2.37	t / c	|CLPR1, NCLPP5, SVR2 | CLP protease proteolyt
529	AT5G66680.1	2.37	t / c	|DGL1 | dolichyl-diphosphooligosaccharide-pro
1656	AT2G37710.1	2.37	t / c	|RLK | receptor lectin kinase | chr2:15814934
70	AT5G42270.1	2.36	t / c	|VAR1, FTSH5 | FtsH extracellular protease fa
1250	AT5G42240.1	2.36	t / c	|scpl42 | serine carboxypeptidase-like 42 | c
446	AT1G61790.1	2.32	t-unique	| | Oligosaccharyltransferase complex/magnesi
1228	AT1G10670.1 (+2)	2.32	t-unique	|ACLA-1 | ATP-citrate lyase A-1 | chr1:353578
1239	AT1G21630.1	2.32	t-unique	| | Calcium-binding EF hand family protein |
506	AT2G18710.1	2.32	t / c	|SCY1 | SECY homolog 1 | chr2:8112231-8114452
723	AT2G36390.1	2.32	t / c	|SBE2.1, BE3 | starch branching enzyme 2.1 |
753	AT3G52140.2 (+1)	2.32	t / c	| | tetratricopeptide repeat (TPR)-containing
755	AT3G13330.1	2.32	t / c	|PA200 | proteasome activating protein 200 |
771	ATCG00710.1	2.32	t / c	|PSBH | photosystem II reaction center protei
789	AT1G04530.1	2.32	t / c	|TPR4 | Tetratricopeptide repeat (TPR)-like s
802	AT5G45510.2	2.32	t / c	| | Leucine-rich repeat (LRR) family protein
835	AT4G17090.1	2.32	t / c	|CT-BMY, BAM3, BMY8 | chloroplast beta-amylas
981	AT3G10690.1	2.32	t / c	|GYRA | DNA GYRASE A | chr3:3339612-3346243 R
1026	AT5G10690.1	2.32	t / c	| | pentatricopeptide (PPR) repeat-containing
1050	AT3G01300.1	2.32	t / c	| | Protein kinase superfamily protein | chr3
1073	AT3G51420.1	2.32	t / c	|SSL4, ATSSL4 | strictosidine synthase-like 4
1125	AT5G26360.1	2.32	t / c	| | TCP-1/cpn60 chaperonin family protein | c
1556	AT5G52520.1	2.32	t / c	|OVA6, PRORS1 | Class II aaRS and biotin synt
1634	ATCG00820.1	2.32	t / c	|RPS19 | ribosomal protein S19 | chrC:84005-8

	Accession.Number	log2tc	label	Description
1711	AT5G09900.2	2.32	t / c	|EMB2107, RPN5A, MSA | 26S proteasome regulat
1898	AT1G62180.1	2.32	t / c	|APR2, APSR, PRH43, PRH, ATAPR2 | 5'adenylylp
1974	AT3G49560.1	2.32	t / c	| | Mitochondrial import inner membrane trans
2000	AT5G14120.1	2.32	t / c	| | Major facilitator superfamily protein | c
73	AT5G14040.1	2.31	t / c	|PHT3;1 | phosphate transporter 3;1 | chr5:45
123	AT3G05530.1	2.31	t / c	|RPT5A, ATS6A.2 | regulatory particle triple-
210	ATCG01110.1	2.31	t / c	|NDHH | NAD(P)H dehydrogenase subunit H | chr
523	AT4G29040.1	2.31	t / c	|RPT2a | regulatory particle AAA-ATPase 2A |
235	AT4G03280.1	2.30	t / c	|PETC, PGR1 | photosynthetic electron transfe
71	AT5G19770.1 (+1)	2.29	t / c	|TUA3 | tubulin alpha-3 | chr5:6682761-668447
496	AT1G08520.1	2.29	t / c	|ALB1, ALB-1V, V157, PDE166, CHLD | ALBINA 1
756	AT2G22360.1	2.29	t / c	| | DNAJ heat shock family protein | chr2:949
192	AT5G12470.1	2.28	t / c	| | Protein of unknown function (DUF3411) | c
505	AT5G03940.1	2.28	t / c	|FFC, 54CP, CPSRP54, SRP54CP | chloroplast si
42	AT1G29930.1	2.27	t / c	|CAB1, AB140, CAB140, LHCB1.3 | chlorophyll A
1635	ATCG00670.1	2.27	t / c	|CLPP1, PCLPP | plastid-encoded CLP P | chrC:
1728	AT3G28710.1	2.27	t / c	| | ATPase, V0/A0 complex, subunit C/D | chr3
60	AT4G10340.1	2.26	t / c	|LHCB5 | light harvesting complex of photosys
643	AT1G53500.1	2.26	t / c	|MUM4, RHM2, ATRHM2, ATMUM4 | NAD-dependent e
1661	AT4G00400.1	2.25	t / c	|GPAT8, AtGPAT8 | glycerol-3-phosphate acyltr
1805	AT1G09795.1	2.25	t / c	|ATATP-PRT2, HISN1B, ATP-PRT2 | ATP phosphori
1819	AT1G64710.1	2.25	t / c	| | GroES-like zinc-binding dehydrogenase fam
520	AT2G33040.1	2.23	t / c	|ATP3 | gamma subunit of Mt ATP synthase | ch
647	AT4G24570.1	2.22	t-unique	|DIC2 | dicarboxylate carrier 2 | chr4:126865
1959	AT4G30610.1	2.22	t-unique	|BRS1, SCPL24 | alpha/beta-Hydrolases superfa
920	AT2G26910.1	2.22	t / c	|PDR4, ATPDR4 | pleiotropic drug resistance 4
1509	AT1G68560.1	2.22	t / c	|ATXYL1, XYL1, TRG1 | alpha-xylosidase 1 | ch
1836	AT5G14950.1	2.22	t / c	|GMII, ATGMII | golgi alpha-mannosidase II |
1923	AT1G14670.1	2.22	t / c	| | Endomembrane protein 70 protein family |
1952	AT5G59420.1	2.22	t / c	|ORP3C | OSBP(oxysterol binding protein)-rela

	Accession.Number	log2tc	label	Description
1960	AT4G34950.1	2.22	t / c	| | Major facilitator superfamily protein | c
176	AT3G02090.1	2.21	t / c	|MPPBETA | Insulinase (Peptidase family M16)
420	AT2G26250.1	2.21	t / c	|FDH, KCS10 | 3-ketoacyl-CoA synthase 10 | ch
8	AT2G39730.1	2.20	t / c	|RCA | rubisco activase | chr2:16570951-16573
386	AT1G16300.1	2.20	t / c	|GAPCP-2 | glyceraldehyde-3-phosphate dehydro
632	AT1G32500.1	2.20	t / c	|ATNAP6, NAP6 | non-intrinsic ABC protein 6 |
637	AT1G59610.1	2.20	t / c	|ADL3, CF1, DRP2B, DL3 | dynamin-like 3 | chr
1343	AT5G49030.3	2.20	t / c	|OVA2 | tRNA synthetase class I (I, L, M and
1511	AT5G22800.1	2.20	t / c	|EMB86, EMB1030, EMB263 | Alanyl-tRNA synthet
384	AT5G54270.1	2.19	t / c	|LHCB3, LHCB3*1 | light-harvesting chlorophyl
860	AT4G28080.1	2.19	t / c	| | Tetratricopeptide repeat (TPR)-like super
47	AT1G15820.1	2.18	t / c	|LHCB6, CP24 | light harvesting complex photo
59	AT1G61520.1 (+1)	2.18	t / c	|LHCA3 | photosystem I light harvesting compl
72	AT2G33800.1	2.18	t / c	| | Ribosomal protein S5 family protein | chr
85	AT5G23060.1	2.18	t / c	|CaS | calcium sensing receptor | chr5:773676
410	AT3G63410.1	2.18	t / c	|APG1, VTE3, IEP37, E37 | S-adenosyl-L-methio
535	AT1G10290.1	2.17	t / c	|ADL6, DRP2A | dynamin-like protein 6 | chr1:
1095	AT2G01350.1	2.17	t / c	|QPT | quinolinate phoshoribosyltransferase |
1381	AT4G00360.1	2.17	t / c	|CYP86A2, ATT1 | cytochrome P450, family 86,
1391	AT1G50430.1 (+1)	2.17	t / c	|DWF5, PA, LE, ST7R, 7RED | Ergosterol biosyn
1707	AT2G28800.1 (+1)	2.17	t / c	|ALB3 | 63 kDa inner membrane family protein
1738	AT4G27700.1	2.17	t / c	| | Rhodanese/Cell cycle control phosphatase
322	AT5G23540.1	2.16	t / c	| | Mov34/MPN/PAD-1 family protein | chr5:793
419	AT5G19550.1	2.16	t / c	|ASP2, AAT2 | aspartate aminotransferase 2 |
125	AT4G35250.1	2.15	t / c	| | NAD(P)-binding Rossmann-fold superfamily
574	AT5G62790.1	2.15	t / c	|DXR, PDE129 | 1-deoxy-D-xylulose 5-phosphate
1596	AT1G65260.1	2.15	t / c	|PTAC4, VIPP1 | plastid transcriptionally act
36	AT1G49240.1	2.14	t / c	|ACT8 | actin 8 | chr1:18216539-18217947 FORW
57	AT1G04820.1 (+1)	2.14	t / c	|TUA4, TOR2 | tubulin alpha-4 chain | chr1:13
443	AT1G78630.1	2.14	t / c	|emb1473 | Ribosomal protein L13 family prote

	Accession.Number	log2tc	label	Description
222	AT1G62020.1	2.13	t / c	| | Coatomer, alpha subunit | chr1:22919814-2
406	AT1G62750.1	2.13	t / c	|ATSCO1, ATSCO1/CPEF-G, SCO1 | Translation el
838	AT1G48350.1	2.13	t / c	| | Ribosomal L18p/L5e family protein | chr1:
1397	AT1G53440.1	2.13	t / c	| | Leucine-rich repeat transmembrane protein
544	AT3G58750.1	2.12	t / c	|CSY2 | citrate synthase 2 | chr3:21724564-21
567	AT5G16660.2	2.12	t / c	| | unknown protein; FUNCTIONS IN: molecular_
687	AT2G33450.1	2.12	t / c	| | Ribosomal L28 family | chr2:14173620-1417
689	AT2G30620.1	2.12	t / c	| | winged-helix DNA-binding transcription fa
891	AT5G51820.1	2.12	t / c	|PGM, ATPGMP, PGM1, STF1 | phosphoglucomutase
1003	AT1G31800.1	2.12	t / c	|CYP97A3, LUT5 | cytochrome P450, family 97,
1107	AT5G61810.1	2.12	t / c	| | Mitochondrial substrate carrier family pr
1352	AT5G23300.1	2.12	t / c	|PYRD | pyrimidine d | chr5:7847792-7850243 R
1732	AT1G51100.1	2.12	t / c	| | unknown protein; FUNCTIONS IN: molecular_
1778	AT3G63520.1	2.12	t / c	|CCD1, ATCCD1, ATNCED1, NCED1 | carotenoid cl
1811	AT4G20830.1	2.12	t / c	| | FAD-binding Berberine family protein | ch
1828	AT4G32300.1	2.12	t / c	|SD2-5 | S-domain-2 5 | chr4:15599970-1560243
1843	AT1G64900.1	2.12	t / c	|CYP89A2, CYP89 | cytochrome P450, family 89,
1877	AT2G40060.1	2.12	t / c	| | Clathrin light chain protein | chr2:16726
1906	AT5G63510.2	2.12	t / c	|GAMMA CAL1 | gamma carbonic anhydrase like 1
1929	AT4G00026.1	2.12	t / c	| | FUNCTIONS IN: molecular_function unknown;
1961	AT3G03100.1	2.12	t / c	| | NADH:ubiquinone oxidoreductase, 17.2kDa s
1996	AT2G28430.1	2.12	t / c	| | unknown protein; Has 28 Blast hits to 28
1958	AT5G59250.1	2.12	t-unique	| | Major facilitator superfamily protein | c
2106	AT3G25070.1	2.12	t-unique	|RIN4 | RPM1 interacting protein 4 | chr3:913
65	AT1G09340.1	2.11	t / c	|CRB, CSP41B, HIP1.3 | chloroplast RNA bindin
396	AT1G75350.1	2.11	t / c	|emb2184 | Ribosomal protein L31 | chr1:28272
577	ATCG01090.1	2.10	t / c	|NDHI | NADPH dehydrogenases | chrC:119244-11
622	AT1G04810.1	2.10	t / c	| | 26S proteasome regulatory complex, non-AT
1569	AT1G04270.1	2.10	t / c	|RPS15 | cytosolic ribosomal protein S15 | ch
1310	AT3G44330.1	2.09	t / c	| | INVOLVED IN: protein processing; LOCATED

	Accession.Number	log2tc	label	Description
1755	AT2G32060.1 (+2)	2.09	t / c	| | Ribosomal protein L7Ae/L30e/S12e/Gadd45 f
1792	AT5G58100.1	2.09	t / c	| | unknown protein; INVOLVED IN: pollen exin
1888	AT3G61650.1	2.09	t / c	|TUBG1 | gamma-tubulin | chr3:22812601-228150
250	AT4G39260.1	2.08	t / c	|CCR1, ATGRP8, GR-RBP8, GRP8 | cold, circadia
84	AT1G74470.1	2.07	t / c	| | Pyridine nucleotide-disulphide oxidoreduc
106	AT4G27440.1 (+1)	2.07	t / c	|PORB | protochlorophyllide oxidoreductase B
234	AT3G56940.1	2.07	t / c	|CRD1, CHL27, ACSF | dicarboxylate diiron pro
262	AT3G59780.1	2.07	t / c	| | Rhodanese/Cell cycle control phosphatase
370	AT4G30010.1	2.07	t / c	| | unknown protein; FUNCTIONS IN: molecular_
627	AT1G05190.1	2.07	t / c	|emb2394 | Ribosomal protein L6 family | chr1
1551	AT3G05560.1 (+2)	2.07	t / c	| | Ribosomal L22e protein family | chr3:1614
1736	AT2G48070.1 (+1)	2.07	t / c	|RPH1 | resistance to phytophthora 1 | chr2:1
664	AT5G27770.1	2.06	t / c	| | Ribosomal L22e protein family | chr5:9836
745	AT2G21580.1 (+1)	2.06	t / c	| | Ribosomal protein S25 family protein | ch
1094	AT1G71220.1	2.06	t / c	|EBS1, UGGT, PSL2 | UDP-glucose:glycoprotein
1600	AT1G53430.1 (+1)	2.06	t / c	| | Leucine-rich repeat transmembrane protein
189	AT1G72370.1 (+1)	2.05	t / c	|P40, AP40, RP40, RPSAA | 40s ribosomal prote
495	AT3G62530.1	2.05	t / c	| | ARM repeat superfamily protein | chr3:231
1655	AT4G12830.1	2.05	t / c	| | alpha/beta-Hydrolases superfamily protein
147	AT5G54770.1	2.04	t / c	|THI1, TZ, THI4 | thiazole biosynthetic enzym
168	AT2G19940.1 (+1)	2.04	t / c	| | oxidoreductases, acting on the aldehyde o
312	AT2G42210.2	2.04	t / c	|ATOEP16-3, OEP16-3 | Mitochondrial import in
398	AT5G47210.1	2.04	t / c	| | Hyaluronan / mRNA binding family | chr5:1
503	AT4G30950.1	2.04	t / c	|FAD6, FADC, SFD4 | fatty acid desaturase 6 |
508	AT3G61820.1	2.04	t / c	| | Eukaryotic aspartyl protease family prote
1604	AT1G70770.1 (+1)	2.04	t / c	| | Protein of unknown function DUF2359, tran
41	AT3G12110.1	2.02	t / c	|ACT11 | actin-11 | chr3:3858116-3859609 FORW
294	AT1G52510.1	2.02	t / c	| | alpha/beta-Hydrolases superfamily protein
49	AT2G37620.1 (+2)	2.01	t / c	|ACT1, AAc1 | actin 1 | chr2:15779761-1578124
136	AT5G50850.1	2.01	t / c	|MAB1 | Transketolase family protein | chr5:2

	Accession.Number	log2tc	label	Description
182	AT3G08530.1	2.01	t / c	| | Clathrin, heavy chain | chr3:2587171-2595
428	AT1G55670.1	2.01	t / c	|PSAG | photosystem I subunit G | chr1:208028
364	AT2G38540.1	2.00	t / c	|LP1, LTP1, ATLTP1 | lipid transfer protein 1
399	AT2G33150.1	2.00	t / c	|PKT3, PED1, KAT2 | peroxisomal 3-ketoacyl-Co
578	AT4G21150.1 (+1)	2.00	t / c	|HAP6 | ribophorin II (RPN2) family protein |
728	AT2G31040.1	2.00	t / c	| | ATP synthase protein I -related | chr2:13
808	AT1G63970.1 (+1)	2.00	t / c	|ISPF, MECPS | isoprenoid F | chr1:23738923-2
830	AT5G08650.1	2.00	t / c	| | Small GTP-binding protein | chr5:2806533-
881	AT3G48560.1	2.00	t / c	|CSR1, ALS, AHAS, TZP5, IMR1 | chlorsulfuron/
903	ATCG00190.1	2.00	t / c	|RPOB | RNA polymerase subunit beta | chrC:23
923	AT4G28710.1	2.00	t / c	|XIH, ATXIH | Myosin family protein with Dil
980	AT5G35970.1	2.00	t / c	| | P-loop containing nucleoside triphosphate
1001	AT3G59110.1	2.00	t / c	| | Protein kinase superfamily protein | chr3
1015	AT2G35800.1	2.00	t / c	| | mitochondrial substrate carrier family pr
1045	AT5G67560.1	2.00	t / c	|ATARLA1D, ARLA1D | ADP-ribosylation factor-l
1192	AT3G61470.1	2.00	t / c	|LHCA2 | photosystem I light harvesting compl
1316	AT3G42170.1	2.00	t / c	| | BED zinc finger ;hAT family dimerisation
1331	AT3G17970.1	2.00	t / c	|atToc64-III, TOC64-III | translocon at the o
1353	AT3G07700.1 (+2)	2.00	t / c	| | Protein kinase superfamily protein | chr3
1371	AT5G11040.1	2.00	t / c	|TRS120, AtTRS120 | TRS120 | chr5:3495332-350
1426	AT2G25870.1	2.00	t / c	| | haloacid dehalogenase-like hydrolase fami
1446	AT5G64860.1	2.00	t / c	|DPE1 | disproportionating enzyme | chr5:2592
1646	AT1G71695.1	2.00	t / c	| | Peroxidase superfamily protein | chr1:269
1686	AT5G36230.1	2.00	t / c	| | ARM repeat superfamily protein | chr5:142
1709	AT5G67385.1	2.00	t / c	| | Phototropic-responsive NPH3 family protei
1800	AT1G29790.1 (+1)	2.00	t / c	| | S-adenosyl-L-methionine-dependent methylt
1837	AT1G06000.1	2.00	t / c	| | UDP-Glycosyltransferase superfamily prote
1874	AT5G61910.4	2.00	t / c	| | DCD (Development and Cell Death) domain p
1894	AT5G23140.1	2.00	t-unique	|CLPP2, NCLPP7 | nuclear-encoded CLP protease
1942	AT4G24550.2	2.00	t / c	| | Clathrin adaptor complexes medium subunit

	Accession.Number	log2tc	label	Description
1993	AT3G15000.1	2.00	t-unique	| | cobalt ion binding | chr3:5050321-5052121
2035	AT3G24530.1	2.00	t / c	| | AAA-type ATPase family protein / ankyrin
2038	AT3G48140.1	2.00	t-unique	| | B12D protein | chr3:17778471-17779299 FOR
2089	AT3G04210.1	2.00	t / c	| | Disease resistance protein (TIR-NBS class
2102	AT4G04570.1	2.00	t / c	|CRK40 | cysteine-rich RLK (RECEPTOR-like pro
2172	AT3G05000.1	2.00	t-unique	| | Transport protein particle (TRAPP) compon
1143	AT4G28740.1	1.87	t-unique	| | FUNCTIONS IN: molecular_function unknown;
1880	AT1G53210.1	1.74	t-unique	| | sodium/calcium exchanger family protein /
2022	AT4G16450.2 (+1)	1.74	t-unique	| | unknown protein; FUNCTIONS IN: molecular_
851	AT3G21200.1	1.58	t-unique	|PGR7 | proton gradient regulation 7 | chr3:7
1410	AT1G32220.1	1.58	t-unique	| | NAD(P)-binding Rossmann-fold superfamily
1449	AT5G14420.1 (+3)	1.58	t-unique	|RGLG2 | RING domain ligase2 | chr5:4648355-4
2029	ATCG00740.1	1.58	t-unique	|RPOA | RNA polymerase subunit alpha | chrC:7
2130	AT1G02120.1	1.58	t-unique	|VAD1 | GRAM domain family protein | chr1:395
2201	AT1G79390.1	1.58	t-unique	| | unknown protein; Has 30201 Blast hits to
2219	AT3G26935.1	1.58	t-unique	| | DHHC-type zinc finger family protein | ch
2225	AT3G62550.1	1.58	t-unique	| | Adenine nucleotide alpha hydrolases-like
2292	AT2G03820.1	1.58	t-unique	| | nonsense-mediated mRNA decay NMD3 family
266	AT4G08950.1	1.42	t-unique	|EXO | Phosphate-responsive 1 family protein
714	AT3G28220.1	1.42	t-unique	| | TRAF-like family protein | chr3:10524420-
2016	AT1G31410.1	1.42	t-unique	| | putrescine-binding periplasmic protein-re
2049	AT1G67785.1	1.42	t-unique	| | unknown protein; Has 30 Blast hits to 30
2239	AT3G51510.1	1.42	t-unique	| | unknown protein; FUNCTIONS IN: molecular_
883	AT5G57020.1	1.22	t-unique	|NMT1, ATNMT1 | myristoyl-CoA:protein N-myris
1016	AT2G20050.1 (+1)	1.22	t-unique	| | protein serine/threonine phosphatases;pro
1206	AT1G01100.1 (+2)	1.22	t-unique	| | 60S acidic ribosomal protein family | chr
1328	AT3G25760.1	1.22	t-unique	|AOC1, ERD12 | allene oxide cyclase 1 | chr3:
1370	AT1G05350.1	1.22	t-unique	| | NAD(P)-binding Rossmann-fold superfamily
1423	AT5G23670.1 (+1)	1.22	t-unique	|LCB2 | long chain base2 | chr5:7981889-79850
1850	AT1G28290.1 (+1)	1.22	t-unique	|AGP31 | arabinogalactan protein 31 | chr1:98

	Accession.Number	log2tc	label	Description
1924	AT2G15430.1	1.22	t-unique	|RBP36A, RPB35.5A, NRPB3, NRPD3, NRPE3A | DNA
2113	AT3G15680.1	1.22	t-unique	| | Ran BP2/NZF zinc finger-like superfamily
2155	AT1G72340.1	1.22	t-unique	| | NagB/RpiA/CoA transferase-like superfamil
2187	AT4G40050.1	1.22	t-unique	| | Protein of unknown function (DUF3550/UPF0
2256	AT2G32160.2 (+1)	1.22	t-unique	| | S-adenosyl-L-methionine-dependent methylt
2282	AT2G27290.1	1.22	t-unique	| | Protein of unknown function (DUF1279) | c
1332	AT5G11840.1	1.00	t-unique	| | Protein of unknown function (DUF1230) | c
1756	AT2G20530.1 (+1)	1.00	t-unique	|ATPHB6, PHB6 | prohibitin 6 | chr2:8842300-8
1936	AT5G53650.1	1.00	t-unique	| | unknown protein; FUNCTIONS IN: molecular_
1955	AT4G26070.2 (+1)	1.00	t-unique	|MEK1, NMAPKK, ATMEK1, MKK1 | MAP kinase/ ERK
2111	AT4G12390.1	1.00	t-unique	|PME1 | pectin methylesterase inhibitor 1 | c
2114	AT1G42960.1	1.00	t-unique	| | expressed protein localized to the inner
2313	AT3G19810.1	1.00	t-unique	| | Protein of unknown function (DUF177) | ch
809	AT4G39090.1	0.74	t-unique	|RD19, RD19A | Papain family cysteine proteas
1182	AT5G54110.1	0.74	t-unique	|ATMAMI, MAMI | membrane-associated mannitol-
1202	AT5G47890.1	0.74	t-unique	| | NADH-ubiquinone oxidoreductase B8 subunit
1288	AT5G51010.1	0.74	t-unique	| | Rubredoxin-like superfamily protein | chr
1429	AT1G10700.1	0.74	t-unique	|PRS3 | phosphoribosyl pyrophosphate (PRPP) s
1881	AT3G43980.1 (+2)	0.74	t-unique	| | Ribosomal protein S14p/S29e family protei
2144	AT3G44720.1	0.74	t-unique	|ADT4 | arogenate dehydratase 4 | chr3:162717
2192	AT4G26500.1	0.74	t-unique	|EMB1374, CPSUFE, ATSUFE, SUFE1 | chloroplast
2205	AT3G12260.1	0.74	t-unique	| | LYR family of Fe/S cluster biogenesis pro
875	AT5G60980.2	0.42	t-unique	| | Nuclear transport factor 2 (NTF2) family
1145	AT3G55430.1	0.42	t-unique	| | O-Glycosyl hydrolases family 17 protein |
1355	AT1G34130.1	0.42	t-unique	|STT3B | staurosporin and temperature sensiti
1402	AT2G20230.1	0.42	t-unique	| | Tetraspanin family protein | chr2:8725762
2163	AT3G59920.1	0.42	t-unique	|ATGDI2, GDI2 | RAB GDP dissociation inhibito
2170	AT5G45420.1	0.42	t-unique	| | Duplicated homeodomain-like superfamily p
614	AT3G09260.1	0.00	c-unique	|PYK10, PSR3.1, BGLU23, LEB | Glycosyl hydrol
1975	AT1G71710.1	0.00	t-unique	| | DNAse I-like superfamily protein | chr1:2

	Accession.Number	log2tc	label	Description
2063	AT5G67590.1	0.00	t-unique	|FRO1 | NADH-ubiquinone oxidoreductase-relate
2123	AT5G17770.1	0.00	t-unique	|ATCBR, CBR1, CBR | NADH:cytochrome B5 reduct
2299	AT1G50600.1	0.00	t-unique	|SCL5 | scarecrow-like 5 | chr1:18737398-1873
2327	AT4G34150.1	0.00	c-unique	| | Calcium-dependent lipid-binding (CaLB dom
1499	AT5G06690.1	-0.42	c-unique	|WCRKC1 | WCRKC thioredoxin 1 | chr5:2060651-
2244	AT2G23600.1	-0.74	c-unique	|ACL, ATMES2, MES2, ATME8, ME8 | acetone-cyan
2263	AT3G25620.2	-0.74	c-unique	| | ABC-2 type transporter family protein | c
2332	AT5G07572.1	-0.74	c-unique	| | unknown protein; FUNCTIONS IN: molecular_
899	AT1G04690.1	-1.22	c-unique	|KAB1, KV-BETA1 | potassium channel beta subu
2084	AT5G13420.1	-1.22	c-unique	| | Aldolase-type TIM barrel family protein |
2161	AT4G00620.1	-1.22	c-unique	| | Amino acid dehydrogenase family protein |
1195	AT4G21180.1	-1.42	c-unique	|ATERDJ2B | DnaJ / Sec63 Brl domains-containi
1984	AT3G12290.1	-1.58	c-unique	| | Amino acid dehydrogenase family protein |
1910	AT1G12850.1	-1.74	c-unique	| | Phosphoglycerate mutase family protein |
1088	AT1G21750.1	-2.00	t / c	|ATPDIL1-1, ATPDI5, PDI5, PDIL1-1 | PDI-like
1618	AT1G52600.1	-2.00	c-unique	| | Peptidase S24/S26A/S26B/S26C family prote
1588	AT3G57290.1	-2.12	t / c	|EIF3E, TIF3E1, ATEIF3E-1, INT-6, ATINT6, INT
2061	AT1G63810.1	-2.42	c-unique	| | CONTAINS InterPro DOMAIN/s: Nrap protein
382	AT2G14110.1	-2.51	t / c	| | Haloacid dehalogenase-like hydrolase (HAD
1421	AT1G79940.1 (+2)	-3.06	t / c	|ATERDJ2A | DnaJ / Sec63 Brl domains-containi
1719	AT1G29880.1	-3.12	t / c	| | glycyl-tRNA synthetase / glycine–tRNA li
1525	AT2G17290.1	-3.99	t / c	|CPK6, ATCDPK3, ATCPK6 | Calcium-dependent pr

	Accession.Number	log2tc	label	Description
121	AT4G35310.1	-4.13	t / c	|CPK5, ATCPK5 | calmodulin-domain protein kin
183	AT1G28380.1	-4.47	t / c	|NSL1 | MAC/Perforin domain-containing protei

2.6 Proteins of interest

Here we find the protein accessions important for this project.

	x
MRK1	AT3G63260

```
## MRK1
##  427
```

2.6.1 Working with the results

We work with the individual items of 'list_df', each holding a result of the pair comparison as defined earlier. We show the original data after and before the imputation to document the treatment of missing values.

```
## [1] "Pair No.: 1 CPK28 (m22) /// NSL1, PM-GFP (m22)"
## [2] "Pair No.: 2 CPK5 (m22) /// NSL1, PM-GFP (m22)"
## [3] "Pair No.: 3 CPK28 (m22) /// CPK5 (m22)"
```

2.6.2 The selected proteins, imputed values

After the filters using our thresholds of acceptance for fold change and reproducibility were applied.

[1] "Pair No. 1 : CPK28 (m22) /// NSL1, PM-GFP (m22)"

	log2tc	m28_22	m28_22.1	m28_22.2	mNSL_22	mNSL_22.1	mNSL_22.2	mPMG_22	mPMG_22.1
MRK1	3.97	18	17	12	1	1	1	1	1

[1] "Pair No. 2 : CPK5 (m22) /// NSL1, PM-GFP (m22)"

The following accession:

AT3G63260

Were not detected in the comparison:

[1] "Pair No. 3 : CPK28 (m22) /// CPK5 (m22)"

	log2tc	m28_22	m28_22.1	m28_22.2	m5_22	m5_22.1	m5_22.2
MRK1	3.23	18	17	12	1	2	2

2.6.3 The selected proteins, original values

… are shown here to check the imputation method results and how the filters applied affected the proteins of interest.

[1] "Pair No. 1 : CPK28 (m22) /// NSL1, PM-GFP (m22)"

	m28_22	m28_22.1	m28_22.2	mNSL_22	mNSL_22.1	mNSL_22.2	mPMG_22	mPMG_22.1
MRK1	18	17	12	0	1	NA	NA	NA

[1] "Pair No. 2 : CPK5 (m22) /// NSL1, PM-GFP (m22)"

	m5_22	m5_22.1	m5_22.2	mNSL_22	mNSL_22.1	mNSL_22.2	mPMG_22	mPMG_22.1
MRK1	0	2	2	0	1	NA	NA	NA

[1] "Pair No. 3 : CPK28 (m22) /// CPK5 (m22)"

	m28_22	m28_22.1	m28_22.2	m5_22	m5_22.1	m5_22.2
MRK1	18	17	12	0	2	2

2.7 Comparison of fold changes of the pairs

The fold changes (log2) can be compared among the different ***pairs*** (treatment/controls) as shown in the following table.
Only "m22" pairs are used.

The table is sorted by the 'pair_3', thus CPK28 interacting candidates should be on top, CPK5 interacting protein candidates at the bottom. The other pairs, CPK28 vs. control and CPK5 vs. control distribute along this division. The pattern we observe let us assess the differences in CPK28 and CPK5 interacting proteins.

```
## Pair No. 1 :  CPK28 (m22) /// NSL1, PM-GFP (m22)
## Pair No. 2 :  CPK5 (m22) /// NSL1, PM-GFP (m22)
## Pair No. 3 :  CPK28 (m22) /// CPK5 (m22)
```

	Accession.Number	Description	pair_1	pair_2	pair_3
643	AT4G36070.2	|CPK18 | calcium-dependent protein	5.0	NA	6.5
646	AT2G20140.1	| | AAA-type ATPase family protein	5.3	NA	5.3
7	AT2G17890.1	|CPK16 | calcium-dependent protein	5.6	NA	5.0
621	AT4G28520.1	|CRU3, CRC | cruciferin 3 | chr4:14	5.1	NA	4.7
412	AT5G46580.1	| | pentatricopeptide (PPR) repeat-	4.7	NA	4.7
1	AT5G66210.1 (+1)	|CPK28 | calcium-dependent protein	5.4	NA	4.6
362	AT1G17840.1	|WBC11, ABCG11, DSO, COF1, ATWBC11	4.6	NA	4.6

	Accession.Number	Description	pair_1	pair_2	pair_3
53	AT4G30190.1	|AHA2, PMA2, HA2 | H(+)-ATPase 2 |	5.2	NA	4.5
223	AT4G35470.1	|PIRL4 | plant intracellular ras gr	4.9	NA	4.5
276	AT1G01790.1	|KEA1, ATKEA1 | K+ efflux antiporte	5.2	NA	4.5
541	AT2G46520.1	| | cellular apoptosis susceptibili	4.4	NA	4.4
164	AT2G22500.1	|UCP5, ATPUMP5, DIC1 | uncoupling p	4.4	NA	4.4
127	AT3G59350.1 (+1)	| | Protein kinase superfamily prot	4.4	NA	4.4
222	AT1G79050.1	| | recA DNA recombination family p	4.3	NA	4.3
632	AT5G20490.1	|XIK, ATXIK, XI-17 | Myosin family	4.2	NA	4.2
490	AT1G30360.1	|ERD4 | Early-responsive to dehydra	4.2	NA	4.2
523	AT2G41560.1	|ACA4 | autoinhibited Ca(2+)-ATPase	4.3	NA	4.2
496	AT5G57110.1 (+1)	|ACA8, AT-ACA8 | autoinhibited Ca2+	4.1	NA	4.1
532	AT1G18270.3	| | ketose-bisphosphate aldolase cl	4.1	NA	4.1
672	AT1G05150.1	| | Calcium-binding tetratricopepti	4.1	NA	4.1
242	AT1G06700.1 (+1)	| | Protein kinase superfamily prot	4.0	NA	4.0
37	AT5G13490.1 (+1)	|AAC2 | ADP/ATP carrier 2 | chr5:43	6.6	2.6	4.0
431	AT1G79600.1	| | Protein kinase superfamily prot	3.5	NA	3.9
485	AT1G01960.1	|EDA10 | SEC7-like guanine nucleoti	3.9	NA	3.9
344	AT4G19710.2	|AK-HSDH II, AK-HSDH | aspartate ki	3.9	NA	3.9
585	AT2G31880.1	|SOBIR1, EVR | Leucine-rich repeat	3.9	NA	3.9
71	AT3G52750.1	|FTSZ2-2 | Tubulin/FtsZ family prot	4.5	NA	3.9
48	AT2G18960.1	|AHA1, PMA, OST2, HA1 | H(+)-ATPase	5.5	NA	3.8
703	AT1G60780.1	|HAP13 | Clathrin adaptor complexes	3.8	NA	3.8
631	AT3G08510.1 (+1)	|ATPLC2, PLC2 | phospholipase C 2 |	5.5	NA	3.8
360	AT5G55610.1	| | unknown protein; LOCATED IN: mi	3.8	NA	3.8
509	AT3G43300.1	|ATMIN7, BEN1 | HOPM interactor 7 |	3.3	NA	3.8
215	AT4G10790.1	| | UBX domain-containing protein |	4.1	NA	3.7
649	AT3G14840.2	| | Leucine-rich repeat transmembra	4.5	NA	3.7
272	AT1G06410.1	|ATTPS7, TPS7, ATTPSA | trehalose-p	4.4	NA	3.7
417	AT1G03160.1	|FZL | FZO-like | chr1:761321-76605	3.7	NA	3.7
677	AT5G48880.2 (+1)	|PKT2, KAT5 | peroxisomal 3-keto-ac	3.6	NA	3.7

	Accession.Number	Description	pair_1	pair_2	pair_3
348	AT4G37925.1	|NDH-M | subunit NDH-M of NAD(P)H:p	2.8	NA	3.7
472	AT3G46970.1	|ATPHS2, PHS2 | alpha-glucan phosph	4.5	NA	3.7
645	AT1G02150.1	| | Tetratricopeptide repeat (TPR)-	4.6	NA	3.7
564	AT4G29900.1	|ACA10, CIF1, ATACA10 | autoinhibit	3.7	NA	3.7
638	AT1G30470.1	| | SIT4 phosphatase-associated fam	3.7	NA	3.7
478	AT5G38660.2	|APE1 | acclimation of photosynthes	4.1	NA	3.6
81	AT1G80480.1	|PTAC17 | plastid transcriptionally	4.1	NA	3.6
162	AT4G29130.1	|ATHXK1, GIN2, HXK1 | hexokinase 1	5.3	NA	3.6
184	AT1G54520.1	| | unknown protein; FUNCTIONS IN:	4.3	NA	3.6
470	AT3G29320.1	| | Glycosyl transferase, family 35	4.1	NA	3.6
552	AT1G31230.1	|AK-HSDH I, AK-HSDH | aspartate kin	3.6	NA	3.6
713	AT2G18730.1	|ATDGK3, DGK3 | diacylglycerol kina	3.6	NA	3.6
307	AT5G05010.1 (+1)	| | clathrin adaptor complexes medi	4.2	NA	3.6
256	AT4G31480.1 (+1)	| | Coatomer, beta subunit | chr4:1	5.1	NA	3.5
581	AT5G61020.1	|ECT3 | evolutionarily conserved C-	3.7	NA	3.5
390	AT4G25960.1	|PGP2 | P-glycoprotein 2 | chr4:131	3.5	NA	3.5
522	AT3G61050.1 (+1)	|NTMC2TYPE4, NTMC2T4 | Calcium-depe	3.3	NA	3.5
674	AT1G52360.1	| | Coatomer, beta’ subunit | chr1:	2.7	NA	3.5
354	AT1G13320.1 (+1)	|PP2AA3 | protein phosphatase 2A s	4.2	NA	3.5
497	AT3G25800.1	|PDF1, PR 65, PP2AA2 | protein phos	4.2	NA	3.5
264	AT3G01060.1	| | unknown protein; Has 640 Blast	3.5	NA	3.5
433	AT5G64940.1 (+1)	|ATATH13, ATH13, ATOSA1, OSA1 | ABC	3.5	NA	3.5
201	AT2G38040.1 (+1)	|CAC3 | acetyl Co-enzyme a carboxyl	3.6	NA	3.5
93	AT3G44110.1	|ATJ3, ATJ | DNAJ homologue 3 | chr	3.4	NA	3.5
133	AT1G64740.1	|TUA1 | alpha-1 tubulin | chr1:2405	3.7	NA	3.5
234	AT4G22690.1	|CYP706A1 | cytochrome P450, family	5.2	NA	3.5
318	AT5G52320.1	|CYP96A4 | cytochrome P450, family	3.5	NA	3.5
347	AT2G46820.1 (+1)	|PTAC8, TMP14, PSAP, PSI-P | photos	2.7	NA	3.5
544	AT5G23630.1	|PDR2, MIA | phosphate deficiency r	3.5	NA	3.5
655	AT4G23650.1	|CDPK6, CPK3 | calcium-dependent pr	4.2	NA	3.5

	Accession.Number	Description	pair_1	pair_2	pair_3
952	AT3G05590.1	|RPL18 | ribosomal protein L18 | ch	NA	NA	3.5
628	AT4G38630.1	|RPN10, MCB1, ATMCB1, MBP1 | regula	3.4	NA	3.4
480	AT5G66200.1	|ARO2 | armadillo repeat only 2 | c	3.4	NA	3.4
551	AT5G04130.1	|GYRB2 | DNA GYRASE B2 | chr5:11220	3.4	NA	3.4
616	AT4G24330.1	| | Protein of unknown function (DU	3.4	NA	3.4
278	AT1G74960.1 (+2)	|FAB1, KAS2, ATKAS2 | fatty acid bi	3.7	NA	3.4
290	AT2G35840.1 (+2)	| | Sucrose-6F-phosphate phosphohyd	2.8	NA	3.4
68	AT3G54890.1	|LHCA1 | photosystem I light harves	3.4	NA	3.4
623	AT5G27380.1	|GSH2, GSHB | glutathione synthetas	3.6	NA	3.4
302	AT1G06530.1	| | Tropomyosin-related | chr1:2001	3.4	NA	3.4
450	AT2G47450.1	|CAO, CPSRP43 | chloroplast signal	3.4	NA	3.4
558	AT1G51500.1	|CER5, D3, ABCG12, WBC12, ATWBC12 |	3.4	NA	3.4
679	AT5G06530.1 (+1)	| | ABC-2 type transporter family p	3.4	NA	3.4
847	AT3G22640.1	|PAP85 | cupin family protein | chr	3.4	NA	3.4
72	AT5G62670.1	|AHA11, HA11 | H(+)-ATPase 11 | chr	4.9	NA	3.4
420	AT5G17380.1	| | Thiamine pyrophosphate dependen	3.6	NA	3.4
154	AT3G54110.1	|ATPUMP1, UCP, PUMP1, ATUCP1, UCP1	4.1	NA	3.3
753	AT4G37200.1	|HCF164 | Thioredoxin superfamily p	3.3	NA	3.3
337	AT4G33090.1	|APM1, ATAPM1 | aminopeptidase M1 |	5.2	NA	3.3
573	AT5G42390.1	| | Insulinase (Peptidase family M1	2.8	NA	3.3
639	AT5G23860.1 (+1)	|TUB8 | tubulin beta 8 | chr5:80429	4.2	NA	3.3
324	AT3G07770.1	|Hsp89.1, AtHsp90.6, AtHsp90-6 | HE	2.9	NA	3.3
326	AT4G00630.1	|KEA2, ATKEA2 | K+ efflux antiporte	4.0	NA	3.3
397	AT1G29310.1	| | SecY protein transport family p	3.3	NA	3.3
462	AT5G64740.1	|CESA6, IXR2, E112, PRC1 | cellulos	3.3	NA	3.3
601	AT5G13430.1	| | Ubiquinol-cytochrome C reductas	2.8	NA	3.3
625	AT3G50950.1 (+1)	|ZAR1 | HOPZ-ACTIVATED RESISTANCE 1	4.3	NA	3.3
746	AT5G02890.1	| | HXXXD-type acyl-transferase fam	3.3	NA	3.3
747	AT4G30340.1	|ATDGK7, DGK7 | diacylglycerol kina	3.3	NA	3.3
755	AT2G34560.1	| | P-loop containing nucleoside tr	3.3	NA	3.3

	Accession.Number	Description	pair_1	pair_2	pair_3
85	AT5G65720.1	|ATNIFS1, NIFS1, NFS1, ATNFS1 | nit	4.2	NA	3.2
200	AT5G22060.1	|ATJ2, J2 | DNAJ homologue 2 | chr5	3.8	NA	3.2
232	AT3G63260.1	|ATMRK1 | Protein kinase superfamil	4.0	NA	3.2
377	AT1G59870.1	|PEN3, PDR8, ATPDR8, ABCG36, ATABCG	4.6	NA	3.2
719	AT1G34000.1	|OHP2 | one-helix protein 2 | chr1:	3.2	NA	3.2
736	AT5G58670.1	|ATPLC1, ATPLC, PLC1 | phospholipas	3.2	NA	3.2
769	AT1G64430.1 (+1)	| | Pentatricopeptide repeat (PPR)	3.2	NA	3.2
239	AT3G51820.1	|ATG4, G4, CHLG | UbiA prenyltransf	3.0	NA	3.2
251	AT5G03880.1	| | Thioredoxin family protein | ch	3.5	NA	3.2
546	AT5G43900.3	|MYA2 | myosin 2 | chr5:17657241-17	3.2	NA	3.2
560	AT3G28860.1	|ATMDR1, ATMDR11, PGP19, MDR11, MDR	3.2	NA	3.2
563	AT1G70320.1	|UPL2 | ubiquitin-protein ligase 2	3.2	NA	3.2
608	AT1G01220.1	|FKGP, AtFKGP | L-fucokinase/GDP-L-	3.2	NA	3.2
754	AT3G45190.1	| | SIT4 phosphatase-associated fam	3.2	NA	3.2
334	AT2G19860.1	|ATHXK2, HXK2 | hexokinase 2 | chr2	3.6	NA	3.2
376	AT3G53520.4	|UXS1 | UDP-glucuronic acid decarbo	3.4	NA	3.2
519	AT3G44340.1	|CEF | clone eighty-four | chr3:160	3.6	NA	3.2
684	AT5G13630.1	|GUN5, CCH, CHLH, CCH1, ABAR | magn	3.6	NA	3.2
249	AT2G41790.1	| | Insulinase (Peptidase family M1	3.9	NA	3.2
293	ATCG00430.1	|PSBG | photosystem II reaction cen	3.2	NA	3.2
507	AT4G16990.2	|RLM3 | disease resistance protein	3.2	NA	3.2
517	AT2G43950.1	|OEP37, ATOEP37 | chloroplast outer	3.2	NA	3.2
540	AT1G78915.1 (+2)	| | Tetratricopeptide repeat (TPR)-	3.2	NA	3.2
680	AT1G07650.2	| | Leucine-rich repeat transmembra	3.2	NA	3.2
797	AT3G23660.1	| | Sec23/Sec24 protein transport f	3.2	NA	3.2
942	ATCG00840.1 (+1)	|RPL23.1, RPL23 | ribosomal protein	NA	NA	3.2
404	AT1G08380.1	|PSAO | photosystem I subunit O | c	2.9	NA	3.2
255	AT1G73990.1	|SPPA, SPPA1 | signal peptide pepti	3.6	NA	3.1
439	AT4G13770.1	|CYP83A1, REF2 | cytochrome P450, f	2.7	NA	3.1
599	AT4G04040.1	|MEE51 | Phosphofructokinase family	3.5	NA	3.1

	Accession.Number	Description	pair_1	pair_2	pair_3
709	AT3G51160.1	|MUR1, MUR_1, GMD2 | NAD(P)-binding	3.1	NA	3.1
140	AT1G73110.1	| | P-loop containing nucleoside tr	4.2	NA	3.1
350	AT1G01610.1	|ATGPAT4, GPAT4 | glycerol-3-phosph	3.1	NA	3.1
454	AT3G62700.1	|ATMRP10, MRP10, ABCC14 | multidrug	3.1	NA	3.1
550	AT4G16130.1	|ARA1, ISA1, ATISA1 | arabinose kin	2.4	NA	3.1
335	AT1G15730.1	| | Cobalamin biosynthesis CobW-lik	3.2	NA	3.1
673	AT2G32450.1	| | Calcium-binding tetratricopepti	3.8	NA	3.1
54	AT5G46800.1	|BOU | Mitochondrial substrate carr	4.3	NA	3.1
92	AT1G09130.3	| | ATP-dependent caseinolytic (Clp	3.4	NA	3.1
315	AT2G33530.1	|scpl46 | serine carboxypeptidase-l	2.6	NA	3.1
441	AT5G09870.1	|CESA5 | cellulose synthase 5 | chr	3.1	NA	3.1
617	AT3G04340.1	|emb2458 | FtsH extracellular prote	3.1	NA	3.1
618	AT4G21710.1	|NRPB2, EMB1989, RPB2 | DNA-directe	3.1	NA	3.1
718	AT2G27730.1	| | copper ion binding | chr2:11820	3.1	NA	3.1
762	AT2G29200.1	|APUM1, PUM1 | pumilio 1 | chr2:125	3.1	NA	3.1
524	AT5G03040.1 (+2)	|iqd2 | IQ-domain 2 | chr5:710380-7	3.1	NA	3.1
109	AT5G46290.1	|KASI, KAS1 | 3-ketoacyl-acyl carri	3.6	NA	3.0
336	AT2G20580.1	|RPN1A, ATRPN1A | 26S proteasome re	4.6	NA	3.0
341	AT3G15980.1 (+3)	| | Coatomer, beta' subunit | chr3:	2.6	NA	3.0
352	AT5G19690.1	|STT3A | staurosporin and temperatu	3.5	NA	3.0
500	AT3G26710.1	|CCB1 | cofactor assembly of comple	3.8	NA	3.0
670	AT2G39010.1	|PIP2E, PIP2;6 | plasma membrane in	4.0	NA	3.0
126	AT3G55360.1	|CER10, ECR, ATTSC13, TSC13 | 3-oxo	3.2	NA	3.0
151	AT1G68830.1	|STN7 | STT7 homolog STN7 | chr1:25	4.2	NA	3.0
311	AT5G24690.1	| | Protein of unknown function (DU	3.5	NA	3.0
438	AT1G11260.1	|STP1, ATSTP1 | sugar transporter 1	3.0	NA	3.0
453	AT1G70940.1	|PIN3, ATPIN3 | Auxin efflux carrie	3.1	NA	3.0
487	AT2G29190.1 (+1)	|APUM2, PUM2 | pumilio 2 | chr2:125	3.0	NA	3.0
503	AT1G08930.1 (+1)	|ERD6 | Major facilitator superfami	3.0	NA	3.0
745	AT5G02160.1	| | unknown protein; FUNCTIONS IN:	3.0	NA	3.0

	Accession.Number	Description	pair_1	pair_2	pair_3
957	AT5G48620.1	| | Disease resistance protein (CC-	NA	NA	3.0
104	AT2G44490.1	|PEN2, BGLU26 | Glycosyl hydrolase	3.3	NA	3.0
185	AT2G32480.1	|ARASP | ARABIDOPSIS SERIN PROTEASE	3.5	NA	3.0
653	AT5G43470.1 (+1)	|RPP8, HRT, RCY1 | Disease resistan	4.2	NA	3.0
106	AT2G47110.1 (+1)	|UBQ6 | ubiquitin 6 | chr2:19344701	3.9	NA	3.0
168	AT1G45000.1	| | AAA-type ATPase family protein	3.8	NA	3.0
45	AT2G36250.1 (+1)	|FTSZ2-1, ATFTSZ2-1 | Tubulin/FtsZ	4.3	NA	3.0
83	AT5G58290.1	|RPT3 | regulatory particle triple-	4.1	NA	3.0
111	AT5G60790.1	|ATGCN1, GCN1 | ABC transporter fam	3.6	NA	3.0
240	AT1G56500.1	| | haloacid dehalogenase-like hydr	4.2	NA	3.0
265	AT5G12860.1 (+1)	|DiT1 | dicarboxylate transporter 1	2.7	NA	3.0
291	AT5G05780.1	|RPN8A, AE3, ATHMOV34 | RP non-ATPa	3.1	NA	3.0
488	AT5G63420.1	|emb2746 | RNA-metabolising metallo	2.5	NA	3.0
658	AT3G62010.2	| | unknown protein; LOCATED IN: ce	3.4	NA	3.0
141	AT4G22890.1 (+2)	|PGR5-LIKE A | PGR5-LIKE A | chr4:1	3.7	NA	2.9
426	AT2G47240.1 (+1)	|CER8, LACS1 | AMP-dependent synthe	2.9	NA	2.9
479	AT4G34830.1	|MRL1 | Pentatricopeptide repeat (P	2.9	NA	2.9
791	AT4G04850.2	|KEA3 | K+ efflux antiporter 3 | ch	2.9	NA	2.9
409	AT2G23670.1	|YCF37 | homolog of Synechocystis Y	2.7	NA	2.9
436	AT3G23820.1	|GAE6 | UDP-D-glucuronate 4-epimera	3.7	NA	2.9
464	AT3G07160.1	|ATGSL10, gsl10, CALS9 | glucan syn	3.3	NA	2.9
160	AT3G63160.1	| | FUNCTIONS IN: molecular_functio	3.2	NA	2.9
671	AT2G45710.1	| | Zinc-binding ribosomal protein	3.1	NA	2.9
102	AT3G62250.1	|UBQ5 | ubiquitin 5 | chr3:23037138	2.6	NA	2.9
657	AT4G39960.1	| | Molecular chaperone Hsp40/DnaJ	3.2	NA	2.9
371	AT1G74730.1	| | Protein of unknown function (DU	2.4	-0.5	2.9
468	AT3G52500.1	| | Eukaryotic aspartyl protease fa	2.9	NA	2.9
749	AT5G60540.1	|EMB2407, ATPDX2, PDX2 | pyridoxine	2.9	NA	2.9
287	AT5G07030.1	| | Eukaryotic aspartyl protease fa	2.0	NA	2.9
305	AT1G71810.1	| | Protein kinase superfamily prot	2.4	NA	2.9

	Accession.Number	Description	pair_1	pair_2	pair_3
435	AT5G17020.1 (+1)	|XPO1A, ATCRM1, ATXPO1, XPO1, HIT2	2.6	NA	2.9
446	AT3G10670.1	|ATNAP7, NAP7 | non-intrinsic ABC p	2.2	NA	2.9
486	AT4G31500.1	|CYP83B1, SUR2, RNT1, RED1, ATR4 |	2.9	NA	2.9
554	AT4G24810.2	| | Protein kinase superfamily prot	2.9	NA	2.9
587	AT4G15550.1	|IAGLU | indole-3-acetate beta-D-gl	2.9	NA	2.9
685	ATCG01060.1	|PSAC | iron-sulfur cluster binding	2.9	NA	2.9
722	AT5G59730.2	|ATEXO70H7, EXO70H7 | exocyst subun	2.9	NA	2.9
731	AT4G23250.1	|EMB1290, DUF26-21, RKC1, CRK17 | k	2.9	NA	2.9
734	AT3G51550.1	|FER | Malectin/receptor-like prote	2.9	NA	2.9
738	AT1G19450.1	| | Major facilitator superfamily p	2.9	NA	2.9
770	AT1G22710.1	|SUC2, SUT1, ATSUC2 | sucrose-proto	2.9	NA	2.9
951	AT4G36130.1	| | Ribosomal protein L2 family | c	NA	NA	2.9
567	AT4G32410.1	|CESA1, RSW1, AtCESA1 | cellulose s	3.3	NA	2.9
888	AT5G09510.1	| | Ribosomal protein S19 family pr	NA	-2.3	2.9
150	AT1G64190.1	| | 6-phosphogluconate dehydrogenas	3.4	NA	2.9
159	AT2G32080.1 (+1)	|PUR ALPHA-1 | purin-rich alpha 1 |	3.6	NA	2.9
572	AT5G08540.1	| | unknown protein; FUNCTIONS IN:	4.1	NA	2.8
312	AT4G16390.1	|SVR7 | pentatricopeptide (PPR) rep	3.8	NA	2.8
18	AT2G29550.1	|TUB7 | tubulin beta-7 chain | chr2	3.9	NA	2.8
282	AT2G45060.1	| | Uncharacterised conserved prote	2.8	NA	2.8
353	AT5G24650.1	| | Mitochondrial import inner memb	2.5	NA	2.8
416	AT3G20810.1 (+1)	|JMJD5 | 2-oxoglutarate (2OG) and F	2.8	NA	2.8
424	AT3G01310.2	| | Phosphoglycerate mutase-like fa	2.8	NA	2.8
514	AT1G73650.3	| | Protein of unknown function (DU	2.8	NA	2.8
534	AT4G03550.1	|ATGSL05, GSL05, ATGSL5, PMR4, GSL5	2.8	NA	2.8
748	AT4G38580.1	|ATFP6, HIPP26, FP6 | farnesylated	2.8	NA	2.8
801	AT5G05200.1	| | Protein kinase superfamily prot	2.8	NA	2.8
816	AT3G47620.1	|AtTCP14, TCP14 | TEOSINTE BRANCHED	2.8	NA	2.8
725	AT2G38670.1	|PECT1 | phosphorylethanolamine cyt	2.8	NA	2.8
55	AT3G08940.2	|LHCB4.2 | light harvesting complex	2.7	NA	2.8

	Accession.Number	Description	pair_1	pair_2	pair_3
231	AT1G30380.1		PSAK	photosystem I subunit K	c	3.1	NA	2.8
411	AT5G58260.1			oxidoreductases, acting on NADH	3.9	NA	2.8
21	AT4G20890.1		TUB9	tubulin beta-9 chain	chr4	4.3	NA	2.8
145	AT4G35100.1 (+1)		PIP3, PIP3A, PIP2;7, SIMIP	plasm	3.5	NA	2.8
243	AT3G02360.1 (+1)			6-phosphogluconate dehydrogenas	3.3	NA	2.8
295	AT1G01320.2			Tetratricopeptide repeat (TPR)-	3.7	NA	2.8
634	AT1G21250.1		WAK1, PRO25	cell wall-associated	3.2	NA	2.8
706	AT5G11770.1			NADH-ubiquinone oxidoreductase	2.9	NA	2.8
11	AT5G44340.1		TUB4	tubulin beta chain 4	chr5	3.9	NA	2.7
170	AT1G06430.1		FTSH8	FTSH protease 8	chr1:196	3.1	NA	2.7
177	AT1G10510.1		emb2004	RNI-like superfamily pro	3.9	NA	2.7
461	AT1G17580.1		MYA1, ATMYA1, XI-1	myosin 1	ch	2.7	NA	2.7
492	AT3G57650.1		LPAT2	lysophosphatidyl acyltrans	2.7	NA	2.7
619	AT5G11380.1		DXPS3	1-deoxy-D-xylulose 5-phosp	2.7	NA	2.7
761	AT2G22125.1		CSI1	binding	chr2:9406793-9414	2.5	NA	2.7
795	AT3G02350.1		GAUT9	galacturonosyltransferase	2.7	NA	2.7
465	AT2G25800.1			Protein of unknown function (DU	2.7	NA	2.7
40	AT1G06950.1		ATTIC110, TIC110	translocon at t	3.8	NA	2.7
60	ATCG00470.1		ATPE	ATP synthase epsilon chain	2.5	NA	2.7
592	AT5G45390.1		CLPP4, NCLPP4	CLP protease P4		2.6	NA	2.7
954	AT1G27400.1			Ribosomal protein L22p/L17e fam	NA	NA	2.7
90	AT3G19820.1 (+2)		DWF1, DIM, EVE1, DIM1, CBB1	cell	3.7	NA	2.7
97	AT2G30950.1		VAR2, FTSH2	FtsH extracellular p	3.4	NA	2.7
124	AT1G79040.1		PSBR	photosystem II subunit R		3.6	NA	2.7
173	AT4G01100.1		ADNT1	adenine nucleotide transpo	3.3	NA	2.7
742	AT4G12320.1		CYP706A6	cytochrome P450, family	3.1	NA	2.7
958	AT2G39390.1			Ribosomal L29 family protein		NA	NA	2.7
355	AT2G32730.1			26S proteasome regulatory compl	3.9	NA	2.7
363	AT3G62830.1 (+1)		UXS2, ATUXS2, AUD1	NAD(P)-bindin	4.2	NA	2.7
615	AT3G46060.1 (+2)	|ARA3, ARA-3, ATRABE1C, ATRAB8A, RA	2.9	NA	2.7

	Accession.Number	Description	pair_1	pair_2	pair_3
692	AT3G09740.1	|SYP71, ATSYP71 | syntaxin of plant	3.9	NA	2.7
15	AT3G08580.1 (+1)	|AAC1 | ADP/ATP carrier 1 | chr3:26	3.8	NA	2.7
128	AT1G53750.1	|RPT1A | regulatory particle triple	4.2	NA	2.7
814	AT1G22700.2	| | Tetratricopeptide repeat (TPR)-	2.7	NA	2.7
408	AT5G21430.1	| | Chaperone DnaJ-domain superfami	2.7	NA	2.7
521	AT4G10120.1 (+1)	|ATSPS4F | Sucrose-phosphate syntha	2.7	NA	2.7
595	AT5G27390.1	| | Mog1/PsbP/DUF1795-like photosys	3.2	NA	2.7
600	AT5G16715.1	|EMB2247 | ATP binding;valine-tRNA	4.4	NA	2.7
836	AT1G14930.1	| | Polyketide cyclase/dehydrase an	2.7	NA	2.7
948	AT1G09430.1	|ACLA-3 | ATP-citrate lyase A-3 | c	NA	NA	2.7
6	AT5G62690.1 (+1)	|TUB2 | tubulin beta chain 2 | chr5	3.7	NA	2.6
23	AT5G12250.1	|TUB6 | beta-6 tubulin | chr5:39613	3.6	NA	2.6
508	AT3G07100.1	|ERMO2, SEC24A | Sec23/Sec24 protei	3.4	NA	2.6
955	ATCG00780.1	|RPL14 | ribosomal protein L14 | ch	NA	NA	2.6
171	AT1G58684.1 (+2)	| | Ribosomal protein S5 family pro	2.5	NA	2.6
499	AT1G62640.1 (+1)	|KAS III | 3-ketoacyl-acyl carrier	3.1	NA	2.6
303	AT1G20200.1	|EMB2719, HAP15 | PAM domain (PCI/P	3.5	NA	2.6
419	AT3G20000.1	|TOM40 | translocase of the outer m	3.1	NA	2.6
75	AT1G71500.1	| | Rieske (2Fe-2S) domain-containi	3.1	NA	2.6
202	AT1G03475.1	|LIN2, HEMF1, ATCPO-I | Coproporphy	3.5	NA	2.6
700	AT1G72750.1	|ATTIM23-2, TIM23-2 | translocase i	3.0	NA	2.6
78	AT5G57350.1 (+1)	|AHA3, ATAHA3, HA3 | H(+)-ATPase 3	4.5	NA	2.6
216	AT5G14780.1	|FDH | formate dehydrogenase | chr5	3.3	NA	2.6
80	ATCG00800.1	| | structural constituent of ribos	2.5	NA	2.6
99	AT2G04030.2	|CR88, Hsp88.1, AtHsp90.5 | Chapero	3.3	NA	2.6
199	AT1G05140.1	| | Peptidase M50 family protein |	3.1	NA	2.6
271	AT3G63460.1	| | transducin family protein / WD-	3.3	NA	2.6
288	AT1G12770.1	|ISE1, EMB1586 | P-loop containing	2.6	NA	2.6
323	AT3G25680.1	| | FUNCTIONS IN: molecular_functio	2.6	NA	2.6
329	AT1G11410.1	| | S-locus lectin protein kinase f	3.0	NA	2.6

	Accession.Number	Description	pair_1	pair_2	pair_3
391	AT5G05170.1	|CESA3, IXR1, ATCESA3, ATH-B, CEV1	3.7	NA	2.6
475	AT5G47910.1	|RBOHD, ATRBOHD | respiratory burst	2.6	NA	2.6
678	AT3G02450.1	| | cell division protein ftsH, put	3.0	NA	2.6
726	AT5G47930.1	| | Zinc-binding ribosomal protein	2.3	NA	2.6
728	AT4G02620.1	| | vacuolar ATPase subunit F famil	2.7	NA	2.6
777	AT4G33220.1	|PME44, ATPME44 | pectin methyleste	2.6	NA	2.6
804	AT4G31390.1	| | Protein kinase superfamily prot	2.6	NA	2.6
937	ATCG00660.1	|RPL20 | ribosomal protein L20 | ch	NA	NA	2.6
165	AT5G41670.1 (+1)	| | 6-phosphogluconate dehydrogenas	3.4	NA	2.6
51	AT3G47470.1	|LHCA4, CAB4 | light-harvesting chl	3.0	NA	2.6
10	AT1G20010.1	|TUB5 | tubulin beta-5 chain | chr1	3.6	NA	2.5
248	AT5G17170.1	|ENH1 | rubredoxin family protein |	3.1	NA	2.5
144	AT5G19990.1	|RPT6A, ATSUG1 | regulatory particl	4.0	NA	2.5
317	AT2G47650.1	|UXS4 | UDP-xylose synthase 4 | chr	4.2	NA	2.5
535	AT5G58410.1	| | HEAT/U-box domain-containing pr	3.3	NA	2.5
57	AT5G19760.1	| | Mitochondrial substrate carrier	3.6	NA	2.5
381	AT3G27240.1	| | Cytochrome C1 family | chr3:100	4.1	NA	2.5
654	AT5G01920.1	|STN8 | Protein kinase superfamily	4.4	NA	2.5
959	AT4G25740.1	| | RNA binding Plectin/S10 domain-	NA	NA	2.5
338	AT2G31810.1	| | ACT domain-containing small sub	3.1	NA	2.5
406	AT3G52730.1	| | ubiquinol-cytochrome C reductas	2.7	NA	2.5
757	AT1G15930.1 (+1)	| | Ribosomal protein L7Ae/L30e/S12	2.5	NA	2.5
237	AT4G36220.1	|FAH1, CYP84A1 | ferulic acid 5-hyd	2.5	NA	2.5
430	AT1G30400.1 (+1)	|ATMRP1, EST1, ABCC1, ATABCC1, MRP1	2.5	NA	2.5
476	AT2G36810.1	| | ARM repeat superfamily protein	2.5	NA	2.5
571	AT5G54160.1	|ATOMT1, OMT1 | O-methyltransferase	3.5	NA	2.5
574	AT1G23080.3	|PIN7 | Auxin efflux carrier family	2.5	NA	2.5
612	AT1G44446.1	|CH1, ATCAO, CAO | Pheophorbide a o	2.5	NA	2.5
724	AT4G30810.1	|scpl29 | serine carboxypeptidase-l	2.5	NA	2.5
737	AT4G24750.1	| | Rhodanese/Cell cycle control ph	2.0	NA	2.5

	Accession.Number	Description	pair_1	pair_2	pair_3
792	AT4G36480.1 (+1)	|ATLCB1, LCB1, EMB2779, FBR11 | lon	2.5	NA	2.5
807	AT4G17770.1	|ATTPS5, TPS5 | trehalose phosphata	2.5	NA	2.5
812	AT1G50370.1	| | Calcineurin-like metallo-phosph	2.5	NA	2.5
432	AT5G22770.1 (+2)	|alpha-ADR | alpha-adaptin | chr5:7	3.2	NA	2.5
905	AT5G26742.2	|emb1138 | DEAD box RNA helicase (R	NA	NA	2.5
187	AT1G54780.1	|TLP18.3 | thylakoid lumen 18.3 kDa	3.2	NA	2.5
934	AT1G07320.1	|RPL4 | ribosomal protein L4 | chr1	NA	NA	2.5
176	AT1G70410.2	|ATBCA4, BCA4 | beta carbonic anhyd	3.8	NA	2.5
228	AT3G09790.1	|UBQ8 | ubiquitin 8 | chr3:3004111-	2.2	NA	2.5
386	AT1G80030.1 (+2)	| | Molecular chaperone Hsp40/DnaJ	3.2	NA	2.5
456	AT5G64580.1	| | AAA-type ATPase family protein	2.9	NA	2.5
538	AT2G07707.1 (+1)	| | Plant mitochondrial ATPase, F0	2.2	NA	2.5
710	AT1G25490.1	|RCN1, REGA, ATB BETA BETA, EER1 |	3.5	NA	2.5
716	AT1G63000.1	|NRS/ER, UER1 | nucleotide-rhamnose	2.9	NA	2.5
732	AT4G22310.1	| | Uncharacterised protein family	2.9	NA	2.5
178	AT5G16070.1	| | TCP-1/cpn60 chaperonin family p	3.7	NA	2.5
640	AT1G75780.1	|TUB1 | tubulin beta-1 chain | chr1	6.8	4.4	2.5
163	AT5G55280.1	|FTSZ1-1, ATFTSZ1-1, CPFTSZ | homol	3.8	NA	2.4
250	AT2G04842.1	|EMB2761 | threonyl-tRNA synthetase	3.5	NA	2.4
349	AT3G51140.1	| | Protein of unknown function (DU	3.2	NA	2.4
380	AT4G34090.2	| | unknown protein; FUNCTIONS IN:	3.4	NA	2.4
442	AT5G22330.1	|ATTIP49A, RIN1 | P-loop containing	3.2	NA	2.4
665	ATCG00420.1	|NDHJ | NADH dehydrogenase subunit	2.4	NA	2.4
460	AT5G19620.1	|EMB213, OEP80, ATOEP80, TOC75 | ou	2.1	NA	2.4
575	AT1G07250.1	|UGT71C4 | UDP-glucosyl transferase	2.1	NA	2.4
635	AT1G45474.1 (+1)	|LHCA5 | photosystem I light harves	3.4	NA	2.4
782	AT5G35590.1	|PAA1 | proteasome alpha subunit A1	2.1	NA	2.4
785	ATCG00170.1	|RPOC2 | DNA-directed RNA polymeras	2.4	NA	2.4
823	AT4G24220.1 (+1)	|VEP1, AWI31 | NAD(P)-binding Rossm	2.4	NA	2.4
830	AT3G09090.1 (+1)	|DEX1 | defective in exine formatio	2.4	NA	2.4

	Accession.Number	Description	pair_1	pair_2	pair_3
906	AT2G21660.1	|ATGRP7, CCR2, GR-RBP7, GRP7 | cold	NA	NA	2.4
923	ATCG00830.1 (+1)	|RPL2.1 | ribosomal protein L2 | ch	NA	NA	2.4
975	AT2G45220.1	| | Plant invertase/pectin methyles	NA	NA	2.4
357	AT2G20920.1	| | Protein of unknown function (DU	2.4	NA	2.4
559	AT3G50930.1	|BCS1 | cytochrome BC1 synthesis |	2.1	NA	2.4
46	AT1G50250.1	|FTSH1 | FTSH protease 1 | chr1:186	3.3	NA	2.4
82	ATCG00500.1	|ACCD | acetyl-CoA carboxylase carb	3.3	NA	2.4
118	AT1G78570.1	|RHM1, ROL1, ATRHM1 | rhamnose bios	4.1	NA	2.4
421	AT4G28470.1	|RPN1B, ATRPN1B | 26S proteasome re	4.0	NA	2.4
444	AT3G53180.1	| | glutamate-ammonia ligases;catal	2.8	NA	2.4
467	AT3G09840.1	|CDC48, ATCDC48, CDC48A | cell divi	5.7	3.3	2.4
566	AT2G24180.1	|CYP71B6 | cytochrome p450 71b6 | c	2.8	NA	2.4
776	AT1G21270.1	|WAK2 | wall-associated kinase 2 |	2.8	NA	2.4
907	AT1G76160.1	|sks5 | SKU5 similar 5 | chr1:28578	NA	NA	2.4
247	AT1G49970.1	|CLPR1, NCLPP5, SVR2 | CLP protease	2.5	NA	2.4
273	AT5G66680.1	|DGL1 | dolichyl-diphosphooligosacc	4.4	2.0	2.4
694	AT2G37710.1	|RLK | receptor lectin kinase | chr	3.4	NA	2.4
33	AT5G42270.1	|VAR1, FTSH5 | FtsH extracellular p	3.1	NA	2.4
555	AT5G42240.1	|scpl42 | serine carboxypeptidase-l	2.7	NA	2.4
227	AT1G61790.1	| | Oligosaccharyltransferase compl	2.3	NA	2.3
553	AT1G21630.1	| | Calcium-binding EF hand family	2.3	NA	2.3
940	AT1G10670.1 (+2)	|ACLA-1 | ATP-citrate lyase A-1 | c	NA	NA	2.3
259	AT2G18710.1	|SCY1 | SECY homolog 1 | chr2:81122	2.6	NA	2.3
364	AT2G36390.1	|SBE2.1, BE3 | starch branching enz	2.3	NA	2.3
378	AT3G13330.1	|PA200 | proteasome activating prot	2.3	NA	2.3
398	AT1G04530.1	|TPR4 | Tetratricopeptide repeat (T	3.1	NA	2.3
402	AT5G45510.2	| | Leucine-rich repeat (LRR) famil	3.1	NA	2.3
415	AT4G17090.1	|CT-BMY, BAM3, BMY8 | chloroplast b	3.9	NA	2.3
466	AT3G10690.1	|GYRA | DNA GYRASE A | chr3:3339612	2.3	NA	2.3
482	AT5G10690.1	| | pentatricopeptide (PPR) repeat-	2.3	NA	2.3

	Accession.Number	Description	pair_1	pair_2	pair_3
491	AT3G01300.1	| | Protein kinase superfamily prot	2.3	NA	2.3
498	AT3G51420.1	|SSL4, ATSSL4 | strictosidine synth	2.3	NA	2.3
518	AT5G26360.1	| | TCP-1/cpn60 chaperonin family p	2.3	NA	2.3
648	AT5G52520.1	|OVA6, PRORS1 | Class II aaRS and b	3.1	NA	2.3
723	AT5G09900.2	|EMB2107, RPN5A, MSA | 26S proteaso	2.7	NA	2.3
806	AT1G62180.1	|APR2, APSR, PRH43, PRH, ATAPR2 | 5	2.3	NA	2.3
824	AT3G49560.1	| | Mitochondrial import inner memb	2.1	NA	2.3
827	AT5G14120.1	| | Major facilitator superfamily p	2.3	NA	2.3
920	AT3G52140.2 (+1)	| | tetratricopeptide repeat (TPR)-	NA	NA	2.3
921	ATCG00710.1	|PSBH | photosystem II reaction cen	NA	NA	2.3
960	ATCG00820.1	|RPS19 | ribosomal protein S19 | ch	NA	NA	2.3
36	AT5G14040.1	|PHT3;1 | phosphate transporter 3;1	3.7	NA	2.3
61	AT3G05530.1	|RPT5A, ATS6A.2 | regulatory partic	3.7	NA	2.3
110	ATCG01110.1	|NDHH | NAD(P)H dehydrogenase subun	3.0	NA	2.3
270	AT4G29040.1	|RPT2a | regulatory particle AAA-AT	4.0	NA	2.3
121	AT4G03280.1	|PETC, PGR1 | photosynthetic electr	3.1	NA	2.3
34	AT5G19770.1 (+1)	|TUA3 | tubulin alpha-3 | chr5:6682	2.8	NA	2.3
254	AT1G08520.1	|ALB1, ALB-1V, V157, PDE166, CHLD |	2.5	NA	2.3
379	AT2G22360.1	| | DNAJ heat shock family protein	3.0	NA	2.3
101	AT5G12470.1	| | Protein of unknown function (DU	3.2	NA	2.3
258	AT5G03940.1	|FFC, 54CP, CPSRP54, SRP54CP | chlo	3.6	NA	2.3
20	AT1G29930.1	|CAB1, AB140, CAB140, LHCB1.3 | chl	3.1	NA	2.3
682	ATCG00670.1	|CLPP1, PCLPP | plastid-encoded CLP	2.6	NA	2.3
735	AT3G28710.1	| | ATPase, V0/A0 complex, subunit	3.3	1.0	2.3
28	AT4G10340.1	|LHCB5 | light harvesting complex o	2.3	NA	2.3
328	AT1G53500.1	|MUM4, RHM2, ATRHM2, ATMUM4 | NAD-d	3.6	NA	2.3
697	AT4G00400.1	|GPAT8, AtGPAT8 | glycerol-3-phosph	2.7	NA	2.2
772	AT1G09795.1	|ATATP-PRT2, HISN1B, ATP-PRT2 | ATP	2.7	NA	2.2
780	AT1G64710.1	| | GroES-like zinc-binding dehydro	2.7	NA	2.2
267	AT2G33040.1	|ATP3 | gamma subunit of Mt ATP syn	3.2	NA	2.2

	Accession.Number	Description	pair_1	pair_2	pair_3
331	AT4G24570.1	|DIC2 | dicarboxylate carrier 2 | c	2.2	NA	2.2
821	AT4G30610.1	|BRS1, SCPL24 | alpha/beta-Hydrolas	2.2	0.0	2.2
447	AT2G26910.1	|PDR4, ATPDR4 | pleiotropic drug re	2.2	NA	2.2
811	AT1G14670.1	| | Endomembrane protein 70 protein	2.2	NA	2.2
818	AT5G59420.1	|ORP3C | OSBP(oxysterol binding pro	2.2	0.0	2.2
950	AT1G68560.1	|ATXYL1, XYL1, TRG1 | alpha-xylosid	NA	NA	2.2
964	AT5G14950.1	|GMII, ATGMII | golgi alpha-mannosi	NA	NA	2.2
969	AT4G34950.1	| | Major facilitator superfamily p	NA	NA	2.2
91	AT3G02090.1	|MPPBETA | Insulinase (Peptidase fa	2.7	NA	2.2
213	AT2G26250.1	|FDH, KCS10 | 3-ketoacyl-CoA syntha	3.6	NA	2.2
2	AT2G39730.1	|RCA | rubisco activase | chr2:1657	2.7	NA	2.2
198	AT1G16300.1	|GAPCP-2 | glyceraldehyde-3-phospha	3.1	NA	2.2
325	AT1G32500.1	|ATNAP6, NAP6 | non-intrinsic ABC p	2.4	NA	2.2
580	AT5G49030.3	|OVA2 | tRNA synthetase class I (I,	2.9	NA	2.2
633	AT5G22800.1	|EMB86, EMB1030, EMB263 | Alanyl-tR	3.9	NA	2.2
868	AT1G59610.1	|ADL3, CF1, DRP2B, DL3 | dynamin-li	NA	-2.1	2.2
196	AT5G54270.1	|LHCB3, LHCB3*1 | light-harvesting	2.0	NA	2.2
422	AT4G28080.1	| | Tetratricopeptide repeat (TPR)-	3.8	NA	2.2
22	AT1G15820.1	|LHCB6, CP24 | light harvesting com	2.4	NA	2.2
27	AT1G61520.1 (+1)	|LHCA3 | photosystem I light harves	2.5	NA	2.2
35	AT2G33800.1	| | Ribosomal protein S5 family pro	2.0	NA	2.2
43	AT5G23060.1	|CaS | calcium sensing receptor | c	2.9	NA	2.2
209	AT3G63410.1	|APG1, VTE3, IEP37, E37 | S-adenosy	4.0	NA	2.2
506	AT2G01350.1	|QPT | quinolinate phoshoribosyltra	2.3	NA	2.2
593	AT4G00360.1	|CYP86A2, ATT1 | cytochrome P450, f	2.6	NA	2.2
596	AT1G50430.1 (+1)	|DWF5, PA, LE, ST7R, 7RED | Ergoste	2.6	NA	2.2
720	AT2G28800.1 (+1)	|ALB3 | 63 kDa inner membrane famil	2.6	NA	2.2
741	AT4G27700.1	| | Rhodanese/Cell cycle control ph	2.9	NA	2.2
866	AT1G10290.1	|ADL6, DRP2A | dynamin-like protein	NA	-2.1	2.2
166	AT5G23540.1	| | Mov34/MPN/PAD-1 family protein	3.5	NA	2.2

	Accession.Number	Description	pair_1	pair_2	pair_3
212	AT5G19550.1	|ASP2, AAT2 | aspartate aminotransf	3.5	NA	2.2
63	AT4G35250.1	| | NAD(P)-binding Rossmann-fold su	3.3	NA	2.1
298	AT5G62790.1	|DXR, PDE129 | 1-deoxy-D-xylulose 5	2.5	NA	2.1
659	AT1G65260.1	|PTAC4, VIPP1 | plastid transcripti	3.4	NA	2.1
16	AT1G49240.1	|ACT8 | actin 8 | chr1:18216539-182	2.7	NA	2.1
25	AT1G04820.1 (+1)	|TUA4, TOR2 | tubulin alpha-4 chain	2.8	NA	2.1
912	AT1G78630.1	|emb1473 | Ribosomal protein L13 fa	NA	NA	2.1
115	AT1G62020.1	| | Coatomer, alpha subunit | chr1:	2.5	NA	2.1
206	AT1G62750.1	|ATSCO1, ATSCO1/CPEF-G, SCO1 | Tran	2.5	NA	2.1
598	AT1G53440.1	| | Leucine-rich repeat transmembra	3.5	NA	2.1
925	AT1G48350.1	| | Ribosomal L18p/L5e family prote	NA	NA	2.1
294	AT5G16660.2	| | unknown protein; FUNCTIONS IN:	2.1	NA	2.1
437	AT5G51820.1	|PGM, ATPGMP, PGM1, STF1 | phosphog	3.9	NA	2.1
474	AT1G31800.1	|CYP97A3, LUT5 | cytochrome P450, f	2.1	NA	2.1
511	AT5G61810.1	| | Mitochondrial substrate carrier	2.1	NA	2.1
583	AT5G23300.1	|PYRD | pyrimidine d | chr5:7847792	2.1	NA	2.1
760	AT3G63520.1	|CCD1, ATCCD1, ATNCED1, NCED1 | car	3.1	NA	2.1
784	AT4G32300.1	|SD2-5 | S-domain-2 5 | chr4:155999	2.1	NA	2.1
790	AT1G64900.1	|CYP89A2, CYP89 | cytochrome P450,	2.1	NA	2.1
799	AT2G40060.1	| | Clathrin light chain protein |	2.1	NA	2.1
809	AT5G63510.2	|GAMMA CAL1 | gamma carbonic anhydr	2.1	NA	2.1
813	AT4G00026.1	| | FUNCTIONS IN: molecular_functio	2.1	NA	2.1
822	AT3G03100.1	| | NADH:ubiquinone oxidoreductase,	2.1	NA	2.1
826	AT2G28430.1	| | unknown protein; Has 28 Blast h	2.1	NA	2.1
870	AT2G30620.1	| | winged-helix DNA-binding transc	NA	-2.2	2.1
913	AT3G58750.1	|CSY2 | citrate synthase 2 | chr3:2	NA	NA	2.1
917	AT2G33450.1	| | Ribosomal L28 family | chr2:141	NA	NA	2.1
962	AT1G51100.1	| | unknown protein; FUNCTIONS IN:	NA	NA	2.1
963	AT4G20830.1	| | FAD-binding Berberine family pr	NA	NA	2.1
820	AT5G59250.1	| | Major facilitator superfamily p	2.1	NA	2.1

	Accession.Number	Description	pair_1	pair_2	pair_3
843	AT3G25070.1	|RIN4 | RPM1 interacting protein 4	2.1	NA	2.1
31	AT1G09340.1	|CRB, CSP41B, HIP1.3 | chloroplast	3.3	NA	2.1
911	AT1G75350.1	|emb2184 | Ribosomal protein L31 |	NA	NA	2.1
300	ATCG01090.1	|NDHI | NADPH dehydrogenases | chrC	2.8	NA	2.1
320	AT1G04810.1	| | 26S proteasome regulatory compl	3.3	NA	2.1
956	AT1G04270.1	|RPS15 | cytosolic ribosomal protei	NA	NA	2.1
568	AT3G44330.1	| | INVOLVED IN: protein processing	2.5	NA	2.1
750	AT2G32060.1 (+2)	| | Ribosomal protein L7Ae/L30e/S12	2.5	NA	2.1
768	AT5G58100.1	| | unknown protein; INVOLVED IN: p	2.5	NA	2.1
802	AT3G61650.1	|TUBG1 | gamma-tubulin | chr3:22812	2.5	NA	2.1
908	AT4G39260.1	|CCR1, ATGRP8, GR-RBP8, GRP8 | cold	NA	NA	2.1
42	AT1G74470.1	| | Pyridine nucleotide-disulphide	3.2	NA	2.1
52	AT4G27440.1 (+1)	|PORB | protochlorophyllide oxidore	2.9	NA	2.1
120	AT3G56940.1	|CRD1, CHL27, ACSF | dicarboxylate	2.8	NA	2.1
139	AT3G59780.1	| | Rhodanese/Cell cycle control ph	4.1	2.0	2.1
191	AT4G30010.1	| | unknown protein; FUNCTIONS IN:	3.3	NA	2.1
739	AT2G48070.1 (+1)	|RPH1 | resistance to phytophthora	2.1	NA	2.1
915	AT1G05190.1	|emb2394 | Ribosomal protein L6 fam	NA	NA	2.1
953	AT3G05560.1 (+2)	| | Ribosomal L22e protein family |	NA	NA	2.1
505	AT1G71220.1	|EBS1, UGGT, PSL2 | UDP-glucose:gly	2.6	NA	2.1
661	AT1G53430.1 (+1)	| | Leucine-rich repeat transmembra	3.1	NA	2.1
916	AT5G27770.1	| | Ribosomal L22e protein family |	NA	NA	2.1
919	AT2G21580.1 (+1)	| | Ribosomal protein S25 family pr	NA	NA	2.1
98	AT1G72370.1 (+1)	|P40, AP40, RP40, RPSAA | 40s ribos	2.3	NA	2.0
253	AT3G62530.1	| | ARM repeat superfamily protein	2.6	NA	2.0
693	AT4G12830.1	| | alpha/beta-Hydrolases superfami	2.6	NA	2.0
76	AT5G54770.1	|THI1, TZ, THI4 | thiazole biosynth	3.8	NA	2.0
87	AT2G19940.1 (+1)	| | oxidoreductases, acting on the	3.3	NA	2.0
161	AT2G42210.2	|ATOEP16-3, OEP16-3 | Mitochondrial	3.1	NA	2.0
203	AT5G47210.1	| | Hyaluronan / mRNA binding famil	2.1	NA	2.0

	Accession.Number	Description	pair_1	pair_2	pair_3
257	AT4G30950.1	|FAD6, FADC, SFD4 | fatty acid desa	3.0	NA	2.0
261	AT3G61820.1	| | Eukaryotic aspartyl protease fa	2.5	NA	2.0
663	AT1G70770.1 (+1)	| | Protein of unknown function DUF	3.1	NA	2.0
19	AT3G12110.1	|ACT11 | actin-11 | chr3:3858116-38	2.9	NA	2.0
153	AT1G52510.1	| | alpha/beta-Hydrolases superfami	3.4	NA	2.0
24	AT2G37620.1 (+2)	|ACT1, AAc1 | actin 1 | chr2:157797	2.6	NA	2.0
69	AT5G50850.1	|MAB1 | Transketolase family protei	3.6	NA	2.0
95	AT3G08530.1	| | Clathrin, heavy chain | chr3:25	2.4	NA	2.0
217	AT1G55670.1	|PSAG | photosystem I subunit G | c	2.4	NA	2.0
204	AT2G33150.1	|PKT3, PED1, KAT2 | peroxisomal 3-k	3.4	NA	2.0
301	AT4G21150.1 (+1)	|HAP6 | ribophorin II (RPN2) family	3.4	NA	2.0
368	AT2G31040.1	| | ATP synthase protein I -related	2.0	NA	2.0
405	AT1G63970.1 (+1)	|ISPF, MECPS | isoprenoid F | chr1:	2.3	NA	2.0
440	ATCG00190.1	|RPOB | RNA polymerase subunit beta	2.0	NA	2.0
448	AT4G28710.1	|XIH, ATXIH | Myosin family protein	2.0	NA	2.0
473	AT3G59110.1	| | Protein kinase superfamily prot	2.4	NA	2.0
477	AT2G35800.1	| | mitochondrial substrate carrier	2.0	NA	2.0
489	AT5G67560.1	|ATARLA1D, ARLA1D | ADP-ribosylatio	2.0	NA	2.0
539	AT3G61470.1	|LHCA2 | photosystem I light harves	3.1	NA	2.0
569	AT3G42170.1	| | BED zinc finger ;hAT family dim	2.4	NA	2.0
576	AT3G17970.1	|atToc64-III, TOC64-III | transloco	2.0	NA	2.0
584	AT3G07700.1 (+2)	| | Protein kinase superfamily prot	2.0	NA	2.0
590	AT5G11040.1	|TRS120, AtTRS120 | TRS120 | chr5:3	2.0	NA	2.0
609	AT2G25870.1	| | haloacid dehalogenase-like hydr	2.0	NA	2.0
614	AT5G64860.1	|DPE1 | disproportionating enzyme |	2.0	NA	2.0
711	AT5G36230.1	| | ARM repeat superfamily protein	2.7	NA	2.0
721	AT5G67385.1	| | Phototropic-responsive NPH3 fam	2.4	NA	2.0
771	AT1G29790.1 (+1)	| | S-adenosyl-L-methionine-depende	2.4	NA	2.0
788	AT1G06000.1	| | UDP-Glycosyltransferase superfa	2.7	NA	2.0
798	AT5G61910.4	| | DCD (Development and Cell Death	2.0	NA	2.0

	Accession.Number	Description	pair_1	pair_2	pair_3
805	AT5G23140.1	|CLPP2, NCLPP7 | nuclear-encoded CL	2.0	NA	2.0
817	AT4G24550.2	| | Clathrin adaptor complexes medi	2.0	NA	2.0
825	AT3G15000.1	| | cobalt ion binding | chr3:50503	2.0	NA	2.0
831	AT3G24530.1	| | AAA-type ATPase family protein	2.0	NA	2.0
832	AT3G48140.1	| | B12D protein | chr3:17778471-17	2.0	NA	2.0
837	AT3G04210.1	| | Disease resistance protein (TIR	2.0	NA	2.0
841	AT4G04570.1	|CRK40 | cysteine-rich RLK (RECEPTO	2.0	NA	2.0
850	AT3G05000.1	| | Transport protein particle (TRA	2.0	NA	2.0
910	AT2G38540.1	|LP1, LTP1, ATLTP1 | lipid transfer	NA	NA	2.0
924	AT5G08650.1	| | Small GTP-binding protein | chr	NA	NA	2.0
928	AT3G48560.1	|CSR1, ALS, AHAS, TZP5, IMR1 | chlo	NA	NA	2.0
931	AT5G35970.1	| | P-loop containing nucleoside tr	NA	NA	2.0
961	AT1G71695.1	| | Peroxidase superfamily protein	NA	NA	2.0
935	AT4G28740.1	| | FUNCTIONS IN: molecular_functio	NA	NA	1.9
966	AT1G53210.1	| | sodium/calcium exchanger family	NA	NA	1.7
973	AT4G16450.2 (+1)	| | unknown protein; FUNCTIONS IN:	NA	NA	1.7
604	AT1G32220.1	| | NAD(P)-binding Rossmann-fold su	1.6	NA	1.6
829	ATCG00740.1	|RPOA | RNA polymerase subunit alph	1.6	NA	1.6
855	AT3G62550.1	| | Adenine nucleotide alpha hydrol	1.6	NA	1.6
926	AT3G21200.1	|PGR7 | proton gradient regulation	NA	NA	1.6
947	AT5G14420.1 (+3)	|RGLG2 | RING domain ligase2 | chr5	NA	NA	1.6
979	AT1G02120.1	|VAD1 | GRAM domain family protein	NA	NA	1.6
987	AT1G79390.1	| | unknown protein; Has 30201 Blas	NA	NA	1.6
989	AT3G26935.1	| | DHHC-type zinc finger family pr	NA	NA	1.6
993	AT2G03820.1	| | nonsense-mediated mRNA decay NM	NA	NA	1.6
909	AT4G08950.1	|EXO | Phosphate-responsive 1 famil	NA	NA	1.4
918	AT3G28220.1	| | TRAF-like family protein | chr3	NA	NA	1.4
972	AT1G31410.1	| | putrescine-binding periplasmic	NA	NA	1.4
974	AT1G67785.1	| | unknown protein; Has 30 Blast h	NA	NA	1.4
990	AT3G51510.1	| | unknown protein; FUNCTIONS IN:	NA	NA	1.4

	Accession.Number	Description	pair_1	pair_2	pair_3
589	AT1G05350.1	| | NAD(P)-binding Rossmann-fold su	1.2	NA	1.2
845	AT3G15680.1	| | Ran BP2/NZF zinc finger-like su	1.2	NA	1.2
857	AT2G27290.1	| | Protein of unknown function (DU	1.2	NA	1.2
880	AT3G25760.1	|AOC1, ERD12 | allene oxide cyclase	NA	-0.8	1.2
929	AT5G57020.1	|NMT1, ATNMT1 | myristoyl-CoA:prote	NA	NA	1.2
932	AT2G20050.1 (+1)	| | protein serine/threonine phosph	NA	NA	1.2
939	AT1G01100.1 (+2)	| | 60S acidic ribosomal protein fa	NA	NA	1.2
945	AT5G23670.1 (+1)	|LCB2 | long chain base2 | chr5:798	NA	NA	1.2
965	AT1G28290.1 (+1)	|AGP31 | arabinogalactan protein 31	NA	NA	1.2
967	AT2G15430.1	|RBP36A, RPB35.5A, NRPB3, NRPD3, NR	NA	NA	1.2
981	AT1G72340.1	| | NagB/RpiA/CoA transferase-like	NA	NA	1.2
985	AT4G40050.1	| | Protein of unknown function (DU	NA	NA	1.2
991	AT2G32160.2 (+1)	| | S-adenosyl-L-methionine-depende	NA	NA	1.2
577	AT5G11840.1	| | Protein of unknown function (DU	1.0	NA	1.0
751	AT2G20530.1 (+1)	|ATPHB6, PHB6 | prohibitin 6 | chr2	1.0	NA	1.0
819	AT4G26070.2 (+1)	|MEK1, NMAPKK, ATMEK1, MKK1 | MAP k	1.0	NA	1.0
846	AT1G42960.1	| | expressed protein localized to	1.0	NA	1.0
968	AT5G53650.1	| | unknown protein; FUNCTIONS IN:	NA	NA	1.0
977	AT4G12390.1	|PME1 | pectin methylesterase inhib	NA	NA	1.0
995	AT3G19810.1	| | Protein of unknown function (DU	NA	NA	1.0
537	AT5G54110.1	|ATMAMI, MAMI | membrane-associated	0.7	NA	0.7
542	AT5G47890.1	| | NADH-ubiquinone oxidoreductase	0.7	NA	0.7
800	AT3G43980.1 (+2)	| | Ribosomal protein S14p/S29e fam	0.7	NA	0.7
922	AT4G39090.1	|RD19, RD19A | Papain family cystei	NA	NA	0.7
941	AT5G51010.1	| | Rubredoxin-like superfamily pro	NA	NA	0.7
946	AT1G10700.1	|PRS3 | phosphoribosyl pyrophosphat	NA	NA	0.7
980	AT3G44720.1	|ADT4 | arogenate dehydratase 4 | c	NA	NA	0.7
986	AT4G26500.1	|EMB1374, CPSUFE, ATSUFE, SUFE1 | c	NA	NA	0.7
988	AT3G12260.1	| | LYR family of Fe/S cluster biog	NA	NA	0.7
602	AT2G20230.1	| | Tetraspanin family protein | ch	0.4	NA	0.4

	Accession.Number	Description	pair_1	pair_2	pair_3
927	AT5G60980.2	| | Nuclear transport factor 2 (NTF	NA	NA	0.4
936	AT3G55430.1	| | O-Glycosyl hydrolases family 17	NA	NA	0.4
943	AT1G34130.1	|STT3B | staurosporin and temperatu	NA	NA	0.4
983	AT3G59920.1	|ATGDI2, GDI2 | RAB GDP dissociatio	NA	NA	0.4
984	AT5G45420.1	| | Duplicated homeodomain-like sup	NA	NA	0.4
835	AT5G67590.1	|FRO1 | NADH-ubiquinone oxidoreduct	0.0	NA	0.0
904	AT4G34150.1	| | Calcium-dependent lipid-binding	NA	0.0	0.0
914	AT3G09260.1	|PYK10, PSR3.1, BGLU23, LEB | Glyco	NA	NA	0.0
970	AT1G71710.1	| | DNAse I-like superfamily protei	NA	NA	0.0
978	AT5G17770.1	|ATCBR, CBR1, CBR | NADH:cytochrome	NA	NA	0.0
994	AT1G50600.1	|SCL5 | scarecrow-like 5 | chr1:187	NA	NA	0.0
949	AT5G06690.1	|WCRKC1 | WCRKC thioredoxin 1 | chr	NA	NA	-0.4
902	AT2G23600.1	|ACL, ATMES2, MES2, ATME8, ME8 | ac	NA	0.7	-0.7
992	AT3G25620.2	| | ABC-2 type transporter family p	NA	NA	-0.7
996	AT5G07572.1	| | unknown protein; FUNCTIONS IN:	NA	NA	-0.7
930	AT1G04690.1	|KAB1, KV-BETA1 | potassium channel	NA	NA	-1.2
976	AT5G13420.1	| | Aldolase-type TIM barrel family	NA	NA	-1.2
982	AT4G00620.1	| | Amino acid dehydrogenase family	NA	NA	-1.2
938	AT4G21180.1	|ATERDJ2B | DnaJ / Sec63 Brl domain	NA	NA	-1.4
971	AT3G12290.1	| | Amino acid dehydrogenase family	NA	NA	-1.6
899	AT1G12850.1	| | Phosphoglycerate mutase family	NA	1.7	-1.7
891	AT1G52600.1	| | Peptidase S24/S26A/S26B/S26C fa	NA	2.0	-2.0
933	AT1G21750.1	|ATPDIL1-1, ATPDI5, PDI5, PDIL1-1 |	NA	NA	-2.0
890	AT3G57290.1	|EIF3E, TIF3E1, ATEIF3E-1, INT-6, A	NA	2.1	-2.1
900	AT1G63810.1	| | CONTAINS InterPro DOMAIN/s: Nra	NA	2.1	-2.4
863	AT2G14110.1	| | Haloacid dehalogenase-like hydr	NA	2.1	-2.5
944	AT1G79940.1 (+2)	|ATERDJ2A | DnaJ / Sec63 Brl domain	NA	NA	-3.1
894	AT1G29880.1	| | glycyl-tRNA synthetase / glycin	NA	2.3	-3.1
884	AT2G17290.1	|CPK6, ATCDPK3, ATCPK6 | Calcium-de	NA	2.2	-4.0
860	AT4G35310.1	|CPK5, ATCPK5 | calmodulin-domain p	NA	2.3	-4.1

	Accession.Number	Description	pair_1	pair_2	pair_3
96	AT1G28380.1	|NSL1 | MAC/Perforin domain-contain	-8.5	-4.0	-4.5
3	ATCG00120.1	|ATPA | ATP synthase subunit alpha	2.2	NA	NA
4	AT1G42970.1	|GAPB | glyceraldehyde-3-phosphate	2.1	NA	NA
5	AT4G20360.1	|ATRAB8D, ATRABE1B, RABE1b | RAB GT	2.1	NA	NA
8	AT5G09810.1	|ACT7 | actin 7 | chr5:3052809-3054	2.7	NA	NA
9	AT1G12900.1	|GAPA-2 | glyceraldehyde 3-phosphat	2.2	NA	NA
12	AT3G26650.1	|GAPA, GAPA-1 | glyceraldehyde 3-ph	2.2	NA	NA
13	AT3G14420.1 (+1)	| | Aldolase-type TIM barrel family	2.2	NA	NA
14	AT2G07698.1	| | ATPase, F1 complex, alpha subun	2.4	NA	NA
17	AT4G04640.1	|ATPC1 | ATPase, F1 complex, gamma	2.4	NA	NA
26	AT3G04120.1	|GAPC, GAPC-1, GAPC1 | glyceraldehy	2.6	NA	NA
29	AT2G34430.1	|LHB1B1, LHCB1.4 | light-harvesting	2.0	NA	NA
30	AT1G13440.1	|GAPC-2, GAPC2 | glyceraldehyde-3-p	2.6	NA	NA
32	AT5G25980.2	|TGG2, BGLU37 | glucoside glucohydr	2.5	2.2	NA
38	AT5G14740.2	|CA2, CA18, BETA CA2 | carbonic anh	2.1	NA	NA
39	AT1G31330.1	|PSAF | photosystem I subunit F | c	2.2	NA	NA
41	AT4G13940.1	|HOG1, EMB1395, SAHH1, MEE58, ATSAH	2.1	NA	NA
44	AT1G78900.1 (+1)	|VHA-A | vacuolar ATP synthase subu	2.2	NA	NA
47	AT1G54270.1	|EIF4A-2 | eif4a-2 | chr1:20260495-	2.4	NA	NA
49	AT3G13920.1	|EIF4A1, RH4, TIF4A1 | eukaryotic t	2.5	NA	NA
50	AT1G23310.1	|GGT1, AOAT1, GGAT1 | glutamate:gly	3.0	NA	NA
56	ATCG00130.1	|ATPF | ATPase, F0 complex, subunit	2.5	NA	NA
58	AT3G04840.1	| | Ribosomal protein S3Ae | chr3:1	2.3	NA	NA
59	AT5G25460.1	| | Protein of unknown function, DU	3.1	NA	NA
62	AT3G14210.1	|ESM1 | epithiospecifier modifier 1	3.4	2.7	NA
64	AT1G45201.1	|ATTLL1, TLL1 | triacylglycerol lip	2.7	NA	NA
65	AT3G53420.1 (+1)	|PIP2A, PIP2, PIP2;1 | plasma membr	2.8	NA	NA
66	AT3G21180.1	|ACA9, ATACA9 | autoinhibited Ca(2+	2.8	NA	NA
67	AT5G17920.1 (+1)	|ATCIMS, ATMETS, ATMS1 | Cobalamin-	2.2	NA	NA
70	AT2G38230.1	|ATPDX1.1, PDX1.1 | pyridoxine bios	2.9	NA	NA

	Accession.Number	Description	pair_1	pair_2	pair_3
73	AT2G21390.1	| | Coatomer, alpha subunit | chr2:	2.4	NA	NA
74	AT3G46780.1	|PTAC16 | plastid transcriptionally	2.1	NA	NA
77	AT5G11420.1	| | Protein of unknown function, DU	3.1	NA	NA
79	AT3G11130.1	| | Clathrin, heavy chain | chr3:34	2.3	NA	NA
84	AT3G44310.1 (+1)	|NIT1, ATNIT1, NITI | nitrilase 1 |	2.4	2.1	NA
86	AT5G35360.3	|CAC2 | acetyl Co-enzyme a carboxyl	2.6	NA	NA
88	AT5G01410.1	|PDX1, ATPDX1.3, RSR4, PDX1.3, ATPD	2.5	NA	NA
89	AT3G01500.2	|CA1 | carbonic anhydrase 1 | chr3:	2.0	NA	NA
94	AT1G22410.1	| | Class-II DAHP synthetase family	3.6	2.4	NA
100	AT1G03630.2	|POR C, PORC | protochlorophyllide	2.7	NA	NA
103	AT4G39980.1	|DHS1 | 3-deoxy-D-arabino-heptuloso	3.5	NA	NA
105	AT3G09630.1	| | Ribosomal protein L4/L1 family	2.0	NA	NA
107	AT4G34670.1	| | Ribosomal protein S3Ae | chr4:1	2.7	NA	NA
108	AT5G50920.1	|CLPC, ATHSP93-V, HSP93-V, DCA1, CL	2.7	NA	NA
112	AT3G54050.1 (+1)	|HCEF1 | high cyclic electron flow	2.5	NA	NA
113	AT3G29360.1 (+1)	| | UDP-glucose 6-dehydrogenase fam	2.4	NA	NA
114	AT1G09750.1	| | Eukaryotic aspartyl protease fa	2.6	NA	NA
116	AT5G15490.1	| | UDP-glucose 6-dehydrogenase fam	2.6	NA	NA
117	AT1G56070.1	|LOS1 | Ribosomal protein S5/Elonga	2.6	NA	NA
119	AT5G58330.2	| | lactate/malate dehydrogenase fa	3.0	NA	NA
122	AT3G19170.1	|ATPREP1, ATZNMP, PREP1 | presequen	3.3	NA	NA
123	AT3G47520.1	|MDH | malate dehydrogenase | chr3:	2.3	NA	NA
125	AT4G02930.1	| | GTP binding Elongation factor T	2.1	NA	NA
129	AT1G72150.1	|PATL1 | PATELLIN 1 | chr1:27148558	3.5	NA	NA
130	AT1G11750.1	|CLPP6, NCLPP1, NCLPP6 | CLP protea	2.5	NA	NA
131	AT5G46110.3	|APE2, TPT | Glucose-6-phosphate/ph	2.6	NA	NA
132	AT1G02500.1 (+1)	|SAM1, SAM-1, MAT1, AtSAM1 | S-aden	2.4	NA	NA
134	AT4G33510.1	|DHS2 | 3-deoxy-d-arabino-heptuloso	3.4	2.2	NA
135	AT3G03780.1 (+2)	|ATMS2, MS2 | methionine synthase 2	2.4	NA	NA
136	AT4G32260.1	| | ATPase, F0 complex, subunit B/B	2.4	NA	NA

	Accession.Number	Description	pair_1	pair_2	pair_3
137	AT1G48030.1 (+1)	|mtLPD1 | mitochondrial lipoamide d	2.4	NA	NA
138	AT3G63140.1	|CSP41A | chloroplast stem-loop bin	2.1	NA	NA
142	AT5G02870.1	| | Ribosomal protein L4/L1 family	2.1	NA	NA
143	AT5G23120.1	|HCF136 | photosystem II stability/	2.7	NA	NA
146	AT2G31610.1	| | Ribosomal protein S3 family pro	2.4	NA	NA
147	AT4G31990.1 (+2)	|ASP5, AAT3, ATAAT1 | aspartate ami	3.0	2.3	NA
148	AT3G22890.1	|APS1 | ATP sulfurylase 1 | chr3:81	2.4	NA	NA
149	AT1G23190.1	|PGM3 | Phosphoglucomutase/phosphom	3.5	2.1	NA
152	AT4G25080.4	|CHLM | magnesium-protoporphyrin IX	3.6	NA	NA
155	AT3G06650.1	|ACLB-1 | ATP-citrate lyase B-1 | c	2.4	2.0	NA
156	AT4G34350.1	|CLB6, ISPH, HDR | 4-hydroxy-3-meth	2.1	NA	NA
157	AT1G01090.1	|PDH-E1 ALPHA | pyruvate dehydrogen	2.1	2.0	NA
158	AT3G48750.1	|CDKA;1, CDC2AAT, CDK2, CDC2, CDC2A	3.8	2.0	NA
167	AT5G63570.1	|GSA1 | glutamate-1-semialdehyde-2,	2.6	NA	NA
169	AT2G33210.2	|HSP60-2 | heat shock protein 60-2	2.8	2.5	NA
172	AT3G48870.1	|ATCLPC, ATHSP93-III, HSP93-III | C	2.7	NA	NA
174	AT3G18490.1	| | Eukaryotic aspartyl protease fa	2.5	NA	NA
175	AT2G34590.1	| | Transketolase family protein |	2.3	NA	NA
179	AT3G06510.2	|SFR2 | Glycosyl hydrolase superfam	2.4	NA	NA
180	AT2G15620.1	|NIR1, NIR, ATHNIR | nitrite reduct	4.6	2.7	NA
181	AT2G47730.1	|ATGSTF8, ATGSTF5, GST6, GSTF8 | gl	2.7	NA	NA
182	AT2G44640.1	| | FUNCTIONS IN: molecular_functio	3.1	NA	NA
183	AT1G02280.1 (+1)	|TOC33, ATTOC33, PPI1 | translocon	2.3	NA	NA
186	AT2G39770.1 (+1)	|CYT1, VTC1, SOZ1, EMB101, GMP1 | G	2.6	NA	NA
188	AT2G36530.1	|LOS2, ENO2 | Enolase | chr2:153210	2.1	NA	NA
189	AT3G22960.1	|PKP1, PKP-ALPHA | Pyruvate kinase	2.7	NA	NA
190	AT1G70730.1	|PGM2 | Phosphoglucomutase/phosphom	3.2	NA	NA
192	AT5G30510.1	|RPS1, ARRPS1 | ribosomal protein S	2.5	NA	NA
193	AT3G61440.1	|ATCYSC1, ARATH;BSAS3;1, CYSC1 | cy	2.8	NA	NA
194	AT3G03960.1	| | TCP-1/cpn60 chaperonin family p	2.4	NA	NA

	Accession.Number	Description	pair_1	pair_2	pair_3
195	AT4G01050.1	|TROL | thylakoid rhodanese-like |	2.6	NA	NA
197	AT2G45960.3	|PIP1B, TMP-A, ATHH2, PIP1;2 | plas	4.4	2.5	NA
205	AT3G58140.1	| | phenylalanyl-tRNA synthetase cl	2.7	NA	NA
207	AT2G38750.1	|ANNAT4 | annexin 4 | chr2:16196582	2.5	NA	NA
208	AT1G80410.2	|EMB2753 | tetratricopeptide repeat	3.7	NA	NA
210	AT1G14810.1	| | semialdehyde dehydrogenase fami	2.9	NA	NA
211	AT3G48730.1	|GSA2 | glutamate-1-semialdehyde 2,	2.3	NA	NA
214	AT2G10940.1 (+1)	| | Bifunctional inhibitor/lipid-tr	2.4	NA	NA
218	AT5G55190.1	|RAN3, ATRAN3 | RAN GTPase 3 | chr5	2.1	NA	NA
219	AT4G34450.1	| | coatomer gamma-2 subunit, putat	4.7	2.8	NA
220	AT3G20790.1	| | NAD(P)-binding Rossmann-fold su	3.0	NA	NA
221	AT1G69740.1 (+1)	|HEMB1 | Aldolase superfamily prote	2.0	NA	NA
224	AT5G28840.1 (+1)	|GME | GDP-D-mannose 3',5'-epimeras	2.6	2.2	NA
225	AT3G58610.1 (+2)	| | ketol-acid reductoisomerase | c	2.4	NA	NA
226	AT3G42050.1	| | vacuolar ATP synthase subunit H	2.7	NA	NA
229	AT2G39800.3	|P5CS1 | delta1-pyrroline-5-carboxy	2.1	NA	NA
230	AT4G08870.1	| | Arginase/deacetylase superfamil	2.3	NA	NA
233	AT1G76030.1	| | ATPase, V1 complex, subunit B p	3.1	NA	NA
235	AT3G23990.1	|HSP60, HSP60-3B | heat shock prote	2.4	NA	NA
236	AT1G30630.1	| | Coatomer epsilon subunit | chr1	2.0	NA	NA
238	AT1G66200.1	|ATGSR2, GSR2, GLN1;2 | glutamine s	2.0	NA	NA
241	AT3G14940.1	|ATPPC3, PPC3 | phosphoenolpyruvate	2.5	NA	NA
244	AT1G19920.1	|APS2, ASA1 | Pseudouridine synthas	2.7	NA	NA
245	AT4G27680.1	| | P-loop containing nucleoside tr	2.1	NA	NA
246	AT1G22530.1	|PATL2 | PATELLIN 2 | chr1:7955773-	3.2	2.1	NA
252	AT2G22250.2 (+1)	|ATAAT, AAT, MEE17 | aspartate amin	2.8	NA	NA
260	AT1G18080.1	|ATARCA, RACK1A_AT, RACK1A | Transd	2.1	NA	NA
262	AT1G52230.1	|PSAH2, PSAH-2, PSI-H | photosystem	2.0	NA	NA
263	AT3G01290.1	| | SPFH/Band 7/PHB domain-containi	3.5	3.3	NA
266	AT5G65010.2	|ASN2 | asparagine synthetase 2 | c	2.1	NA	NA

	Accession.Number	Description	pair_1	pair_2	pair_3
268	AT3G25520.1	|ATL5, PGY3, OLI5, RPL5A | ribosoma	2.0	NA	NA
269	AT4G39710.1	|FKBP16-2 | FK506-binding protein 1	2.8	NA	NA
274	AT1G57720.1 (+1)	| | Translation elongation factor E	3.4	NA	NA
275	AT3G58730.1	| | vacuolar ATP synthase subunit D	2.6	NA	NA
277	AT4G30920.1	| | Cytosol aminopeptidase family p	2.4	2.2	NA
279	AT4G34200.1	|EDA9 | D-3-phosphoglycerate dehydr	3.2	NA	NA
280	AT1G09640.1	| | Translation elongation factor E	3.0	2.3	NA
281	AT4G34870.1	|ROC5, ATCYP1 | rotamase cyclophili	2.0	NA	NA
283	AT1G01300.1	| | Eukaryotic aspartyl protease fa	2.1	NA	NA
284	AT2G21960.1	| | unknown protein; LOCATED IN: ch	2.5	NA	NA
285	AT3G09820.2	|ADK1 | adenosine kinase 1 | chr3:3	2.1	NA	NA
286	AT3G49720.1 (+1)	| | unknown protein; FUNCTIONS IN:	2.4	NA	NA
289	AT5G17990.1	|TRP1, pat1 | tryptophan biosynthes	3.4	NA	NA
292	AT1G65960.2	|GAD2 | glutamate decarboxylase 2 |	2.9	2.1	NA
296	AT2G34470.1	|UREG, PSKF109 | urease accessory p	1.9	NA	NA
297	AT3G06050.1	|PRXIIF, ATPRXIIF | peroxiredoxin I	2.2	NA	NA
299	AT1G34430.1	|EMB3003 | 2-oxoacid dehydrogenases	3.1	2.2	NA
304	AT5G40770.1	|ATPHB3, PHB3 | prohibitin 3 | chr5	3.2	NA	NA
306	AT2G05990.1 (+1)	|MOD1, ENR1 | NAD(P)-binding Rossma	2.3	NA	NA
308	AT3G52880.1	|ATMDAR1, MDAR1 | monodehydroascorb	2.3	2.1	NA
309	AT1G18500.1	|MAML-4, IPMS1 | methylthioalkylmal	2.9	NA	NA
310	AT5G51070.1	|ERD1, CLPD, SAG15 | Clp ATPase | c	2.4	NA	NA
313	AT3G56150.1 (+1)	|EIF3C, ATEIF3C-1, EIF3C-1, ATTIF3C	2.3	2.3	NA
314	AT5G11480.1	| | P-loop containing nucleoside tr	1.7	NA	NA
316	AT1G79560.1	|EMB156, EMB36, EMB1047, FTSH12 | F	3.4	NA	NA
319	AT5G06870.1	|PGIP2, ATPGIP2 | polygalacturonase	1.9	NA	NA
321	AT1G79550.1 (+1)	|PGK | phosphoglycerate kinase | ch	2.0	NA	NA
322	AT4G24190.1	|SHD, HSP90.7, AtHsp90.7, AtHsp90-7	3.8	2.0	NA
327	AT3G09830.1 (+1)	| | Protein kinase superfamily prot	2.0	NA	NA
330	AT1G17745.2	|PGDH | D-3-phosphoglycerate dehydr	2.5	NA	NA

	Accession.Number	Description	pair_1	pair_2	pair_3
332	AT1G70520.1	|CRK2 | cysteine-rich RLK (RECEPTOR	2.9	NA	NA
333	AT4G38510.5	| | ATPase, V1 complex, subunit B p	3.0	NA	NA
339	AT5G42650.1	|AOS, CYP74A, DDE2 | allene oxide s	2.6	NA	NA
340	AT1G67730.1	|YBR159, KCR1, ATKCR1 | beta-ketoac	2.2	NA	NA
342	AT5G67630.1	| | P-loop containing nucleoside tr	2.7	NA	NA
343	AT1G01080.1	| | RNA-binding (RRM/RBD/RNP motifs	2.3	NA	NA
345	AT4G17040.1	|CLPR4 | CLP protease R subunit 4 |	2.0	NA	NA
346	AT3G04790.1	| | Ribose 5-phosphate isomerase, t	2.6	NA	NA
351	AT1G50480.1	|THFS | 10-formyltetrahydrofolate s	3.9	2.1	NA
356	AT1G16720.1	|HCF173 | high chlorophyll fluoresc	2.3	NA	NA
358	AT2G18330.1	| | AAA-type ATPase family protein	2.1	NA	NA
359	AT1G74640.1	| | alpha/beta-Hydrolases superfami	1.7	NA	NA
361	AT3G25860.1	|LTA2, PLE2 | 2-oxoacid dehydrogena	2.1	NA	NA
365	AT1G15690.1	|AVP1, ATAVP3, AVP-3, AtVHP1;1 | In	2.4	NA	NA
366	AT1G15980.1	|NDF1, NDH48 | NDH-dependent cyclic	2.7	NA	NA
367	AT2G44530.2	| | Phosphoribosyltransferase famil	2.4	NA	NA
369	AT2G28900.1	|OEP16, ATOEP16-L, ATOEP16-1, OEP16	2.3	NA	NA
370	AT3G56910.1	|PSRP5 | plastid-specific 50S ribos	2.2	NA	NA
372	AT5G03455.1	|CDC25, ARATH;CDC25, ACR2 | Rhodane	1.4	NA	NA
373	AT1G24510.1	| | TCP-1/cpn60 chaperonin family p	2.1	NA	NA
374	AT1G51980.1	| | Insulinase (Peptidase family M1	3.1	NA	NA
375	AT3G48000.1	|ALDH2B4, ALDH2, ALDH2A | aldehyde	2.1	2.1	NA
382	AT1G79870.1	| | D-isomer specific 2-hydroxyacid	2.3	NA	NA
383	AT4G23850.1	|LACS4 | AMP-dependent synthetase a	2.6	NA	NA
384	AT1G59900.1	|AT-E1 ALPHA, E1 ALPHA | pyruvate d	2.4	NA	NA
385	AT5G20280.1	|ATSPS1F, SPS1F | sucrose phosphate	2.5	NA	NA
387	AT2G20760.1	| | Clathrin light chain protein |	2.5	NA	NA
388	AT2G20990.3	|SYTA | synaptotagmin A | chr2:9014	3.2	NA	NA
389	AT5G67030.1	|ABA1, LOS6, NPQ2, ATABA1, ZEP, IBS	2.3	NA	NA
392	AT3G22845.1	| | emp24/gp25L/p24 family/GOLD fam	2.7	NA	NA

	Accession.Number	Description	pair_1	pair_2	pair_3
393	AT4G36250.1	|ALDH3F1 | aldehyde dehydrogenase 3	2.5	1.0	NA
394	AT3G19960.1	|ATM1 | myosin 1 | chr3:6949787-695	3.2	3.1	NA
395	AT1G14150.1	|PQL1, PQL2 | PsbQ-like 2 | chr1:48	2.5	NA	NA
396	AT3G17020.1	| | Adenine nucleotide alpha hydrol	2.2	NA	NA
399	AT5G64290.1	|DCT, DIT2.1 | dicarboxylate transp	2.8	NA	NA
400	AT4G24820.1 (+1)	| | 26S proteasome, regulatory subu	2.5	NA	NA
401	AT1G51805.1	| | Leucine-rich repeat protein kin	3.1	NA	NA
403	AT4G04770.1	|ATABC1, LAF6, ATNAP1, ABC1 | ATP b	3.1	NA	NA
407	AT5G20890.1	| | TCP-1/cpn60 chaperonin family p	3.3	NA	NA
410	AT1G48520.1	|GATB | GLU-ADT subunit B | chr1:17	2.8	NA	NA
413	AT3G24430.1	|HCF101 | ATP binding | chr3:886873	3.2	NA	NA
414	AT3G11710.1	|ATKRS-1 | lysyl-tRNA synthetase 1	2.6	NA	NA
418	AT2G20420.1	| | ATP citrate lyase (ACL) family	2.1	NA	NA
423	AT2G16950.1	|TRN1, ATTRN1 | transportin 1 | chr	2.7	NA	NA
425	AT5G61790.1	|CNX1, ATCNX1 | calnexin 1 | chr5:2	2.1	2.2	NA
427	AT1G09780.1	| | Phosphoglycerate mutase, 2,3-bi	2.5	2.8	NA
428	AT3G59970.3	|MTHFR1 | methylenetetrahydrofolate	2.2	NA	NA
429	AT5G50950.2	|FUM2 | FUMARASE 2 | chr5:20729687-	2.3	NA	NA
434	AT1G69830.1	|ATAMY3, AMY3 | alpha-amylase-like	4.4	2.7	NA
443	AT2G05710.1	|ACO3 | aconitase 3 | chr2:2141591-	3.3	2.2	NA
445	AT5G58140.3	|PHOT2, NPL1 | phototropin 2 | chr5	2.0	NA	NA
449	AT3G10380.1	|SEC8, ATSEC8 | subunit of exocyst	3.4	NA	NA
451	AT2G27600.1	|SKD1, VPS4, ATSKD1 | AAA-type ATPa	3.3	NA	NA
452	AT4G24830.1	| | arginosuccinate synthase family	2.0	NA	NA
455	AT2G27860.1	|AXS1 | UDP-D-apiose/UDP-D-xylose s	3.0	2.0	NA
457	AT4G23940.1	| | FtsH extracellular protease fam	2.6	NA	NA
458	AT3G48720.1	| | HXXXD-type acyl-transferase fam	2.0	NA	NA
459	AT5G13280.1	|AK-LYS1, AK1, AK | aspartate kinas	2.6	NA	NA
463	AT3G51890.1	| | Clathrin light chain protein |	2.5	NA	NA
469	AT3G15730.1	|PLDALPHA1, PLD | phospholipase D a	2.3	NA	NA

	Accession.Number	Description	pair_1	pair_2	pair_3
471	AT3G53700.1	|MEE40 | Pentatricopeptide repeat (	1.7	NA	NA
481	AT4G03430.1	|STA1, EMB2770 | pre-mRNA splicing	2.2	NA	NA
483	AT4G11150.1	|TUF, emb2448, TUFF, VHA-E1 | vacuo	2.1	NA	NA
484	ATCG01130.1	|YCF1.2 | Ycf1 protein | chrC:12388	2.0	NA	NA
493	AT4G08850.1	| | Leucine-rich repeat receptor-li	3.1	2.9	NA
494	AT2G36850.1	|ATGSL08, GSL8, GSL08, ATGSL8, CHOR	2.3	NA	NA
495	AT1G02560.1	|CLPP5, NCLPP5, NCLPP1 | nuclear en	2.4	NA	NA
501	AT5G65620.1	| | Zincin-like metalloproteases fa	2.6	NA	NA
502	AT1G49750.1	| | Leucine-rich repeat (LRR) famil	2.1	NA	NA
504	AT2G05840.1	|PAA2 | 20S proteasome subunit PAA2	2.5	NA	NA
510	AT4G30580.1	|ATS2, EMB1995, LPAT1 | Phospholipi	1.7	NA	NA
512	AT1G10430.1	|PP2A-2 | protein phosphatase 2A-2	0.7	NA	NA
513	AT2G40840.1	|DPE2 | disproportionating enzyme 2	3.1	2.6	NA
515	AT2G44160.1	|MTHFR2 | methylenetetrahydrofolate	3.7	2.1	NA
516	AT3G44620.1 (+1)	| | protein tyrosine phosphatases;p	2.1	1.2	NA
520	AT3G23750.1	| | Leucine-rich repeat protein kin	3.2	3.0	NA
525	AT4G01330.2	| | Protein kinase superfamily prot	2.1	NA	NA
526	AT1G73600.2	| | S-adenosyl-L-methionine-depende	2.3	NA	NA
527	AT5G66470.1	| | RNA binding;GTP binding | chr5:	2.5	NA	NA
528	AT4G11420.1	|EIF3A, ATEIF3A-1, EIF3A-1, ATTIF3A	3.0	2.8	NA
529	AT1G26850.1 (+1)	| | S-adenosyl-L-methionine-depende	2.9	NA	NA
530	AT4G02510.1	|TOC159, TOC86, PPI2, TOC160, ATTOC	3.0	2.0	NA
531	AT2G47390.1	| | Prolyl oligopeptidase family pr	2.7	2.1	NA
533	AT3G48110.1	|EDD1, EDD | glycine-tRNA ligases |	2.5	NA	NA
536	AT3G52180.1	|ATPTPKIS1, DSP4, SEX4, ATSEX4 | du	2.3	NA	NA
543	AT2G28190.1	|CSD2, CZSOD2 | copper/zinc superox	2.2	NA	NA
545	AT1G65220.1	| | ARM repeat superfamily protein	2.2	NA	NA
547	AT1G20440.1	|COR47, RD17, AtCOR47 | cold-regula	2.5	NA	NA
548	AT3G16950.1	|LPD1, ptlpd1 | lipoamide dehydroge	3.1	2.6	NA
549	AT5G56760.1	|ATSERAT1;1, SAT5, SAT-52, SERAT1;1	1.4	NA	NA

	Accession.Number	Description	pair_1	pair_2	pair_3
556	AT4G32250.1 (+2)	| | Protein kinase superfamily prot	2.1	NA	NA
557	AT3G10920.2	|MSD1 | manganese superoxide dismut	2.9	NA	NA
561	AT1G47550.1	|SEC3A | exocyst complex component	2.2	NA	NA
562	AT4G35830.1	|ACO1 | aconitase 1 | chr4:16973007	3.8	2.5	NA
565	AT1G12000.1	| | Phosphofructokinase family prot	2.2	NA	NA
570	AT3G54760.1 (+1)	| | dentin sialophosphoprotein-rela	1.4	NA	NA
578	AT4G24770.1	|RBP31, ATRBP31, CP31, ATRBP33 | 31	2.0	NA	NA
579	AT3G54470.1	| | uridine 5'-monophosphate syntha	2.3	NA	NA
582	AT1G77590.1	|LACS9 | long chain acyl-CoA synthe	2.9	NA	NA
586	AT1G16880.1	| | uridylyltransferase-related | c	2.1	NA	NA
588	AT4G26300.1	|emb1027 | Arginyl-tRNA synthetase,	3.1	NA	NA
591	AT3G25690.1 (+1)	|CHUP1 | Hydroxyproline-rich glycop	2.3	NA	NA
594	AT5G54180.1	|PTAC15 | plastid transcriptionally	1.4	NA	NA
597	AT4G13930.1	|SHM4 | serine hydroxymethyltransfe	2.4	NA	NA
603	AT1G79920.1	| | Heat shock protein 70 (Hsp 70)	2.3	2.1	NA
605	AT2G23200.1	| | Protein kinase superfamily prot	2.7	2.1	NA
606	AT4G32400.1	|EMB104, SHS1, EMB42, ATBT1 | Mitoc	2.0	NA	NA
607	AT1G64090.1	|RTNLB3 | Reticulan like protein B3	0.7	NA	NA
610	AT3G46740.1	|TOC75-III, MAR1 | translocon at th	3.7	4.1	NA
611	AT3G23400.1	|FIB4 | Plastid-lipid associated pr	2.7	NA	NA
613	AT2G19520.1	|FVE, ACG1, MSI4, NFC4, NFC04, ATMS	1.7	NA	NA
620	AT1G29900.1	|CARB | carbamoyl phosphate synthet	2.7	NA	NA
622	AT3G19480.1	| | D-3-phosphoglycerate dehydrogen	3.5	NA	NA
624	AT2G41040.1	| | S-adenosyl-L-methionine-depende	1.7	NA	NA
626	AT3G01440.1	|PQL1, PQL2 | PsbQ-like 1 | chr3:16	2.6	NA	NA
627	AT1G74690.1	|IQD31 | IQ-domain 31 | chr1:280614	1.6	NA	NA
629	AT1G55160.3	| | unknown protein; FUNCTIONS IN:	2.5	NA	NA
630	AT1G22280.1	|PAPP2C | phytochrome-associated pr	2.1	NA	NA
636	AT5G25757.1 (+1)	| | RNA polymerase I-associated fac	2.4	2.1	NA
637	AT4G27070.1	|TSB2 | tryptophan synthase beta-su	1.6	1.2	NA

	Accession.Number	Description	pair_1	pair_2	pair_3
641	AT1G72730.1	| | DEA(D/H)-box RNA helicase famil	5.5	3.9	NA
642	AT3G23810.1	|SAHH2, ATSAHH2 | S-adenosyl-l-homo	2.9	NA	NA
644	AT4G01850.1 (+1)	|SAM-2, MAT2, SAM2, AtSAM2 | S-aden	2.2	NA	NA
647	AT1G76180.1 (+1)	|ERD14 | Dehydrin family protein |	2.5	2.9	NA
650	AT1G35720.1	|ANNAT1, OXY5, ATOXY5 | annexin 1 |	2.5	2.7	NA
651	AT5G15090.1 (+1)	|VDAC3, ATVDAC3 | voltage dependent	2.4	2.2	NA
652	AT1G52290.1	| | Protein kinase superfamily prot	3.8	2.5	NA
656	AT3G01280.1	|VDAC1, ATVDAC1 | voltage dependent	3.0	NA	NA
660	AT1G63940.4	|MDAR6 | monodehydroascorbate reduc	2.4	2.3	NA
662	AT1G74040.1	|IMS1, MAML-3, IPMS2 | 2-isopropylm	2.1	NA	NA
664	AT5G13650.2	| | elongation factor family protei	2.6	NA	NA
666	AT5G38990.1	| | Malectin/receptor-like protein	3.0	NA	NA
667	AT1G30230.1 (+1)	| | Glutathione S-transferase, C-te	2.2	NA	NA
668	AT3G52300.1	|ATPQ | ATP synthase D chain, mitoc	2.1	NA	NA
669	AT4G01800.1	|AGY1, AtcpSecA, SECA1 | Albino or	2.8	NA	NA
675	AT3G18130.1	|RACK1C_AT | receptor for activated	2.8	NA	NA
676	AT5G11670.1	|ATNADP-ME2, NADP-ME2 | NADP-malic	2.4	2.7	NA
681	AT1G29150.1	|ATS9, RPN6 | non-ATPase subunit 9	2.4	NA	NA
683	AT1G09620.1	| | ATP binding;leucine-tRNA ligase	3.1	NA	NA
686	AT2G44060.1 (+1)	| | Late embryogenesis abundant pro	2.5	NA	NA
687	AT1G16670.1	| | Protein kinase superfamily prot	2.7	NA	NA
688	AT2G40100.1	|LHCB4.3 | light harvesting complex	2.3	NA	NA
689	AT2G21870.1	|MGP1 | copper ion binding;cobalt i	2.3	NA	NA
690	AT5G42790.1	|PAF1, ATPSM30, ARS5 | proteasome a	2.7	2.2	NA
691	AT4G38740.1	|ROC1 | rotamase CYP 1 | chr4:18083	2.1	NA	NA
695	AT5G15650.1	|RGP2, ATRGP2 | reversibly glycosyl	2.2	NA	NA
696	AT2G35490.1	| | Plastid-lipid associated protei	2.1	NA	NA
698	AT5G14060.1 (+1)	|CARAB-AK-LYS | Aspartate kinase fa	2.6	NA	NA
699	AT1G30440.1	| | Phototropic-responsive NPH3 fam	1.7	NA	NA
701	AT4G24620.1	|PGI1, PGI | phosphoglucose isomera	2.6	2.0	NA

	Accession.Number	Description	pair_1	pair_2	pair_3
702	AT1G14610.1	|TWN2, VALRS | valyl-tRNA synthetas	2.2	NA	NA
704	AT4G29840.1	|MTO2, TS | Pyridoxal-5'-phosphate-	2.4	2.1	NA
705	AT1G78830.1	| | Curculin-like (mannose-binding)	2.3	NA	NA
707	AT5G04590.1	|SIR | sulfite reductase | chr5:131	2.1	NA	NA
708	AT3G20050.1	|ATTCP-1, TCP-1 | T-complex protein	2.7	2.0	NA
712	AT5G49810.1	|MMT | methionine S-methyltransfera	3.0	NA	NA
714	AT3G20390.1	| | endoribonuclease L-PSP family p	2.5	NA	NA
715	AT5G03320.1	| | Protein kinase superfamily prot	2.1	NA	NA
717	AT3G23300.1	| | S-adenosyl-L-methionine-depende	2.3	NA	NA
727	AT5G59880.1	|ADF3 | actin depolymerizing factor	2.4	NA	NA
729	AT2G42220.1	| | Rhodanese/Cell cycle control ph	2.0	NA	NA
730	AT3G18190.1	| | TCP-1/cpn60 chaperonin family p	3.2	NA	NA
733	AT1G04430.1 (+1)	| | S-adenosyl-L-methionine-depende	1.7	NA	NA
740	AT3G06580.1	|GAL1, GALK | Mevalonate/galactokin	2.5	NA	NA
743	AT3G11830.1	| | TCP-1/cpn60 chaperonin family p	2.9	NA	NA
744	AT1G33810.1	| | unknown protein; FUNCTIONS IN:	2.7	NA	NA
752	AT4G39080.1	|VHA-A3 | vacuolar proton ATPase A3	2.1	NA	NA
756	AT3G10060.1	| | FKBP-like peptidyl-prolyl cis-t	2.1	NA	NA
758	AT4G37000.1	|ACD2, ATRCCR | accelerated cell de	2.7	NA	NA
759	AT5G45620.1	| | Proteasome component (PCI) doma	2.1	NA	NA
763	AT3G05910.1	| | Pectinacetylesterase family pro	2.3	NA	NA
764	AT5G02240.1	| | NAD(P)-binding Rossmann-fold su	-1.4	NA	NA
765	AT5G66140.1	|PAD2 | proteasome alpha subunit D2	2.0	2.0	NA
766	AT5G20090.1 (+1)	| | Uncharacterised protein family	2.3	0.4	NA
767	AT2G43560.1	| | FKBP-like peptidyl-prolyl cis-t	2.3	2.0	NA
773	AT1G74910.1 (+1)	| | ADP-glucose pyrophosphorylase f	2.1	NA	NA
774	AT1G67280.1	| | Glyoxalase/Bleomycin resistance	0.7	2.3	NA
775	AT1G63770.5	| | Peptidase M1 family protein | c	2.7	NA	NA
778	AT1G13110.1	|CYP71B7 | cytochrome P450, family	2.3	NA	NA
779	AT5G47200.1	|ATRABD2B, ATRAB1A, RAB1A | RAB GTP	2.4	NA	NA

	Accession.Number	Description	pair_1	pair_2	pair_3
781	AT1G56050.1	| | GTP-binding protein-related | c	2.0	2.0	NA
783	AT5G26830.1	| | Threonyl-tRNA synthetase | chr5	2.4	NA	NA
786	AT4G01690.1	|PPOX, HEMG1, PPO1 | Flavin contain	2.3	NA	NA
787	AT4G16180.2	| | unknown protein; FUNCTIONS IN:	2.0	NA	NA
789	AT1G55480.1	|ZKT | protein containing PDZ domai	1.0	1.2	NA
793	AT2G35780.1	|scpl26 | serine carboxypeptidase-l	2.7	NA	NA
794	AT5G08530.1	|CI51 | 51 kDa subunit of complex I	2.6	NA	NA
796	AT3G62360.1	| | Carbohydrate-binding-like fold	2.0	NA	NA
803	AT5G11450.1	| | Mog1/PsbP/DUF1795-like photosys	2.1	NA	NA
808	AT3G57280.1	| | Transmembrane proteins 14C | ch	2.2	NA	NA
810	AT1G66150.1	|TMK1 | transmembrane kinase 1 | ch	0.0	NA	NA
815	AT5G58490.1	| | NAD(P)-binding Rossmann-fold su	2.1	NA	NA
828	AT4G36390.1	| | Methylthiotransferase | chr4:17	2.2	NA	NA
833	AT5G48230.2	|EMB1276, ACAT2 | acetoacetyl-CoA t	0.7	NA	NA
834	AT5G51110.1 (+1)	| | Transcriptional coactivator/pte	1.6	1.4	NA
838	AT5G48810.1	|ATB5-B, B5 #3, ATCB5-D, CB5-D | cy	0.7	NA	NA
839	AT2G33120.1	|SAR1, VAMP722, ATVAMP722 | synapto	1.4	NA	NA
840	AT5G04530.1	|KCS19 | 3-ketoacyl-CoA synthase 19	1.9	NA	NA
842	AT5G63620.1	| | GroES-like zinc-binding alcohol	1.0	NA	NA
844	AT4G35230.1	|BSK1 | BR-signaling kinase 1 | chr	0.4	1.0	NA
848	AT3G24570.1	| | Peroxisomal membrane 22 kDa (Mp	1.7	0.0	NA
849	AT3G47960.1	| | Major facilitator superfamily p	1.2	NA	NA
851	AT5G10780.1 (+1)	| | CONTAINS InterPro DOMAIN/s: Unc	1.7	NA	NA
852	AT3G56140.1	| | Protein of unknown function (DU	0.4	NA	NA
853	AT4G35860.1	|ATRABB1B, ATGB2, ATRAB2C, GB2 | GT	0.7	NA	NA
854	AT1G75130.1	|CYP721A1 | cytochrome P450, family	1.2	NA	NA
856	AT1G16890.2	|UBC36, UBC13B | ubiquitin-conjugat	0.7	NA	NA
858	AT3G57260.1	|BGL2, PR2, BG2, PR-2 | beta-1,3-gl	1.6	NA	NA
859	AT5G07190.2	|ATS3 | seed gene 3 | chr5:2237783-	1.4	NA	NA
861	AT5G13030.1	| | unknown protein; FUNCTIONS IN:	NA	2.7	NA

	Accession.Number	Description	pair_1	pair_2	pair_3
862	AT5G40370.1	| | Glutaredoxin family protein | c	NA	2.3	NA
864	AT1G23740.1	| | Oxidoreductase, zinc-binding de	NA	2.2	NA
865	AT3G24503.1	|ALDH2C4, ALDH1A, REF1 | aldehyde d	NA	2.2	NA
867	AT5G57655.2	| | xylose isomerase family protein	NA	2.3	NA
869	AT5G14260.1 (+2)	| | Rubisco methyltransferase famil	NA	2.0	NA
871	AT3G47800.1	| | Galactose mutarotase-like super	NA	0.7	NA
872	AT4G18440.1	| | L-Aspartase-like family protein	NA	2.6	NA
873	AT2G41530.1	|ATSFGH, SFGH | S-formylglutathione	NA	2.0	NA
874	AT4G01370.1	|ATMPK4, MPK4 | MAP kinase 4 | chr4	NA	2.1	NA
875	AT1G80380.4	| | P-loop containing nucleoside tr	NA	2.1	NA
876	AT2G31660.1	|SAD2, URM9 | ARM repeat superfamil	NA	2.3	NA
877	AT3G59020.2	| | ARM repeat superfamily protein	NA	2.1	NA
878	AT3G11400.2	|EIF3G1, ATEIF3G1 | eukaryotic tran	NA	2.2	NA
879	AT2G39960.1	| | Microsomal signal peptidase 25	NA	1.2	NA
881	AT5G27640.2	|TIF3B1, EIF3B, ATEIF3B-1, EIF3B-1,	NA	2.2	NA
882	AT5G15450.1	|APG6, CLPB3, CLPB-P | casein lytic	NA	2.4	NA
883	AT1G30580.1	| | GTP binding | chr1:10831953-108	NA	2.4	NA
885	AT5G01590.1	| | unknown protein; FUNCTIONS IN:	NA	2.8	NA
886	AT1G35160.1	|GRF4, 14-3-3PHI, GF14 PHI | GF14 p	NA	3.2	NA
887	AT2G46280.1 (+1)	|TRIP-1, TIF3I1 | TGF-beta receptor	NA	2.2	NA
889	AT5G22640.1	|emb1211 | MORN (Membrane Occupatio	NA	2.1	NA
892	AT4G14040.1	|EDA38, SBP2 | selenium-binding pro	NA	2.0	NA
893	AT2G39990.1	|EIF2, AteIF3f, eIF3F | eukaryotic	NA	2.2	NA
895	AT4G02420.1	| | Concanavalin A-like lectin prot	NA	0.0	NA
896	AT1G60710.1	|ATB2 | NAD(P)-linked oxidoreductas	NA	2.2	NA
897	AT5G43830.1	| | Aluminium induced protein with	NA	2.2	NA
898	AT5G11880.1	| | Pyridoxal-dependent decarboxyla	NA	2.0	NA
901	AT5G59890.1	|ADF4, ATADF4 | actin depolymerizin	NA	1.2	NA
903	AT4G30530.1	| | Class I glutamine amidotransfer	NA	0.0	NA

2.8 End

```
## THE END
```
